# An Automated Electrochemical Flow Platform to Accelerate Library Synthesis and Reaction Optimization

**DOI:** 10.1002/anie.202412045

**Published:** 2024-11-07

**Authors:** Eduardo Rial‐Rodríguez, Jason D. Williams, David Cantillo, Thomas Fuchß, Alena Sommer, Hans‐Michael Eggenweiler, C. Oliver Kappe, Gabriele Laudadio

**Affiliations:** ^1^ Institute of Chemistry, NAWI Graz,. Department University of Graz Heinrichstrasse 28 8010 Graz Austria; ^2^ Center for Continuous Flow Synthesis and Processing (CCFLOW) Research Center Pharmaceutical Engineering GmbH (RCPE) Inffeldgasse 13 8010 Graz Austria; ^3^ School of Chemistry and Molecular Biosciences The University of Queensland Brisbane, Queensland 4072 Australia; ^4^ Medicinal Chemistry and Drug Design Merck Healthcare KGaA Frankfurter Strasse 250 64293 Darmstadt Germany

**Keywords:** Automation, Electrochemistry, Library Synthesis, Electrocatalysis, Medicinal Chemistry

## Abstract

Automated batch and flow setups are well‐established for high throughput experimentation in both thermal chemistry and photochemistry. However, the development of automated electrochemical platforms is hindered by cell miniaturization challenges in batch and difficulties in designing effective single‐pass flow systems. In order to address these issues, we have designed and implemented a new, slug‐based automated electrochemical flow platform. This platform was successfully demonstrated for electrochemical C−N cross‐couplings of E3 ligase binders with diverse amines (44 examples), which were subsequently transferred to a continuous‐flow mode for confirmation and isolation, showing its applicability for medicinal chemistry purposes. To further validate the versatility of the platform, Design of Experiments (DoE) optimization was performed for an unsuccessful library target. This optimization process, fully automated by the platform, resulted in a remarkable 6‐fold increase in reaction yield.

## Graphical abstract

An automated electrochemical flow platform for operator‐free data generation is presented, leveraging slug‐based reaction mixtures for low material consumption. This platform was employed in the context of library synthesis of 44 medicinal chemistry‐relevant compounds. The platform was also used to optimize one previously unsuccessful target via DoE, leading to a 6‐fold yield increment.

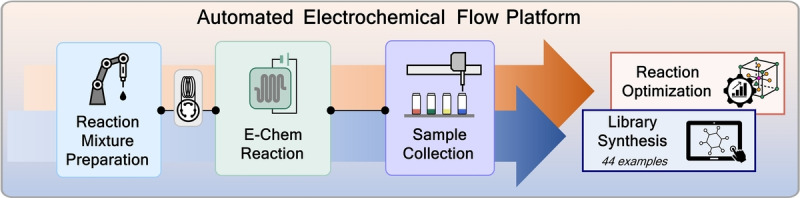

Automated platforms are emerging as valuable tools in organic synthesis due to their ability to gather significant amounts of data in a short period of time.[^1^, ^2^] Although most commonly used to accelerate ligand screening for catalytic reactions in well‐plate High Throughput Experimentation (HTE) systems, their application has rapidly expanded to numerous platform designs across all fields of organic chemistry.^[3]^ In particular, this technology is mainly used to generate large datasets for optimization purposes or for molecular libraries.^[4]^

Technological advancements in automation and reaction setups have led to the establishment of reliable thermal[^5^, ^6^, ^7^] and photochemical[^8^, ^9^, ^10^] HTE platforms, which are nowadays used routinely in pharmaceutical industries.[^1^, ^11^]

Parallel to the evolution of microbatch experimentation, the development of automated continuous‐flow platforms has become increasingly relevant.^[12]^ The translation of the small‐volume reaction concept to flow reactors has been successfully exploited, achieving reduced reaction times and scales compared to the traditional batch designs.[^13^, ^14^] Furthermore, the modular nature of flow setups has driven the development of compact, powerful, and versatile automated platforms.[^15^, ^16^, ^17^, ^18^] These systems employ reagent stock solutions to prepare reaction mixtures in an operator‐free manner, followed by precise control of reaction parameters in the reactor itself (e.g., temperature, reaction time and light irradiation), delivering highly reproducible data.[^12^, ^19^] While both batch and flow automated platforms have been effectively implemented in the context of thermal chemistry and photochemistry, the field of automated electrochemical platforms is still underdeveloped (Figure 1A).[^20^, ^21^]

**Figure 1:**
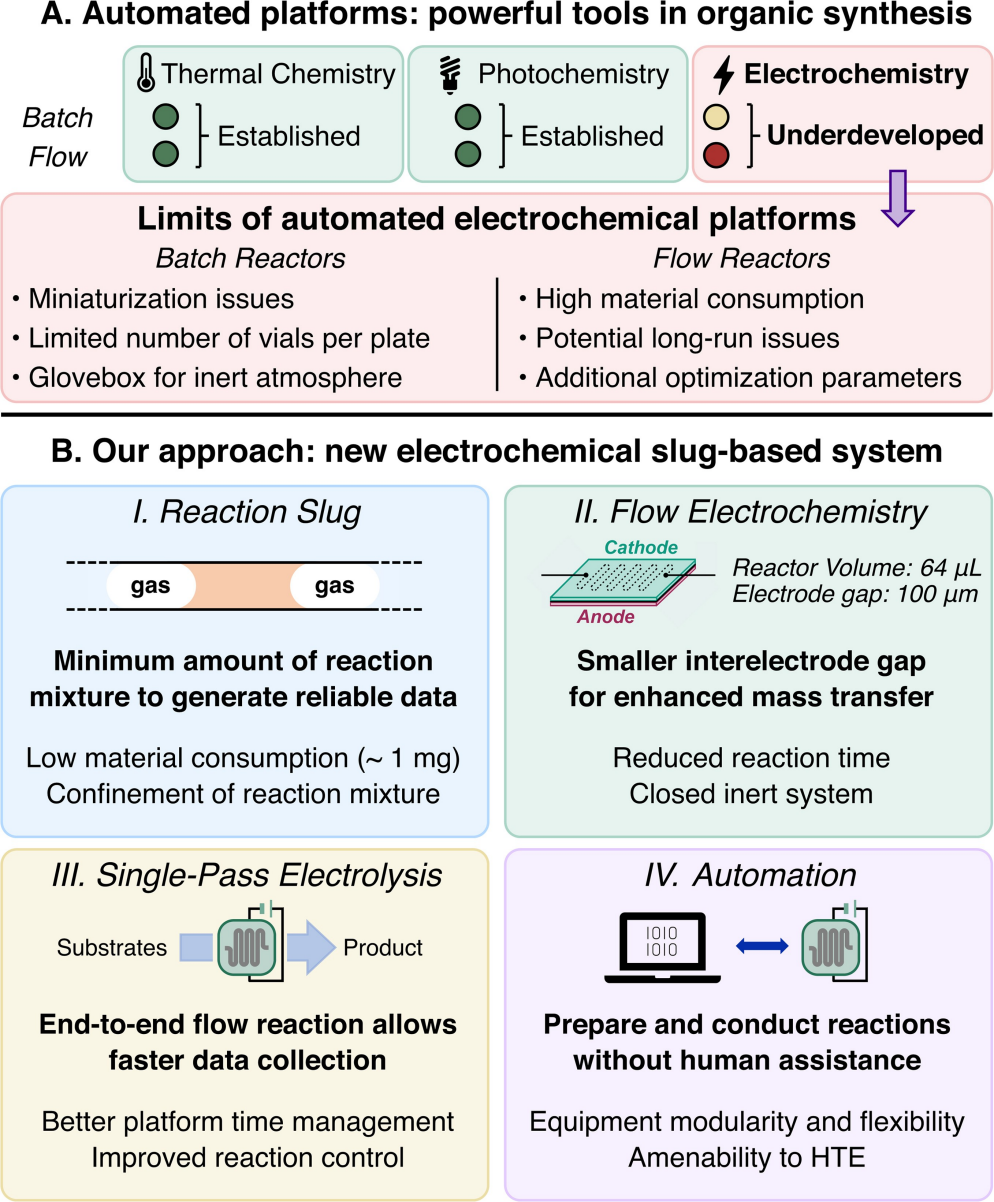
(A) State of the art of batch and flow chemical platforms. (B) Features of the novel automated electrochemical flow platform.

Electrochemistry has seen a recent surge of interest in organic synthesis, thanks to its innate sustainability and the ability to generate highly reactive intermediates under mild conditions.[^22^, ^23^, ^24^, ^25^] However, the design of the ideal automated electrochemical platform poses significant challenges.^[21]^ Batch electrochemical devices, inspired by the well‐plate construction, have shown great progress toward creating parallel screening systems.[^26^, ^27^, ^28^, ^29^, ^30^, ^31^, ^32^] Nevertheless, such batch systems face issues in the miniaturization of standard cells, especially for the incompatibility of fragile electrode materials. In addition, inert atmosphere can only be ensured when the reactions are executed in a glovebox.

On the other hand, electrochemical flow platforms present issues in processing small amounts of material, and potential fouling problems may remain undetected, as the electrode surface is enclosed within the cell. These problems are aggravated by the difficult transition from batch to single‐pass electrochemical reactions.^[33]^ An ‘end‐to‐end’ reactor assembly is desirable to ensure a straightforward and high throughput automated workflow. Highlighting these challenges, previous attempts to realize an automated electrochemical flow platform have been affected by non‐ideal reaction times,^[34]^ recirculation configurations,^[35]^ and material‐intensive continuous‐flow operation mode.[^36^, ^37^] Alternatively, unconventional electrochemical reactors have been proposed to obviate this problem, but their application is restricted to kinetic analysis of specific classes of reactions.^[38]^ Despite their remarkable contribution in the field, the previous examples lack in system flexibility as the implementation of different modules is not contemplated by design, workflow, or software employed, which are identified as highly desirable features.

Intrigued by the scientific challenges outlined above, we envisioned the development of a new electrochemical flow platform designed to deliver high quality data in a rapid and versatile way. Four key features were identified to address the engineering and electrochemical challenges (Figure 1B). Firstly, a gas‐separated reaction slug approach was selected to exclude dispersion and minimize material consumption.^[16]^ Secondly, a low volume electrochemical microreactor was chosen to minimize reaction times and enhance mass transfer, facilitating a reliable and efficient single‐pass configuration.[^39^, ^40^, ^41^] Next, a highly controllable electrochemical setup was considered to reduce the experiment time, maximizing the number of data points collected per day. Finally, a simple yet flexible software/hardware combination was adopted to enable the collection of different dataset types, including library synthesis as well as reaction optimization. Herein we report the development of an automated electrochemical flow platform that fully incorporates these features. It offers an innovative approach to generate reaction datasets without human intervention, employing straightforward instrumentation and single‐pass mode while maintaining precise control of all reaction parameters. The platform has been successfully applied to both library synthesis and reaction optimization via Design of Experiments (DoE). Both applications were demonstrated on a challenging C−N cross‐coupling methodology for the derivatization of E3 ligase binders,[^42^, ^43^, ^44^] toward linkers for targeted protein degrader molecules. This workflow aims to provide a streamlined way to generate medicinal chemistry relevant scaffolds via convenient electrochemical transformations, minimizing the material consumption for preliminary screenings and accelerating the structure survey process otherwise labor and time intensive.

The design and realization of the platform led to the installation depicted in the upper part of Figure 2. The system can be divided into four modules: the reaction mixture preparation zone, the electrolysis zone, and the sample collection zone, all overseen by the script controller. The workflow begins in the reaction mixture preparation zone, where a 256 μL reaction solution is prepared by sequentially aspirating stock solutions of each reagent. Efficient mixing of the reaction slug is ensured by aspirating each component into a section of tubing with a large internal diameter (3.18 mm).[^15^, ^38^] Once the reaction mixture has been prepared, an argon stream pushes the reaction slug, injecting it into a sample loop (1.0 mL) via a six‐port valve. The slug is then conveyed to the electrochemical reaction zone by a solvent stream.

**Figure 2:**
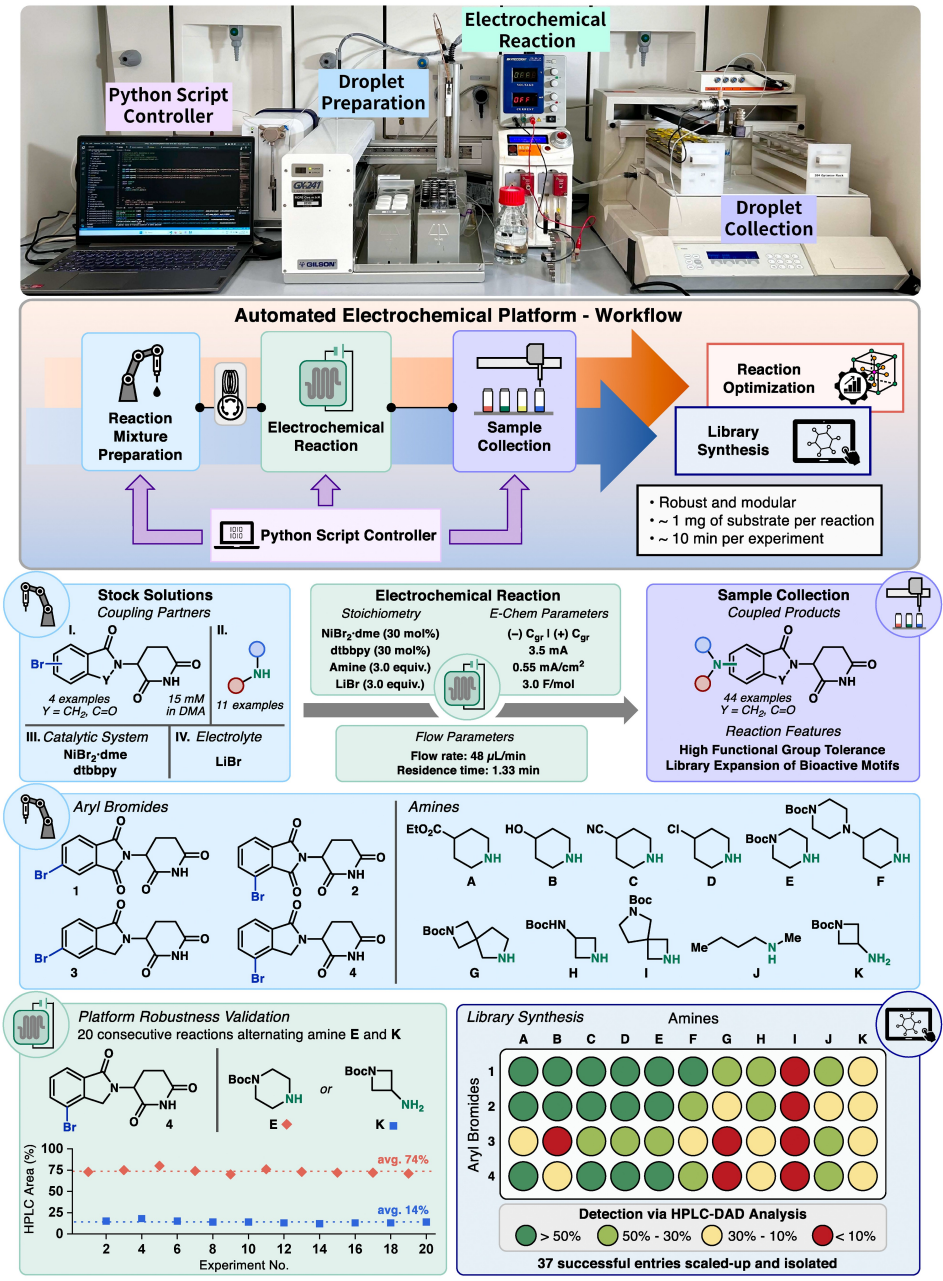
(Top) Picture of the setup and workflow schematization. (Center) Stock solutions, electrochemical reaction conditions, and reagents for the library synthesis. (Bottom) Platform robustness and library synthesis.

Upon filling the reactor, the solvent pump flow rate is switched from 1.0 mL/min to 48 μL/min to achieve the desired residence time and the power supply activates, initiating the electrochemical reaction. Once the electrolyzed slug exits the reactor, the mixture enters the collection zone, where an aliquot is delivered to a collection vial by the fraction collector. Throughout the experiment, the script controller orchestrates the entire process, activating each module with precise timing. This intuitive Python script requires minimal inputs to initiate the experimental run: the user simply inputs rack positions and equivalents of each reagent along with the desired electrochemical reaction parameters. With this protocol, each experimental datapoint is collected in approximately 10 minutes, and the material consumed is around 1 mg per reagent.

The described workflow not only aligns perfectly with the ideal features proposed at the beginning of our investigation, but also results in a highly reproducible system, facilitated by the commercial availability of all the instrumentation controlled by the script. In addition, the high modularity and versatility of the system allow for on‐demand modification of the apparatus. For the core of the platform, a simple parallel plate reactor with small interelectrode gap (100 μm) and reactor volume (64 μL) was chosen.^[40]^ This decision was taken to enhance design flexibility, avoiding the need for complex machining of the electrodes or any specialized and over‐engineered design. Following this principle, phase sensors were considered unnecessary as the slug progression through the platform is dictated by time spent in each zone.

After the successful installation and preliminary testing of the platform, we proceeded to select a suitable electrochemical transformation that could validate our system. Due to the immense current interest in targeted protein degraders in medicinal chemistry,[^45^, ^46^] we aimed to generate a library of protein degrader fragments based on cereblon (CRBN) binders (thalidomide and lenalidomide). This provided the perfect opportunity to leverage electrochemistry as a sustainable enabling technology to forge C−N bonds on the arene ring (Figure 2, Center). Among the potential synthetic approaches, the electrochemical, Ni‐catalyzed, C−N cross‐coupling previously reported by Baran and co‐workers[^47^, ^48^] was identified as an ideal starting point. This transformation posed a great challenge as it required the engagement of both electrodes by the transition metal catalyst under strictly inert atmosphere, hence it was considered a difficult test to validate our platform.^[47]^ After revisiting the reaction conditions, adjustments were made to transfer the protocol to single‐pass flow operation with the designated (poorly soluble and base‐sensitive) coupling partners (see Supporting Information for adjusted reaction conditions).

Interestingly, during the reoptimization with these compounds, better performance was observed in flow compared to the batch system. Nevertheless, our new flow conditions were found also directly transferable to IKA Electrasyn 2.0 batch reactions.

Thereafter, 4 different aryl bromides and 11 amines were chosen to apply the reoptimized conditions in the generation of a library. The aryl bromides (**1**–**4**) encompass both thalidomide and lenalidomide binders, both with two different exit vectors for linker attachment. Meanwhile the selected amines (**A**–**K**) cover a broad range of functional group handles for further linker expansion in a drug discovery setting. Furthermore, the structures encompass a high degree of saturation, a diverse range of rigidities and expansion vectors, which play a central role in linker design.

Prior to initiating the library synthesis, we assessed whether our system could sustain prolonged operation over an extended time period. This evaluation is important as it is not uncommon to observe a loss in performance in electrochemical microreactors during prolonged operation time. Hence, a robustness validation experiment was designed, selecting 2 reactant combinations that showed good and modest performance: **4E** and **4K**, respectively. A sequence of 20 experiments was conducted by alternating the synthesis of the two compounds (Figure 2, Bottom left). After approximately 4 hours of continuous experimentation, the platform delivered consistent results with negligible variation in the observed product formation, confirming the operational stability of the automated platform.

It is noteworthy that no cross‐contamination between different samples was observed, as the slug is effectively isolated within each reaction. Additionally, a simple reactor solvent flush for 30 seconds after sample collection proved to be sufficient to maintain the electrochemical performance of the reactor, avoiding any electrode fouling. This positive observation can be attributed to the relatively low concentration of the reaction mixtures (15 mM of limiting reagent) and short reaction time (4 min), which helps mitigate the deposition of material at the electrodes.

Following these initial tests, the 44‐member library synthesis proceeded smoothly on the automated electrochemical flow platform, with all reactions completed within 8 hours of continuous operation under inert conditions without the necessity of using a glovebox. Out of the 44 entries, high amounts of products were detected in 15 (>50 %), good amounts in 11 (50–30 %), moderate amounts in another 11 (30–10 %), and only 7 entries showed low amounts or none of the desired product (<10 %) (Figure 2, Bottom right).

All of these results were confirmed by transferring slug flow conditions to a continuous‐flow protocol (0.15–0.30 mmol) and isolating all the library products, delivering in all cases a sufficient amount for medicinal chemistry purposes (~10–60 mg). Notably, the results observed in the continuous‐flow reactor were in excellent agreement with those from the slug flow experiments, highlighting the applicability and transferability of the developed automated platform. This dataset also provided valuable insights to reactivity trends. In particular, aryl bromide **3** proved to be the most challenging to couple with the amine set, while the other aryl bromides exhibited similar outcomes across the scope. Concerning the amines, piperidine derivatives containing different functional groups showed reliable reactivity and robust product formation. Simple azetidines, acyclic secondary amines, and primary amines were coupled providing serviceable amounts of products in all cases. On the contrary, spirocyclic amines **G** and **I** consistently showed poor results in all cases, indicating an incompatibility with these conditions.

In a medicinal chemistry setting, scenarios similar to the unsuccessful coupling of **G** and **I** are quite commonly encountered. Synthetic methodologies often have limited substrate compatibility, particularly when molecular complexity increases. In most cases, such entries are simply excluded from drug analog campaigns due to the laborious nature of further optimization, with no guaranteed success. In this context, having an automated platform that conducts microscale reactions with high precision can be a powerful resource for filling gaps in mapping selected chemical space. Leveraging the simplicity of our workflow, we envision that a rapid reaction survey via screening parameters in a straightforward manner could aid the medicinal chemists in overcoming preparation of hardly accessible compounds.

To prove that our electrochemical system can accomplish the task of collecting on‐demand datasets in a short timeframe, we optimized the previously unsuccessful coupling between **4** and **G** (6 % of product observed) via DoE (Figure 3, Top). Four key reaction parameters were studied: catalyst loading, amine loading, current, and applied charge. Once the conditions for all the experiments were generated, data points were collected by the automated platform. The 23 results necessary for the DoE optimization were promptly carried out in approximately 4 hours. From these results, a multiple linear regression (MLR) model was fitted, with excellent agreement between the predicted and observed values (R^2^=0.950, Figure 3, Center left). This quantitative model can be used to predict the outcome of any set of input conditions (see surface plot, Figure 3, Center right). The data revealed that catalyst loading, and current were the most impactful factors on the success of the reaction. Specifically, an optimum catalyst concentration and low current were crucial to maximize the product formation. Applying the final optimized conditions, product **4G** could be observed in good amounts (35 %), approximately 6 times higher than the unsatisfactory results obtained with the initial library protocol (6 %, Figure 3, Bottom). With the knowledge gained, seven additional reactions were performed, but better results were not obtained. Overall, the synergistic combination of DoE and automated data collection exemplifies the versatility of our platform, whose rapid data collection can accelerate the synthesis of complex medicinal chemistry relevant scaffolds via electrochemistry.

**Figure 3:**
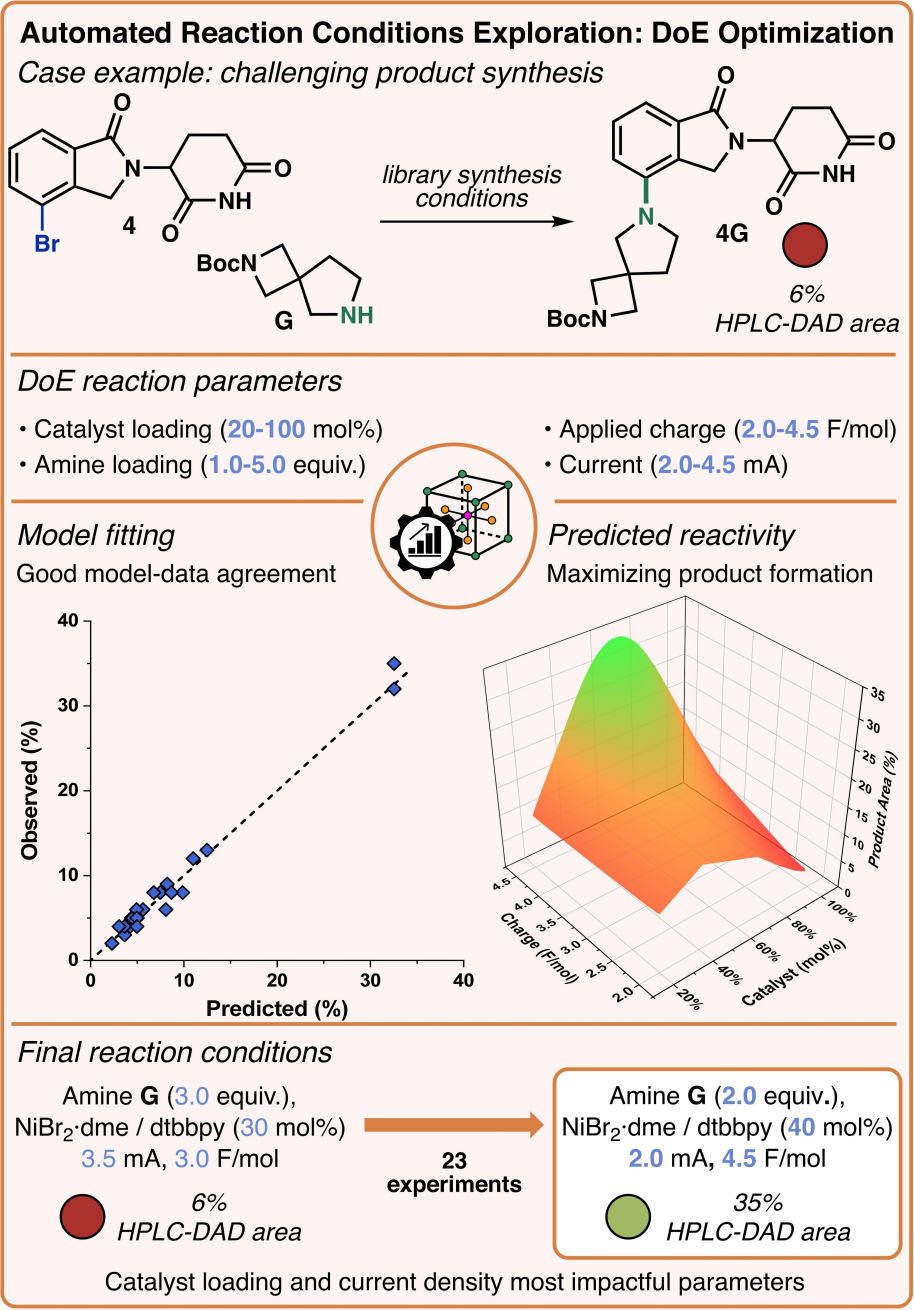
(Top) Case study for reaction optimization from the library synthesis, with parameters examined using DoE. (Center) Fit of reaction model (predicted vs observed) resulting from DoE optimization and model surface plot. (Bottom) Comparison of initial and final reaction conditions.

In conclusion, we have presented the successful development and application of an automated electrochemical flow platform, showcasing the unique features of our system in generating relevant datasets for drug discovery purposes. Through its application to the synthesis of targeted protein degraders via electrocatalytic C−N cross‐coupling, the platform demonstrated the capability to execute reactions over a long period with high reproducibility and no loss in performance. A 44‐member compound library was then synthesized without human intervention. Optimization of one target from this library (**4G**) via DoE led to a remarkable 6‐fold increase of product formation, highlighting the versatility and effectiveness of the platform. We anticipate that the modular nature of our apparatus will create opportunities to integrate process analytical technology (PAT) tools, further enhancing its capabilities. Overall, this system and approach will have a marked impact on the technological evolution of medicinal chemistry, by bridging organic electrochemistry HTE to the fore.

## Supporting Information

The authors have cited additional references within the Supporting Information.[^49^, ^50^]

## Conflict of Interests

The authors declare no conflict of interest.

## Supporting information

As a service to our authors and readers, this journal provides supporting information supplied by the authors. Such materials are peer reviewed and may be re‐organized for online delivery, but are not copy‐edited or typeset. Technical support issues arising from supporting information (other than missing files) should be addressed to the authors.

Supporting Information

## Data Availability

The data that support the findings of this study are available in the supplementary material of this article.
