# Supplementary material for: An Automated Electrochemical Flow Platform to Accelerate Library Synthesis and Reaction Optimization
Source: Angew Chem Int Ed Engl. 2024 Nov 7;63(51):e202412045. doi: 10.1002/anie.202412045 (PMC11627123; doi:10.1002/anie.202412045)

Angewandte Chemie
Eine Zeitschrift der Gesellschaft Deutscher Chemiker

Supporting Information

An Automated Electrochemical Flow Platform to Accelerate Library Synthesis and Reaction Optimization

E. Rial-Rodríguez, J. D. Williams, D. Cantillo, T. Fuchß, A. Sommer, H.-M. Eggenweiler, C. O. Kappe*, G. Laudadio**

An Automated Electrochemical Flow Platform to Accelerate Library Synthesis and Reaction Optimization

Eduardo Rial-Rodríguez[a,b], Jason D. Williams[a,b], David Cantillo[a,b,c], Thomas Fuchß[d], Alena Sommer[d], Hans-Michael Eggenweiler[d]*, C. Oliver Kappe[a,b]*, and Gabriele Laudadio[a,b]*

[a] Institute of Chemistry, University of Graz, NAWI Graz, Heinrichstrasse 28, 8010 Graz, Austria.
[b] Center for Continuous Flow Synthesis and Processing (CCFLOW), Research Center Pharmaceutical Engineering GmbH (RCPE), Inffeldgasse 13, 8010 Graz, Austria.
[c] School of Chemistry and Molecular Biosciences, The University of Queensland, Brisbane, Queensland 4072, Australia.
[d] Medicinal Chemistry and Drug Design, Merck Healthcare KGaA, Frankfurter Strasse 250, 64293 Darmstadt, Germany.

Correspondence to *gabriele.laudadio@uni-graz.at, oliver.kappe@uni-graz.at, michael.eggenweiler@merckgroup.com

Supporting Information

Table of contents

1. General Experimental Information 3
2. Electrochemical Platform 4
   Platform Schematization 4
3. Python Script Workflow 7
4. Reaction Slug Preparation and Sampling 9
5. Synthetic Procedures and Characterization 10
   General Procedure 1: Library Synthesis 10
   General Procedure 2: Preparative Scale 11
6. Library Synthesis with the Automated Platform 13
   Results Summary 13
   Library Synthesis Chromatograms 14
7. Design of Experiments (DoE) 40
   Software and Experimental Design 40
   Experimental Results 41
   Supplementary Experiments for Reaction Optimization 44
8. Adjustment of the Reaction Conditions 46
   Batch Experimentation (ElectraSyn 2.0) 46
   Continuous-Flow Experimentation 48
   Reaction Conditions Comparison 49
9. Compound Characterization 50
10. References 69
11. NMR Spectra 70

1. General Experimental Information

For preparative and automated experiments, all reactions were carried out under an inert argon atmosphere with dry solvents under anhydrous conditions unless otherwise stated. Solvents and reagents were either purchased of the highest commercial quality, and used without further purification, unless otherwise stated. The bromo lenalidomide (aryl bromide **1** and **2**) and bromo thalidomide (aryl bromide **3** and **4**) substrates were synthesized by researchers at Merck KGaA, based on published procedures.[1,2]

HPLC analysis was performed using a Shimadzu LC20 system. LC-MS analysis were performed on a Shimadzu HPLC system, equipped with a Shimadzu LCMS-QP2020 instrument.

The flow equipment, including the reactor, is described in detail in Section 2.

2. Electrochemical Platform

Platform Schematization

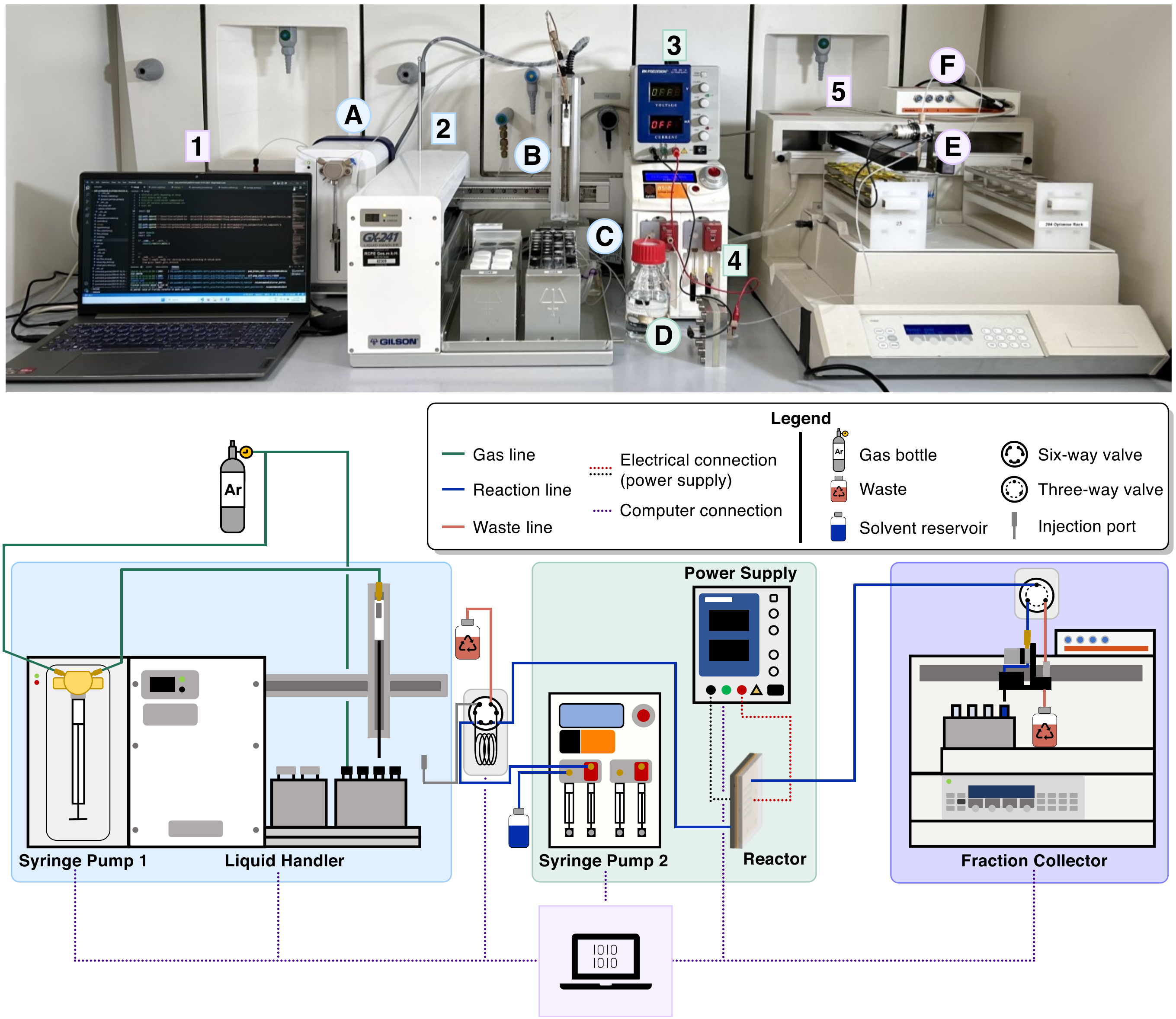

Figure S1. Top: photograph of the flow electrochemistry automated platform. Bottom: scheme of the platform showing all the tubing and cable connections.

All the equipment used – except for the flow electrolysis cell (*D* in Figure S1)- is commercially available. The simplicity of the platform allows it to be modular and reproducible. It contains the following elements:

- **Liquid handler, Gilson, GX-241** (*2* in Figure S1): used for preparing the reaction mixture. It is connected to the computer via RS-232 and communication is performed using a custom driver, written in python. The liquid handler includes three different modules:

  - **Single-syringe pump** (*A* in Figure S1): coupled with an inbuilt three-way-valve, it allows solutions to aspirated and injected through the needle. The inlet of the pump is connected to a flask with an argon balloon, so the reaction slug is created under inert conditions.
  - **Probe arm** (*B* in Figure S1): equipped with a steel needle, it can reach any position XYZ within the sample racks that contain the reagent stock solutions. A mixing unit consisting on a PFA tube with larger diameter (6 cm long, 1/8“ ID) is connected to the top of the needle in order to ensure that the reaction slug is well mixed, prior to injection. The fluidic connections for the 1/8“ ID tubing were achieved using IDEX-HS 5/16-

24 flat-bottom PEEK fittings for 3/16" OD tubing (IDEX Part Number: XP-137). For the 1/32“ ID tubing, IDEX-HS 1/4-28 - 1/8 PEEK fittings (IDEX Part Number: P-948) were used.

- **Six-way injection valve** (*C* in Figure S1): the “load” position allows the probe arm to load the reaction slug into the sample loop (1 mL, 1/32" OD). Then, the “inject” position allows the Syrris syringe pump (Syringe Pump 2) to transport the slug from the sample loop to the flow electrolysis cell. The fluidic connections were achieved using IDEX-HS coned PEEK fittings for 1/16" OD tubing (IDEX Part Number: F-130X).

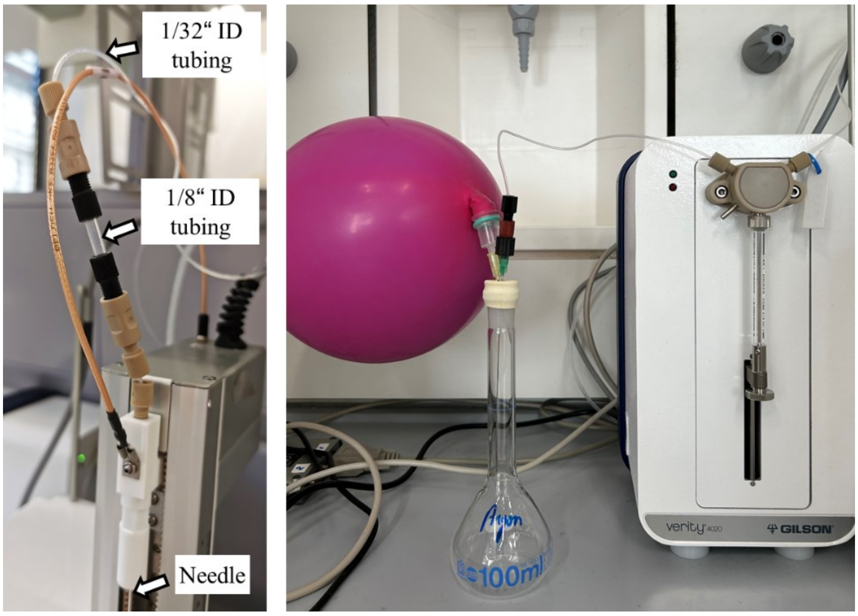

Figure S2. Left: photograph of the mixing unit of the probe arm. Right: three-way-valve of the single-syringe pump connected to a volumetric flask filled with argon ensuring that the system is kept under inert conditions

- **Power supply, BK Precision, 1739** (*3* in Figure S1): used for applying current to the flow electrochemical cell during the reaction and is connected with the computer via RS-232. Communication is performed using a custom driver, written in python.

- **Syringe pump, Syrris, Asia Syringe Pump** (*4* in Figure S1): used for transporting the reaction slug to the reactor, applying the desired flow rate during the reaction, transporting the slug after the reaction to the fraction collector for collection and cleaning the tubing and the reactor. This pump conveys only the reaction solvent (dimethylacetamide). Since the reaction slug is isolated by gas at both ends, dispersion into the carrier solvent does not occur. This syringe pump is connected with the computer via USB and the commands are sent to the “Syrris External Control” application via an OPC UA server (corresponding OPC UA client written in python).

- **Flow electrolysis cell** (*D* in Figure S1): parallel plate reactor developed previously in the group[3] where the electrochemical reaction takes place. The electrodes used are both impervious graphite (FC-GR347B, Graphtek LLC, 50 × 50 mm). A laser-cut Mylar foil is used as electrode separator/reaction channel and it presents a thickness of 100 μm and a surface area of 6.4 cm^2, creating a reactor volume of 64 μL.

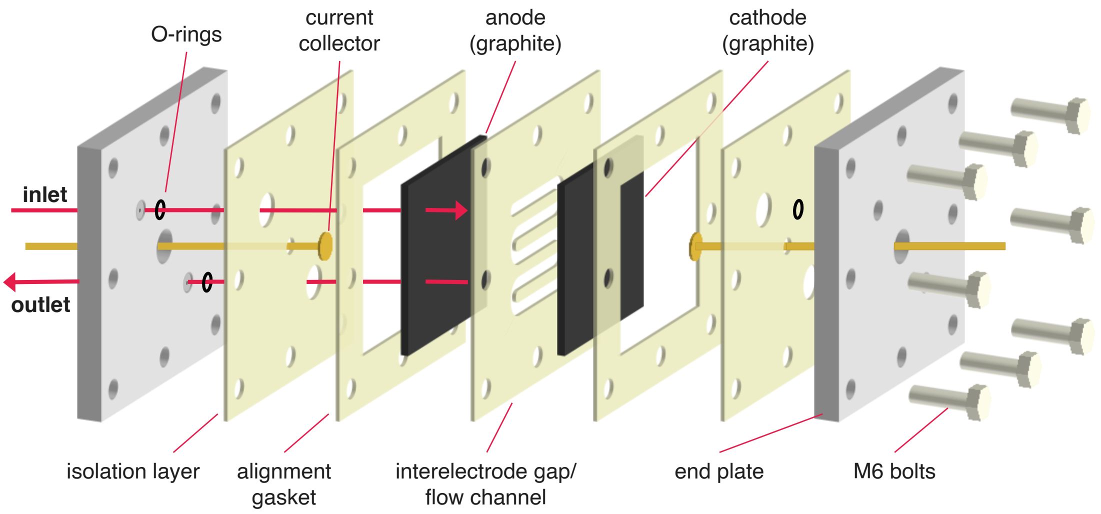

Figure S3. Exploded view of the flow electrolysis cell and details on the interelectrode separator. Images reproduced from reproduced from ref 3 under CC BY-NC-ND.

- **Fraction collector, Syrris, Asia Automated Collector Regular** (*5* in Figure S1): used for the collection of a representative sample from the reaction slug. The robotic arm has a three-way-valve ("E" in Figure S1) that can switch to "collect" or "waste" position. In order to send commands from the computer, it needs to be connected to the **automator Syrris, Asia Automator** (*F* in Figure S1). The fraction collector is connected via a custom 9-pin cable to the automator and the automator is connected via USB to the computer. Commands are sent to the "Syrris External Control" application via an OPC UA server (corresponding OPC UA client written in python).

3. Python Script Workflow

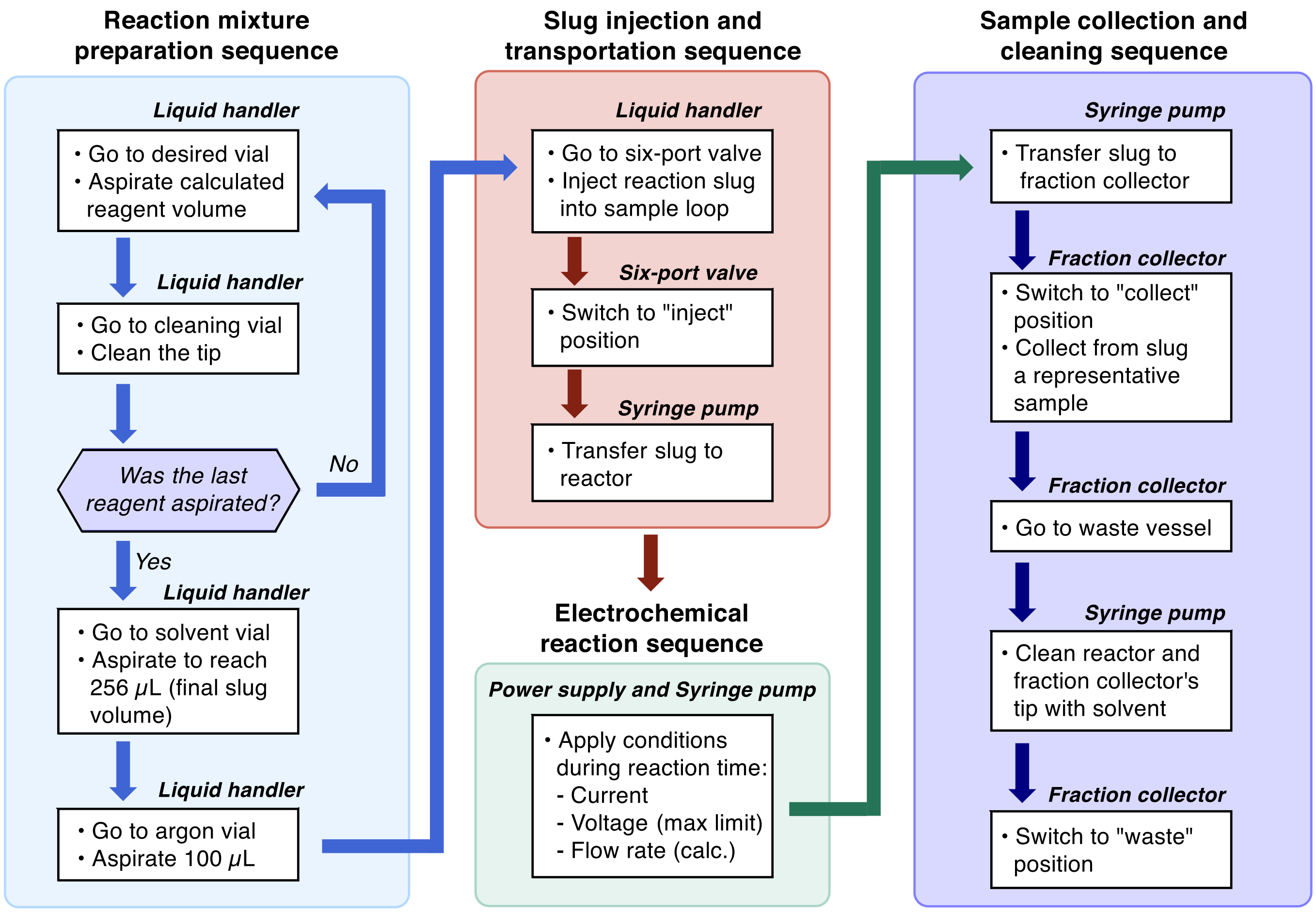

Figure S4. Workflow of the Python script for generating the reaction mixture, transporting the slug to the reactor, performing an electrochemical reaction, and collecting the representative product section.

The sequence has four main units: 1. *Reaction mixture generation*, 2. *Slug injection and transportation*, 3. *Electrochemical reaction* and 4. S*ample collection and cleaning*.
The concentration of all the stock solutions must be specified in a list, together with the desired concentration of the main reagent (aryl bromide in this case) in the final reaction mixture.
The main function that starts the experiment sequence is "automated_run". The function must be written and awaited (asynchronous programming is used, based on the asyncio python package) for each experiment sequence (44 times in the case of the library synthesis):

```
async def initialize():
    await automated_run (tube, concentration_stock_sol_reagents, reagents_list,
    equiv_reagents, position_main_reagent, current, charge)
```

The arguments required to run this function are:

- **tube**: which vial position in the fraction collector will be used for collection.
- **concentration_stock_sol_reagents**: list of all stock solutions and their concentrations. This remains constant for all automated experiments once the stock solutions are prepared.
- **reagents_list**: list of the positions of the reagents that will be used for preparing the reaction slug.
- **equiv_reagents**: list of the molar equivalents of each reagent for preparing the reaction slug. From the stock solution concentrations, the program calculates the required volume. A dictionary is generated connecting the positions of the reagents to be aspirated and their corresponding volumes.

- **position_main_reagent**: the position of the main (limiting) reagent is specified in a separate argument. In this work, the aryl bromide component is always the limiting reagent, but this can be changed as necessary for different reactions.
- **current** (optional): extra argument introduced for the DoE experiments.
- **charge** (optional): extra argument introduced for the DoE experiments.

4. Reaction Slug Preparation and Sampling

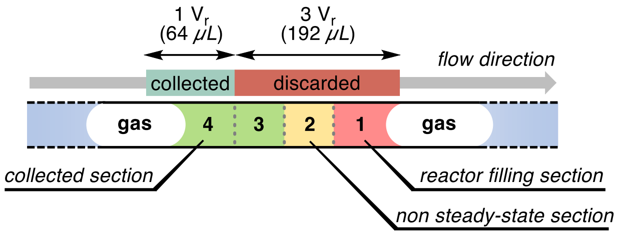

Figure S5. Schematic representation of the reaction slug, which is isolated by gas at both ends.

By having a slug volume of 256 µL (four times the reactor volume), it is ensured that the reaction achieves steady-state conditions. In order to ensure that a representative sample of the reaction slug is analysed, only the section 4 is collected with the fraction collector. All the other sections are discarded by using the "waste" position of its three-way valve.

Transportation of the reaction slug through the platform is performed based on time, thus no phase sensors are needed.

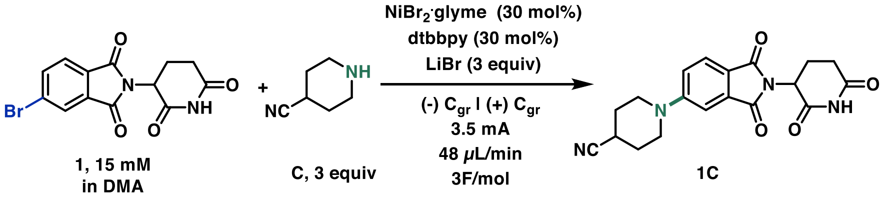

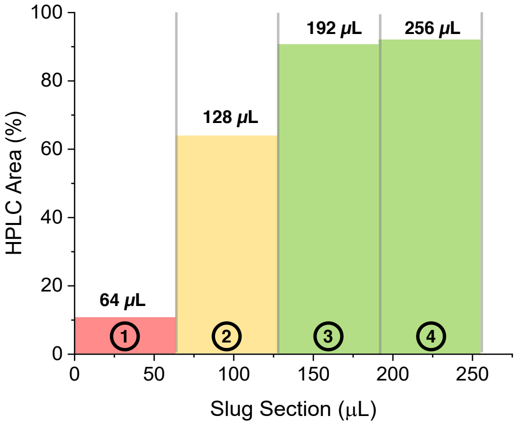

Slug Section	HPLC Area DP (Area %)
1	11
2	63
3	90
4	91

Figure S6. Analysis of the conversion at different points of the reaction slug using a model reaction

Since 4 min is required for the entire reaction slug to pass through the reactor, a sample was taken during each minute to compare the extent of reaction (product area %) within each section of the slug. Section 1 is used to fill the reactor before the reaction starts. Once the reactor is filled with reaction mixture, the power supply starts to apply current and the electrochemical reaction starts. Because this segment does not receive the full electrolysis residence time, low amounts of product are observed. Section 2 presents a higher amount of product but still does not reach the representative reaction performance. Finally, the last sections 3 and 4 both present a similar amount of product, confirming that representative conditions are reached. Only section 4 is collected in order to be sure that section 2 does not diffuse or contaminate the representative sample of the reaction.

5. Synthetic Procedures and Characterization

General Procedure 1: Library Synthesis

Stock solutions preparation

The following stock solutions were prepared for the automated library synthesis. In total, 23 vials were needed:

- **Aryl bromide**: to a 4 mL vial, the corresponding aryl bromide (0.090 mmol, 0.030 M) was added in dimethylacetamide (3 mL) and sonicated until it was completely solubilized. Then, the solution was dried over 3 Å molecular sieves and purged with argon. In total, 4 solutions with different aryl bromides (**1** to **4**) were prepared.

- **Amine**: to a 4 mL vial, the corresponding amine (0.90 mmol, 0.30 M) was added in dimethylacetamide (3 mL) and sonicated until it was completely solubilized. Then, the solution was dried over 3 Å molecular sieves and purged with argon. In total, 11 solutions with different amines (**A** to **K**) were prepared.

- **Catalytic system**: to a 4 mL vial, $NiBr_2$·dme (46 mg, 0.15 mmol, 0.050 M) and 4,4′-di-*tert*-butyl-2,2′-dipyridyl (40 mg, 0.15 mmol, 0.050 M) were added in dimethylacetamide (3 mL) and sonicated until they were completely solubilized. Then, the solution was dried over 3 Å molecular sieves and purged with argon. In total, 4 different solutions were prepared for the library synthesis (one per aryl bromide) in order to avoid deactivation of just one stock solution over time.

- **Electrolyte**: to a 4 mL vial, lithium bromide (78 mg, 0.90 mmol, 0.30 M) was added in dimethylacetamide (3 mL) and sonicated until it was completely solubilized. Then, the solution was dried over 3 Å molecular sieves and purged with argon. Two solutions were prepared.

- **Solvent vials**: two 4 mL vials were filled with dry dimethylacetamide and purged with argon. One of them was used for making up the total volume of 256 µL of reaction mixture. The other vial was used to clean the tip of the liquid handler between aspirations of reagents. Only one vial was needed for each function.

Automated experiments

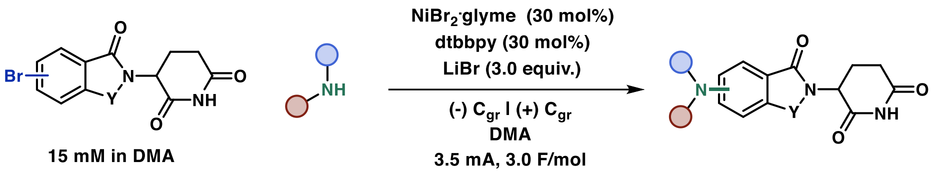

A 256 µL reaction slug containing the corresponding aryl bromide (1.0 eq, 0.015 M), the corresponding amine (3.0 eq), LiBr (3.0 eq), $NiBr_2$·dme (30% mol), 4,4′-di-*tert*-butyl-2,2′-dipyridyl (30% mol) in dimethylacetamide was automatically prepared and injected to a sample loop by the liquid handler. Then, the slug was transported using a syringe pump (Syrris, Asia) by pumping dimethylacetamide until the flow electrolysis cell was filled with reaction mixture. Then, a constant current of 3.5 mA and a flow rate of 48 µL/min were applied. After 4 min, the reaction was completed and the slug was transported to the fraction collector. Once the desired fraction of the slug had been collected, the syringe pump continued pumping solvent for 30 s in order to clean the reactor, tubing and tip of the fraction collector.

General Procedure 2: Preparative Scale

For the preparative scale, two different procedures were applied depending on the result obtained during the library synthesis. For those reactions that gave >35% HPLC area, procedure A was applied. For the rest that yielded a lower result, procedure B was applied.

Procedure A:

To a 10 mL volumetric flask, the corresponding aryl bromide (1.0 eq), the corresponding amine (3.0 eq), LiBr (3.0 eq), $NiBr_2$·dme (30% mol) and 4,4′-di-*tert*-butyl-2,2′-dipyridyl (30% mol) were added and the flask was filled to the mark with dimethylacetamide (resulting in 0.015 M concentration of aryl bromide). Then, the resulting mixture was sonicated until all the reagents were completely solubilized. Then, the solution was dried over 3 Å molecular sieves and purged with argon.

The reaction mixture was pumped through the flow electrolysis cell using a syringe pump (Syrris, Asia) under inert conditions until it was filled. Then, a constant current of 3.5 mA and a flow rate of 48 µL/min was applied. Once steady-state conditions were reached, the resulting reaction mixture was collected in a vial at the outlet of the reactor for 2-3 h.

The collected reaction mixture was quenched with aqueous citric acid solution (5% w/w, 1 × 30 mL) and extracted with EtOAc (3 × 20 mL) or DCM:MeOH 3:1 (3 × 20 mL) for the compounds that had a higher solubility in water. The combined organic layers were washed with brine (2 × 30 mL), dried over Na_2SO_4 anhydrous, filtered and evaporated under reduced pressure. The desired product was purified from the crude mixture via SFC or prep-HPLC (see compound characterization section for isolation details).

Procedure B:

To a 20 mL volumetric flask, the corresponding aryl bromide (1.0 eq), the corresponding amine (3.0 eq), LiBr (3.0 eq), $NiBr_2$·dme (30% mol) and 4,4′-di-*tert*-butyl-2,2′-dipyridyl (30% mol) were added and the flask was filled to the mark with dimethylacetamide (resulting in 0.015 M concentration of aryl bromide). Then, the resulting mixture was sonicated until all the reagents were completely solubilized. Then, the solution was dried over 3 Å molecular sieves and purged with argon.

The reaction mixture was pumped through the flow electrolysis cell using a syringe pump (Syrris, Asia) under inert conditions until it was filled. Then, a constant current of 3.5 mA and a flow rate of 48 µL/min was applied. Once steady-state conditions were reached, the resulting reaction mixture was collected in a vial at the outlet of the reactor for 4-6 h.

The collected reaction mixture was quenched with aqueous citric acid solution (5% w/w, 1 × 60 mL) and extracted with EtOAc (3 × 40 mL) or DCM:MeOH 3:1 (3 × 40 mL) for the compounds that had a higher solubility in water The combined organic layers were washed with brine (2 × 60 mL), dried over Na_2SO_4 anhydrous, filtered and evaporated under reduced pressure. The desired product was purified from the crude mixture via SFC or prep-HPLC (see compound characterization section for isolation details).

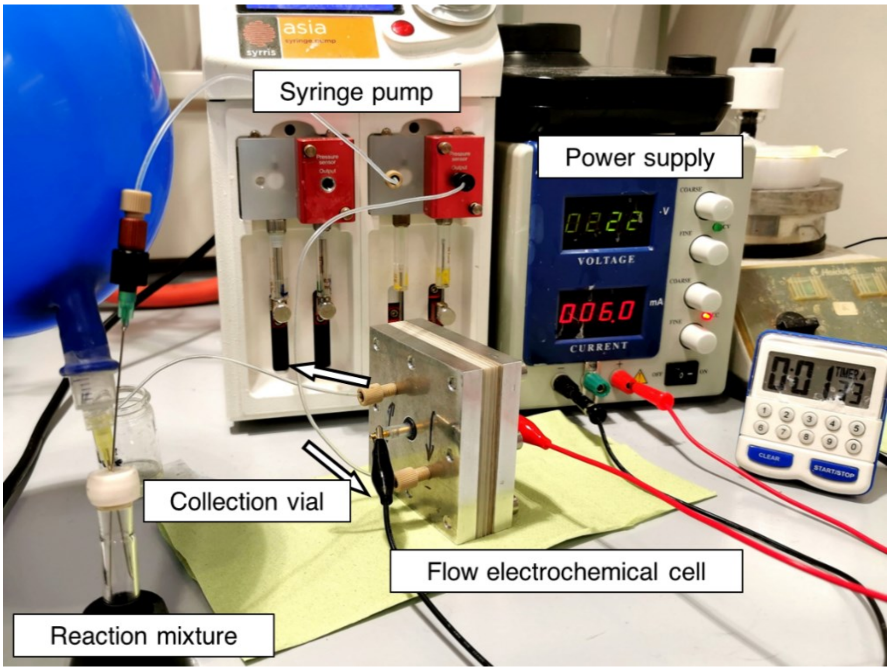

Figure S7. Picture of the setup used for the reactions in preparative scale. The reaction mixture is pumped from a volumetric flask under argon directly into the flow electrolysis cell while the outcome of the reaction is collected in a vial.

6. Library Synthesis with the Automated Platform

Results Summary

Table S1 Summary of the library (reported as HPLC area of desired product) obtained with the automated platform.

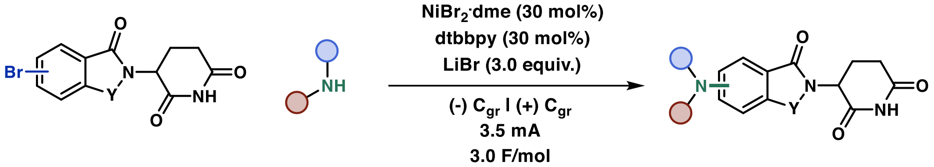

	1	2	3	4
A	72%	62%	27%	73%
B	73%	51%	0%	25%
C	77%	68%	43%	82%
D	75%	54%	40%	73%
E	79%	65%	47%	74%
F	56%	34%	26%	42%
G	32%	11%	8%	6%
H	37%	31%	18%	16%
I	3%	4%	0%	7%
J	44%	12%	33%	44%
K	19%	16%	18%	12%

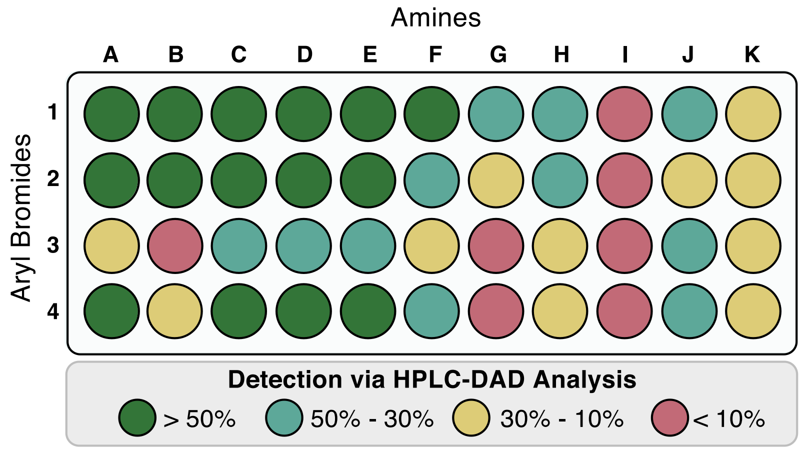

Figure S8. Library synthesis results in Tol's palette.

Library Synthesis Chromatograms

Preliminary information

All the chromatograms were recorded employing an HPLC-DAD instrument. The products were confirmed by LC-MS.

HPLC-DAD Chromatogram

HPLC analysis was performed using a Shimadzu LC20 system with a reversed phase C18 column (150 mm × 4.6 mm; 5 µm particle size) at 37 °C with a total flow rate of 1.5 mL/min. The following gradient program was applied: starting from 3% solvent B, the amount of solvent B was increased to 5% over 3 min, followed by an increase to 30% B over 4 min, then an increase to 100% B over 3 min and a hold time of 2 min. Solvent B was reduced back to 3% and held for 3 min to equilibrate. The mobile phases A (water/acetonitrile 9+1 v/v + 0.1% TFA) and B (acetonitrile + 0.1% TFA) were prepared from HPLC grade reagents. Analysis was performed at 215 nm unless stated otherwise.

LC-MS Chromatogram

LC-MS analysis were performed on a Shimadzu HPLC system comprised of a degassing unit (DGU-20A), solvent delivering unit (LC-20AD), an autosampler (SIL-20A), and a thermostated column oven (CTO-20A). The separation was carried out on a Macherey-Nagel Nucleodur C18 HTec column (150 mm × 4.6 mm, particle size 5 µm) at 37 °C using mobile phase A (H_2O/MeCN (9+1 v/v) + 0.1% TFA) and mobile phase B (MeCN + 0.1% TFA) at a flow rate of 0.6 $mL\cdot min^{-1}$. The following gradient was applied: hold 5% of B for 2 minutes, then linear increase from 5% B to 20% B in 6 min, followed by a linear increase from 20% B to 100% B in 8 min, then hold 100% B for 6 min, followed by column equilibration time at 5% B for 5 min. The detection of compounds was accomplished by diode array detector (SPD-M20A) prior electrospray ionization (ESI) using a Shimadzu LCMS-QP2020 instrument. The ESI-MS was operating either in positive or negative mode within a scan range of 150 - 600 m/z.
The interface voltage was either 4.5 kV (positive mode) or –4.5 kV (negative mode) and the detector voltage typically 0.7 kV. The interface temperature was 350 °C, the DL temperature 250 °C and the heat block temperature was 250 °C. Nitrogen was used as carrier gas and the dry gas flow rate was 18 $L\cdot min^{-1}$ and the nebulizer gas flow rate was 1.5 $L\cdot min^{-1}$.

Reaction Background

The following peaks were not integrated in the reactions chromatograms since they belong to the catalytic system (stock solution: $NiBr_2$·dme + dtbbpy).

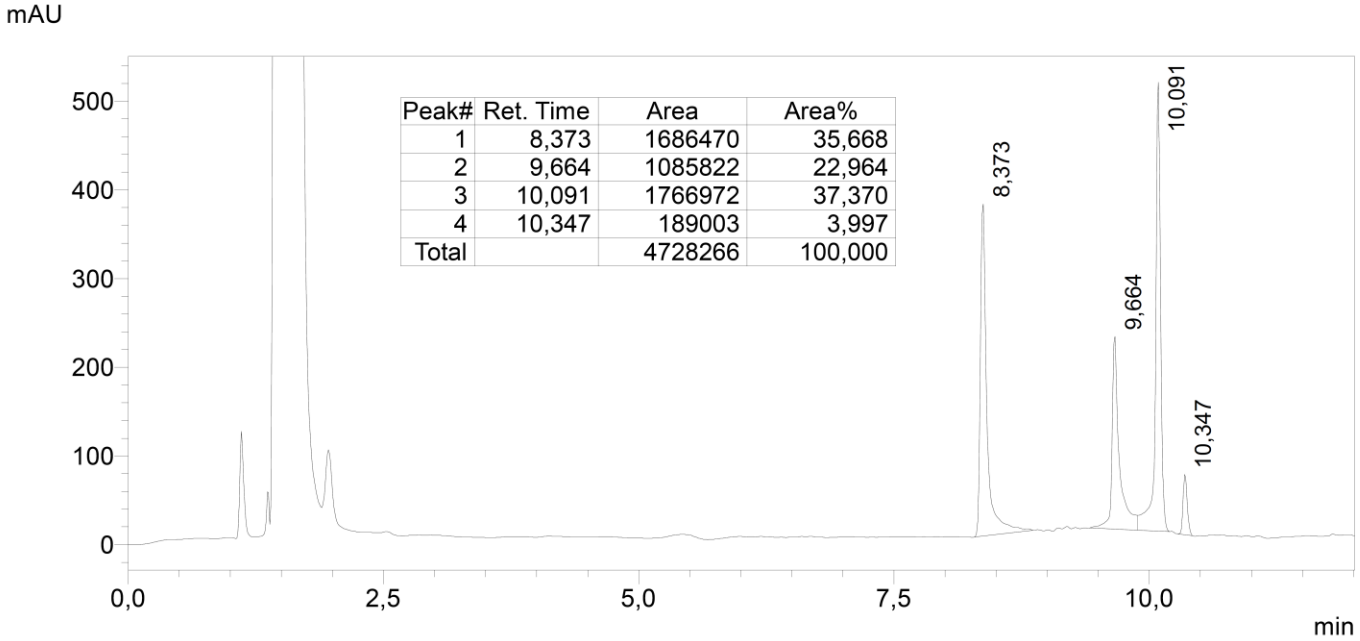

Peak#	Ret. Time	Area	Area%
1	8,373	1686470	35,668
2	9,664	1085822	22,964
3	10,091	1766972	37,370
4	10,347	189003	3,997
Total		4728266	100,000

Compound 1A

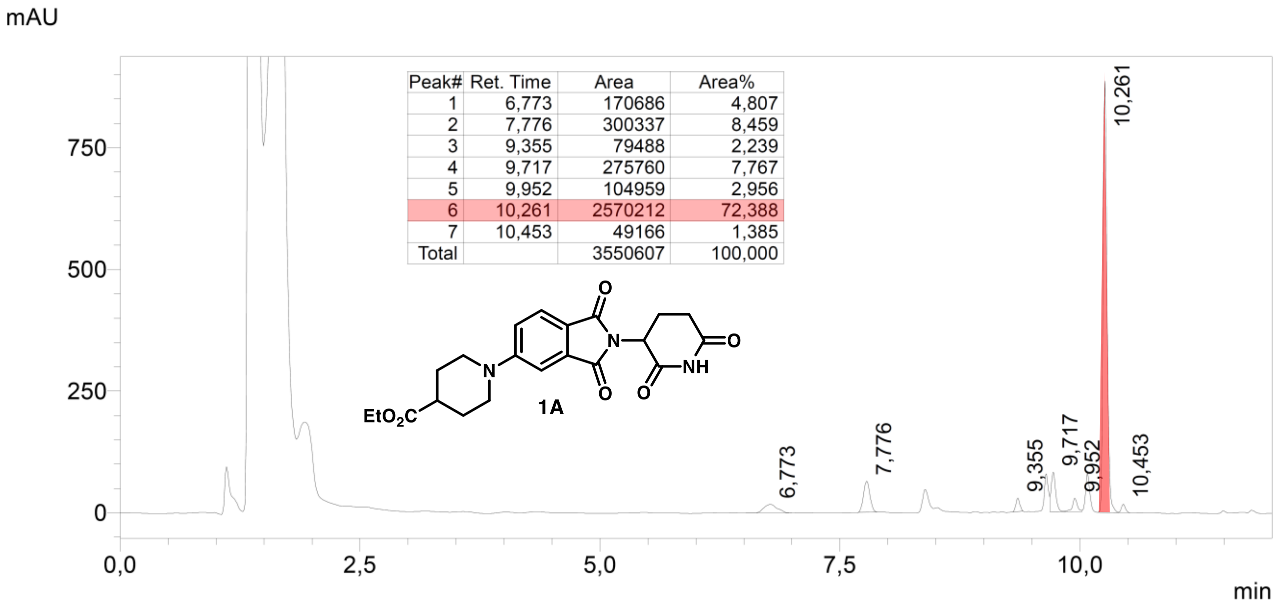

Peak#	Ret. Time	Area	Area%
1	6,773	170686	4,807
2	7,776	300337	8,459
3	9,355	79488	2,239
4	9,717	275760	7,767
5	9,952	104959	2,956
6	10,261	2570212	72,388
7	10,453	49166	1,385
Total		3550607	100,000

LC-MS: $[M + H]^+$ Calcd for $C_{21}H_{24}N_3O_6$ 414; Found 414.

Compound 1B

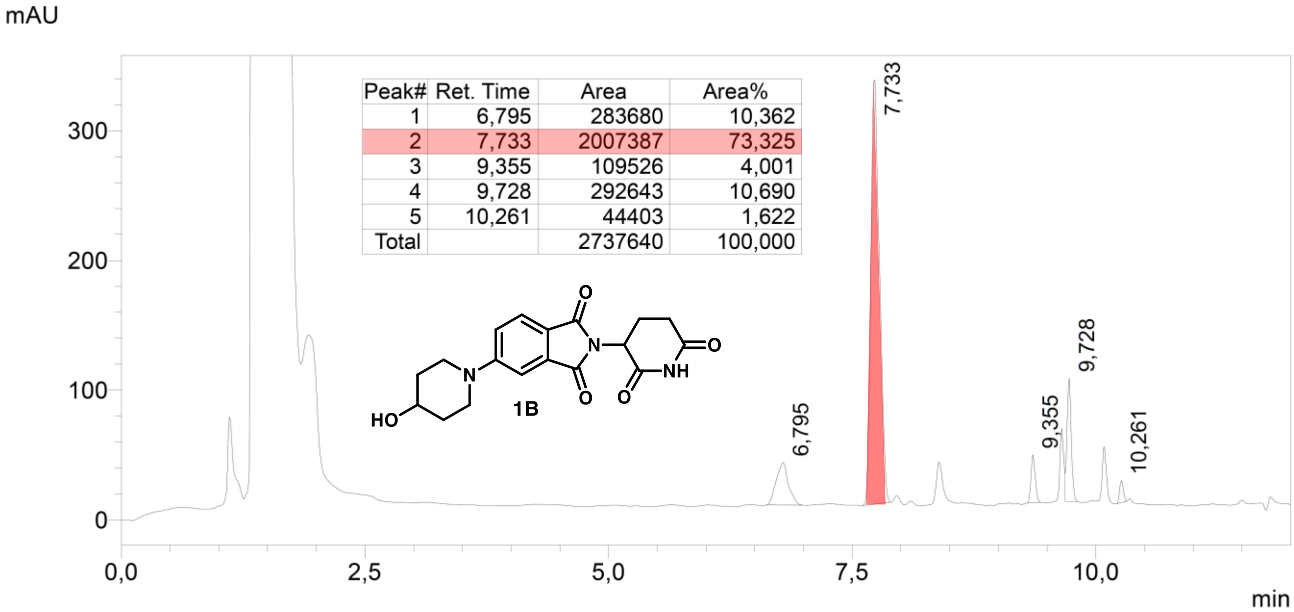

Peak#	Ret. Time	Area	Area%
1	6,795	283680	10,362
2	7,733	2007387	73,325
3	9,355	109526	4,001
4	9,728	292643	10,690
5	10,261	44403	1,622
Total		2737640	100,000

LC-MS: $[M + H]^+$ Calcd for $C_{18}H_{20}N_3O_5$ 358; Found 358.

Compound 1C

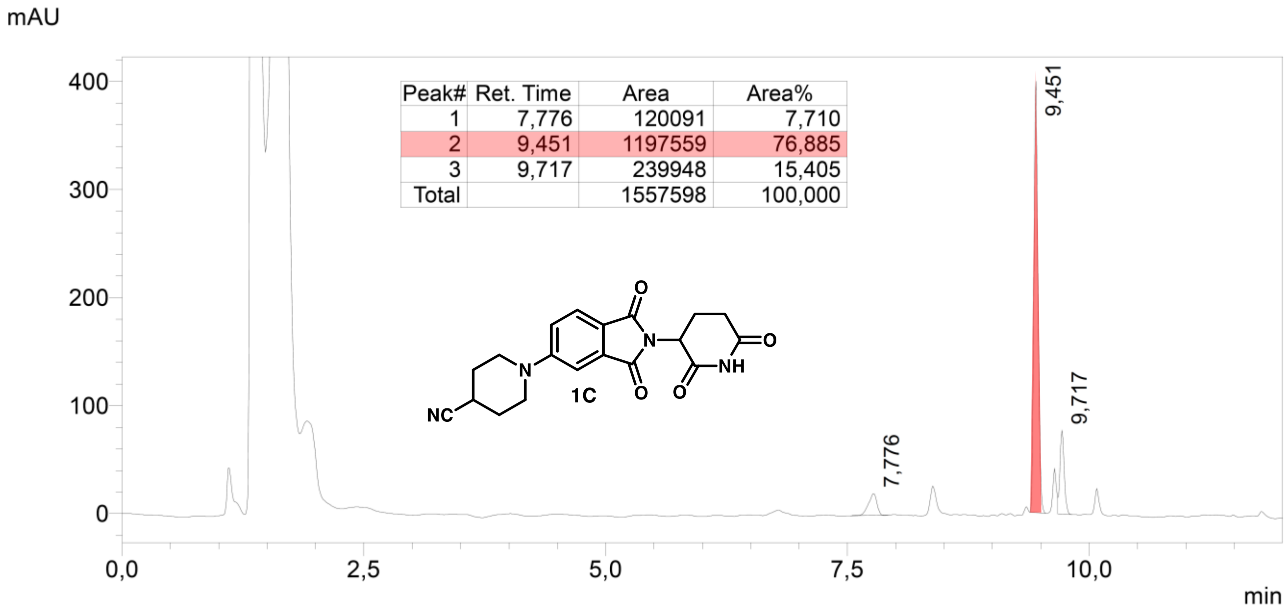

Peak#	Ret. Time	Area	Area%
1	7,776	120091	7,710
2	9,451	1197559	76,885
3	9,717	239948	15,405
Total		1557598	100,000

LC-MS: $[M + H]^+$ Calcd for $C_{19}H_{19}N_4O_4$ 367; Found 367.

Compound 1D

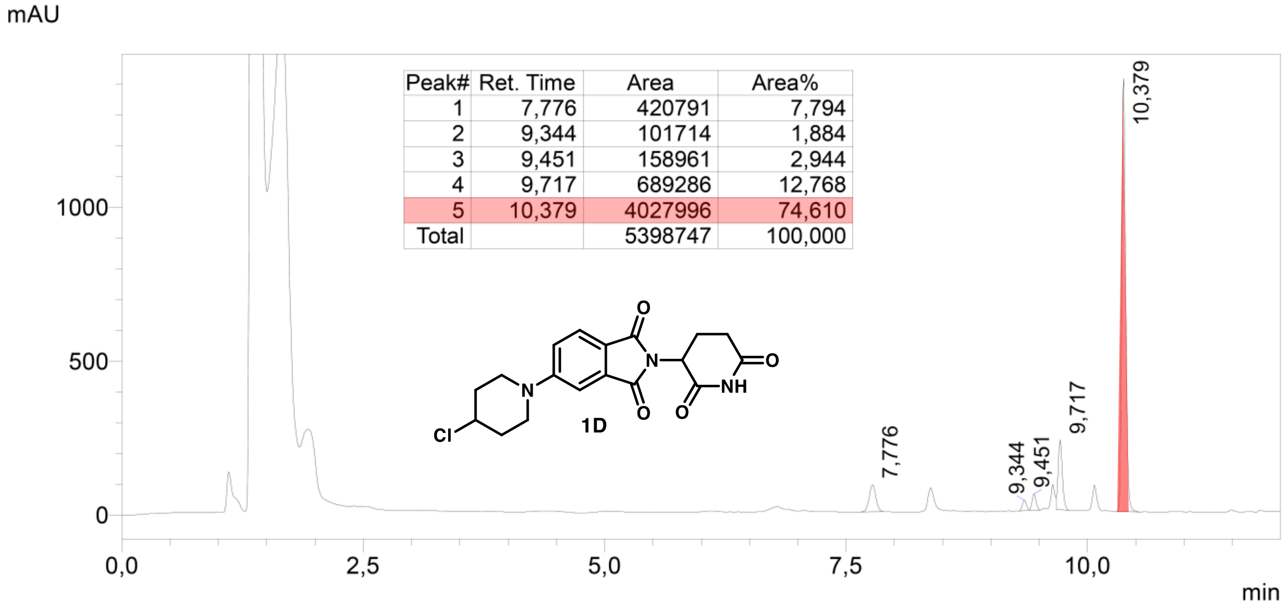

Peak#	Ret. Time	Area	Area%
1	7,776	420791	7,794
2	9,344	101714	1,884
3	9,451	158961	2,944
4	9,717	689286	12,768
5	10,379	4027996	74,610
Total		5398747	100,000

LC-MS: $[M + H]^+$ Calcd for $C_{18}H_{19}ClN_3O_4$ 376; Found 376.

Compound 1E

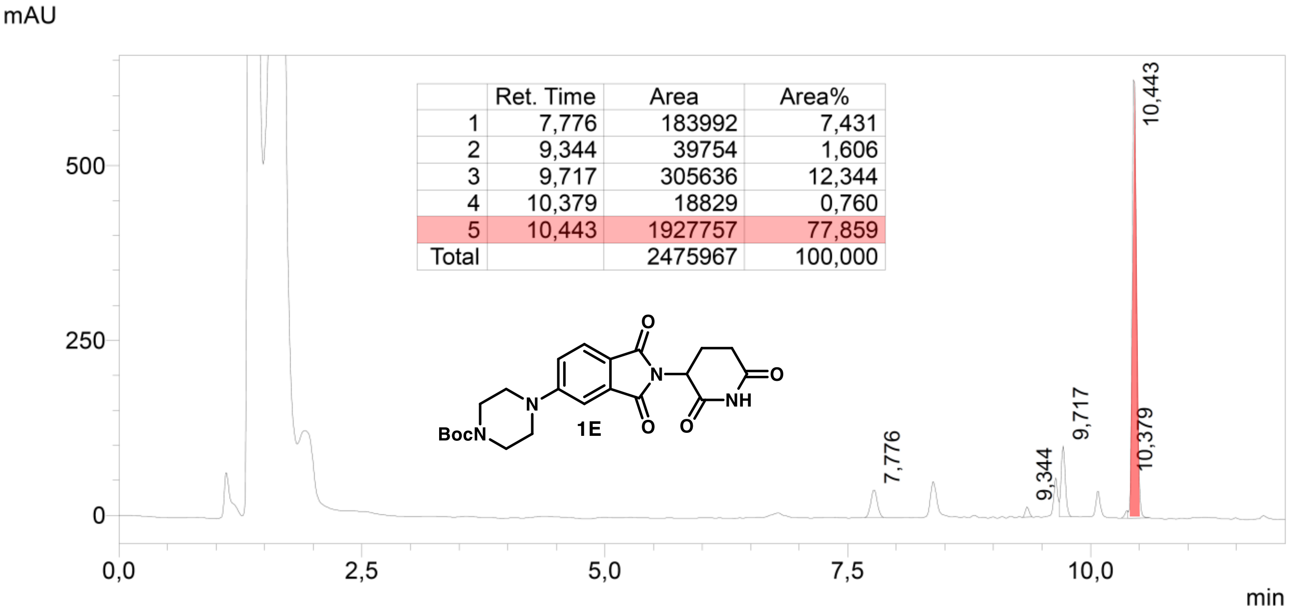

	Ret. Time	Area	Area%
1	7,776	183992	7,431
2	9,344	39754	1,606
3	9,717	305636	12,344
4	10,379	18829	0,760
5	10,443	1927757	77,859
Total		2475967	100,000

LC-MS: $[M - H]^-$ Calcd for $C_{22}H_{25}N_4O_6$ 441; Found 441.

Compound 1F

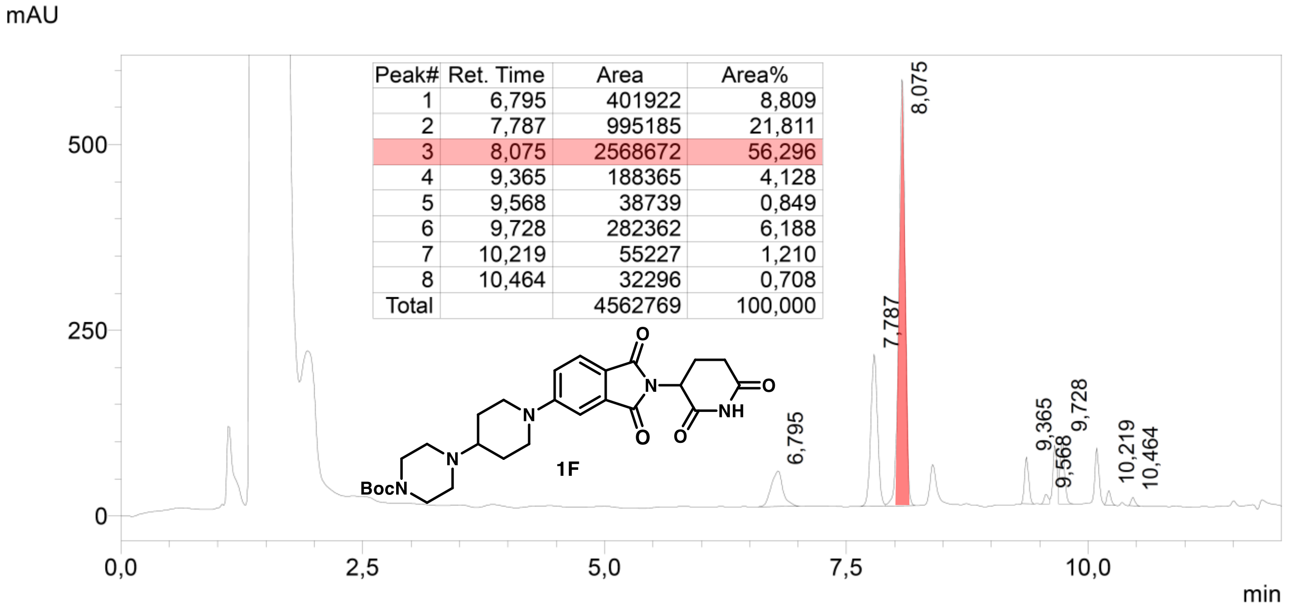

Peak#	Ret. Time	Area	Area%
1	6,795	401922	8,809
2	7,787	995185	21,811
3	8,075	2568672	56,296
4	9,365	188365	4,128
5	9,568	38739	0,849
6	9,728	282362	6,188
7	10,219	55227	1,210
8	10,464	32296	0,708
Total		4562769	100,000

LC-MS: $[M + H]^+$ Calcd for $C_{27}H_{36}N_5O_6$ 526; Found 526.

Compound 1G

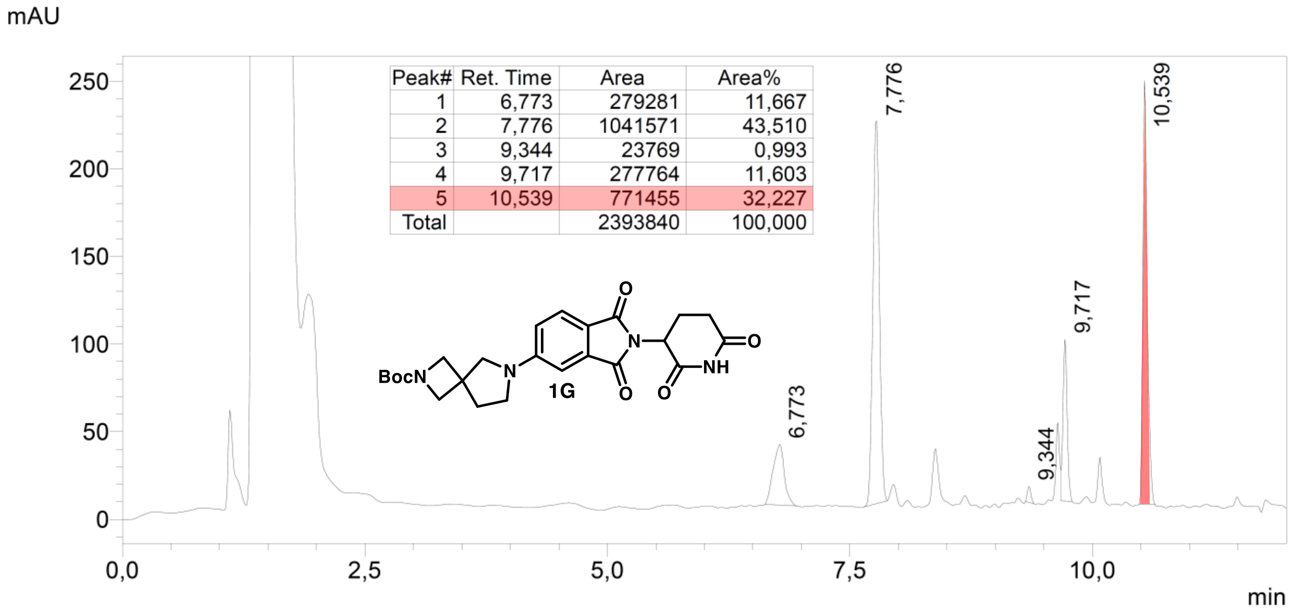

Peak#	Ret. Time	Area	Area%
1	6,773	279281	11,667
2	7,776	1041571	43,510
3	9,344	23769	0,993
4	9,717	277764	11,603
5	10,539	771455	32,227
Total		2393840	100,000

LC-MS: $[M - H]^-$ Calcd for $C_{24}H_{27}N_4O_6$ 467; Found 467.

Compound 1H

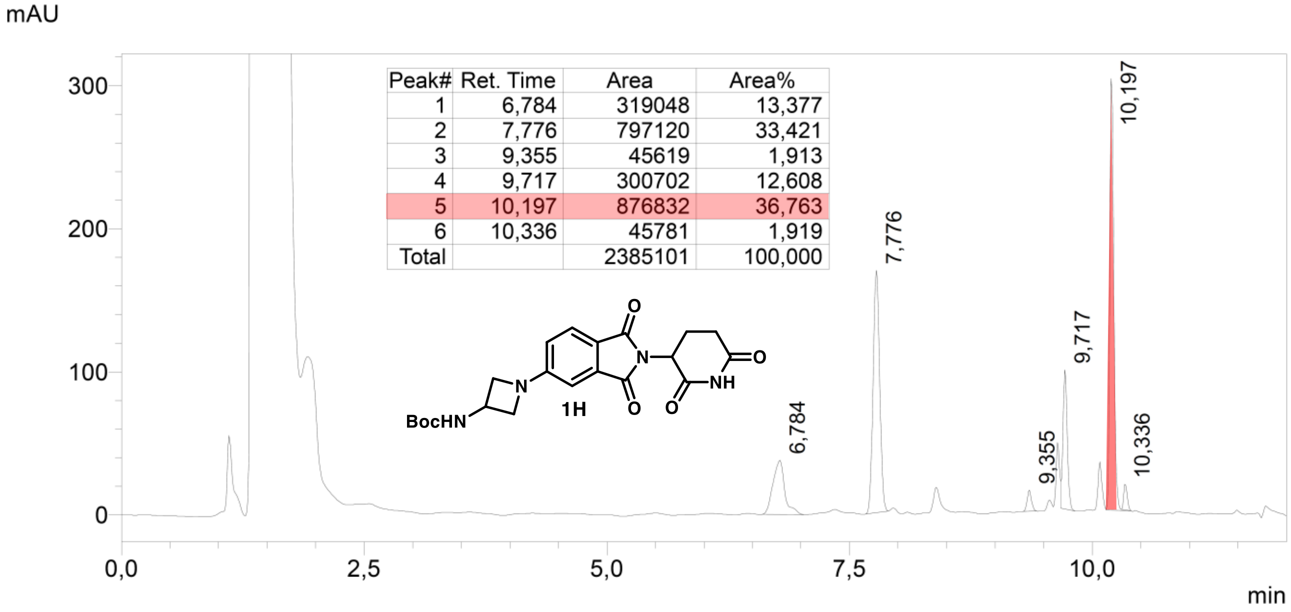

Peak#	Ret. Time	Area	Area%
1	6,784	319048	13,377
2	7,776	797120	33,421
3	9,355	45619	1,913
4	9,717	300702	12,608
5	10,197	876832	36,763
6	10,336	45781	1,919
Total		2385101	100,000

LC-MS: $[M + H]^+$ Calcd for $C_{21}H_{25}N_4O_6$ 429; Found 429.

Compound 1I

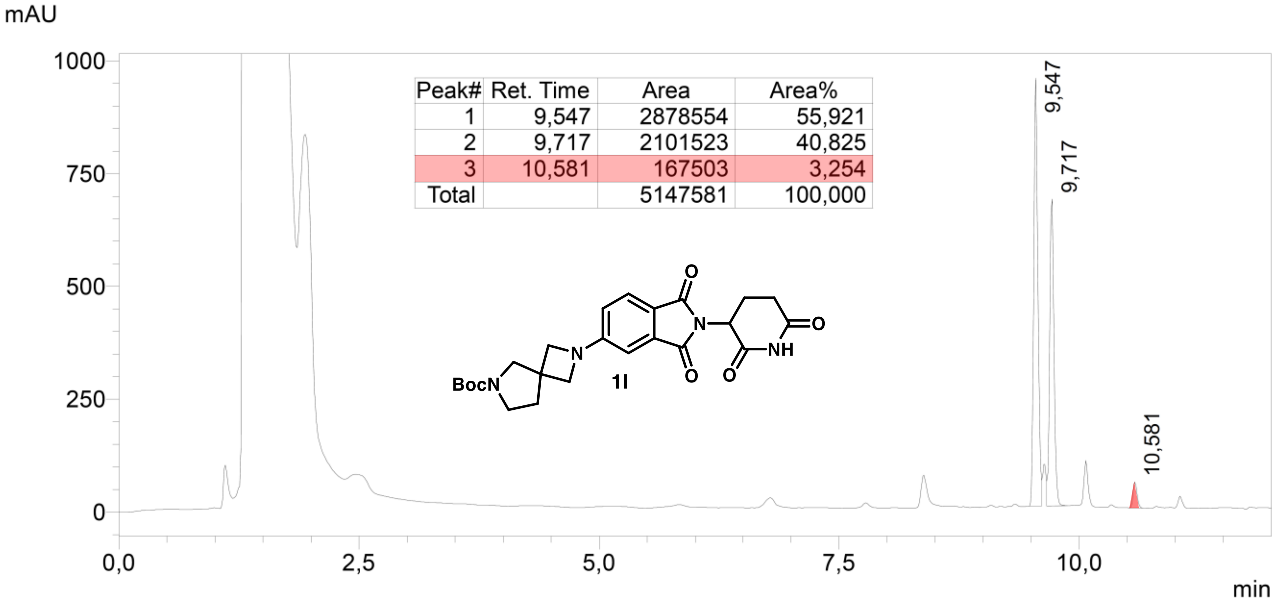

Peak#	Ret. Time	Area	Area%
1	9,547	2878554	55,921
2	9,717	2101523	40,825
3	10,581	167503	3,254
Total		5147581	100,000

LC-MS: $[M - H]^-$ Calcd for $C_{24}H_{27}N_4O_6$ 467; Found 467.

Compound 1J

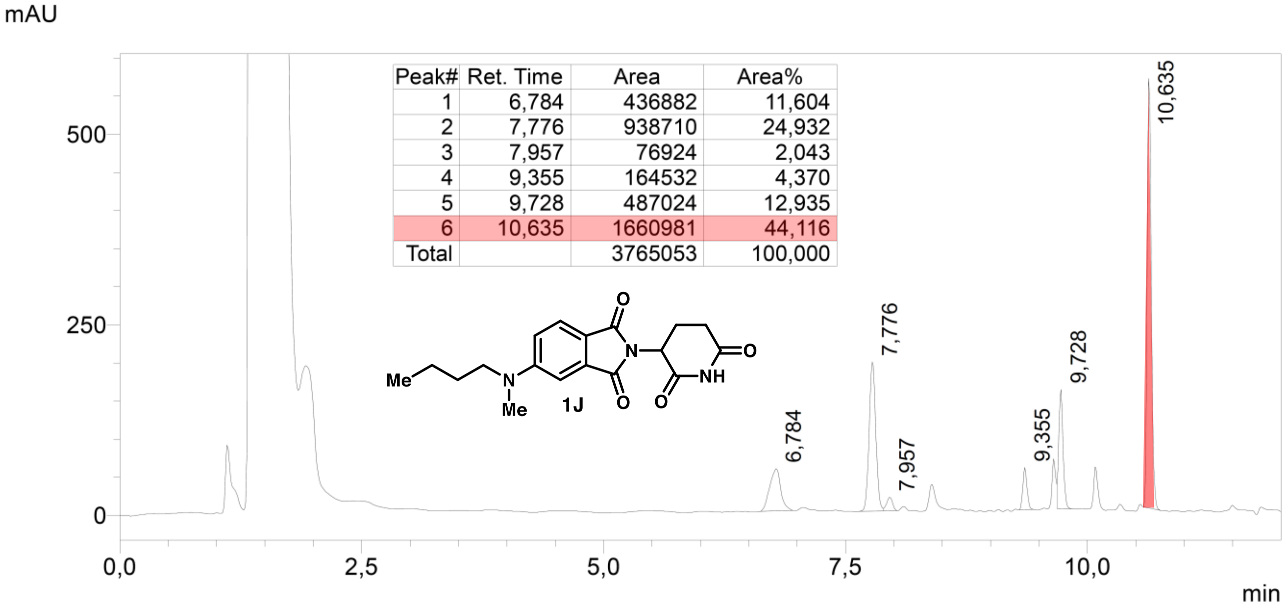

Peak#	Ret. Time	Area	Area%
1	6,784	436882	11,604
2	7,776	938710	24,932
3	7,957	76924	2,043
4	9,355	164532	4,370
5	9,728	487024	12,935
6	10,635	1660981	44,116
Total		3765053	100,000

LC-MS: $[M + H]^+$ Calcd for $C_{18}H_{22}N_3O_4$ 344; Found 344.

Compound 1K

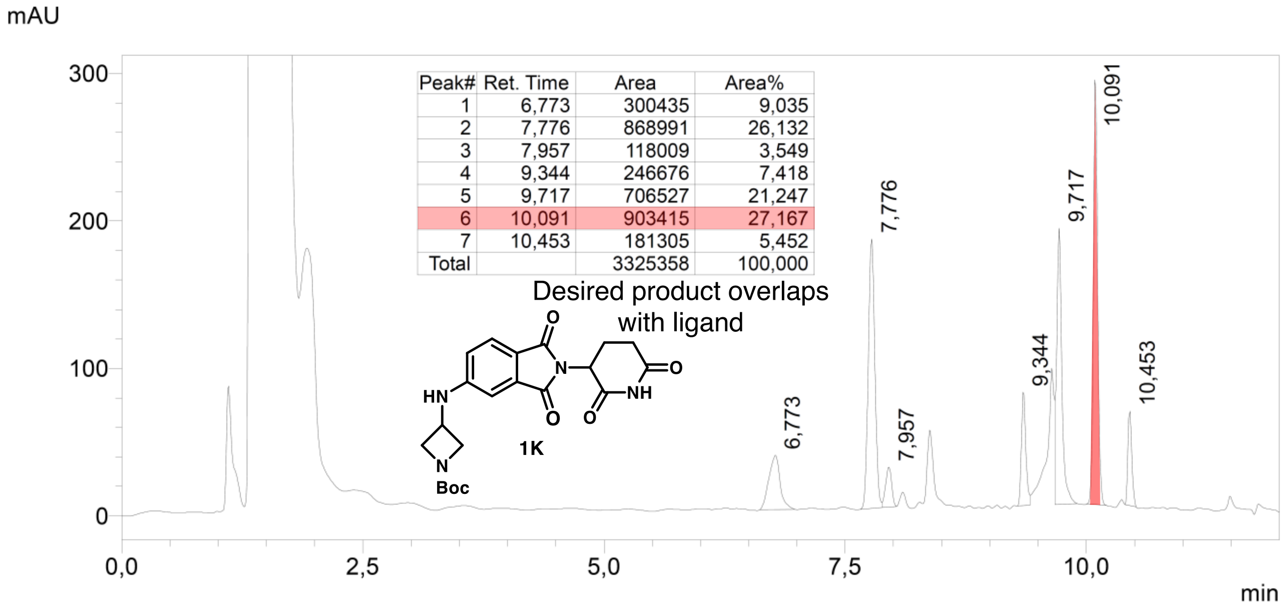

LC-MS: [M - H]$^-$ Calcd for $C_{21}H_{23}N_4O_6$ 427; Found 427.

Compound 2A

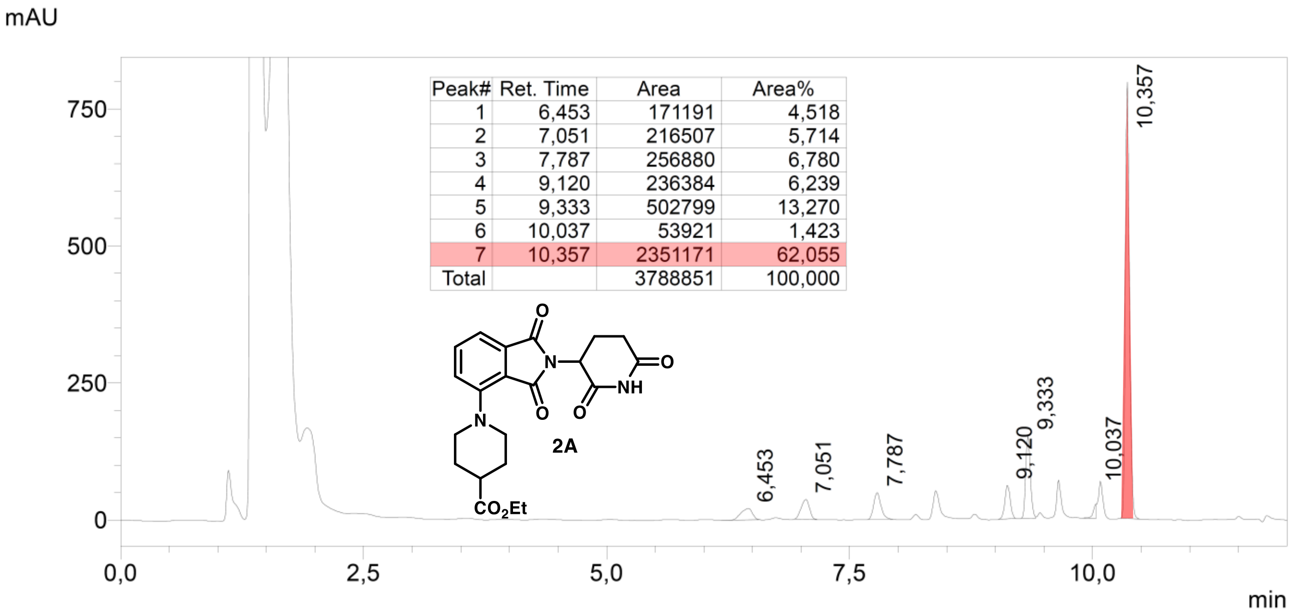

Peak#	Ret. Time	Area	Area%
1	6,453	171191	4,518
2	7,051	216507	5,714
3	7,787	256880	6,780
4	9,120	236384	6,239
5	9,333	502799	13,270
6	10,037	53921	1,423
7	10,357	2351171	62,055
Total		3788851	100,000

LC-MS: $[M + H]^+$ Calcd for $C_{21}H_{24}N_3O_6$ 414; Found 414.

Compound 2B

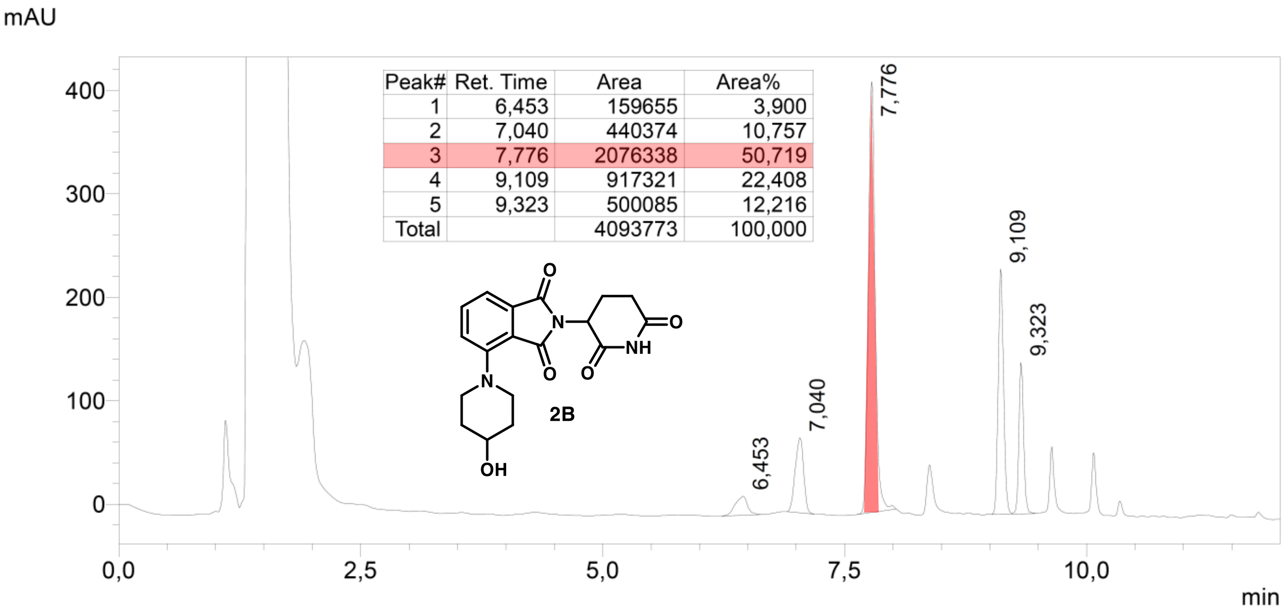

Peak#	Ret. Time	Area	Area%
1	6,453	159655	3,900
2	7,040	440374	10,757
3	7,776	2076338	50,719
4	9,109	917321	22,408
5	9,323	500085	12,216
Total		4093773	100,000

LC-MS: $[M + H]^+$ Calcd for $C_{18}H_{20}N_3O_5$ 358; Found 358.

Compound 2C

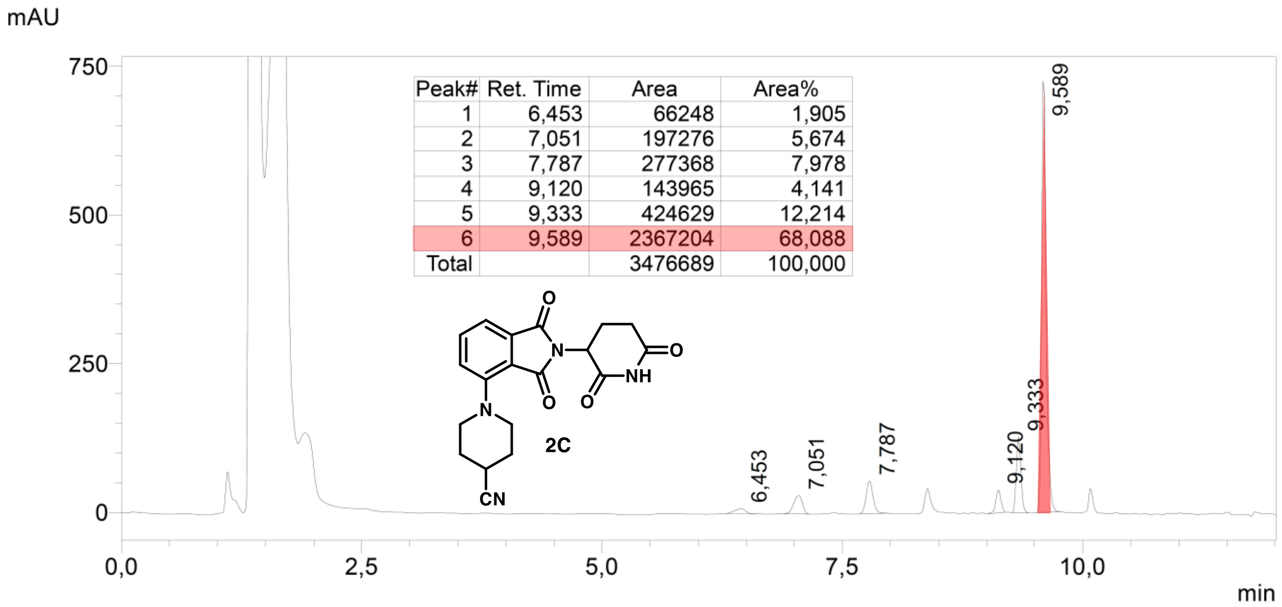

Peak#	Ret. Time	Area	Area%
1	6,453	66248	1,905
2	7,051	197276	5,674
3	7,787	277368	7,978
4	9,120	143965	4,141
5	9,333	424629	12,214
6	9,589	2367204	68,088
Total		3476689	100,000

LC-MS: $[M + H]^+$ Calcd for $C_{19}H_{19}N_4O_4$ 367; Found 367.

Compound 2D

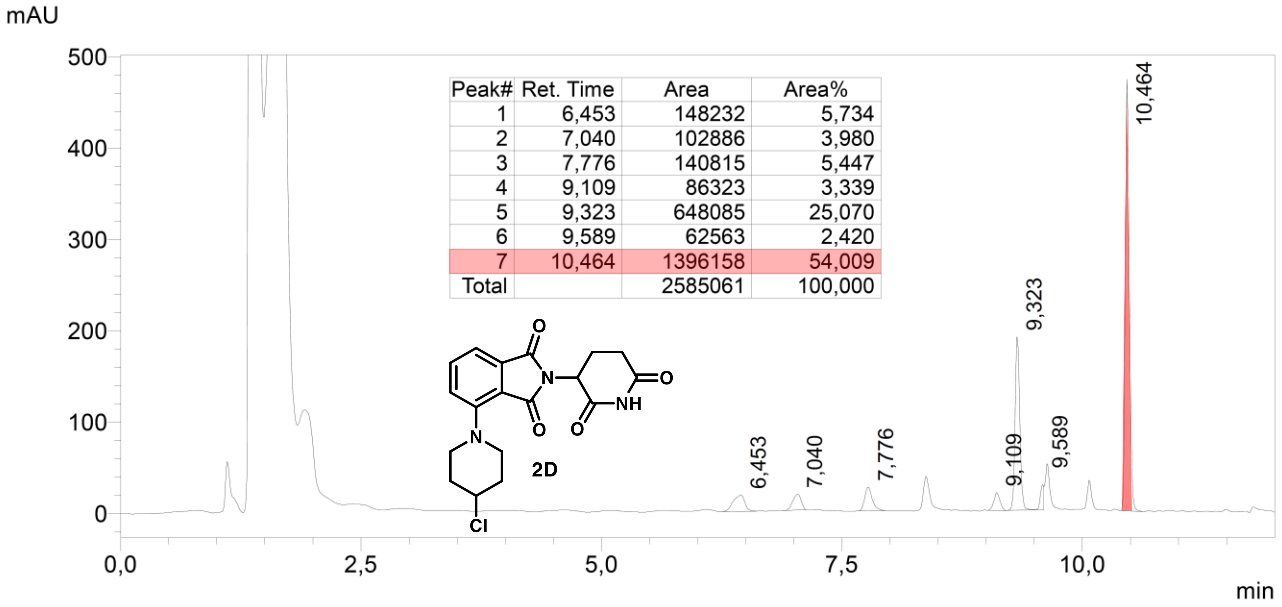

Peak#	Ret. Time	Area	Area%
1	6,453	148232	5,734
2	7,040	102886	3,980
3	7,776	140815	5,447
4	9,109	86323	3,339
5	9,323	648085	25,070
6	9,589	62563	2,420
7	10,464	1396158	54,009
Total		2585061	100,000

LC-MS: $[M + H]^+$ Calcd for $C_{18}H_{19}ClN_3O_4$ 376; Found 376.

Compound 2E

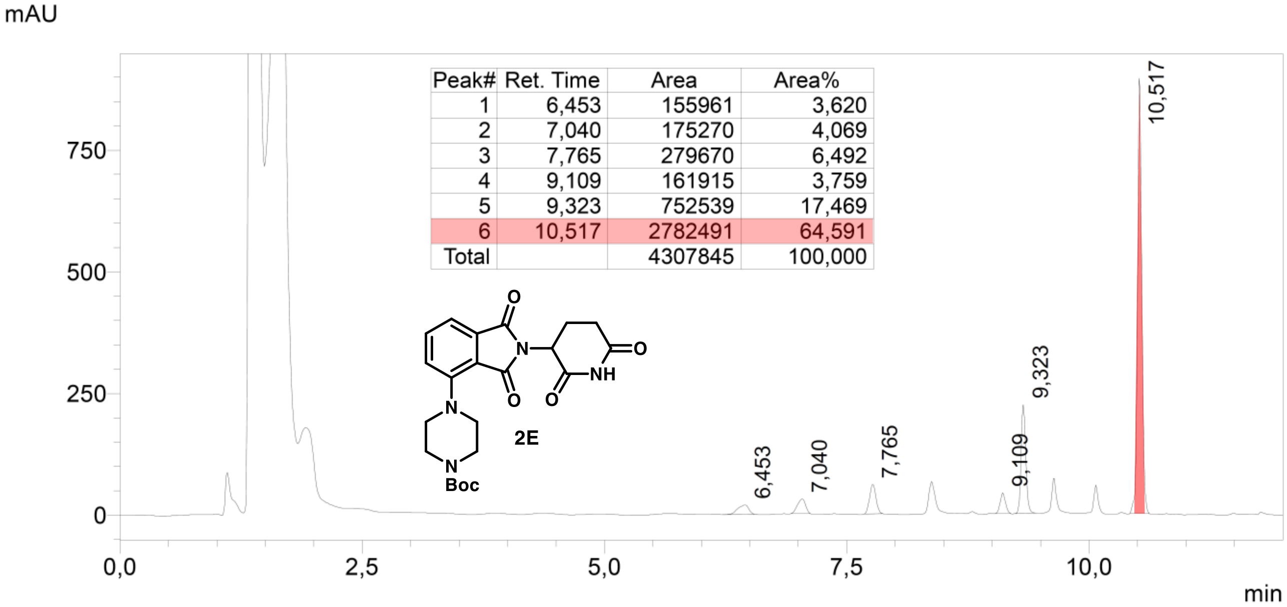

Peak#	Ret. Time	Area	Area%
1	6,453	155961	3,620
2	7,040	175270	4,069
3	7,765	279670	6,492
4	9,109	161915	3,759
5	9,323	752539	17,469
6	10,517	2782491	64,591
Total		4307845	100,000

LC-MS: $[M + H]^+$ Calcd for $C_{22}H_{27}N_4O_6$ 443; Found 443.

Compound 2F

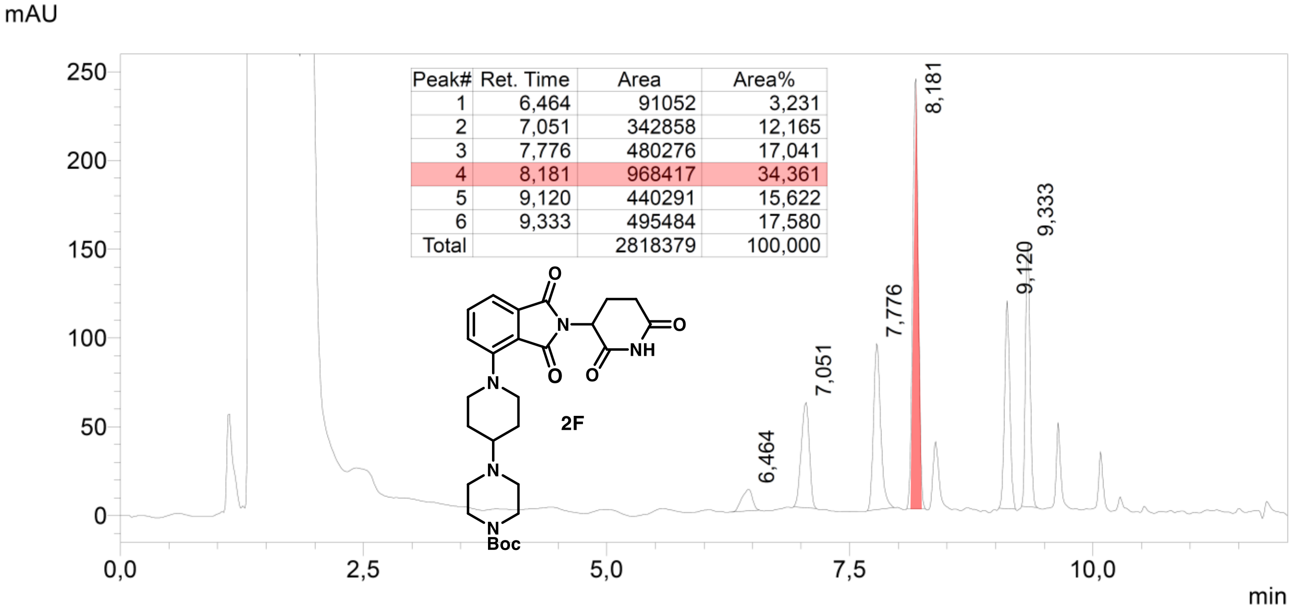

Peak#	Ret. Time	Area	Area%
1	6,464	91052	3,231
2	7,051	342858	12,165
3	7,776	480276	17,041
4	8,181	968417	34,361
5	9,120	440291	15,622
6	9,333	495484	17,580
Total		2818379	100,000

LC-MS: $[M + H]^+$ Calcd for $C_{27}H_{36}N_5O_6$ 526; Found 526.

Compound 2G

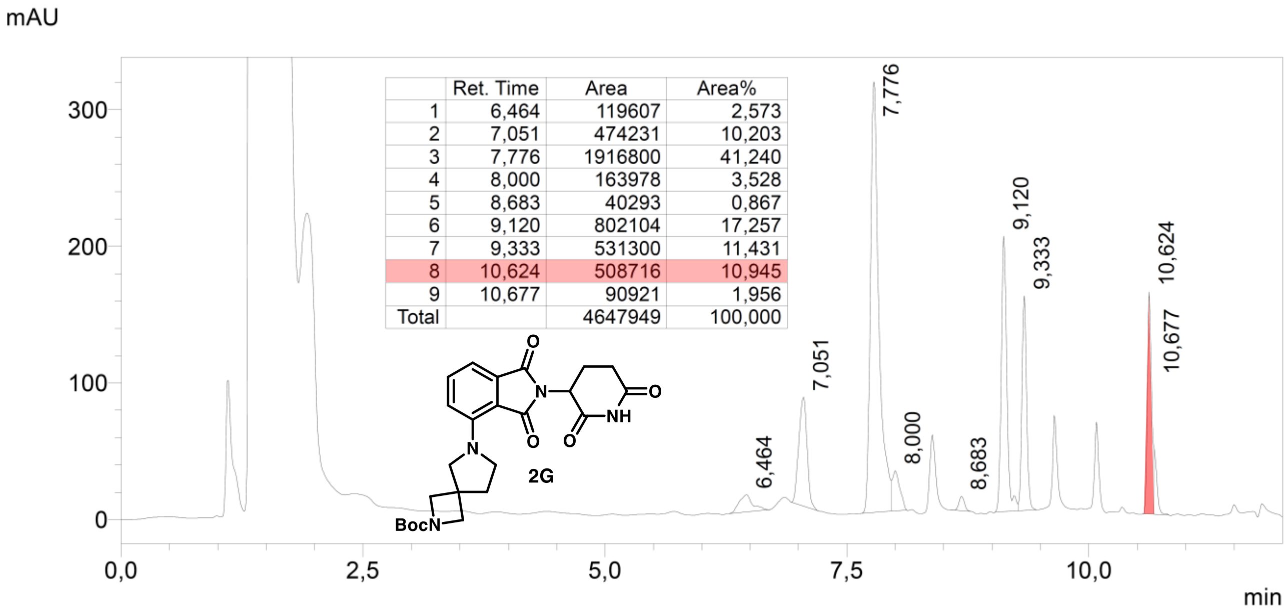

	Ret. Time	Area	Area%
1	6,464	119607	2,573
2	7,051	474231	10,203
3	7,776	1916800	41,240
4	8,000	163978	3,528
5	8,683	40293	0,867
6	9,120	802104	17,257
7	9,333	531300	11,431
8	10,624	508716	10,945
9	10,677	90921	1,956
Total		4647949	100,000

LC-MS: $[M + H]^+$ Calcd for $C_{24}H_{29}N_4O_6$ 469; Found 469.

Compound 2H

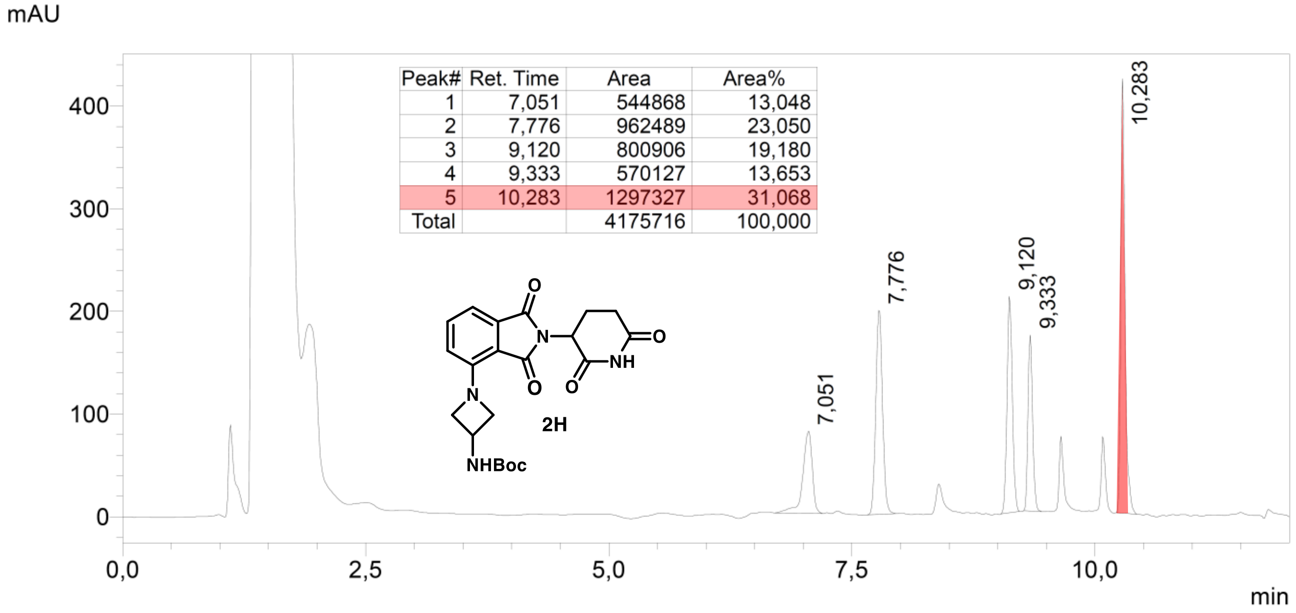

Peak#	Ret. Time	Area	Area%
1	7,051	544868	13,048
2	7,776	962489	23,050
3	9,120	800906	19,180
4	9,333	570127	13,653
5	10,283	1297327	31,068
Total		4175716	100,000

LC-MS: $[M + H]^+$ Calcd for $C_{21}H_{25}N_4O_6$ 429; Found 429.

Compound 2I

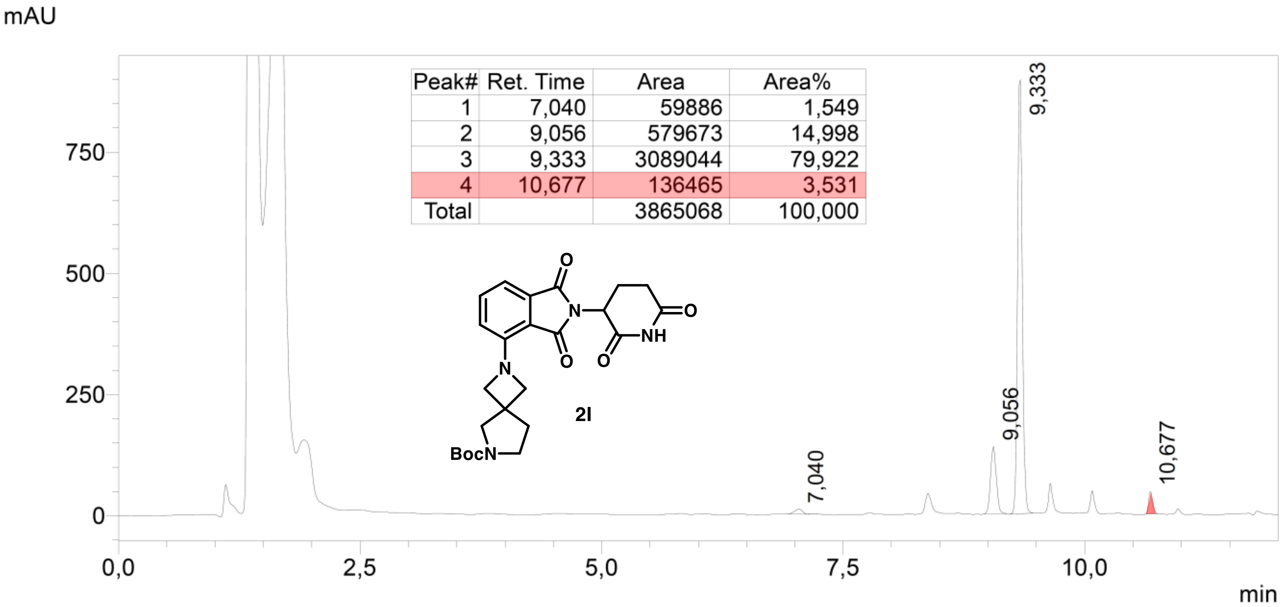

Peak#	Ret. Time	Area	Area%
1	7,040	59886	1,549
2	9,056	579673	14,998
3	9,333	3089044	79,922
4	10,677	136465	3,531
Total		3865068	100,000

LC-MS: $[M + H]^+$ Calcd for $C_{24}H_{29}N_4O_6$ 469; Found 469.

Compound 2J

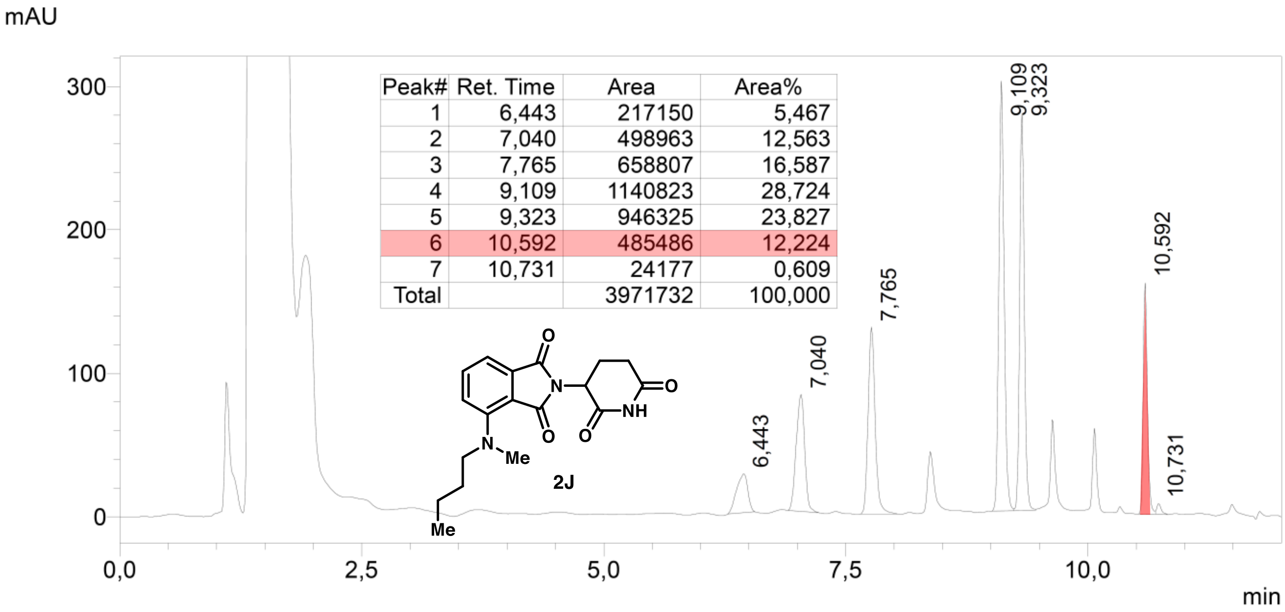

Peak#	Ret. Time	Area	Area%
1	6,443	217150	5,467
2	7,040	498963	12,563
3	7,765	658807	16,587
4	9,109	1140823	28,724
5	9,323	946325	23,827
6	10,592	485486	12,224
7	10,731	24177	0,609
Total		3971732	100,000

LC-MS: $[M + H]^+$ Calcd for $C_{18}H_{22}N_3O_4$ 343; Found 344.

Compound 2K

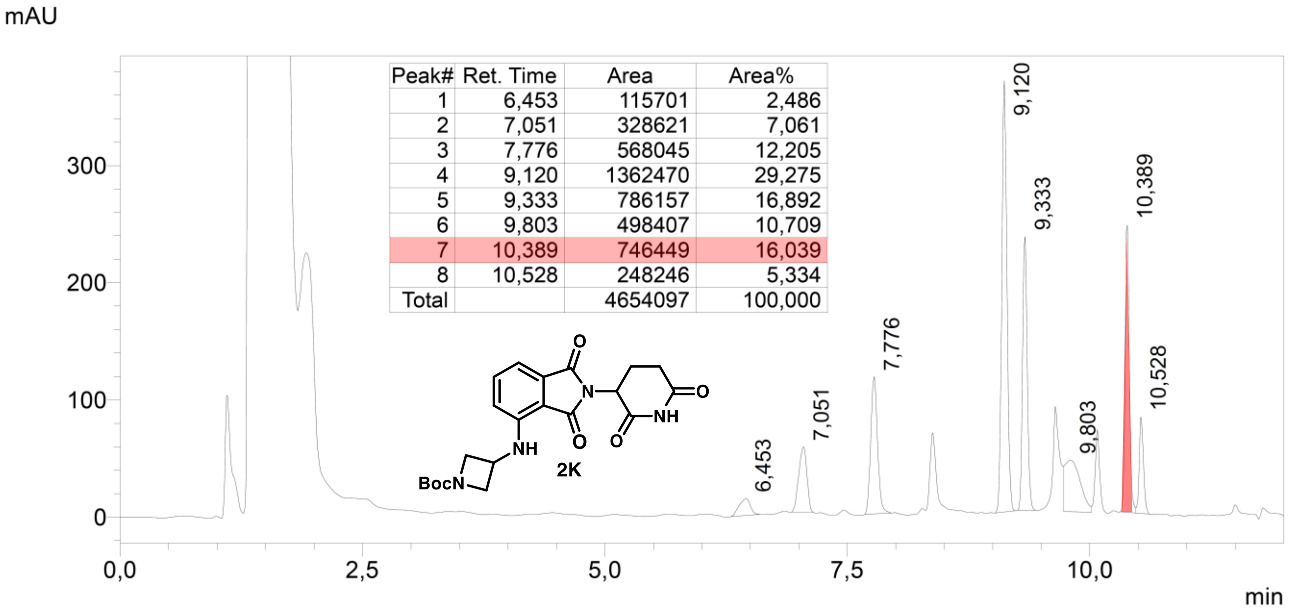

Peak#	Ret. Time	Area	Area%
1	6,453	115701	2,486
2	7,051	328621	7,061
3	7,776	568045	12,205
4	9,120	1362470	29,275
5	9,333	786157	16,892
6	9,803	498407	10,709
7	10,389	746449	16,039
8	10,528	248246	5,334
Total		4654097	100,000

LC-MS: $[M + H]^+$ Calcd for $C_{21}H_{25}N_4O_6$ 429; Found 429.

Compound 3A

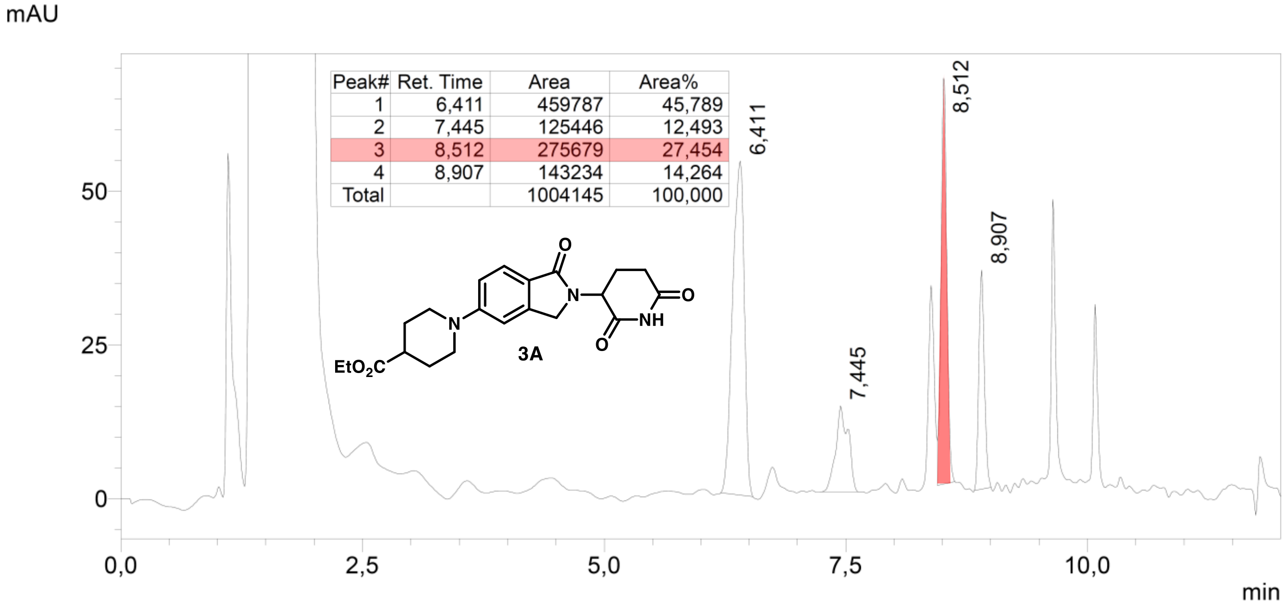

Peak#	Ret. Time	Area	Area%
1	6,411	459787	45,789
2	7,445	125446	12,493
3	8,512	275679	27,454
4	8,907	143234	14,264
Total		1004145	100,000

LC-MS: $[M + H]^+$ Calcd for $C_{21}H_{26}N_3O_5$ 400; Found 400.

Compound 3B

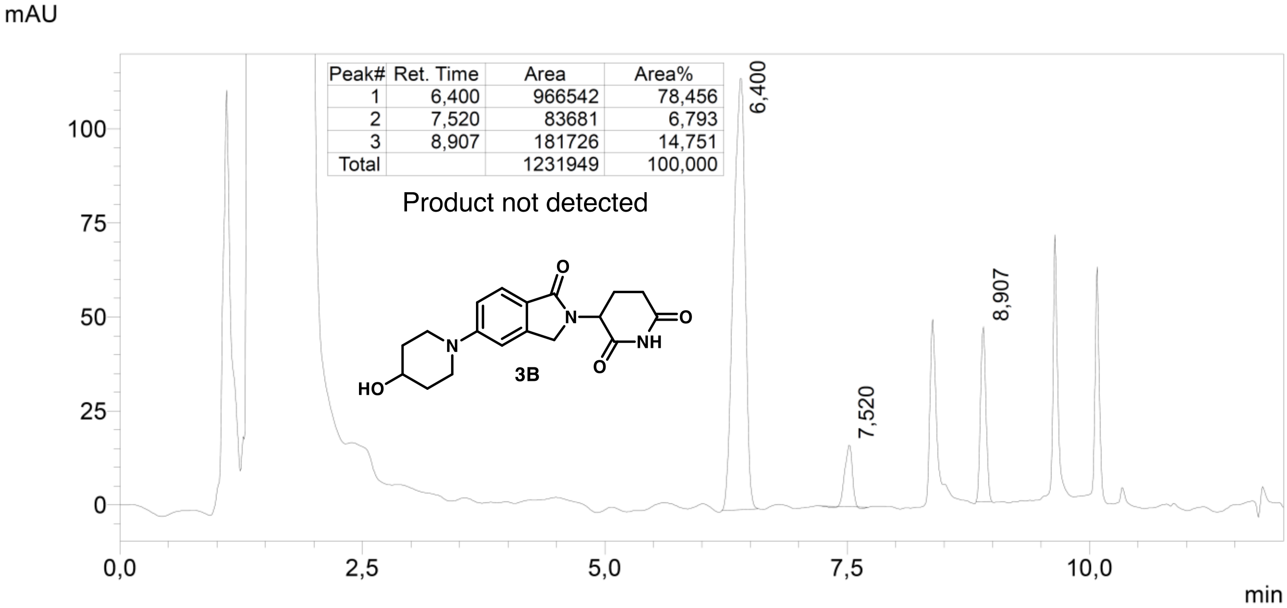

Peak#	Ret. Time	Area	Area%
1	6,400	966542	78,456
2	7,520	83681	6,793
3	8,907	181726	14,751
Total		1231949	100,000

Compound 3C

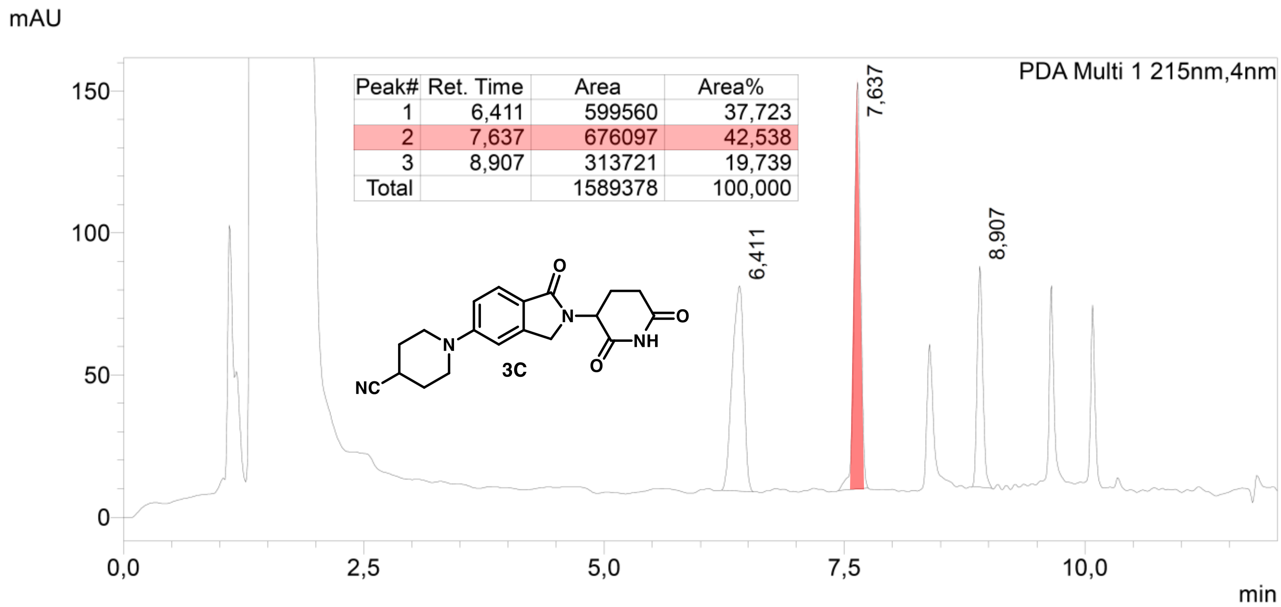

LC-MS: $[M + H]^+$ Calcd for $C_{19}H_{21}N_4O_3$ 353; Found 353.

Compound 3D

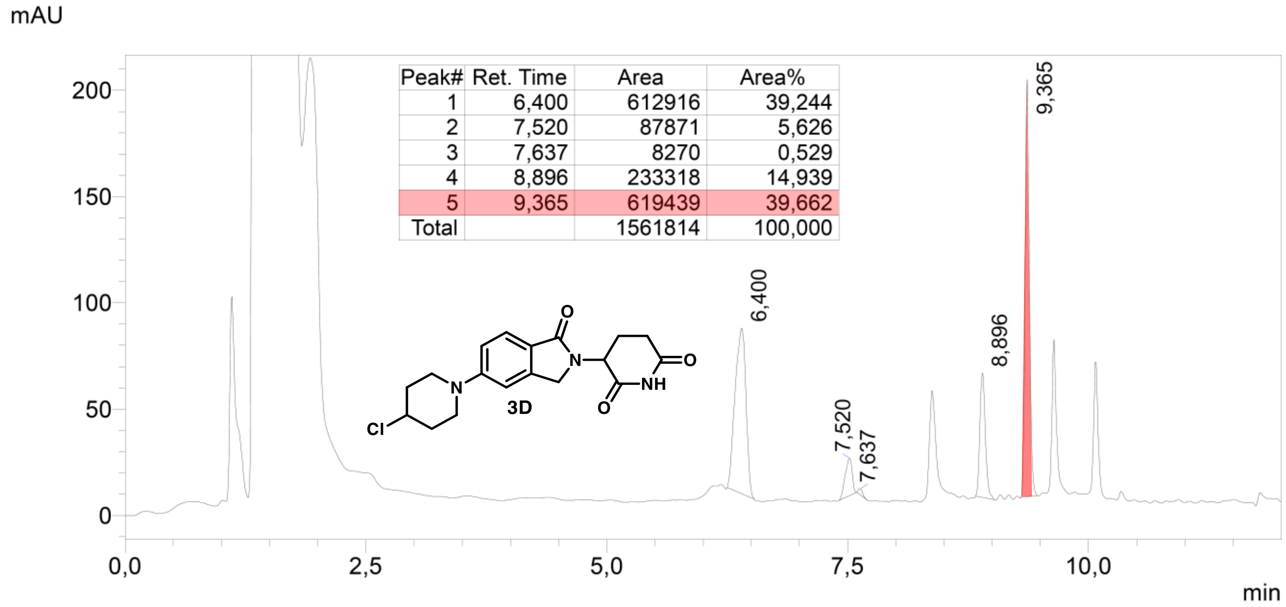

LC-MS: $[M + H]^+$ Calcd for $C_{18}H_{21}ClN_3O_3$ 362; Found 362.

Compound 3E

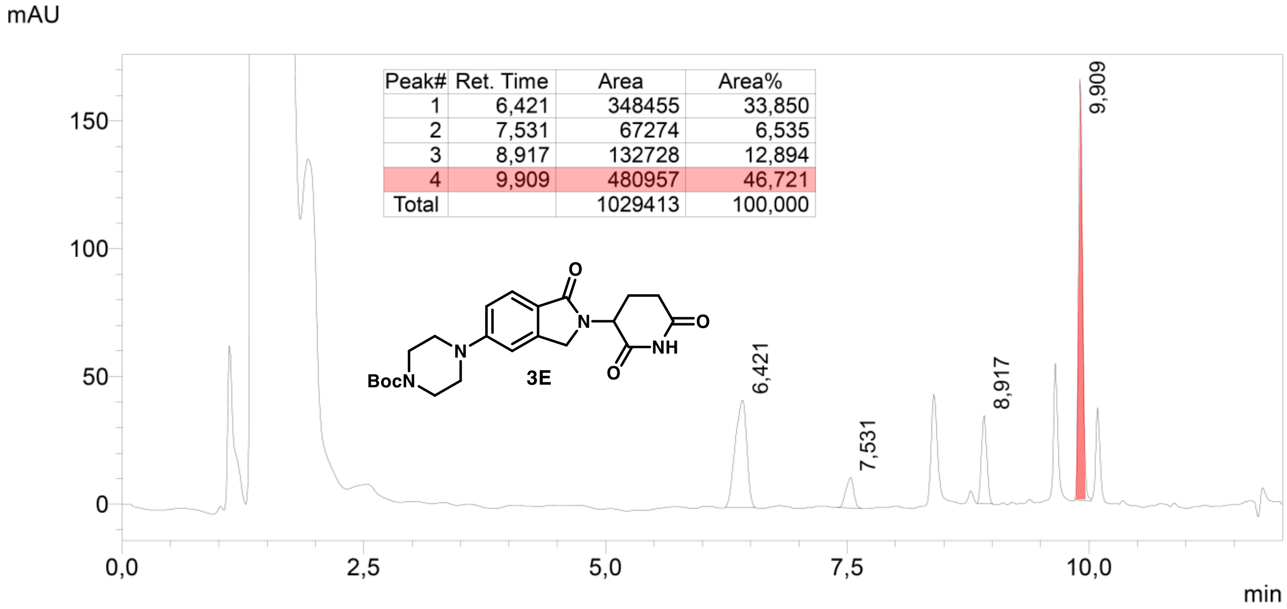

Peak#	Ret. Time	Area	Area%
1	6,421	348455	33,850
2	7,531	67274	6,535
3	8,917	132728	12,894
4	9,909	480957	46,721
Total		1029413	100,000

LC-MS: $[M + H]^+$ Calcd for $C_{22}H_{29}N_4O_5$ 429; Found 429.

Compound 3F

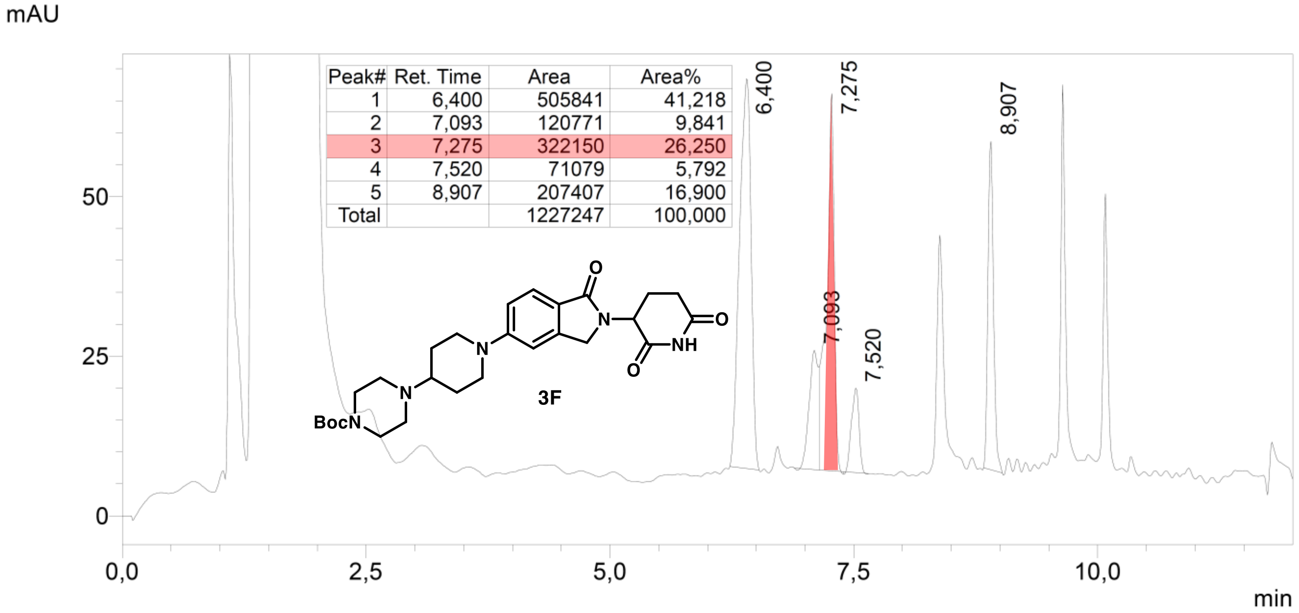

Peak#	Ret. Time	Area	Area%
1	6,400	505841	41,218
2	7,093	120771	9,841
3	7,275	322150	26,250
4	7,520	71079	5,792
5	8,907	207407	16,900
Total		1227247	100,000

LC-MS: $[M + H]^+$ Calcd for $C_{27}H_{38}N_5O_5$ 512; Found 512.

Compound 3G

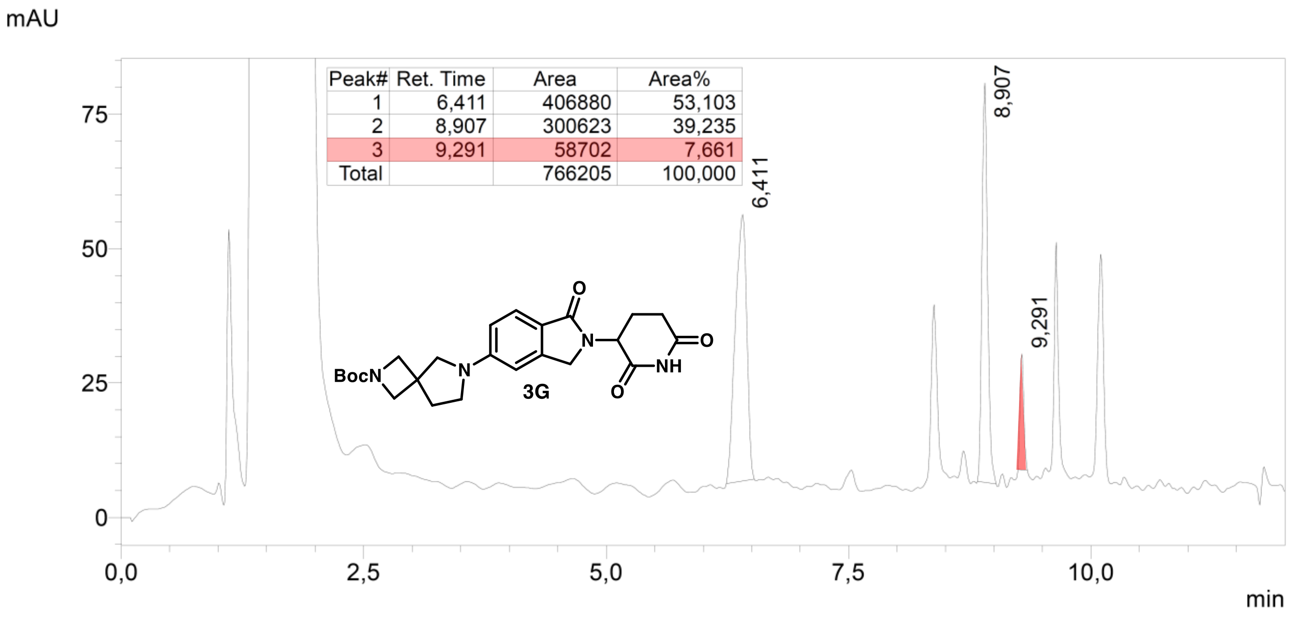

LC-MS: $[M + H]^+$ Calcd for $C_{24}H_{31}N_4O_5$ 455; Found 455.

Compound 3H

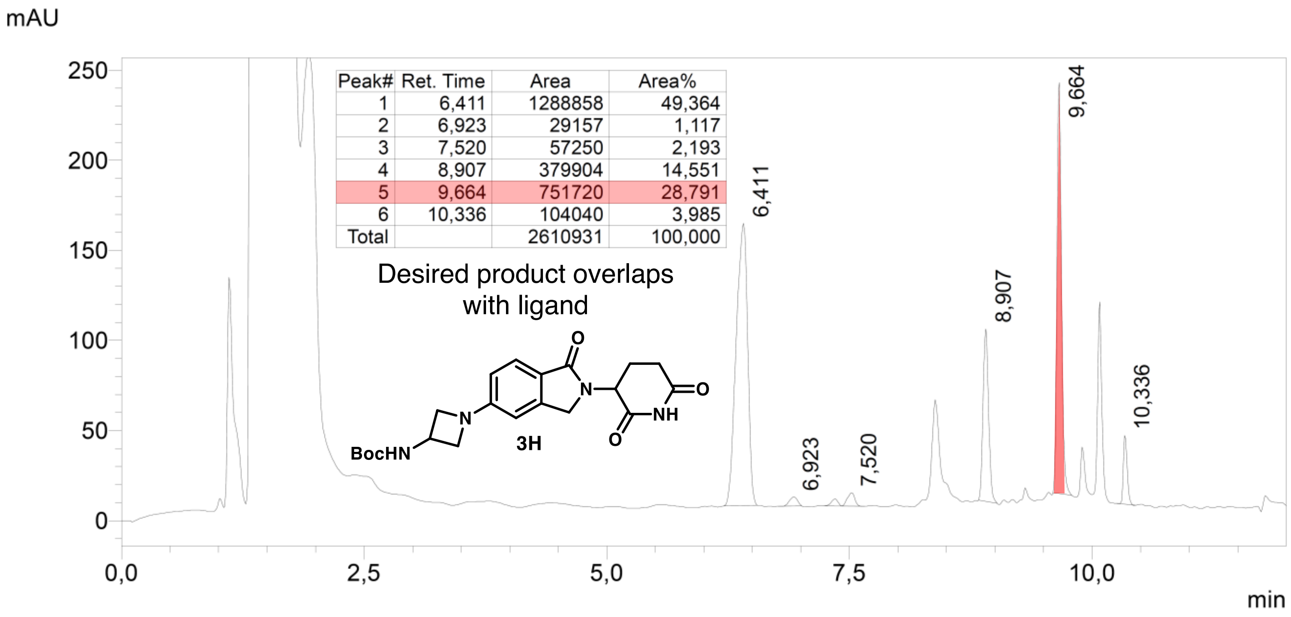

LC-MS: $[M + H]^+$ Calcd for $C_{21}H_{27}N_4O_5$ 415; Found 415.

Compound 3I

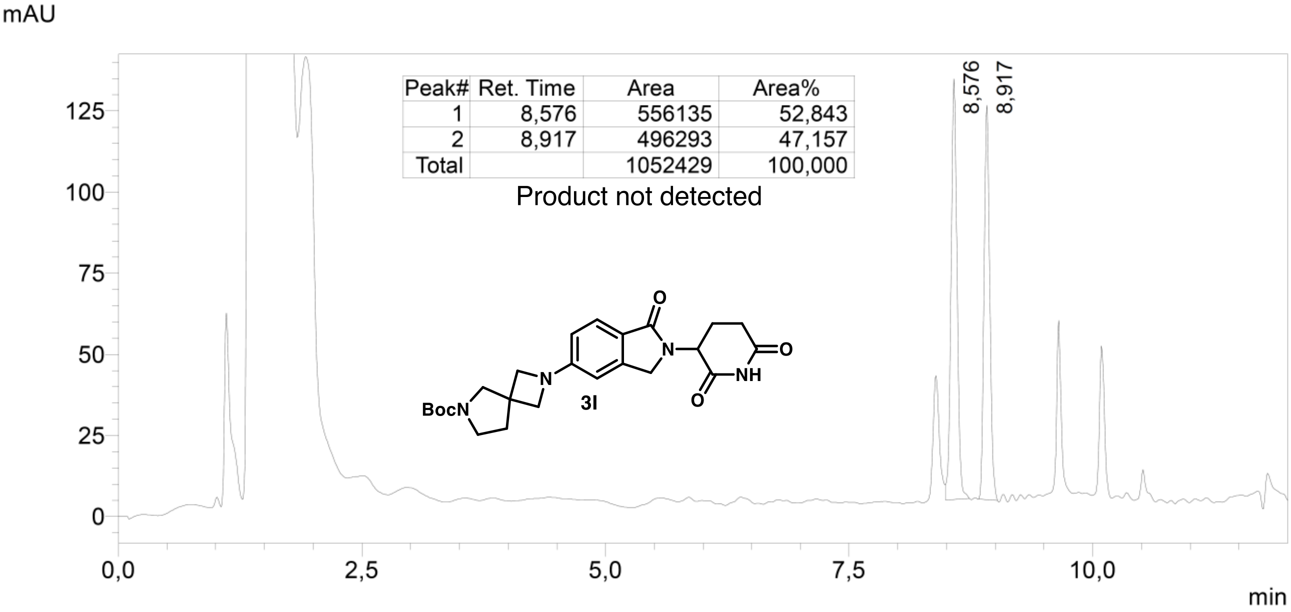

Compound 3J

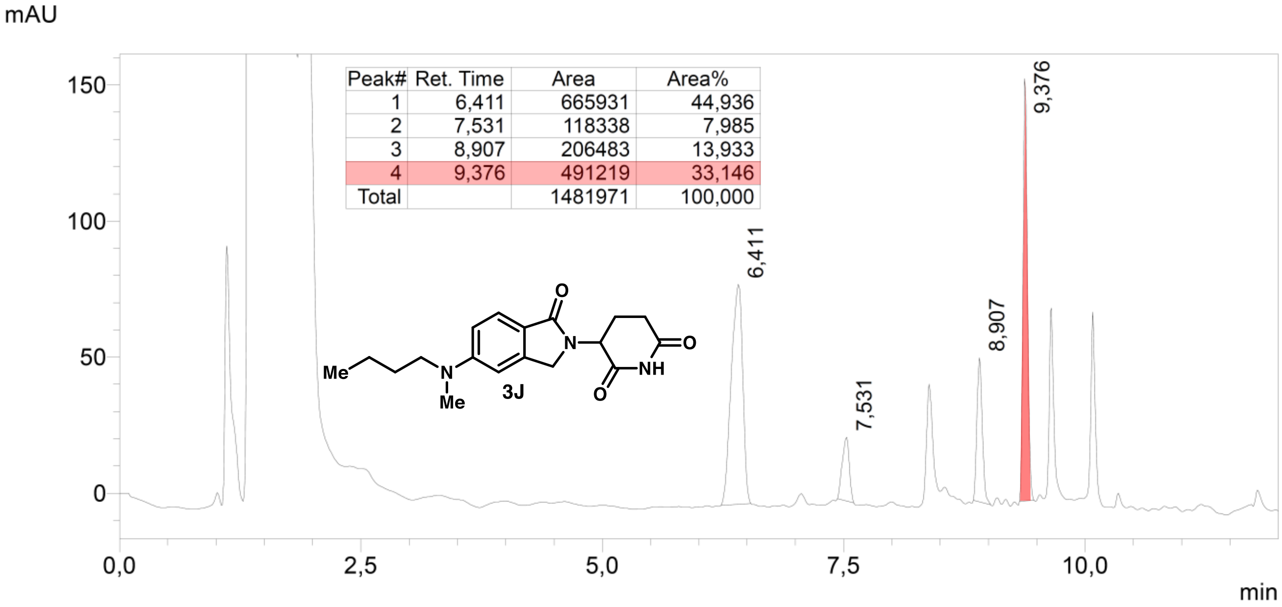

LC-MS: $[M + H]^+$ Calcd for $C_{18}H_{24}N_3O_3$ 330; Found 330.

Compound 3K

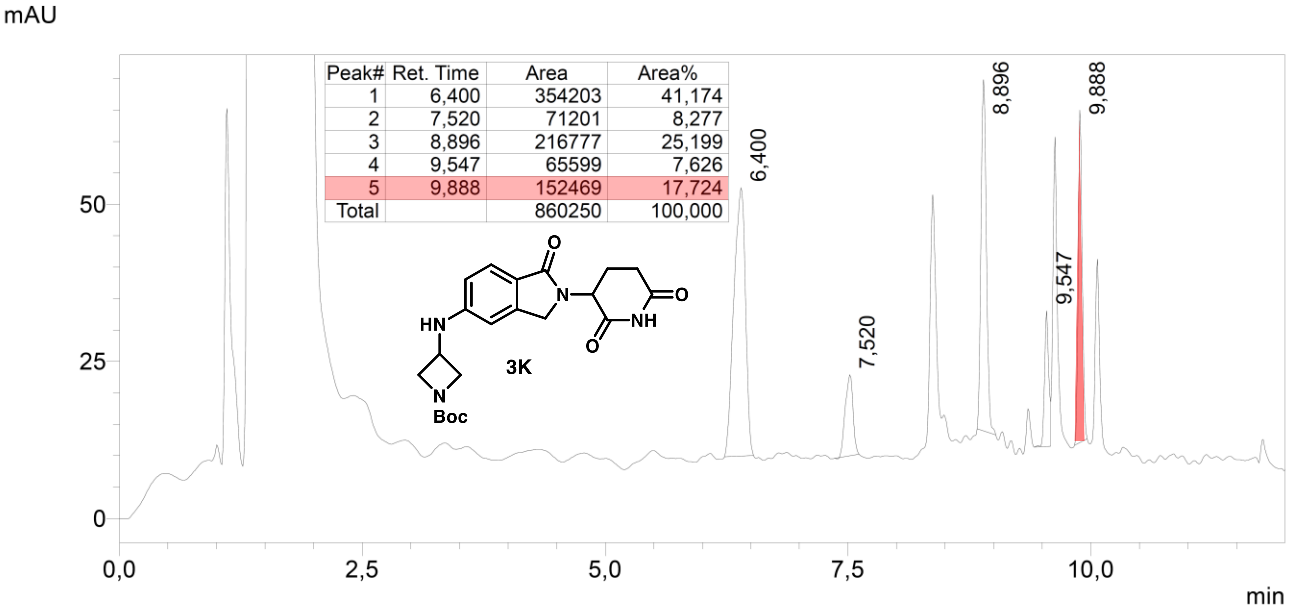

Peak#	Ret. Time	Area	Area%
1	6,400	354203	41,174
2	7,520	71201	8,277
3	8,896	216777	25,199
4	9,547	65599	7,626
5	9,888	152469	17,724
Total		860250	100,000

LC-MS: $[M + H]^+$ Calcd for $C_{21}H_{27}N_4O_5$ 414; Found 414.

Compound 4A

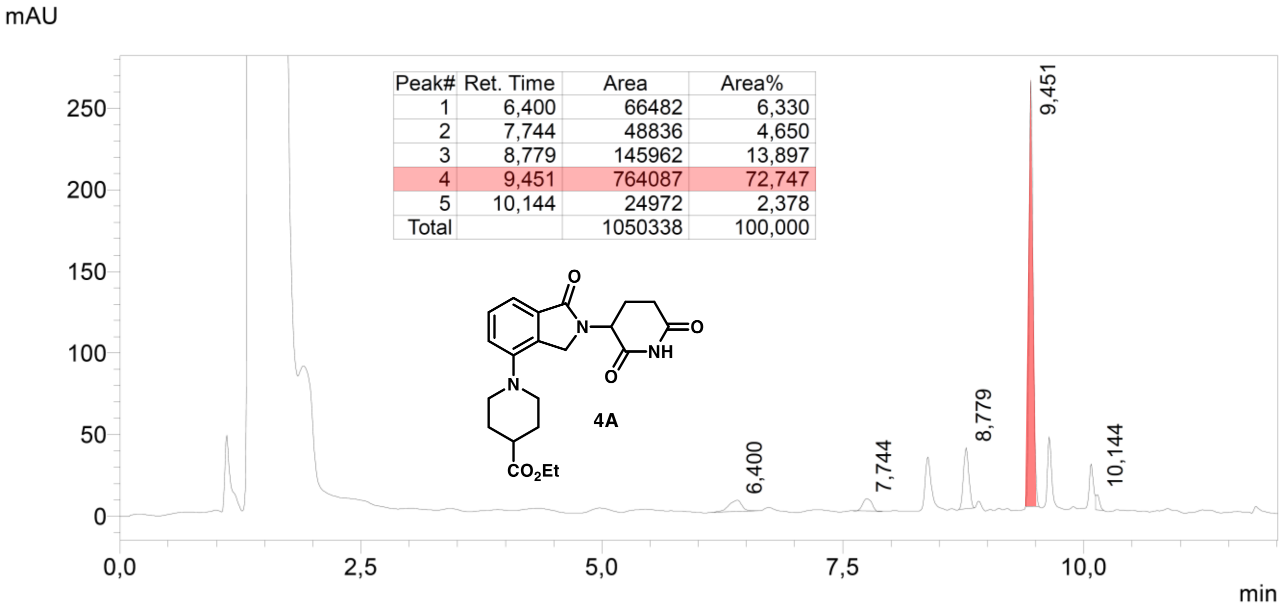

Peak#	Ret. Time	Area	Area%
1	6,400	66482	6,330
2	7,744	48836	4,650
3	8,779	145962	13,897
4	9,451	764087	72,747
5	10,144	24972	2,378
Total		1050338	100,000

LC-MS: $[M + H]^+$ Calcd for $C_{21}H_{26}N_3O_5$ 400; Found 400.

Compound 4B

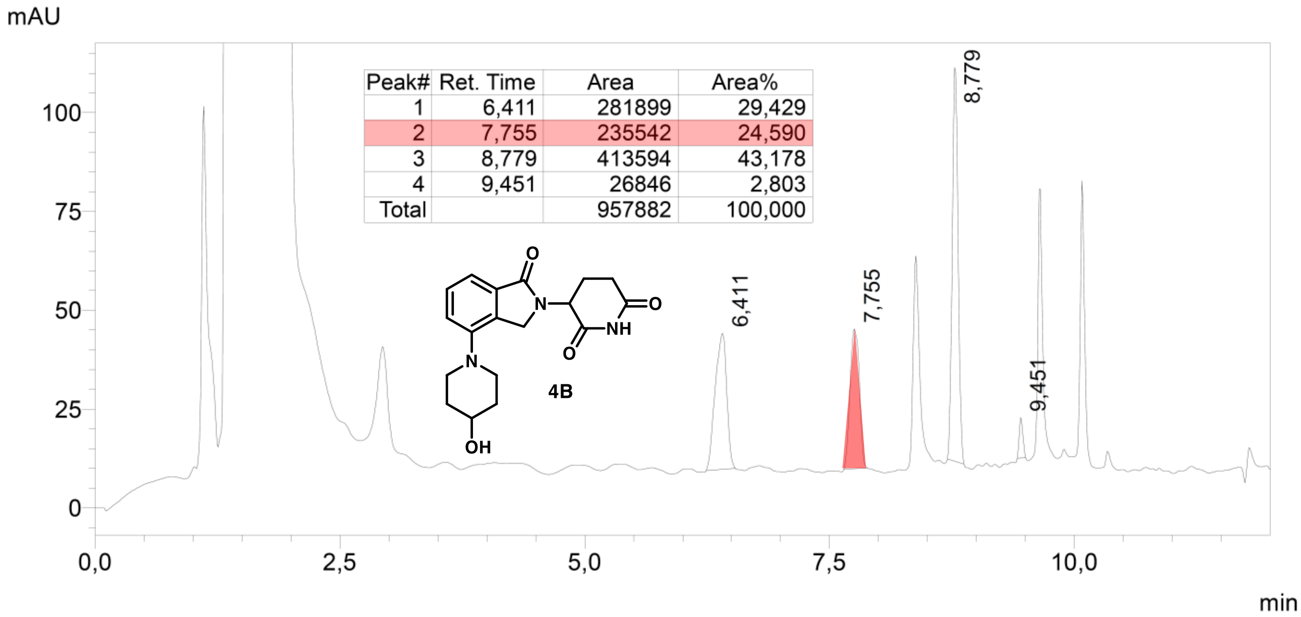

Peak#	Ret. Time	Area	Area%
1	6,411	281899	29,429
2	7,755	235542	24,590
3	8,779	413594	43,178
4	9,451	26846	2,803
Total		957882	100,000

LC-MS: $[M + H]^+$ Calcd for $C_{18}H_{22}N_3O_4$ 344; Found 344.

Compound 4C

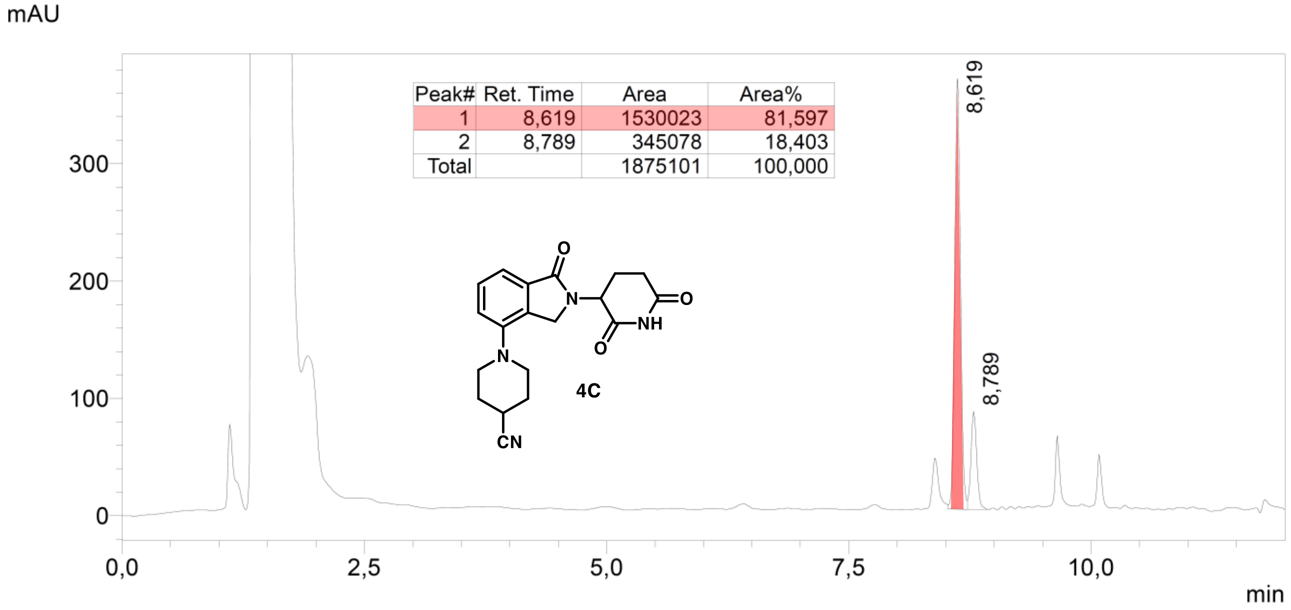

Peak#	Ret. Time	Area	Area%
1	8,619	1530023	81,597
2	8,789	345078	18,403
Total		1875101	100,000

LC-MS: $[M + H]^+$ Calcd for $C_{19}H_{21}N_4O_3$ 353; Found 353.

Compound 4D

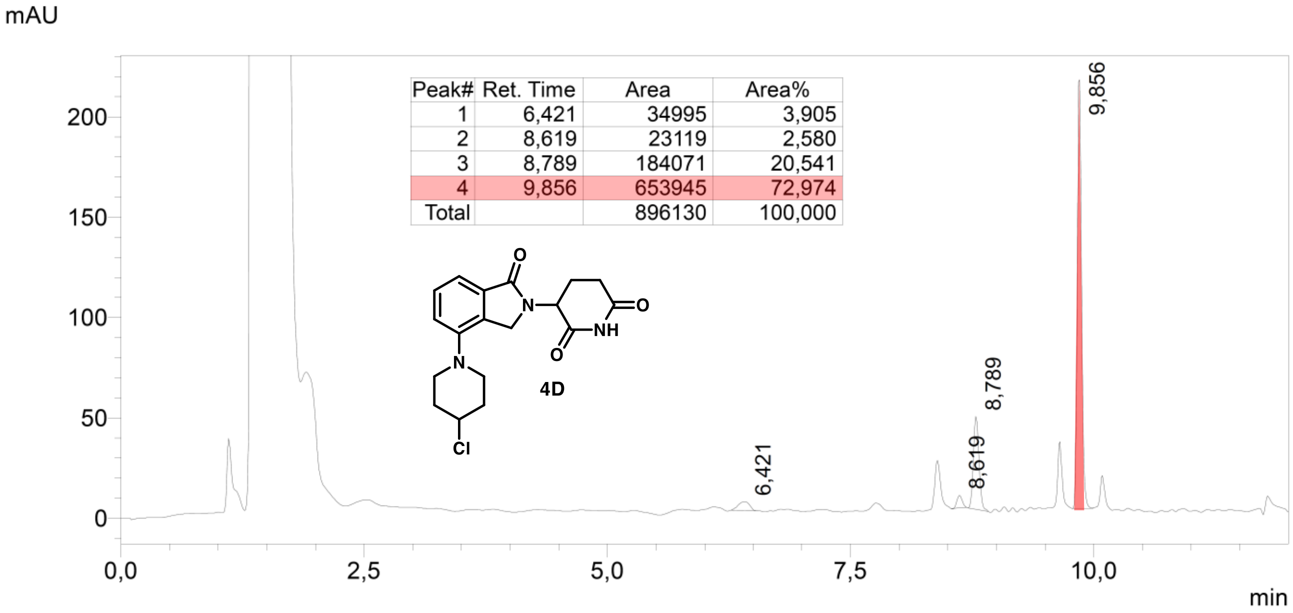

Peak#	Ret. Time	Area	Area%
1	6,421	34995	3,905
2	8,619	23119	2,580
3	8,789	184071	20,541
4	9,856	653945	72,974
Total		896130	100,000

LC-MS: $[M + H]^+$ Calcd for $C_{18}H_{21}ClN_3O_3$ 362; Found 362.

Compound 4E

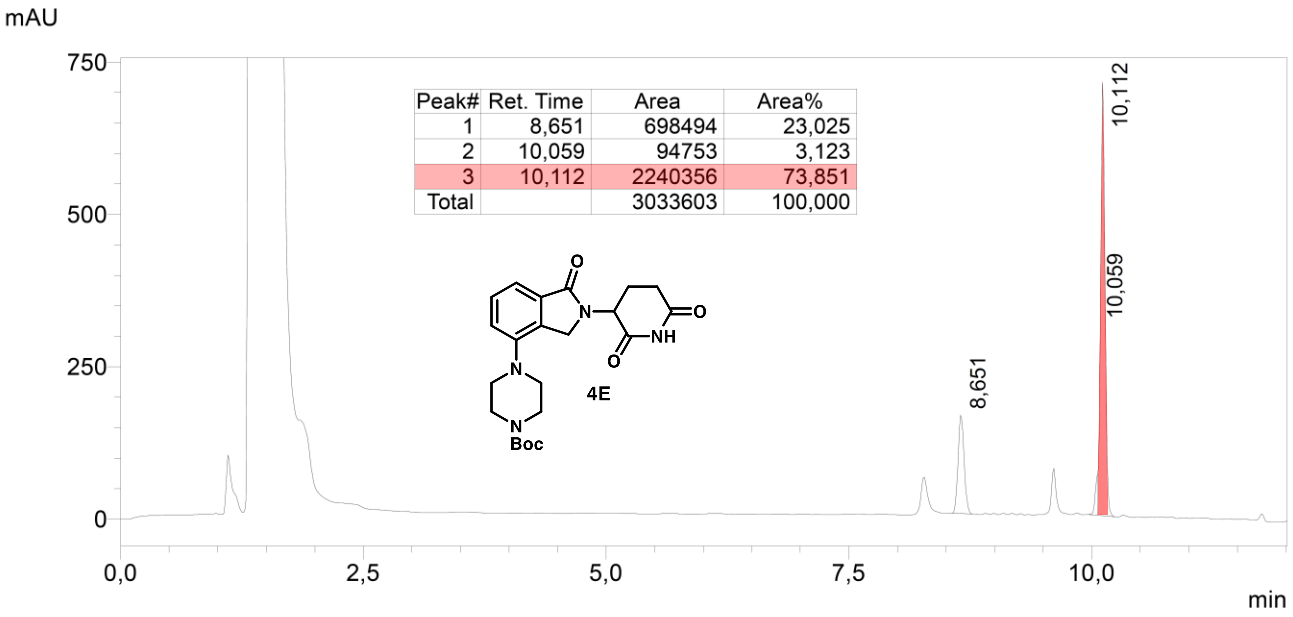

Peak#	Ret. Time	Area	Area%
1	8,651	698494	23,025
2	10,059	94753	3,123
3	10,112	2240356	73,851
Total		3033603	100,000

LC-MS: $[M + H]^+$ Calcd for $C_{22}H_{29}N_4O_5$ 429; Found 429.

Compound 4F

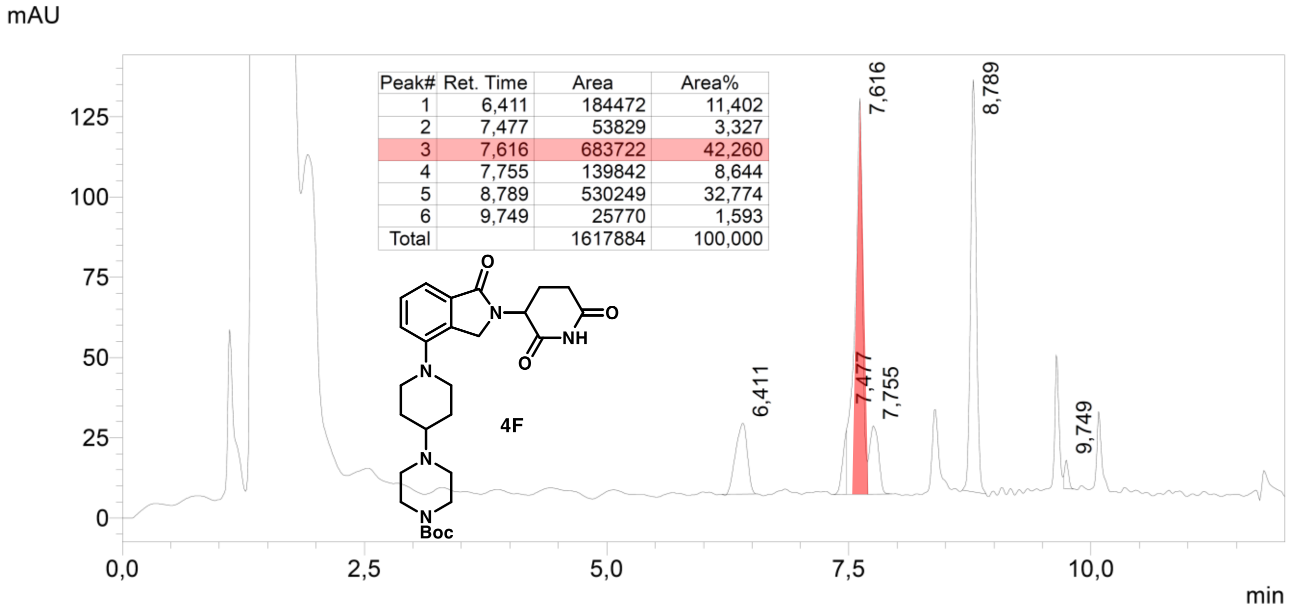

Peak#	Ret. Time	Area	Area%
1	6,411	184472	11,402
2	7,477	53829	3,327
3	7,616	683722	42,260
4	7,755	139842	8,644
5	8,789	530249	32,774
6	9,749	25770	1,593
Total		1617884	100,000

LC-MS: $[M + H]^+$ Calcd for $C_{27}H_{38}N_5O_5$ 512; Found 512.

Compound 4G

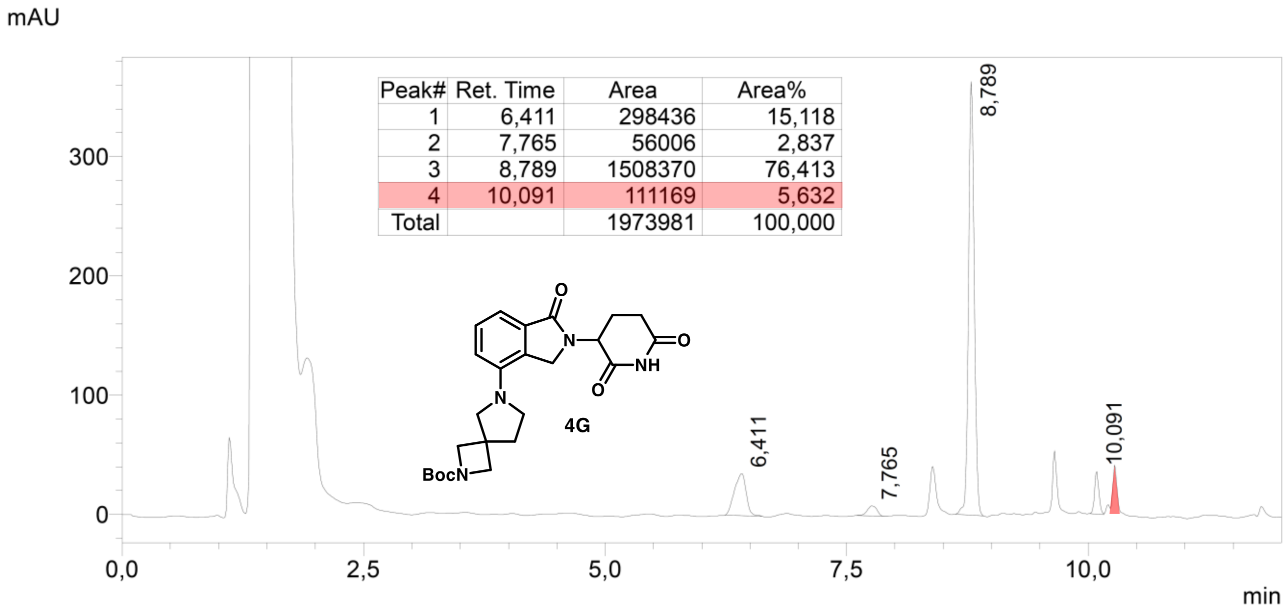

Peak#	Ret. Time	Area	Area%
1	6,411	298436	15,118
2	7,765	56006	2,837
3	8,789	1508370	76,413
4	10,091	111169	5,632
Total		1973981	100,000

LC-MS: $[M + H]^+$ Calcd for $C_{24}H_{31}N_4O_5$ 455; Found 455.

Compound 4H

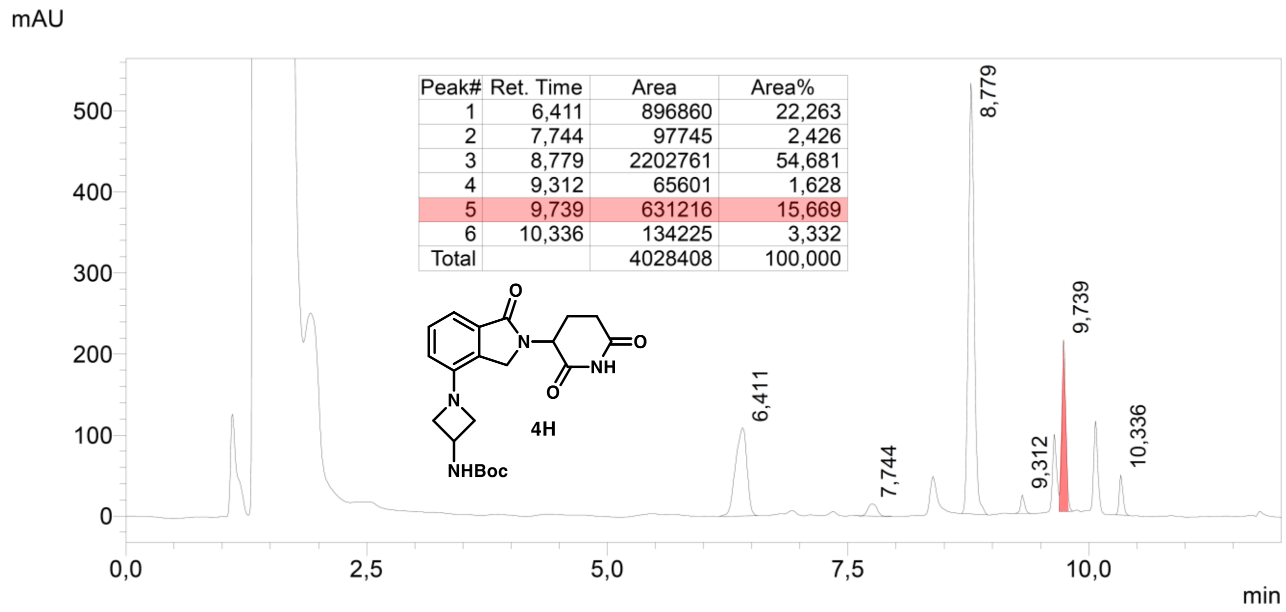

Peak#	Ret. Time	Area	Area%
1	6,411	896860	22,263
2	7,744	97745	2,426
3	8,779	2202761	54,681
4	9,312	65601	1,628
5	9,739	631216	15,669
6	10,336	134225	3,332
Total		4028408	100,000

LC-MS: $[M + H]^+$ Calcd for $C_{21}H_{27}N_4O_5$ 415; Found 415.

Compound 4I

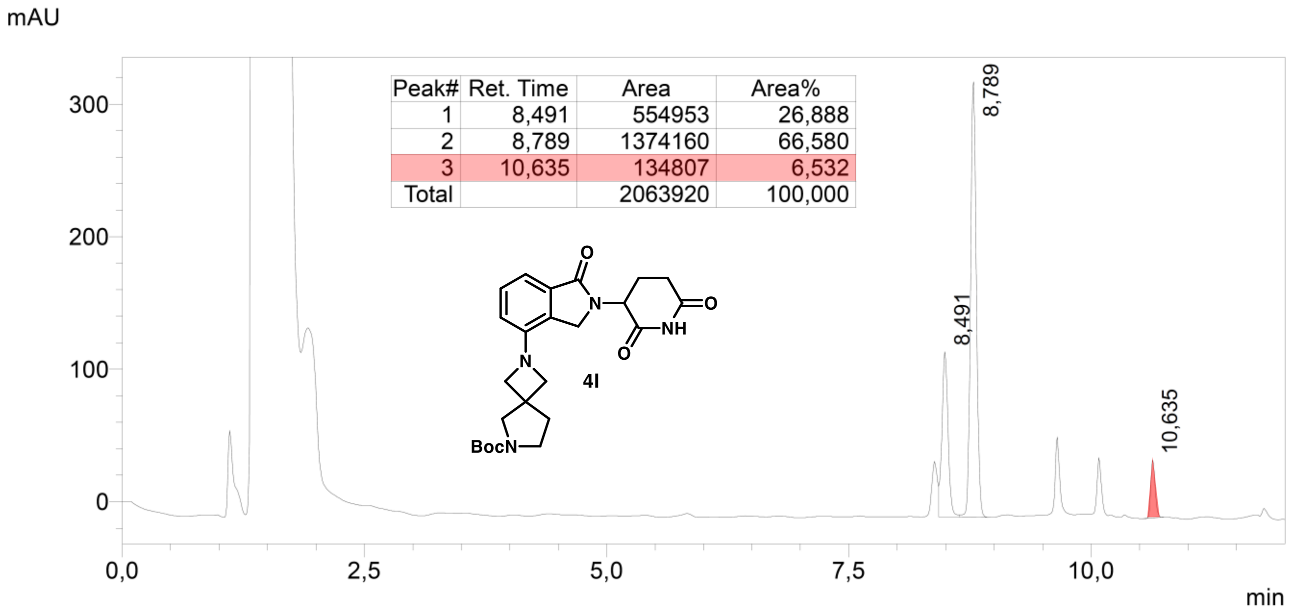

Peak#	Ret. Time	Area	Area%
1	8,491	554953	26,888
2	8,789	1374160	66,580
3	10,635	134807	6,532
Total		2063920	100,000

LC-MS: $[M + H]^+$ Calcd for $C_{24}H_{31}N_4O_5$ 455; Found 455

Compound 4J

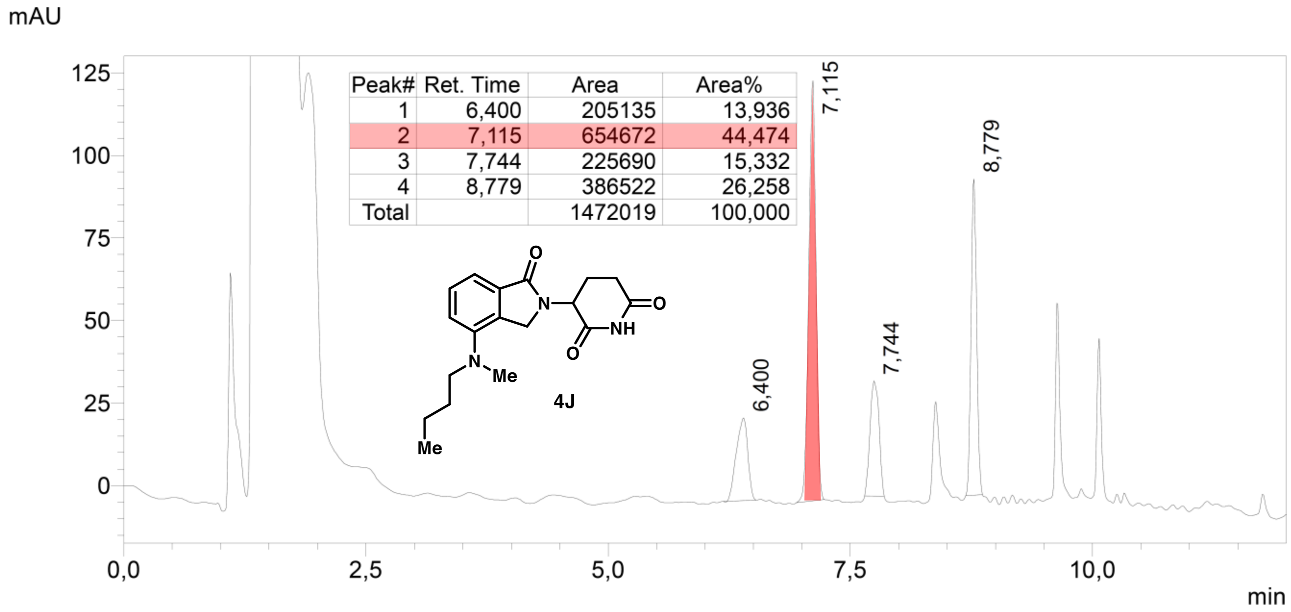

Peak#	Ret. Time	Area	Area%
1	6,400	205135	13,936
2	7,115	654672	44,474
3	7,744	225690	15,332
4	8,779	386522	26,258
Total		1472019	100,000

LC-MS: $[M + H]^+$ Calcd for $C_{18}H_{24}N_3O_3$ 330; Found 330.

Compound 4K

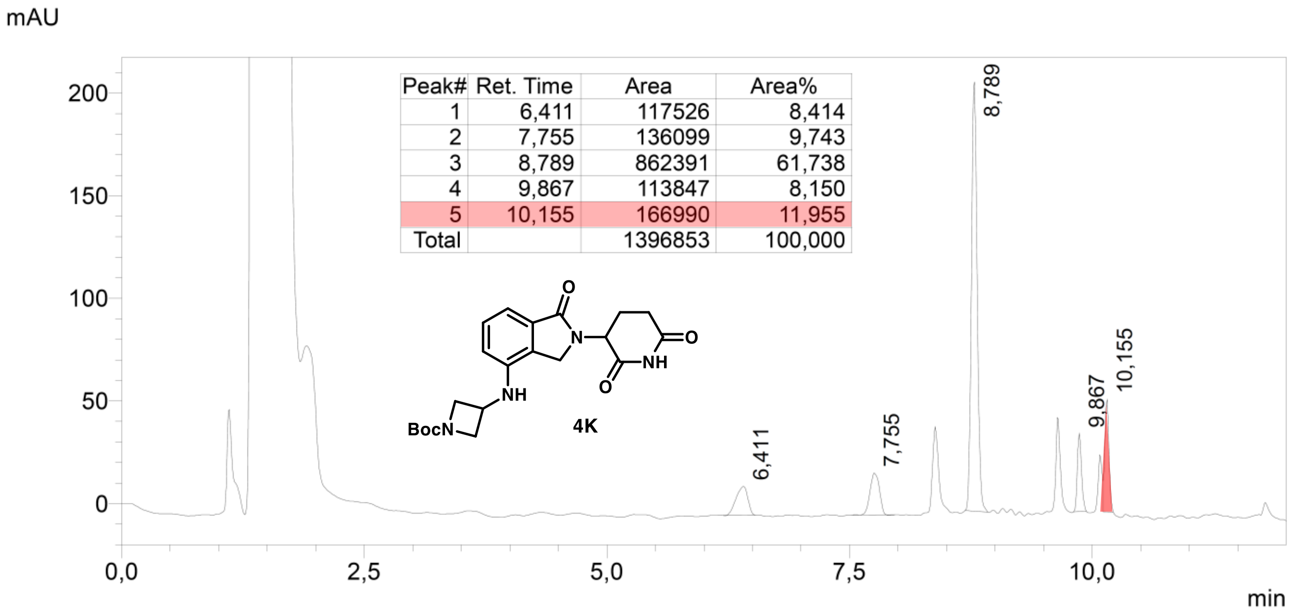

Peak#	Ret. Time	Area	Area%
1	6,411	117526	8,414
2	7,755	136099	9,743
3	8,789	862391	61,738
4	9,867	113847	8,150
5	10,155	166990	11,955
Total		1396853	100,000

LC-MS: [M - H]$^-$ Calcd for $C_{21}H_{25}N_4O_5$ 413; Found 413.

7. Design of Experiments (DoE)

Software and Experimental Design

To optimize the reaction of aryl bromide **4** with amine **I**, an experimental design was set up using experimental design software (Sartorius, Modde Pro v13.0.0). The input parameter ranges are displayed below (Table S1)

Table S2. Parameters, units ranges used for initial experimental design.

Parameter	Unit	Range
Current	mA	2 to 4.5
Amine	equiv.	2 to 5
Catalyst	mol%	20 to 40
Charge	F/mol	2 to 4.5

A face-centered cubic design was chosen (Figure S9), combining a reduced factorial (12 experiments, reduced from $2^4 = 16$), plus face points ($2 \times 4 = 8$) to determine quadratic effects. Three center points were added (as a measure of reproducibility), for a total of 23 experiments.

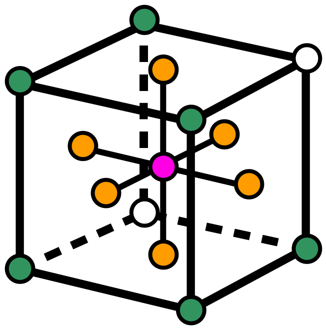

Figure S9. Schematic representation of the design used for this set of optimization experiments, based on a 3-parameter visualization. White circles represent experiments removed to achieve a reduced factorial.

Experimental Results

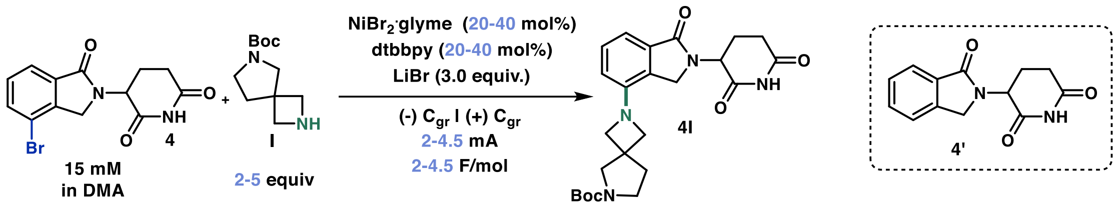

Table S3. Experimental conditions of the DoE experiments performed for automated reaction optimization.

Entry	Current (mA)	Amine (equiv.)	Catalyst (mol%)	Charge (F/mol)	4I (HPLC Area)	4’ (HPLC Area)	Remaining 4 (HPLC Area)
1	2	2	20	2	8%	9%	83%
2	4.5	2	20	2	4%	11%	82%
3	2	5	20	2	5%	8%	87%
4	4.5	2	40	2	8%	8%	81%
5	2	5	40	2	13%	3%	84%
6	4.5	5	40	2	5%	10%	81%
7	2	2	20	4.5	9%	19%	70%
8	2	5	20	4.5	3%	14%	79%
9	4.5	5	20	4.5	2%	24%	65%
10	2	2	40	4.5	32%	4%	62%
11	4.5	2	40	4.5	8%	64%	23%
12	4.5	5	40	4.5	6%	17%	77%
13	2	3.5	30	3.25	12%	13%	75%
14	4.5	3.5	30	3.25	5%	13%	77%
15	3.25	2	30	3.25	8%	14%	76%
16	3.25	5	30	3.25	4%	15%	79%
17	3.25	3.5	20	3.25	4%	14%	80%
18	3.25	3.5	40	3.25	6%	12%	82%
19	3.25	3.5	30	2	5%	8%	87%
20	3.25	3.5	30	4.5	6%	18%	73%
21	3.25	3.5	30	3.25	5%	12%	83%
22	3.25	3.5	30	3.25	4%	15%	81%
23	3.25	3.5	30	3.25	4%	10%	86%
**24**	**2**	**2**	**40**	**4.5**	**35%**	**9%**	**47%**

Initial Reaction Model

Using the experimental results, a multi-linear regression (MLR) model was constructed. Due to the significant number of low-yielding results, a log transformation ($\log_{10}$(Y)) was performed to obtain closer to a normal distribution. No outliers were present in the resulting model, and excellent model statistics were achieved (**Figure S10**).

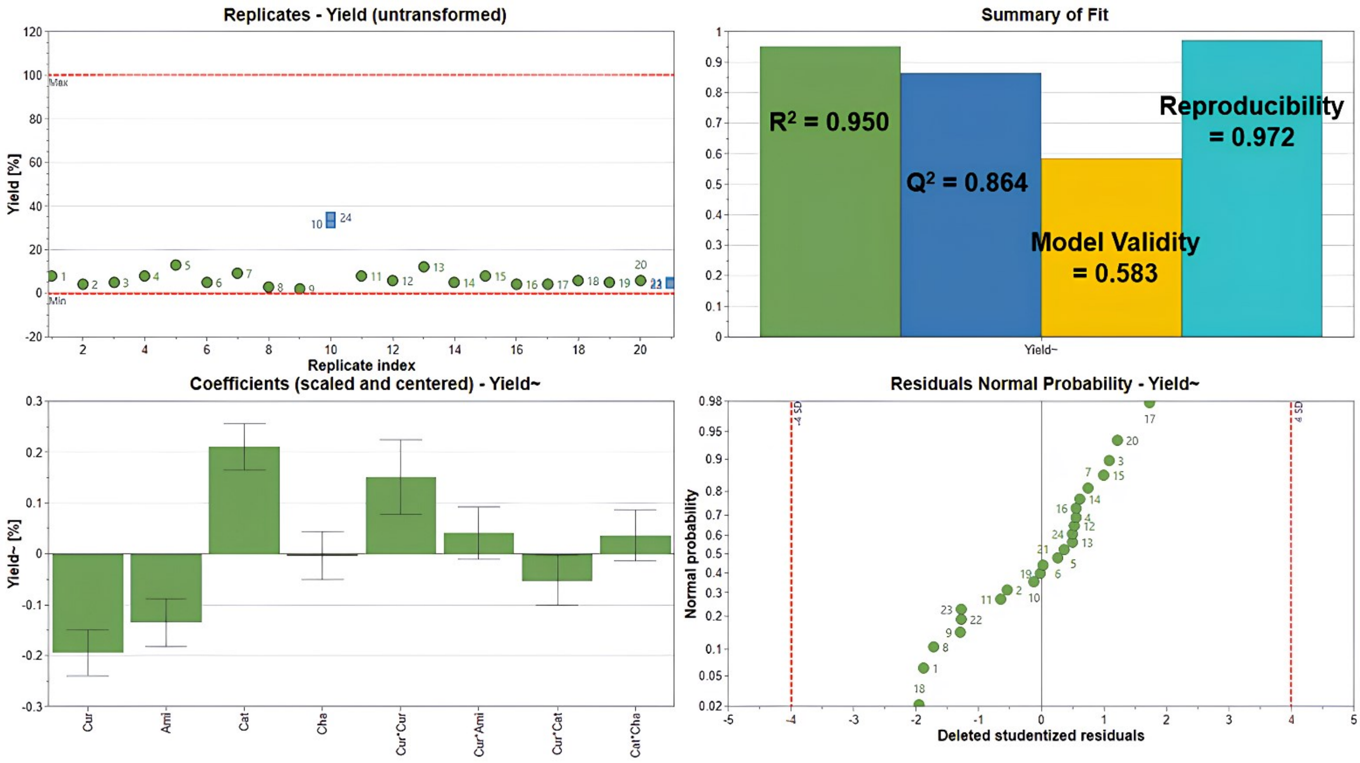

Figure S10. Summary of the model generated for yield (HPLC area%) of desired product **4l**. **Top left:** Replicates plot, showing the overall variation in the data and agreement of experimental replicates. **Top right:** Summary of fit statistics. R^2 is a measure of how well the model fits the experimental data points. Q^2 measures how well the model predicts future data. Reproducibility is a measure of experimental error. Model validity can be low in very good models due to very good replicates. **Bottom left:** The (scaled and centered) coefficients contributing to the calculated yield. This consists of four linear terms, one squared term (current) and three interaction terms. **Bottom right:** A normal probability plot of the residuals, showing no statistical outliers (±4 standard deviations).

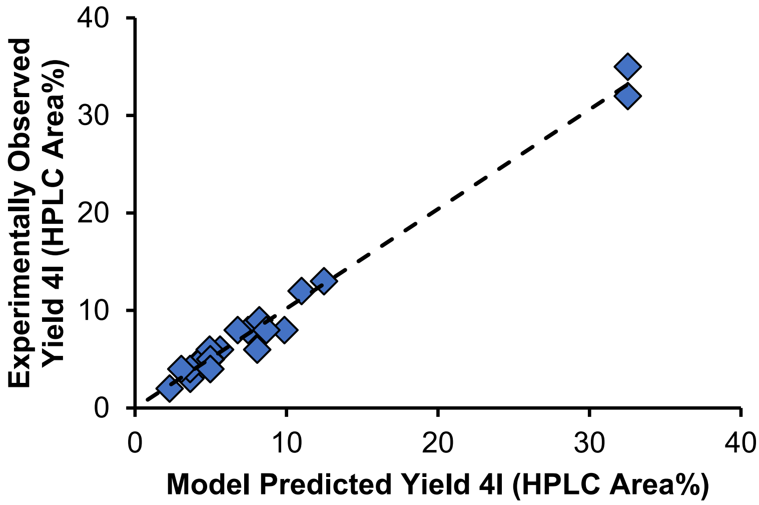

Figure S11. A plot of the predicted vs observed values from the MLR model, showing excellent agreement: R^2 = 0.950.

Table S4. Tabulated summary of the fitted coefficient values, contributing to the calculated yield in the model. Yield = 0.696175 – (0.19438 × Current) – (0.13505 × Amine) + (0.210801 × Catalyst) – (0.00383 × Charge) + (0.150761 × $Current^2$) + (0.040783 × Current × Amine) – (0.05208 × Current × Catalyst) + (0.036136 × Catalyst × Current).

	Coefficient magnitude.
Constant	0.696175
Current	-0.19438
Amine	-0.13505
Catalyst	0.210801
Charge	-0.00383
Current*Current	0.150761
Current*Amine	0.040783
Current*Catalyst	-0.05208
Catalyst*Charge	0.036136

Supplementary Experiments for Reaction Optimization

After performing the initial experiments and building the model, a selection of additional experiments was performed, in attempt to improve upon the previous best results. According to the original model, these experiments explored combinations of: low current, low amine, high catalyst and high charge. The results are shown below (**Table S5**).

Table S5 Experimental conditions of the second round of automated experiments for reaction optimization.

Entry	Current (mA)	Amine (equiv.)	Catalyst (equiv.)	Charge (F/mol)	**4l** (HPLC Area)	**4'** (HPLC Area)	Remaining **4** (HPLC Area)
1	2	2	0.7	4.5	27%	0%	64%
2	2	2	1	4.5	19%	0%	73%
3	1.5	2	0.4	3	29%	0%	71%
4	1.5	2	0.7	3	17%	0%	83%
5	1.5	2	1	3	11%	0%	87%
6	2	1.5	0.4	4.5	34%	3%	62%
7	2	1	0.4	4.5	25%	6%	69%

Revised Reaction Model

The reaction model was updated to include the results of these 7 additional experiments. The resulting model is summarized below (**Figure S12**).

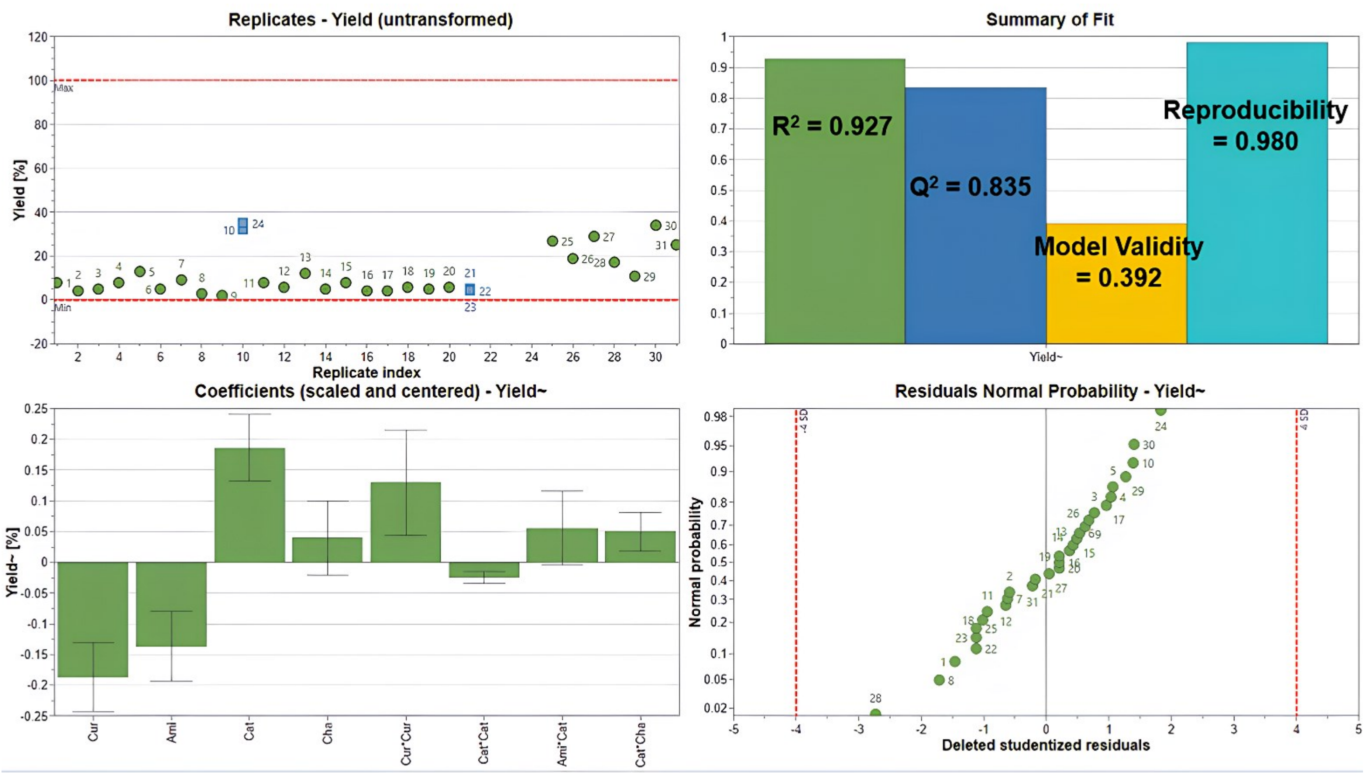

Figure S12. Summary of the model generated for yield (HPLC area%) of desired product **4l**. **Top left:** Replicates plot, showing the overall variation in the data and agreement of experimental replicates. **Top right:** Summary of fit statistics. R^2 is a measure of how well the model fits the experimental data points. Q^2 measures how well the model predicts future data. Reproducibility is a measure of experimental error. Model validity can be low in very good models due to very good replicates. **Bottom left:** The (scaled and centered) coefficients contributing to the calculated yield. This consists of four linear terms, one squared term (current) and three interaction terms. **Bottom right:** A normal probability plot of the residuals, showing no statistical outliers (±4 standard deviations).

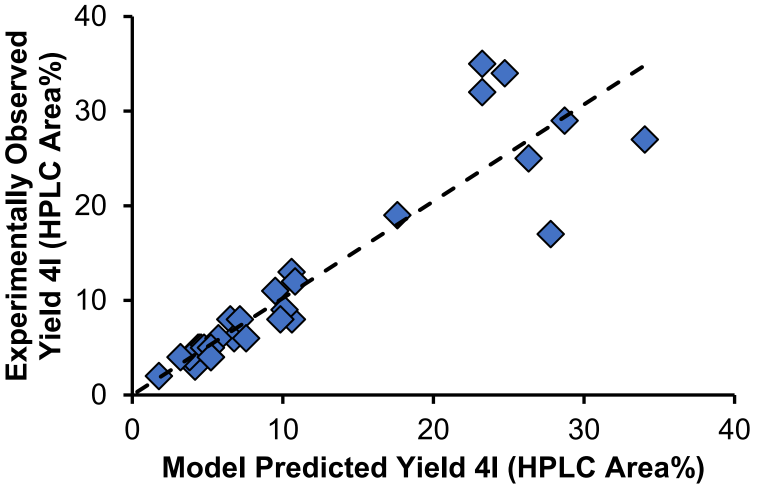

Figure S13. A plot of the predicted vs observed values from the revised MLR model. Although the newly added points are less well predicted, there is still a good overall agreement: $R^2 = 0.927$.

Since the newly added points were outside the parameter ranges of the initial design, it is to be expected that these results do not fit as closely to the model. However, the model remains valid and has useful predictive power. A surface plot showing the results of this model is presented in the manuscript **Figure 3**.

8. Adjustment of the Reaction Conditions

Batch Experimentation (ElectraSyn 2.0)

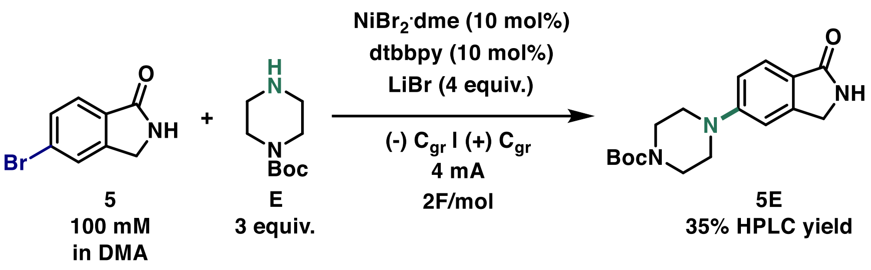

The aryl bromide **5** was chosen as a model substrate for the preliminary experiments due to its lower chemical complexity and high commercial availability. With the goal of transferring the conditions to flow, the porous electrodes used in the original conditions (Ni_{foam} and RVC) were substituted with impervious graphite for both the anode and cathode. After that change, the HPLC yield (calibrated vs biphenyl as internal standard) obtained was 35%.

Different Ni sources ($NiCl_2$·dme, Ni(II)Br_2, Ni(II) acac, Ni(II)I_2), ligands (bipyridine, methoxybipyridine, CF_3 bipyridine), cathode materials (Fe, Sn, Ti, B, Al, Pb, Ni) and amine loading were tested but none of them provided a better result. However, a higher catalyst loading, and a lower electrolyte loading were found to have a positive impact on the reaction.

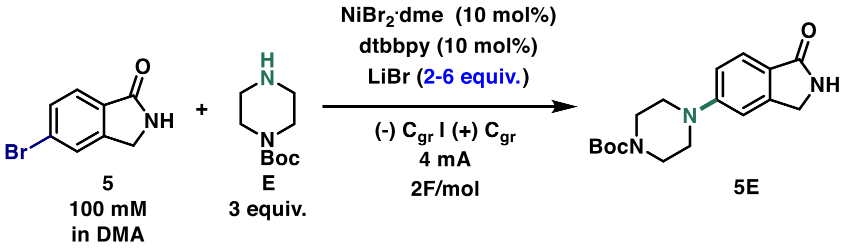

Table S6. optimization of the electrolyte loading in batch.

Entry	LiBr (equiv.)	Conversion (%)	5E HPLC Yield (%)
1	2	74	26
2	3	82	41
3	4	80	35
4	5	88	29
5	6	71	15

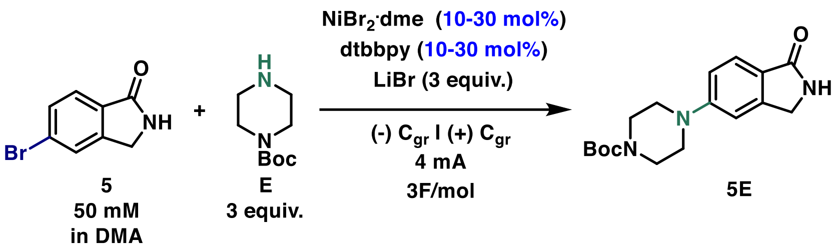

The higher catalyst loading translated into a decrease of the dehalogenation of the starting material (main side product of the reaction). This means that whereas the conversion decreased, the selectivity of the reaction was greatly improved. In order to compensate the lower conversion, the charge applied was augmented to 3F/mol. Moreover, it was also observed that the reaction benefited from a decrease in concentration to 50 mM.

Table S7. optimization of the catalyst loading in batch.

Entry	Catalyst (mol%)	Conversion (%)	5E HPLC Yield (%)
1	15	62	55
2	20	57	61
3	30	53	70

Continuous-Flow Experimentation

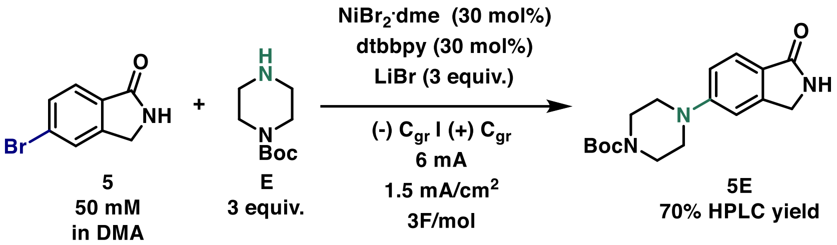

The transfer of the reaction conditions from batch to single-pass flow only needed to adjust the current density (from 4 mA/cm^2 to 1.5 mA/cm^2), obtaining a similar result. At this point, a channel layer with lower surface was used (4.1 cm^2).

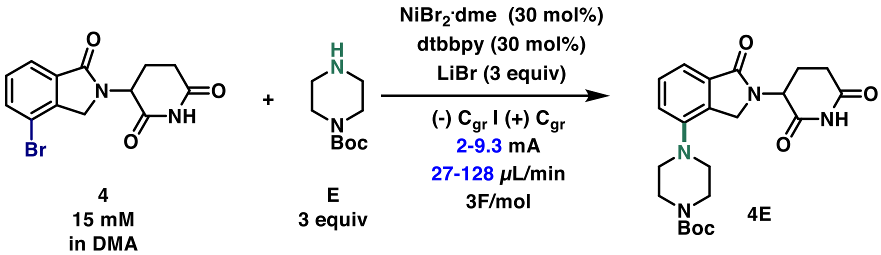

After obtaining a good result for this transformation in flow with the model substrate, the aryl bromide **4** and the amine **E** were tested, as a readjustment of the reaction conditions was needed for these challenging substrates. The main reason is that the aryl bromides containing the thalidomide and lenalidomide moieties are more prone to direct dehalogenation. Thus, lowering the current density was needed one more time.

Furthermore, the reaction concentration was decreased due to the poor solubility of the aryl bromides in DMA. 25 mM was the maximum identified and therefore that was the concentration chosen for the stock solutions. That meant that the reaction concentration decreased to 15 mM.

In order to be able to have higher flow rates, the flow channel layer was changed for one with higher area (6.4 cm^2).

Table S8. Optimization of the current density in flow.

Entry	Current density (mA/cm^2)	Flow rate (µL/min)	4E HPLC Area (%)
1	0.3	27	61
2	0.55	48	70
3	0.72	63	67
4	1.45	128	35

Thus, 0.55 mA/cm^2 and 48 µL/min were chosen as the best flow electrochemical parameters for the library synthesis.

Reaction Conditions Comparison

The last comparison was done between the original conditions reported by Baran and coworkers,[4] and the continuous-flow optimized conditions both in flow and batch.

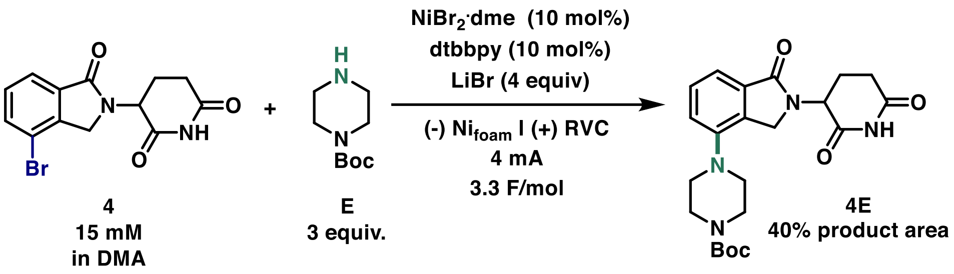

Original conditions from Baran and coworkers applied to the coupling between aryl bromide 4 and amine E gave a 40% HPLC yield.

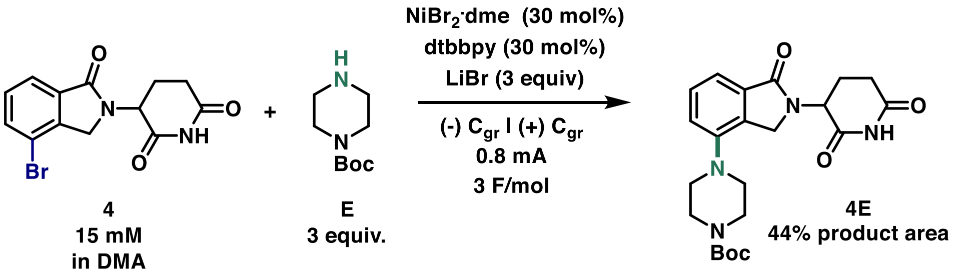

The optimized flow conditions applied to batch (ElectraSyn) resulted in the formation of **4E** in 44% HPLC yield.

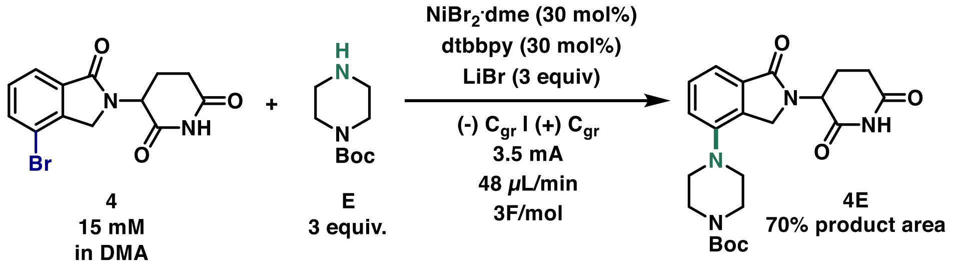

Among the three experiments, the reaction performed in single-pass continuous-flow was the one that provided better result (70% HPLC yield).

9. Compound Characterization

Compound 1A

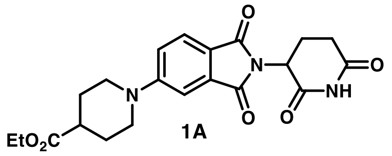

Following the General Procedure A with aryl bromide 1 (50.6 mg, 0.15 mmol, 1.0 equiv.), amine A (70.7 mg, 0.45 mmol, 3.0 equiv.), LiBr (39.1 mg, 0.45 mmol, 3.0 equiv.), $NiBr_2$·dme (13.8 mg, 0.045 mmol, 30% mol) and 4,4′-di-tert-butyl-2,2′-dipyridyl (12.1 mg, 0.045 mmol, 30% mol) in dimethylacetamide (10 mL), an HPLC product area of 80% was obtained. After work-up, the crude was purified by supercritical fluid chromatography (Viridis BEH 2-EP, Agilent 1260, eluents: CO_2 = 85%, Isopropanol = 15%, flow rate = 4.0 mL/min) to afford 27.9 mg (76%) of the title compound.

^{1}H NMR (700 MHz, DMSO-d$_6$): δ 11.07 (s, 1H), 7.66 (d, J = 8.5 Hz, 1H), 7.33 (s, 1H), 7.28 – 7.21 (m, 1H), 5.06 (dd, J = 12.9, 5.5 Hz, 1H), 4.08 (q, J = 7.1 Hz, 2H), 4.01 – 3.93 (m, 2H), 3.14 – 3.04 (m, 2H), 2.92 – 2.84 (m, 1H), 2.69 – 2.62 (m, 1H), 2.61 – 2.56 (m, 1H), 2.56 – 2.51 (m, 1H), 2.05 – 1.98 (m, 1H), 1.94 – 1.86 (m, 2H), 1.65 – 1.56 (m, 2H), 1.18 (t, J = 7.1 Hz, 3H).

^{13}C NMR (176 MHz, DMSO-d$_6$): δ 174.1, 172.9, 171.3, 168.3, 153.4, 144.1, 123.8, 121.1, 114.9, 108.6, 59.9, 51.4, 47.2, 46.9, 31.2, 30.3, 27.1, 22.6, 14.1.

HRMS (ESI-TOF) m/z: $[M + H]^+$ Calcd for $C_{21}H_{24}N_3O_6$ 414.1660; Found 414.1656.

Compound 1B

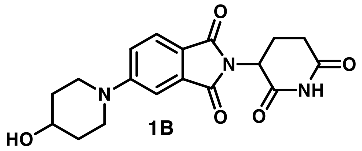

Following the General Procedure A with aryl bromide 1 (50.6 mg, 0.15 mmol, 1.0 equiv.), amine B (45.5 mg, 0.45 mmol, 3.0 equiv.), LiBr (39.1 mg, 0.45 mmol, 3.0 equiv.), $NiBr_2$·dme (13.8 mg, 0.045 mmol, 30% mol) and 4,4′-di-tert-butyl-2,2′-dipyridyl (12.1 mg, 0.045 mmol, 30% mol) in dimethylacetamide (10 mL), an HPLC product area of 82% was obtained. After work-up, the crude was purified by supercritical fluid chromatography (ChiralCel OD-H, THAR SFC, eluents: CO_2 = 70%, MeOH = 30%, flow rate = 5.0 mL/min) to afford 22.6 mg (62%) of the title compound.

^{1}H NMR (400 MHz, DMSO-d$_6$): δ 11.04 (s, 1H), 7.64 (d, J = 8.5 Hz, 1H), 7.31 (d, J = 2.4 Hz, 1H), 7.23 (dd, J = 8.7, 2.4 Hz, 1H), 5.05 (dd, J = 12.9, 5.4 Hz, 1H), 4.72 (d, J = 4.2 Hz, 1H), 3.87 – 3.78 (m, 2H), 3.78 – 3.70 (m, 1H), 3.25 – 3.15 (m, 2H), 2.94 – 2.81 (m, 1H), 2.63 – 2.52 (m, 2H), 2.06 – 1.96 (m, 1H), 1.85 – 1.76 (m, 2H), 1.49 – 1.36 (m, 2H).

^{13}C NMR (126 MHz, DMSO-d$_6$): δ 171.2, 168.5, 166.0, 165.3, 153.1, 132.4, 123.4, 115.9, 115.8, 106.0, 63.9, 47.1, 43.3, 31.6, 29.3, 20.5.

HRMS (ESI-TOF) m/z: $[M + H]^+$ Calcd for $C_{18}H_{20}N_3O_5$ 358.1397; Found 358.1389.

Compound 1C

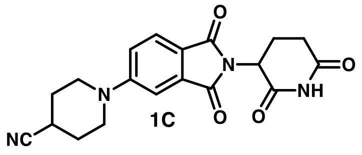

Following the General Procedure A with aryl bromide 1 (50.6 mg, 0.15 mmol, 1.0 equiv.), amine C (49.5 mg, 0.45 mmol, 3.0 equiv.), LiBr (39.1 mg, 0.45 mmol, 3.0 equiv.), $NiBr_2$·dme (13.8 mg, 0.045 mmol, 30% mol) and 4,4′-di-tert-butyl-2,2′-dipyridyl (12.1 mg, 0.045 mmol, 30% mol) in dimethylacetamide (10 mL), an HPLC product area of 86% was obtained. After work-up, the crude was purified by supercritical fluid chromatography (Viridis BEH 2-EP, Agilent 1260, eluents: CO_2 = 85%, Isopropanol = 15%, flow rate = 4.0 mL/min) to afford 28.5 mg (60%) of the title compound.

^{1}H NMR (700 MHz, DMSO-d$_6$): δ 11.07 (s, 1H), 7.68 (d, J = 8.6 Hz, 1H), 7.37 (d, J = 2.4 Hz, 1H), 7.27 (dd, J = 8.6, 2.4 Hz, 1H), 5.07 (dd, J = 12.9, 5.5 Hz, 1H), 3.74 – 3.67 (m, 2H), 3.40 – 3.34 (m, 2H), 3.18 – 3.12 (m, 1H), 2.92 – 2.84 (m, 1H), 2.62 – 2.56 (m, 1H), 2.56 – 2.50 (m, 1H), 2.05 – 1.99 (m, 1H), 1.99 – 1.94 (m, 2H), 1.82 – 1.74 (m, 2H).

^{13}C NMR (176 MHz, DMSO-d$_6$): δ 172.78, 170.05, 167.51, 166.92, 154.67, 133.95, 124.98, 121.97, 118.34, 118.06, 108.27, 48.76, 45.66, 30.97, 27.32, 25.24, 22.16.

HRMS (ESI-TOF) m/z: $[M + H]^+$ Calcd for $C_{19}H_{19}N_4O_4$ 367.1401; Found 367.1500.

Compound 1D

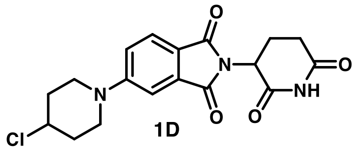

Following the General Procedure A with aryl bromide 1 (50.6 mg, 0.15 mmol, 1.0 equiv.), amine D (53.6 mg, 0.45 mmol, 3.0 equiv.), LiBr (39.1 mg, 0.45 mmol, 3.0 equiv.), $NiBr_2$·dme (13.8 mg, 0.045 mmol, 30% mol) and 4,4′-di-tert-butyl-2,2′-dipyridyl (12.1 mg, 0.045 mmol, 30% mol) in dimethylacetamide (10 mL), an HPLC product area of 83% was obtained. After work-up, the crude was purified by supercritical fluid chromatography (Viridis Silica 2-Ethylpyridin 150-30, Agilent 1260, eluents: CO_2 = 90%, Isopropanol = 10%, flow rate = 4.0 mL/min) to afford 32.8 mg (65%) of the title compound.

^{1}H NMR (400 MHz, DMSO-d$_6$): δ 11.04 (s, 1H), 7.67 (d, J = 8.5 Hz, 1H), 7.37 (d, J = 2.3 Hz, 1H), 7.28 (dd, J = 8.6, 2.4 Hz, 1H), 5.06 (dd, J = 12.9, 5.4 Hz, 1H), 4.53 – 4.43 (m, 1H), 3.83 – 3.76 (m, 2H), 3.44 – 3.33 (m, 2H), 2.95 – 2.81 (m, 1H), 2.64 – 2.51 (m, 2H), 2.19 – 2.10 (m, 2H), 2.07 – 1.96 (m, 1H), 1.86 – 1.73 (m, 2H).

^{13}C NMR (126 MHz, DMSO-d$_6$): δ 171.1, 165.9, 165.3, 152.9, 132.4, 123.4, 116.5, 116.3, 106.5, 56.0, 47.1, 43.6, 32.5, 29.3, 20.5.

HRMS (ESI-TOF) m/z: $[M + H]^+$ Calcd for $C_{18}H_{19}N_3O_4Cl$ 376.1059; Found 376.1050.

Compound 1E

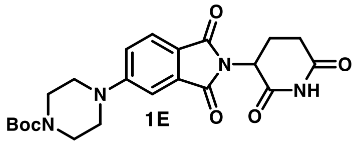

Following the General Procedure A with aryl bromide 1 (50.6 mg, 0.15 mmol, 1.0 equiv.), amine E (83.7 mg, 0.45 mmol, 3.0 equiv.), LiBr (39.1 mg, 0.45 mmol, 3.0 equiv.), $NiBr_2$·dme (13.8 mg, 0.045 mmol, 30% mol) and 4,4′-di-tert-butyl-2,2′-dipyridyl (12.1 mg, 0.045 mmol, 30% mol) in dimethylacetamide (10 mL), an HPLC product area of 80% was obtained. After work-up, the crude was purified by supercritical fluid chromatography (ChiralPak IH, THAR SFC, eluents: CO_2 = 85%, Methanol = 15%, flow rate = 5.0 mL/min) to afford 36.8 mg (57%) of the title compound.

^{1}H NMR (400 MHz, DMSO-d$_6$): δ 11.05 (s, 1H), 7.69 (d, J = 8.5 Hz, 1H), 7.34 (d, J = 2.3 Hz, 1H), 7.24 (dd, J = 8.6, 2.4 Hz, 1H), 5.07 (dd, J = 12.9, 5.4 Hz, 1H), 3.47 (s, 8H), 2.95 – 2.81 (m, 1H), 2.64 – 2.51 (m, 2H), 2.07 – 1.96 (m, 1H), 1.43 (s, 9H).

^{13}C NMR (126 MHz, DMSO-d$_6$): δ 172.8, 170.0, 167.5, 166.9, 155.0, 153.8, 133.8, 124.9, 118.5, 117.9, 108.0, 79.1, 48.8, 46.6, 31.0, 28.0, 22.2.

HRMS (ESI-TOF) m/z: $[M + H]^+$ Calcd for $C_{22}H_{27}N_4O_6$ 443.1925; Found 443.1911.

Compound 1F

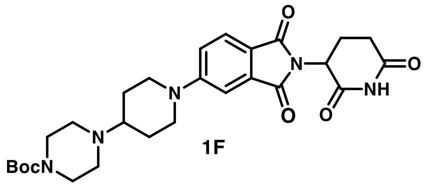

Following the General Procedure A with aryl bromide 1 (50.6 mg, 0.15 mmol, 1.0 equiv.), amine F (121 mg, 0.45 mmol, 3.0 equiv.), LiBr (39.1 mg, 0.45 mmol, 3.0 equiv.), $NiBr_2$·dme (13.8 mg, 0.045 mmol, 30% mol) and 4,4′-di-tert-butyl-2,2′-dipyridyl (12.1 mg, 0.045 mmol, 30% mol) in dimethylacetamide (10 mL), an HPLC product area of 52% was obtained. After work-up, the crude was purified by supercritical fluid chromatography (Viridis Silica 2-EP, Agilent 1260, eluents: CO_2 = 85%, Isopropanol = 15%, flow rate = 4.0 mL/min) to afford 34.6 mg (47%) of the title compound.

^{1}H NMR (700 MHz, DMSO-d$_6$): δ 11.07 (s, 1H), 7.65 (d, J = 8.5 Hz, 1H), 7.32 (d, J = 2.4 Hz, 1H), 7.24 (dd, J = 8.6, 2.4 Hz, 1H), 5.06 (dd, J = 12.9, 5.5 Hz, 1H), 4.07 (d, J = 12.9 Hz, 2H), 3.30 – 3.24 (m, 4H), 2.98 – 2.92 (m, 2H), 2.92 – 2.84 (m, 1H), 2.61 – 2.51 (m, 3H), 2.46 – 2.39 (m, 4H), 2.04 – 1.98 (m, 1H), 1.85 – 1.79 (m, 2H), 1.49 – 1.41 (m, 2H), 1.38 (s, 9H).

^{13}C NMR (176 MHz, DMSO-d$_6$): δ 172.8, 170.1, 167.6, 166.9, 154.7, 153.8, 134.0, 125.0, 117.7, 117.6, 107.8, 78.7, 62.0, 60.6, 48.7, 48.5, 46.6, 31.0, 28.1, 27.1, 25.5, 22.2.

HRMS (ESI-TOF) m/z: $[M + H]^+$ Calcd for $C_{27}H_{36}N_5O_6$ 526.2660; Found 526.2665.

Compound 1G

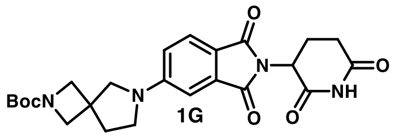

Following the General Procedure B with aryl bromide 1 (101 mg, 0.30 mmol, 1.0 equiv.), amine G (191 mg, 0.90 mmol, 3.0 equiv.), LiBr (78.2 mg, 0.90 mmol, 3.0 equiv.), $NiBr_2$·dme (27.6 mg, 0.090 mmol, 30% mol) and 4,4′-di-tert-butyl-2,2′-dipyridyl (24.2 mg, 0.090 mmol, 30% mol) in dimethylacetamide (20 mL), an HPLC product area of 38% was obtained. After work-up, the crude was purified by preparative liquid chromatography (XSelect CSH C18, Agilent D50 454 Prep-MS, eluents: H_2O + 0.1% HCOOH, MeCN, flow rate = 60 mL/min) to afford 26.7 mg (25%) of the title compound.

^{1}H NMR (700 MHz, DMSO-d$_6$): δ 11.05 (s, 1H), 7.65 (d, J = 8.4 Hz, 1H), 6.90 (d, J = 2.3 Hz, 1H), 6.80 (dd, J = 8.6, 2.3 Hz, 1H), 5.05 (dd, J = 12.9, 5.4 Hz, 1H), 3.84 (d, J = 37.9 Hz, 4H), 3.61 (s, 2H), 3.45 (t, J = 6.9 Hz, 2H), 2.92 – 2.84 (m, 1H), 2.61 – 2.56 (m, 1H), 2.53 – 2.50 (m, 1H), 2.21 (t, J = 7.0 Hz, 2H), 2.04 – 1.98 (m, 1H), 1.39 (s, 9H).

^{13}C NMR (176 MHz, DMSO-d$_6$): δ 172.8, 170.1, 167.6, 167.2, 155.5, 151.8, 134.0, 124.9, 115.9, 115.3, 105.6, 78.6, 56.8, 48.7, 46.7, 39.9, 39.8, 39.6, 39.5, 39.4, 39.3, 39.2, 35.1, 31.0, 28.1, 22.2.

HRMS (ESI-TOF) m/z: $[M + H]^+$ Calcd for $C_{24}H_{29}N_4O_6$ 469.2082; Found 469.2070.

Compound 1H

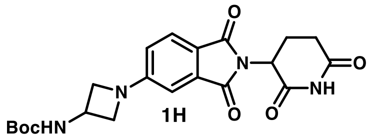

Following the General Procedure B with aryl bromide 1 (101 mg, 0.30 mmol, 1.0 equiv.), amine H (155 mg, 0.90 mmol, 3.0 equiv.), LiBr (78.2 mg, 0.90 mmol, 3.0 equiv.), $NiBr_2$·dme (27.6 mg, 0.090 mmol, 30% mol) and 4,4′-di-tert-butyl-2,2′-dipyridyl (24.2 mg, 0.090 mmol, 30% mol) in dimethylacetamide (20 mL), an HPLC product area of 28% was obtained. After work-up, the crude was purified by preparative liquid chromatography (XSelect CSH C18, Agilent D50 454 Prep-MS, eluents: H_2O + 0.1% HCOOH, MeCN, flow rate = 60 mL/min) to afford 30.8 mg (27%) of the title compound.

^{1}H NMR (700 MHz, DMSO-d$_6$): δ 11.06 (s, 1H), 7.64 (d, J = 8.2 Hz, 1H), 6.80 (d, J = 2.2 Hz, 1H), 6.66 (dd, J = 8.4, 2.1 Hz, 1H), 5.05 (dd, J = 12.9, 5.5 Hz, 1H), 4.53 – 4.42 (m, 1H), 4.28 (t, J = 8.1 Hz, 2H), 3.82 (dd, J = 8.6, 5.7 Hz, 2H), 2.91 – 2.84 (m, 1H), 2.61 – 2.56 (m, 1H), 2.54 – 2.52 (m, 1H), 2.51 – 2.50 (m, 1H), 2.04 – 1.97 (m, 1H), 1.40 (s, 9H).

^{13}C NMR (176 MHz, DMSO-d$_6$): δ 172.8, 170.1, 167.4, 167.1, 154.9, 154.8, 133.8, 124.8, 117.1, 114.5, 104.7, 78.3, 58.5, 48.7, 39.9, 39.8, 39.6, 39.5, 39.4, 39.3, 39.2, 31.0, 28.2, 22.2.

HRMS (ESI-TOF) m/z: $[M + H]^+$ Calcd for $C_{21}H_{25}N_4O_6$ 429.1769; Found 429.1754.

Compound 1J

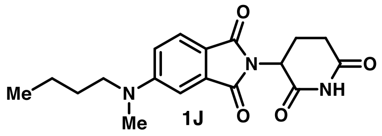

Following the General Procedure B with aryl bromide 1 (101 mg, 0.30 mmol, 1.0 equiv.), amine J (78.3 mg, 0.90 mmol, 3.0 equiv.), LiBr (78.2 mg, 0.90 mmol, 3.0 equiv.), $NiBr_2$·dme (27.6 mg, 0.090 mmol, 30% mol) and 4,4′-di-tert-butyl-2,2′-dipyridyl (24.2 mg, 0.090 mmol, 30% mol) in dimethylacetamide (20 mL), an HPLC product area of 28% was obtained. After work-up, the crude was purified by preparative liquid chromatography (XSelect CSH C18, Agilent D50 454 Prep-MS, eluents: H_2O + 0.1% HCOOH, MeCN, flow rate = 60 mL/min) to afford 23.6 mg (26%) of the title compound.

^{1}H NMR (700 MHz, DMSO-d$_6$): δ 11.06 (s, 1H), 7.63 (d, J = 8.5 Hz, 1H), 7.04 (d, J = 2.4 Hz, 1H), 6.96 (dd, J = 8.6, 2.4 Hz, 1H), 5.05 (dd, J = 12.9, 5.5 Hz, 1H), 3.53 – 3.47 (m, 2H), 3.06 (s, 3H), 2.92 – 2.84 (m, 1H), 2.61 – 2.56 (m, 1H), 2.54 – 2.51 (m, 1H), 2.03 – 1.97 (m, 1H), 1.56 – 1.49 (m, 2H), 1.36 – 1.28 (m, 2H), 0.91 (t, J = 7.4 Hz, 3H).

^{13}C NMR (176 MHz, DMSO-d$_6$): δ 172.8, 170.1, 167.7, 167.1, 153.5, 134.1, 125.0, 115.6, 115.0, 105.2, 51.5, 48.7, 39.9, 39.8, 39.6, 39.5, 39.4, 39.3, 39.2, 38.6, 31.0, 28.3, 22.2, 19.5, 13.8.

HRMS (ESI-TOF) m/z: $[M + H]^+$ Calcd for $C_{18}H_{22}N_3O_4$ 344.1605; Found 344.1597.

Compound 1K

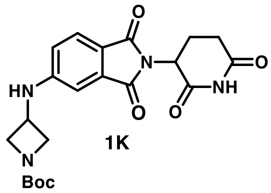

Following the General Procedure B with aryl bromide 1 (101 mg, 0.30 mmol, 1.0 equiv.), amine K (155 mg, 0.90 mmol, 3.0 equiv.), LiBr (78.2 mg, 0.90 mmol, 3.0 equiv.), $NiBr_2$·dme (27.6 mg, 0.090 mmol, 30% mol) and 4,4′-di-tert-butyl-2,2′-dipyridyl (24.2 mg, 0.090 mmol, 30% mol) in dimethylacetamide (20 mL), an HPLC product area of 17% was obtained. After work-up, the crude was purified by preparative liquid chromatography (XSelect CSH C18, Agilent D50 454 Prep-MS, eluents: H_2O + 0.1% TFA, MeCN, flow rate = 60 mL/min) to afford 12.9 mg (11%) of the title compound.

^{1}H NMR (700 MHz, DMSO-d$_6$): δ 11.06 (s, 1H), 7.64 (d, J = 6.4 Hz, 1H), 7.61 (d, J = 8.2 Hz, 1H), 6.89 (s, 1H), 6.81 (dd, J = 8.3, 2.1 Hz, 1H), 5.07 – 5.02 (m, 1H), 4.38 – 4.32 (m, 1H), 4.25 (s, 2H), 3.73 – 3.62 (m, 2H), 2.92 – 2.83 (m, 1H), 2.60 – 2.55 (m, 1H), 2.52 (d, J = 4.4 Hz, 1H), 2.03 – 1.97 (m, 1H), 1.39 (s, 9H).

^{13}C NMR (176 MHz, DMSO-d$_6$): δ 172.8, 170.1, 167.5, 167.1, 155.5, 152.8, 134.2, 125.2, 117.5, 78.8, 48.7, 41.8, 39.9, 39.8, 39.6, 39.5, 39.4, 39.3, 39.2, 31.0, 28.0, 22.2.

HRMS (ESI-TOF) m/z: $[M + H]^+$ Calcd for $C_{21}H_{25}N_4O_6$ 429.1769; Found 429.1750.

Compound 2A

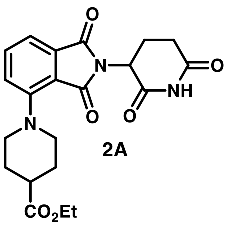

2A

Following the General Procedure A with aryl bromide 2 (50.6 mg, 0.15 mmol, 1.0 equiv.), amine A (70.7 mg, 0.45 mmol, 3.0 equiv.), LiBr (39.1 mg, 0.45 mmol, 3.0 equiv.), $NiBr_2$·dme (13.8 mg, 0.045 mmol, 30% mol) and 4,4′-di-tert-butyl-2,2′-dipyridyl (12.1 mg, 0.045 mmol, 30% mol) in dimethylacetamide (10 mL), an HPLC product area of 69% was obtained. After work-up, the crude was purified by supercritical fluid chromatography (ChiralPak IH, THAR SFC, eluents: CO_2 = 75%, MeOH = 25%, flow rate = 4.0 mL/min) to afford 30.6 mg (55%) of the title compound.

^{1}H NMR (700 MHz, DMSO-d$_6$): δ 11.08 (s, 1H), 7.68 (dd, J = 8.4, 7.1 Hz, 1H), 7.39 – 7.32 (m, 2H), 5.09 (dd, J = 12.9, 5.4 Hz, 1H), 4.10 (q, J = 7.1 Hz, 2H), 3.70 – 3.60 (m, 2H), 3.01 – 2.92 (m, 2H), 2.91 – 2.84 (m, 1H), 2.62 – 2.51 (m, 3H), 2.06 – 1.99 (m, 1H), 1.98 – 1.92 (m, 2H), 1.83 – 1.73 (m, 2H), 1.20 (t, J = 7.1 Hz, 3H).

^{13}C NMR (176 MHz, DMSO-d$_6$): δ 174.1, 172.8, 170.0, 167.1, 166.3, 149.9, 135.8, 133.6, 123.9, 116.6, 114.7, 59.9, 50.2, 50.1, 48.8, 31.0, 27.9, 22.0, 14.1.

HRMS (ESI-TOF) m/z: $[M + H]^+$ Calcd for $C_{21}H_{24}N_3O_6$ 414.1660; Found 414.1659.

Compound 2B

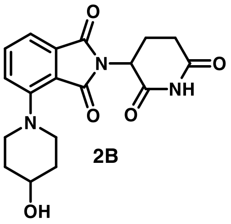

2B

Following the General Procedure A with aryl bromide 2 (50.6 mg, 0.15 mmol, 1.0 equiv.), amine B (45.5 mg, 0.45 mmol, 3.0 equiv.), LiBr (39.1 mg, 0.45 mmol, 3.0 equiv.), $NiBr_2$·dme (13.8 mg, 0.045 mmol, 30% mol) and 4,4′-di-tert-butyl-2,2′-dipyridyl (12.1 mg, 0.045 mmol, 30% mol) in dimethylacetamide (10 mL), an HPLC product area of 56% was obtained. After work-up, the crude was purified by supercritical fluid chromatography (Viridis Silica 2-Ethylpyridin 150-30, Agilent 1260, eluents: CO_2 = 75%, Methanol = 25%, flow rate = 4.0 mL/min) to afford 12.5 mg (39%) of the title compound.

^{1}H NMR (400 MHz, DMSO-d$_6$): δ 11.07 (s, 1H), 7.67 (t, J = 7.8 Hz, 1H), 7.36 – 7.26 (m, 2H), 5.09 (dd, J = 12.7, 5.4 Hz, 1H), 4.71 (d, J = 4.1 Hz, 1H), 3.72 – 3.64 (m, 1H), 3.57 – 3.46 (m, 2H), 3.03 (t, J = 10.8 Hz, 2H), 2.93 – 2.80 (m, 1H), 2.62 – 2.52 (m, 2H), 2.06 – 1.98 (m, 1H), 1.87 (d, J = 12.5 Hz, 2H), 1.62 – 1.52 (m, 2H).

^{13}C NMR (126 MHz, DMSO-d$_6$): δ 172.8, 170.0, 167.1, 166.3, 150.0, 135.7, 133.7, 123.9, 116.3, 114.4, 65.3, 48.7, 48.6, 34.2, 31.0, 22.0.

HRMS (ESI-TOF) m/z: $[M + H]^+$ Calcd for $C_{18}H_{20}N_3O_5$ 358.1397; Found 358.1389.

Compound 2C

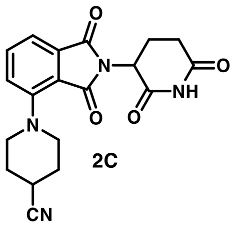

Following the General Procedure A with aryl bromide 2 (50.6 mg, 0.15 mmol, 1.0 equiv.), amine C (49.5 mg, 0.45 mmol, 3.0 equiv.), LiBr (39.1 mg, 0.45 mmol, 3.0 equiv.), $NiBr_2$·dme (13.8 mg, 0.045 mmol, 30% mol) and 4,4′-di-tert-butyl-2,2′-dipyridyl (12.1 mg, 0.045 mmol, 30% mol) in dimethylacetamide (10 mL), an HPLC product area of 75% was obtained. After work-up, the crude was purified by supercritical fluid chromatography (Viridis Silica 2-Ethylpyridin 150-30, Agilent 1260, eluents: CO_2 = 85%, Isopropanol = 15%, flow rate = 4.0 mL/min) to afford 31.8 mg (66%) of the title compound.

^{1}H NMR (700 MHz, DMSO-d$_6$): δ 11.08 (s, 1H), 7.70 (dd, J = 8.3, 7.2 Hz, 1H), 7.37 (dd, J = 7.9, 2.6 Hz, 2H), 5.10 (dd, J = 12.9, 5.5 Hz, 1H), 3.47 – 3.37 (m, 2H), 3.24 – 3.16 (m, 2H), 3.14 – 3.08 (m, 1H), 2.92 – 2.84 (m, 1H), 2.63 – 2.57 (m, 1H), 2.57 – 2.50 (m, 1H), 2.08 – 2.00 (m, 3H), 1.94 – 1.86 (m, 2H).

^{13}C NMR (176 MHz, DMSO-d$_6$): δ 172.8, 170.0, 167.0, 166.3, 149.7, 135.9, 133.6, 124.0, 122.1, 117.0, 115.1, 49.1, 49.1, 48.8, 30.9, 28.2, 24.9, 22.0.

HRMS (ESI-TOF) m/z: $[M + H]^+$ Calcd for $C_{19}H_{19}N_4O_4$ 367.1401; Found 367.1401.

Compound 2D

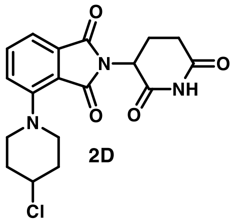

Following the General Procedure A with aryl bromide 2 (50.6 mg, 0.15 mmol, 1.0 equiv.), amine D (53.6 mg, 0.45 mmol, 3.0 equiv.), LiBr (39.1 mg, 0.45 mmol, 3.0 equiv.), $NiBr_2$·dme (13.8 mg, 0.045 mmol, 30% mol) and 4,4′-di-tert-butyl-2,2′-dipyridyl (12.1 mg, 0.045 mmol, 30% mol) in dimethylacetamide (10 mL), an HPLC product area of 72% was obtained. After work-up, the crude was purified by supercritical fluid chromatography (Viridis Silica 2-Ethylpyridin 150-30, Agilent 1260, eluents: CO_2 = 90%, Isopropanol = 10%, flow rate = 4.0 mL/min) to afford 32.9 mg (64%) of the title compound.

^{1}H NMR (400 MHz, DMSO-d$_6$): δ 11.05 (s, 1H), 7.70 (dd, J = 8.5, 7.1 Hz, 1H), 7.36 (dd, J = 7.8, 4.2 Hz, 2H), 5.09 (dd, J = 12.9, 5.4 Hz, 1H), 4.50 – 4.39 (m, 1H), 3.59 – 3.49 (m, 2H), 3.25 – 3.16 (m, 2H), 2.95 – 2.81 (m, 1H), 2.65 – 2.51 (m, 2H), 2.28 – 2.18 (m, 2H), 2.07 – 2.00 (m, 1H), 1.99 – 1.88 (m, 2H).

^{13}C NMR (126 MHz, DMSO-d$_6$): δ 172.8, 170.0, 167.0, 166.3, 149.5, 135.8, 133.6, 124.0, 116.8, 114.9, 57.5, 48.8, 48.7, 48.6, 35.1, 30.9, 22.0.

HRMS (ESI-TOF) m/z: $[M + H]^+$ Calcd for $C_{18}H_{19}N_3O_4Cl$ 376.1059; Found 376.1050.

Compound 2E

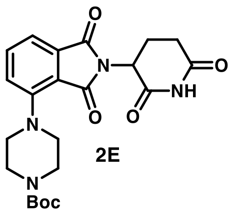

Following the General Procedure A with aryl bromide 2 (50.6 mg, 0.15 mmol, 1.0 equiv.), amine E (83.7 mg, 0.45 mmol, 3.0 equiv.), LiBr (39.1 mg, 0.45 mmol, 3.0 equiv.), $NiBr_2$·dme (13.8 mg, 0.045 mmol, 30% mol) and 4,4′-di-tert-butyl-2,2′-dipyridyl (12.1 mg, 0.045 mmol, 30% mol) in dimethylacetamide (10 mL), an HPLC product area of 65% was obtained. After work-up, the crude was purified by supercritical fluid chromatography (Viridis Silica 2-Ethylpyridin 150-30, THAR SFC, eluents: CO_2 = 85%, Isopropanol = 15%, flow rate = 4.0 mL/min) to afford 35.4 mg (56%) of the title compound.

^{1}H NMR (400 MHz, DMSO-d$_6$): δ 11.06 (s, 1H), 7.72 (dd, J = 8.4, 7.2 Hz, 1H), 7.37 (dd, J = 14.6, 7.7 Hz, 2H), 5.10 (dd, J = 12.9, 5.4 Hz, 1H), 3.56 – 3.47 (m, 4H), 3.28 – 3.22 (m, 4H), 2.95 – 2.81 (m, 1H), 2.64 – 2.51 (m, 2H), 2.07 – 1.98 (m, 1H), 1.43 (s, 9H).

^{13}C NMR (126 MHz, DMSO-d$_6$): δ 172.8, 169.9, 167.0, 166.3, 153.9, 149.6, 135.9, 133.6, 123.9, 117.0, 115.2, 79.1, 50.4, 48.8, 30.9, 28.0, 22.0.

HRMS (ESI-TOF) m/z: $[M + H]^+$ Calcd for $C_{22}H_{27}N_4O_6$ 443.1925; Found 443.1913.

Compound 2F

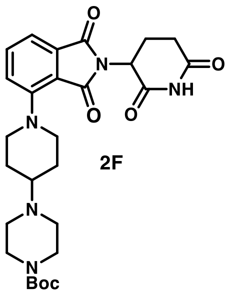

Following the General Procedure A with aryl bromide 2 (50.6 mg, 0.15 mmol, 1.0 equiv.), amine F (121 mg, 0.45 mmol, 3.0 equiv.), LiBr (39.1 mg, 0.45 mmol, 3.0 equiv.), $NiBr_2$·dme (13.8 mg, 0.045 mmol, 30% mol) and 4,4′-di-tert-butyl-2,2′-dipyridyl (12.1 mg, 0.045 mmol, 30% mol) in dimethylacetamide (10 mL), an HPLC product area of 39% was obtained. After work-up, the crude was purified by supercritical fluid chromatography (Viridis Silica 2-EP, Agilent 1260, eluents: CO_2 = 85%, Isopropanol = 15%, flow rate = 4.0 mL/min) to afford 34.6 mg (32%) of the title compound.

^{1}H NMR (700 MHz, DMSO-d$_6$): δ 11.12 (s, 1H), 7.71 (t, J = 7.7 Hz, 1H), 7.40 – 7.32 (m, 2H), 5.12 (dd, J = 12.9, 5.4 Hz, 1H), 3.77 (d, J = 11.8 Hz, 2H), 3.34 (s, 4H), 2.95 – 2.87 (m, 3H), 2.66 – 2.60 (m, 1H), 2.60 – 2.55 (m, 1H), 2.54 – 2.51 (m, 2H), 2.50 – 2.44 (m, 3H), 2.09 – 2.03 (m, 1H), 1.88 (d, J = 12.1 Hz, 2H), 1.70 – 1.61 (m, 2H), 1.43 (s, 9H).

^{13}C NMR (176 MHz, DMSO-d$_6$): δ 172.8, 170.0, 167.1, 166.3, 153.8, 149.8, 135.7, 133.6, 123.9, 116.4, 114.5, 78.7, 60.5, 50.4, 48.8, 48.5, 28.1, 27.8, 27.8, 22.0.

HRMS (ESI-TOF) m/z: $[M + H]^+$ Calcd for $C_{27}H_{36}N_5O_6$ 526.2660; Found 526.2663.

Compound 2G

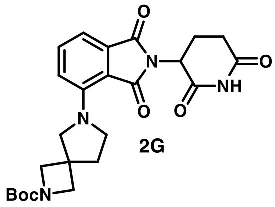

Following the General Procedure B with aryl bromide 2 (101 mg, 0.30 mmol, 1.0 equiv.), amine G (191 mg, 0.90 mmol, 3.0 equiv.), LiBr (78.2 mg, 0.90 mmol, 3.0 equiv.), $NiBr_2$·dme (27.6 mg, 0.090 mmol, 30% mol) and 4,4′-di-tert-butyl-2,2′-dipyridyl (24.2 mg, 0.090 mmol, 30% mol) in dimethylacetamide (20 mL), an HPLC product area of 20% was obtained. After work-up, the crude was purified by preparative liquid chromatography (XSelect CSH C18, Agilent D50 454 Prep-MS, eluents: H_2O + 0.1% HCOOH, MeCN, flow rate = 60 mL/min) to afford 33.3 mg (27%) of the title compound.

^{1}H NMR (700 MHz, DMSO-d$_6$): δ 11.05 (s, 1H), 7.58 (dd, J = 8.6, 6.9 Hz, 1H), 7.14 (d, J = 6.9 Hz, 1H), 7.08 (dd, J = 8.7, 0.8 Hz, 1H), 5.06 (dd, J = 12.8, 5.5 Hz, 1H), 3.89 – 3.76 (m, 4H), 3.73 (s, 2H), 3.57 (t, J = 6.9 Hz, 2H), 2.91 – 2.84 (m, 1H), 2.61 – 2.56 (m, 1H), 2.54 – 2.51 (m, 1H), 2.17 – 2.11 (m, 2H), 2.04 – 1.97 (m, 1H), 1.38 (s, 9H).

^{13}C NMR (176 MHz, DMSO-d$_6$): δ 172.8, 170.0, 167.1, 166.5, 155.5, 145.8, 134.9, 133.9, 121.1, 111.6, 110.4, 78.6, 60.1, 49.8, 48.7, 39.9, 39.8, 39.6, 39.5, 39.4, 39.3, 39.2, 35.0, 30.9, 28.0, 22.1.

HRMS (ESI-TOF) m/z: $[M + H]^+$ Calcd for $C_{24}H_{29}N_4O_6$ 469.2082; Found 469.2084.

Compound 2H

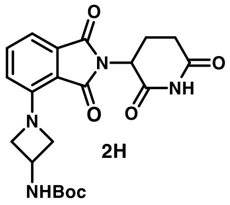

Following the General Procedure B with aryl bromide 2 (101 mg, 0.30 mmol, 1.0 equiv.), amine H (155 mg, 0.90 mmol, 3.0 equiv.), LiBr (78.2 mg, 0.90 mmol, 3.0 equiv.), $NiBr_2$·dme (27.6 mg, 0.090 mmol, 30% mol) and 4,4′-di-tert-butyl-2,2′-dipyridyl (24.2 mg, 0.090 mmol, 30% mol) in dimethylacetamide (20 mL), an HPLC product area of 24% was obtained. After work-up, the crude was purified by preparative liquid chromatography (XSelect CSH C18, Agilent D50 454 Prep-MS, eluents: H_2O + 0.1% TFA, MeCN, flow rate = 60 mL/min) to afford 15.3 mg (13%) of the title compound.

^{1}H NMR (700 MHz, DMSO-d$_6$): δ 11.06 (s, 1H), 7.60 – 7.54 (m, 2H), 7.12 (d, J = 6.9 Hz, 1H), 6.79 (d, J = 8.5 Hz, 1H), 5.05 (dd, J = 12.9, 5.5 Hz, 1H), 4.49 – 4.40 (m, 2H), 4.39 – 4.31 (m, 1H), 3.98 – 3.93 (m, 2H), 2.90 – 2.84 (m, 1H), 2.61 – 2.56 (m, 1H), 2.52 (d, J = 4.4 Hz, 1H), 2.03 – 1.97 (m, 1H), 1.39 (s, 9H).

^{13}C NMR (176 MHz, DMSO-d$_6$): δ 172.8, 170.0, 167.2, 166.5, 154.9, 147.7, 135.0, 133.3, 120.1, 111.8, 110.2, 78.2, 60.7, 48.6, 39.9, 39.8, 39.6, 39.5, 39.4, 39.3, 39.2, 30.9, 28.2, 22.1.

HRMS (ESI-TOF) m/z: $[M + H]^+$ Calcd for $C_{21}H_{25}N_4O_6$ 429.1769; Found 429.1767

Compound 2J

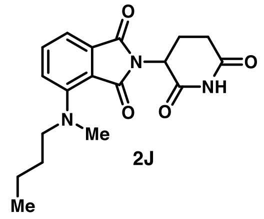

2J

Following the General Procedure B with aryl bromide 2 (101 mg, 0.30 mmol, 1.0 equiv.), amine J (78.3 mg, 0.90 mmol, 3.0 equiv.), LiBr (78.2 mg, 0.90 mmol, 3.0 equiv.), $NiBr_2$·dme (27.6 mg, 0.090 mmol, 30% mol) and 4,4′-di-tert-butyl-2,2′-dipyridyl (24.2 mg, 0.090 mmol, 30% mol) in dimethylacetamide (20 mL), an HPLC product area of 8% was obtained. After work-up, the crude was purified by preparative liquid chromatography (XSelect CSH C18, Agilent D50 454 Prep-MS, eluents: H_2O + 0.1% HCOOH, MeCN, flow rate = 60 mL/min) to afford 11.5 mg (12%) of the title compound.

^{1}H NMR (700 MHz, DMSO-d$_6$): δ 10.92 (s, 1H), 7.50 – 7.44 (m, 1H), 6.80 – 6.74 (m, 2H), 5.02 (dd, J = 13.3, 5.1 Hz, 1H), 4.30 (d, J = 16.6 Hz, 1H), 4.18 (d, J = 16.5 Hz, 1H), 3.43 – 3.37 (m, 2H), 2.92 – 2.86 (m, 1H), 2.62 – 2.56 (m, 2H), 2.38 – 2.30 (m, 1H), 1.98 – 1.91 (m, 1H), 1.55 – 1.47 (m, 2H), 1.35 – 1.27 (m, 2H), 0.91 (t, J = 7.4 Hz, 3H).

^{13}C NMR (176 MHz, DMSO-d$_6$): δ 173.0, 171.4, 168.7, 151.8, 144.3, 123.9, 118.5, 111.4, 104.9, 51.5, 51.3, 46.9, 39.9, 39.8, 39.6, 39.5, 39.4, 39.3, 39.2, 38.3, 31.3, 28.3, 22.6, 19.6, 13.9.

HRMS (ESI-TOF) m/z: $[M + H]^+$ Calcd for $C_{18}H_{22}N_3O_4$ 344.1605; Found 344.1598.

Compound 2K

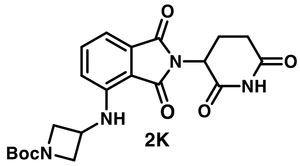

2K

Following the General Procedure B with aryl bromide 2 (101 mg, 0.30 mmol, 1.0 equiv.), amine K (155 mg, 0.90 mmol, 3.0 equiv.), LiBr (78.2 mg, 0.90 mmol, 3.0 equiv.), $NiBr_2$·dme (27.6 mg, 0.090 mmol, 30% mol) and 4,4′-di-tert-butyl-2,2′-dipyridyl (24.2 mg, 0.090 mmol, 30% mol) in dimethylacetamide (20 mL), an HPLC product area of 16% was obtained. After work-up, the crude was purified by preparative liquid chromatography (XSelect CSH C18, Agilent D50 454 Prep-MS, eluents: H_2O + 0.1% HCOOH, MeCN, flow rate = 60 mL/min) to afford 11.5 mg (25%) of the title compound.

^{1}H NMR (700 MHz, DMSO-d$_6$): δ 11.10 (s, 1H), 7.60 (dd, J = 8.5, 7.1 Hz, 1H), 7.13 (d, J = 7.1 Hz, 1H), 6.95 (d, J = 8.5 Hz, 1H), 6.88 (d, J = 6.5 Hz, 1H), 5.07 (dd, J = 12.9, 5.5 Hz, 1H), 4.48 – 4.42 (m, 1H), 4.21 (s, 2H), 3.81 (s, 2H), 2.93 – 2.85 (m, 1H), 2.63 – 2.56 (m, 1H), 2.53 – 2.51 (m, 1H), 2.06 – 2.00 (m, 1H), 1.39 (s, 9H).

^{13}C NMR (176 MHz, DMSO-d$_6$): δ 173.0, 171.4, 168.7, 151.8, 144.3, 123.9, 118.5, 111.4, 104.9, 51.5, 51.3, 46.9, 39.9, 39.8, 39.6, 39.5, 39.4, 39.3, 39.2, 38.3, 31.3, 28.3, 22.6, 19.6, 13.9.

HRMS (ESI-TOF) m/z: $[M + H]^+$ Calcd for $C_{21}H_{25}N_4O_6$ 429.1769; Found 429.1749.

Compound 3A

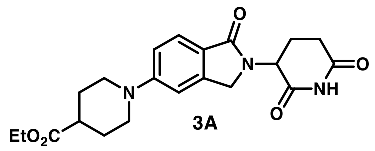

Following the General Procedure A with aryl bromide 3 (48.5 mg, 0.15 mmol, 1.0 equiv.), amine A (70.7 mg, 0.45 mmol, 3.0 equiv.), LiBr (39.1 mg, 0.45 mmol, 3.0 equiv.), $NiBr_2$·dme (13.8 mg, 0.045 mmol, 30% mol) and 4,4′-di-tert-butyl-2,2′-dipyridyl (12.1 mg, 0.045 mmol, 30% mol) in dimethylacetamide (10 mL), an HPLC product area of 45% was obtained. After work-up, the crude was purified by supercritical fluid chromatography (PharmPrep SI 100, THAR SFC, eluents: CO_2 = 75%, Isopropanol = 25%, flow rate = 5.0 mL/min) to afford 7.9 mg (19%) of the title compound.

^{1}H NMR (700 MHz, DMSO-d$_6$): δ 10.93 (s, 1H), 7.51 (d, J = 8.5 Hz, 1H), 7.11 – 7.00 (m, 2H), 5.04 (dd, J = 13.3, 5.1 Hz, 1H), 4.32 (d, J = 16.7 Hz, 1H), 4.20 (d, J = 16.7 Hz, 1H), 4.08 (q, J = 7.1 Hz, 2H), 3.85 – 3.78 (m, 2H), 2.98 – 2.92 (m, 2H), 2.92 – 2.85 (m, 1H), 2.62 – 2.55 (m, 2H), 2.40 – 2.32 (m, 1H), 1.99 – 1.93 (m, 1H), 1.92 – 1.86 (m, 2H), 1.70 – 1.58 (m, 2H), 1.19 (t, J = 7.1 Hz, 3H).

^{13}C NMR (176 MHz, DMSO-d$_6$): δ 173.9, 172.8, 170.1, 167.6, 166.9, 154.8, 134.0, 125.0, 117.8, 117.8, 108.0, 60.0, 48.7, 46.5, 31.0, 26.9, 25.5, 22.2, 14.1.

HRMS (ESI-TOF) m/z: $[M + H]^+$ Calcd for $C_{21}H_{26}N_3O_5$ 500.1867; Found 500.1867.

Compound 3C

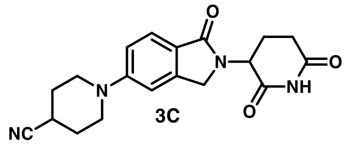

Following the General Procedure A with aryl bromide 3 (48.5 mg, 0.15 mmol, 1.0 equiv.), amine B (45.5 mg, 0.45 mmol, 3.0 equiv.), LiBr (39.1 mg, 0.45 mmol, 3.0 equiv.), $NiBr_2$·dme (13.8 mg, 0.045 mmol, 30% mol) and 4,4′-di-tert-butyl-2,2′-dipyridyl (12.1 mg, 0.045 mmol, 30% mol) in dimethylacetamide (10 mL), an HPLC product area of 45% was obtained. After work-up, the crude was purified by supercritical fluid chromatography (Viridis Silica 2-EP, Agilent 1260, eluents: CO_2 = 75%, Isopropanol = 25%, flow rate = 4.0 mL/min) to afford 7.9 mg (24%) of the title compound.

^{1}H NMR (700 MHz, DMSO-d$_6$): δ 10.94 (s, 1H), 7.52 (d, J = 8.5 Hz, 1H), 7.12 – 7.05 (m, 2H), 5.04 (dd, J = 13.3, 5.2 Hz, 1H), 4.33 (d, J = 16.6 Hz, 1H), 4.21 (d, J = 16.7 Hz, 1H), 3.57 – 3.50 (m, 2H), 3.25 – 3.19 (m, 2H), 3.14 – 3.07 (m, 1H), 2.94 – 2.86 (m, 1H), 2.61 – 2.55 (m, 1H), 2.41 – 2.32 (m, 1H), 2.01 – 1.93 (m, 3H), 1.84 – 1.76 (m, 2H).

^{13}C NMR (176 MHz, DMSO-d$_6$): δ 172.9, 171.3, 168.2, 153.3, 144.1, 123.8, 122.1, 121.6, 115.2, 108.9, 51.4, 47.0, 46.4, 31.2, 27.4, 25.2, 22.6.

HRMS (ESI-TOF) m/z: $[M + H]^+$ Calcd for $C_{19}H_{21}N_4O_3$ 353.1608; Found 353.1607.

Compound 3D

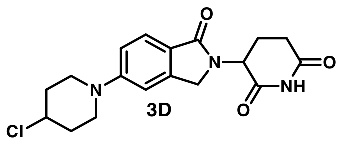

Following the General Procedure A with aryl bromide 3 (48.5 mg, 0.15 mmol, 1.0 equiv.), amine D (53.6 mg, 0.45 mmol, 3.0 equiv.), LiBr (39.1 mg, 0.45 mmol, 3.0 equiv.), $NiBr_2$·dme (13.8 mg, 0.045 mmol, 30% mol) and 4,4′-di-tert-butyl-2,2′-dipyridyl (12.1 mg, 0.045 mmol, 30% mol) in dimethylacetamide (10 mL), an HPLC product area of 43% was obtained. After work-up, the crude was purified by supercritical fluid chromatography (ChiralCel OD-H, THAR SFC, eluents: CO_2 = 60%, MeOH = 40%, flow rate = 5.0 mL/min) to afford 7.8 mg (23%) of the title compound.

^{1}H NMR (400 MHz, DMSO-d$_6$): δ 10.93 (s, 1H), 7.52 (d, J = 8.5 Hz, 1H), 7.16 – 7.01 (m, 2H), 5.11 – 4.98 (m, 1H), 4.49 – 4.38 (m, 1H), 4.33 (d, J = 16.7 Hz, 1H), 4.21 (d, J = 16.7 Hz, 1H), 3.71 – 3.59 (m, 2H), 3.25 – 3.15 (m, 2H), 2.95 – 2.83 (m, 1H), 2.60 (d, 1H), 2.40 – 2.31 (m, 1H), 2.19 – 2.08 (m, 2H), 2.00 – 1.91 (m, 1H), 1.88 – 1.75 (m, 2H).

^{13}C NMR (126 MHz, DMSO-d$_6$): δ 172.9, 171.3, 168.3, 153.0, 144.1, 123.8, 121.3, 114.9, 108.6, 57.9, 51.4, 46.9, 45.9, 34.3, 31.2, 22.6.

HRMS (ESI-TOF) m/z: $[M + H]^+$ Calcd for $C_{18}H_{21}N_3O_3Cl$ 362.1266; Found 362.1259.

Compound 3E

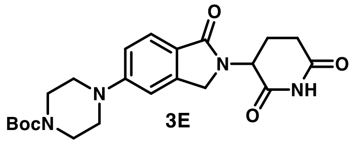

Following the General Procedure A with aryl bromide 3 (48.5 mg, 0.15 mmol, 1.0 equiv.), amine E (83.7 mg, 0.45 mmol, 3.0 equiv.), LiBr (39.1 mg, 0.45 mmol, 3.0 equiv.), $NiBr_2$·dme (13.8 mg, 0.045 mmol, 30% mol) and 4,4′-di-tert-butyl-2,2′-dipyridyl (12.1 mg, 0.045 mmol, 30% mol) in dimethylacetamide (10 mL), an HPLC product area of 50% was obtained. After work-up, the crude was purified by supercritical fluid chromatography (ChiralCel OD-H, THAR SFC, eluents: CO_2 = 60%, MeOH = 40%, flow rate = 5.0 mL/min) to afford 13.7 mg (37%) of the title compound.

^{1}H NMR (400 MHz, DMSO-d$_6$): δ 10.91 (s, 1H), 7.54 (d, J = 8.4 Hz, 1H), 7.10 – 7.03 (m, 2H), 5.04 (dd, J = 13.3, 5.1 Hz, 1H), 4.34 (d, J = 16.8 Hz, 1H), 4.22 (d, J = 16.9 Hz, 1H), 3.53 – 3.44 (m, 4H), 3.27 (d, J = 4.3 Hz, 4H), 2.97 – 2.83 (m, 1H), 2.63 – 2.54 (m, 1H), 2.45 – 2.29 (m, 1H), 2.01 – 1.92 (m, 1H), 1.43 (s, 9H).

^{13}C NMR (126 MHz, DMSO-d$_6$): δ 172.9, 171.2, 168.2, 153.9, 153.5, 144.0, 123.8, 121.9, 115.1, 108.8, 79.1, 51.4, 47.5, 47.0, 31.2, 28.1, 22.6.

HRMS (ESI-TOF) m/z: $[M + H]^+$ Calcd for $C_{22}H_{29}N_4O_5$ 429.2132; Found 429.2121.

Compound 3F

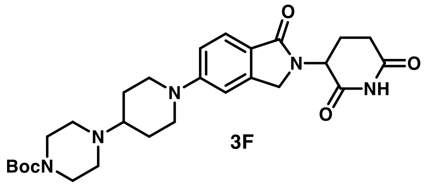

Following the General Procedure B with aryl bromide 3 (96.9 mg, 0.30 mmol, 1.0 equiv.), amine F (242 mg, 0.90 mmol, 3.0 equiv.), LiBr (78.2 mg, 0.90 mmol, 3.0 equiv.), $NiBr_2$·dme (27.6 mg, 0.090 mmol, 30% mol) and 4,4′-di-tert-butyl-2,2′-dipyridyl (24.2 mg, 0.090 mmol, 30% mol) in dimethylacetamide (20 mL), an HPLC product area of 40% was obtained. After work-up, the crude was purified by supercritical fluid chromatography (Viridis Silica 2-EP, Agilent 1260, eluents: CO_2 = 75%, Isopropanol = 25%, flow rate = 4.0 mL/min) to afford 4.9 mg (5%) of the title compound.

^{1}H NMR (700 MHz, DMSO-d$_6$): δ 11.00 (s, 1H), 7.56 (d, J = 8.5 Hz, 1H), 7.16 – 7.06 (m, 2H), 5.10 (dd, J = 13.3, 5.2 Hz, 1H), 4.37 (d, J = 16.7 Hz, 1H), 4.26 (d, J = 16.7 Hz, 1H), 4.01 – 3.93 (m, 2H), 3.36 – 3.33 (m, 4H), 3.00 – 2.92 (m, 1H), 2.91 – 2.84 (m, 2H), 2.68 – 2.62 (m, 1H), 2.50 – 2.48 (m, 3H), 2.45 – 2.38 (m, 1H), 2.05 – 1.98 (m, 1H), 1.91 – 1.84 (m, 2H), 1.56 – 1.50 (m, 2H), 1.45 (s, 9H), 1.10 (d, J = 6.1 Hz, 2H).

^{13}C NMR (176 MHz, DMSO-d$_6$): δ 172.9, 171.3, 168.3, 153.8, 153.4, 144.1, 123.8, 121.0, 114.8, 108.4, 78.7, 60.9, 51.4, 48.5, 47.4, 46.9, 39.9, 39.8, 39.6, 39.5, 39.4, 39.3, 39.2, 31.3, 31.1, 28.1, 27.2, 25.5, 22.6.

HRMS (ESI-TOF) m/z: $[M + H]^+$ Calcd for $C_{27}H_{38}N_5O_5$ 512.2867; Found 512.2865.

Compound 3H

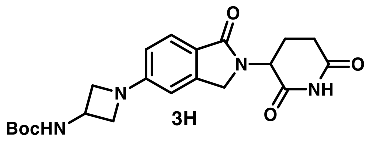

Following the General Procedure B with aryl bromide 3 (96.9 mg, 0.30 mmol, 1.0 equiv.), amine H (155 mg, 0.90 mmol, 3.0 equiv.), LiBr (78.2 mg, 0.90 mmol, 3.0 equiv.), $NiBr_2$·dme (27.6 mg, 0.090 mmol, 30% mol) and 4,4′-di-tert-butyl-2,2′-dipyridyl (24.2 mg, 0.090 mmol, 30% mol) in dimethylacetamide (20 mL), an HPLC product area of 16% was obtained. After work-up, the crude was purified by preparative liquid chromatography (XSelect CSH C18, Agilent D50 454 Prep-MS, eluents: H_2O + 0.1% TFA, MeCN, flow rate = 60 mL/min) to afford 14.3 mg (12%) of the title compound.

^{1}H NMR (700 MHz, DMSO-d$_6$): δ 10.93 (s, 1H), 7.59 (d, J = 7.5 Hz, 1H), 7.49 (d, J = 8.3 Hz, 1H), 6.53 (d, J = 2.1 Hz, 1H), 6.48 (dd, J = 8.3, 2.1 Hz, 1H), 5.03 (dd, J = 13.3, 5.1 Hz, 1H), 4.49 – 4.41 (m, 1H), 4.30 (d, J = 16.8 Hz, 1H), 4.21 – 4.12 (m, 3H), 3.71 – 3.66 (m, 2H), 2.94 – 2.84 (m, 1H), 2.61 – 2.56 (m, 1H), 2.39 – 2.29 (m, 1H), 1.98 – 1.92 (m, 1H), 1.39 (s, 9H).

^{13}C NMR (176 MHz, DMSO-d$_6$): δ 172.9, 171.3, 168.5, 154.8, 153.9, 144.0, 123.8, 120.2, 111.1, 104.7, 78.2, 58.6, 58.6, 51.4, 46.8, 39.9, 39.8, 39.6, 39.5, 39.4, 39.3, 39.2, 31.3, 30.2, 28.2, 22.6.

HRMS (ESI-TOF) m/z: $[M + H]^+$ Calcd for $C_{21}H_{27}N_4O_5$ 415.1976; Found 415.1975.

Compound 3J

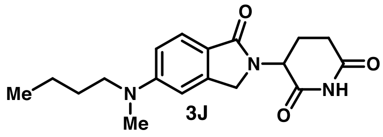

Following the General Procedure B with aryl bromide 3 (96.9 mg, 0.30 mmol, 1.0 equiv.), amine J (78.3 mg, 0.90 mmol, 3.0 equiv.), LiBr (78.2 mg, 0.90 mmol, 3.0 equiv.), $NiBr_2$·dme (27.6 mg, 0.090 mmol, 30% mol) and 4,4′-di-tert-butyl-2,2′-dipyridyl (24.2 mg, 0.090 mmol, 30% mol) in dimethylacetamide (20 mL), an HPLC product area of 19% was obtained. After work-up, the crude was purified by preparative liquid chromatography (XSelect CSH C18, Agilent D50 454 Prep-MS, eluents: H_2O + 0.1% HCOOH, MeCN, flow rate = 60 mL/min) to afford 8.8 mg (10%) of the title compound.

^{1}H NMR (700 MHz, DMSO-d$_6$): δ 11.06 (s, 1H), 7.61 (dd, J = 8.6, 7.0 Hz, 1H), 7.27 (d, J = 8.6 Hz, 1H), 7.21 (d, J = 6.9 Hz, 1H), 5.08 (dd, J = 12.8, 5.5 Hz, 1H), 3.49 – 3.43 (m, 2H), 3.32 – 3.27 (m, 1H), 3.00 (s, 3H), 2.91 – 2.85 (m, 1H), 2.61 – 2.57 (m, 1H), 2.53 – 2.50 (m, 2H), 2.04 – 2.00 (m, 1H), 1.58 – 1.54 (m, 2H), 1.27 – 1.21 (m, 2H), 0.86 (t, J = 7.4 Hz, 3H).

^{13}C NMR (176 MHz, DMSO-d$_6$): δ 172.8, 170.0, 167.1, 166.3, 149.3, 135.1, 134.0, 123.1, 113.1, 112.8, 54.3, 48.8, 40.1, 39.9, 39.8, 39.6, 39.5, 39.4, 39.3, 39.2, 30.9, 28.9, 22.1, 19.5, 13.8.

HRMS (ESI-TOF) m/z: $[M + H]^+$ Calcd for $C_{18}H_{24}N_3O_3$ 330.1812; Found 330.1805.

Compound 3K

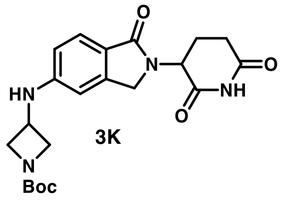

Following the General Procedure B with aryl bromide 3 (96.9 mg, 0.30 mmol, 1.0 equiv.), amine K (155 mg, 0.90 mmol, 3.0 equiv.), LiBr (78.2 mg, 0.90 mmol, 3.0 equiv.), $NiBr_2$·dme (27.6 mg, 0.090 mmol, 30% mol) and 4,4′-di-tert-butyl-2,2′-dipyridyl (24.2 mg, 0.090 mmol, 30% mol) in dimethylacetamide (20 mL), an HPLC product area of 17% was obtained. After work-up, the crude was purified by preparative liquid chromatography (XSelect CSH C18, Agilent D50 454 Prep-MS, eluents: H_2O + 0.1% HCOOH, MeCN, flow rate = 60 mL/min) to afford 5.2 mg (5%) of the title compound.

purification by preparative liquid chromatography (XSelect CSH C18, Agilent D50 454 Prep-MS, eluents: H_2O + 0.1% HCOOH, MeCN, flow rate = 60 mL/min).

^{1}H NMR (700 MHz, DMSO-d$_6$): δ 10.92 (s, 1H), 7.43 (d, J = 8.3 Hz, 1H), 6.96 (s, 1H), 6.62 (d, J = 8.4 Hz, 1H), 6.56 (s, 1H), 5.05 – 4.97 (m, 1H), 4.31 – 4.13 (m, 5H), 3.65 (s, 2H), 2.93 – 2.85 (m, 1H), 2.58 (d, J = 17.4 Hz, 1H), 2.38 – 2.29 (m, 1H), 1.97 – 1.90 (m, 1H), 1.38 (s, 9H).

^{13}C NMR (176 MHz, DMSO-d$_6$): δ 172.9, 171.3, 168.5, 155.5, 150.4, 144.5, 124.1, 120.1, 112.9, 105.0, 78.7, 51.3, 46.8, 42.0, 39.9, 39.8, 39.6, 39.5, 39.4, 39.3, 39.2, 31.3, 28.0, 22.6.

HRMS (ESI-TOF) m/z: $[M + H]^+$ Calcd for $C_{21}H_{27}N_4O_5$ 415.1976; Found 415.1970.

Compound 4A

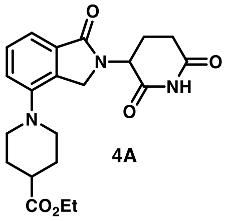

4A

Following the General Procedure A with aryl bromide 4 (48.5 mg, 0.15 mmol, 1.0 equiv.), amine A (70.7 mg, 0.45 mmol, 3.0 equiv.), LiBr (39.1 mg, 0.45 mmol, 3.0 equiv.), $NiBr_2$·dme (13.8 mg, 0.045 mmol, 30% mol) and 4,4′-di-tert-butyl-2,2′-dipyridyl (12.1 mg, 0.045 mmol, 30% mol) in dimethylacetamide (10 mL), an HPLC product area of 71% was obtained. After work-up, the crude was purified by supercritical fluid chromatography (PharmPrep SI 100, THAR SFC, eluents: CO_2 = 75%, Isopropanol = 25%, flow rate = 5.0 mL/min) to afford 25.3 mg (68%) of the title compound.

^{1}H NMR (700 MHz, DMSO-d$_6$): δ 10.97 (s, 1H), 7.43 (t, J = 7.7 Hz, 1H), 7.31 (dd, J = 7.5, 0.8 Hz, 1H), 7.16 (dd, J = 8.0, 0.9 Hz, 1H), 5.11 (dd, J = 13.4, 5.2 Hz, 1H), 4.43 (d, J = 17.2 Hz, 1H), 4.29 (d, J = 17.2 Hz, 1H), 4.09 (q, J = 7.1 Hz, 2H), 3.39 – 3.32 (m, 2H), 2.95 – 2.88 (m, 1H), 2.85 – 2.76 (m, 2H), 2.63 – 2.56 (m, 1H), 2.54 – 2.50 (m, 1H), 2.49 – 2.45 (m, 1H), 2.02 – 1.97 (m, 1H), 1.97 – 1.90 (m, 2H), 1.77 – 1.68 (m, 2H), 1.20 (t, J = 7.1 Hz, 3H).

^{13}C NMR (176 MHz, DMSO-d$_6$): δ 174.2, 172.9, 171.1, 168.2, 148.1, 133.8, 133.0, 129.2, 120.4, 116.1, 59.9, 51.5, 49.7, 49.7, 46.6, 40.0, 31.2, 28.3, 22.4, 14.1.

HRMS (ESI-TOF) m/z: $[M + H]^+$ Calcd for $C_{21}H_{26}N_3O_5$ 500.1867; Found 500.1865.

Compound 4B

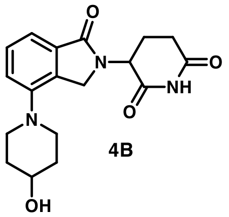

4B

Following the General Procedure B with aryl bromide 4 (96.9 mg, 0.30 mmol, 1.0 equiv.), amine B (90.9 mg, 0.90 mmol, 3.0 equiv.), LiBr (78.2 mg, 0.90 mmol, 3.0 equiv.), $NiBr_2$·dme (27.6 mg, 0.090 mmol, 30% mol) and 4,4′-di-tert-butyl-2,2′-dipyridyl (24.2 mg, 0.090 mmol, 30% mol) in dimethylacetamide (20 mL), an HPLC product area of 20% was obtained. After work-up, the crude was purified by preparative liquid chromatography (XSelect CSH C18, Agilent D50 454 Prep-MS, eluents: H_2O + 0.1% HCOOH, MeCN, flow rate = 60 mL/min) to afford 12.4 mg (14%) of the title compound.

^{1}H NMR (700 MHz, DMSO-d$_6$): δ 10.97 (s, 1H), 7.42 (t, J = 7.7 Hz, 1H), 7.29 (dd, J = 7.5, 0.8 Hz, 1H), 7.16 (dd, J = 8.0, 1.0 Hz, 1H), 5.11 (dd, J = 13.3, 5.2 Hz, 1H), 4.70 (d, J = 4.1 Hz, 1H), 4.43 (d, J = 17.1 Hz, 1H), 4.29 (d, J = 17.2 Hz, 1H), 3.67 – 3.61 (m, 1H), 3.30 – 3.24 (m, 2H), 2.95 – 2.87 (m, 1H), 2.87 – 2.78 (m, 2H), 2.62 – 2.56 (m, 1H), 2.50 – 2.45 (m, 1H), 2.02 – 1.96 (m, 1H), 1.88 – 1.82 (m, 2H), 1.58 – 1.50 (m, 2H).

^{13}C NMR (176 MHz, DMSO-d$_6$): δ 172.9, 171.1, 168.2, 148.1, 133.5, 132.9, 129.2, 120.3, 115.7, 65.7, 51.5, 48.1, 48.1, 46.7, 39.9, 39.8, 39.6, 39.5, 39.4, 39.3, 39.2, 34.6, 31.2, 22.4.

HRMS (ESI-TOF) m/z: $[M + H]^+$ Calcd for $C_{18}H_{22}N_3O_4$ 344.1605; Found 344.1598.

Compound 4C

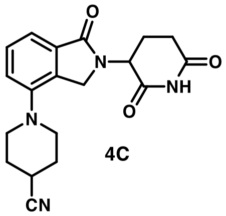
4C

Following the General Procedure A with aryl bromide 4 (48.5 mg, 0.15 mmol, 1.0 equiv.), amine C (49.5 mg, 0.45 mmol, 3.0 equiv.), LiBr (39.1 mg, 0.45 mmol, 3.0 equiv.), $NiBr_2$·dme (13.8 mg, 0.045 mmol, 30% mol) and 4,4′-di-tert-butyl-2,2′-dipyridyl (12.1 mg, 0.045 mmol, 30% mol) in dimethylacetamide (10 mL), an HPLC product area of 87% was obtained. After work-up, the crude was purified by supercritical fluid chromatography (PharmPrep SI 100, THAR SFC, eluents: CO_2 = 88%, Isopropanol = 12%, flow rate = 5.0 mL/min) to afford 13.5 mg (44%) of the title compound.

^{1}H NMR (700 MHz, DMSO-d_6): δ 10.98 (s, 1H), 7.44 (t, J = 7.7 Hz, 1H), 7.34 (d, J = 7.4 Hz, 1H), 7.19 (d, J = 8.0 Hz, 1H), 5.12 (dd, J = 13.3, 5.2 Hz, 1H), 4.44 (d, J = 17.2 Hz, 1H), 4.29 (d, J = 17.2 Hz, 1H), 3.24 – 3.14 (m, 2H), 3.10 – 3.04 (m, 1H), 3.03 – 2.89 (m, 3H), 2.65 – 2.57 (m, 1H), 2.49 – 2.43 (m, 1H), 2.05 – 1.96 (m, 3H), 1.90 – 1.82 (m, 2H).

^{13}C NMR (176 MHz, DMSO-d_6): δ 172.9, 171.0, 168.2, 147.8, 134.0, 133.0, 129.3, 122.2, 120.7, 116.4, 51.5, 48.7, 48.7, 46.5, 31.2, 28.6, 25.0, 22.4.

HRMS (ESI-TOF) m/z: $[M + H]^+$ Calcd for $C_{19}H_{21}N_4O_3$ 353.1608; Found 353.1606.

Compound 4D

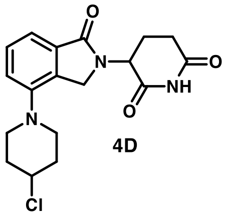
4D

Following the General Procedure A with aryl bromide 4 (48.5 mg, 0.15 mmol, 1.0 equiv.), amine D (53.6 mg, 0.45 mmol, 3.0 equiv.), LiBr (39.1 mg, 0.45 mmol, 3.0 equiv.), $NiBr_2$·dme (13.8 mg, 0.045 mmol, 30% mol) and 4,4′-di-tert-butyl-2,2′-dipyridyl (12.1 mg, 0.045 mmol, 30% mol) in dimethylacetamide (10 mL), an HPLC product area of 84% was obtained. After work-up, the crude was purified by supercritical fluid chromatography (ChiralCel OD-H, THAR SFC, eluents: CO_2 = 85%, MeOH = 15%, flow rate = 5.0 mL/min) to afford 17.7 mg (55%) of the title compound.

^{1}H NMR (400 MHz, DMSO-d_6): δ 10.98 (s, 1H), 7.44 (t, J = 7.7 Hz, 1H), 7.32 (dd, J = 7.5, 0.8 Hz, 1H), 7.19 (dd, J = 8.0, 0.9 Hz, 1H), 5.11 (dd, J = 13.3, 5.1 Hz, 1H), 4.45 (d, J = 17.3 Hz, 1H), 4.42 – 4.37 (m, 1H), 4.30 (d, J = 17.3 Hz, 1H), 3.29 – 3.24 (m, 1H), 3.04 – 2.88 (m, 3H), 2.64 – 2.56 (m, 1H), 2.49 – 2.46 (m, 2H), 2.22 – 2.16 (m, 2H), 2.04 – 1.96 (m, 1H), 1.96 – 1.86 (m, 2H).

^{13}C NMR (126 MHz, DMSO-d_6): δ 172.9, 171.1, 168.2, 147.7, 133.7, 133.0, 129.3, 120.6, 116.2, 57.6, 51.5, 48.3, 48.2, 46.6, 35.5, 31.2, 26.8, 22.4.

HRMS (ESI-TOF) m/z: $[M + H]^+$ Calcd for $C_{18}H_{21}N_3O_3Cl$ 362.1266; Found 362.1258.

Compound 4E

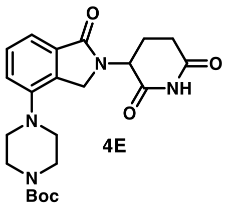

4E

Following the General Procedure A with aryl bromide 4 (48.5 mg, 0.15 mmol, 1.0 equiv.), amine E (83.7 mg, 0.45 mmol, 3.0 equiv.), LiBr (39.1 mg, 0.45 mmol, 3.0 equiv.), $NiBr_2$·dme (13.8 mg, 0.045 mmol, 30% mol) and 4,4′-di-tert-butyl-2,2′-dipyridyl (12.1 mg, 0.045 mmol, 30% mol) in dimethylacetamide (10 mL), an HPLC product area of 79% was obtained. After work-up, the crude was purified by supercritical fluid chromatography (ChiralPak IH, Agilent 1260, eluents: CO_2 = 85%, Isopropanol = 15%, flow rate = 4.0 mL/min) to afford 31.9 mg (55%) of the title compound.

^{1}H NMR (500 MHz, DMSO-d$_6$): δ 10.96 (s, 1H), 7.45 (t, J = 7.7 Hz, 1H), 7.34 (dd, J = 7.4, 0.9 Hz, 1H), 7.18 (dd, J = 8.0, 1.0 Hz, 1H), 5.12 (dd, J = 13.3, 5.1 Hz, 1H), 4.47 (d, J = 17.4 Hz, 1H), 4.32 (d, J = 17.4 Hz, 1H), 3.47 (t, J = 5.0 Hz, 4H), 3.10 – 2.96 (m, 4H), 2.96 – 2.86 (m, 1H), 2.65 – 2.56 (m, 1H), 2.49 – 2.39 (m, 1H), 2.05 – 1.95 (m, 1H), 1.43 (s, 9H).

^{13}C NMR (126 MHz, DMSO-d$_6$): δ 172.9, 171.0, 168.1, 153.8, 147.5, 133.6, 133.0, 129.3, 120.6, 116.5, 79.0, 51.5, 49.9, 46.6, 31.2, 28.0, 22.4.

HRMS (ESI-TOF) m/z: $[M + H]^+$ Calcd for $C_{22}H_{29}N_4O_5$ 429.2132; Found 429.2135.

Compound 4F

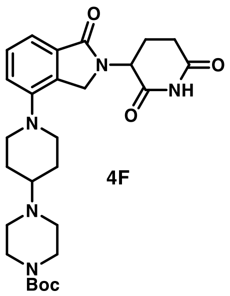

4F

Following the General Procedure B with aryl bromide 4 (96.9 mg, 0.30 mmol, 1.0 equiv.), amine F (242 mg, 0.90 mmol, 3.0 equiv.), LiBr (78.2 mg, 0.90 mmol, 3.0 equiv.), $NiBr_2$·dme (27.6 mg, 0.090 mmol, 30% mol) and 4,4′-di-tert-butyl-2,2′-dipyridyl (24.2 mg, 0.090 mmol, 30% mol) in dimethylacetamide (20 mL), an HPLC product area of 52% was obtained. After work-up, the crude was purified by preparative liquid chromatography (XSelect CSH C18, Agilent D50 454 Prep-MS, eluents: H_2O + 0.1% HCOOH, MeCN, flow rate = 60 mL/min) to afford 11.7 mg (13%) of the title compound.

^{1}H NMR (700 MHz, DMSO-d$_6$): δ 10.99 (s, 1H), 7.46 – 7.39 (m, 1H), 7.31 (d, J = 7.4 Hz, 1H), 7.16 (d, J = 7.9 Hz, 1H), 5.12 (dd, J = 13.4, 5.2 Hz, 1H), 4.44 (d, J = 17.1 Hz, 1H), 4.30 (d, J = 17.1 Hz, 1H), 3.52 – 3.36 (m, 3H), 2.96 – 2.89 (m, 1H), 2.80 – 2.68 (m, 2H), 2.60 (d, J = 17.3 Hz, 2H), 2.51 (s, 5H), 2.49 – 2.45 (m, 3H), 2.03 – 1.97 (m, 1H), 1.91 – 1.80 (m, 2H), 1.58 (s, 2H), 1.41 (s, 9H).

^{13}C NMR (176 MHz, DMSO-d$_6$): δ 172.9, 171.1, 168.2, 153.8, 147.9, 133.5, 132.9, 129.2, 120.3, 115.9, 78.7, 60.8, 51.5, 49.9, 49.8, 48.5, 46.7, 39.9, 39.8, 39.6, 39.5, 39.4, 39.3, 39.2, 31.2, 28.1, 22.4.

HRMS (ESI-TOF) m/z: $[M + H]^+$ Calcd for $C_{27}H_{38}N_5O_5$ 512.2867; Found 512.2851.

Compound 4G

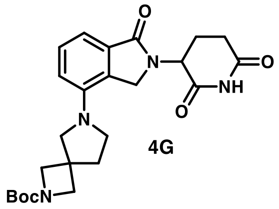

4G

Following the General Procedure B (DoE optimized conditions) with aryl bromide 4 (96.9 mg, 0.30 mmol, 1.0 equiv.), amine G (127 mg, 0.90 mmol, 2.0 equiv.), LiBr (78.2 mg, 0.90 mmol, 3.0 equiv.), $NiBr_2$·dme (36.8 mg, 0.090 mmol, 40% mol) and 4,4′-di-tert-butyl-2,2′-dipyridyl (32.3 mg, 0.090 mmol, 40% mol) in dimethylacetamide (20 mL), an HPLC product area of 40% was obtained. After work-up, the crude was purified by preparative liquid chromatography (XSelect CSH C18, Agilent D50 454 Prep-MS, eluents: H_2O + 0.1% HCOOH, MeCN, flow rate = 60 mL/min) to afford 31.1 mg (26%) of the title compound.

^{1}H NMR (700 MHz, DMSO-d$_6$): δ 10.98 (s, 1H), 7.29 (t, J = 7.7 Hz, 1H), 6.99 (dd, J = 7.3, 0.8 Hz, 1H), 6.67 (dd, J = 8.2, 0.9 Hz, 1H), 5.11 (dd, J = 13.3, 5.2 Hz, 1H), 4.70 (d, J = 16.7 Hz, 1H), 4.52 (d, J = 16.8 Hz, 1H), 3.82 (d, J = 51.1 Hz, 4H), 3.66 (d, J = 9.4 Hz, 1H), 3.62 (d, J = 9.4 Hz, 1H), 3.48 – 3.39 (m, 2H), 2.96 – 2.89 (m, 1H), 2.63 – 2.57 (m, 1H), 2.49 – 2.42 (m, 1H), 2.13 (t, J = 6.9 Hz, 2H), 2.01 – 1.95 (m, 1H), 1.38 (s, 9H).

^{13}C NMR (176 MHz, DMSO-d$_6$): δ 172.9, 171.1, 168.2, 153.8, 147.9, 133.5, 132.9, 129.2, 120.3, 115.9, 78.7, 60.8, 51.5, 49.9, 49.8, 48.5, 46.7, 39.9, 39.8, 39.6, 39.5, 39.4, 39.3, 39.2, 31.2, 28.1, 22.4.

HRMS (ESI-TOF) m/z: $[M + H]^+$ Calcd for $C_{24}H_{31}N_4O_5$ 455.2289; Found 455.2276.

Compound 4H

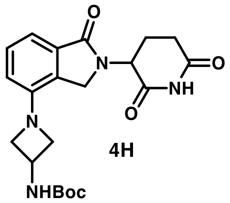

4H

Following the General Procedure B (DoE optimized conditions) with aryl bromide 4 (96.9 mg, 0.30 mmol, 1.0 equiv.), amine H (155 mg, 0.90 mmol, 2.0 equiv.), LiBr (78.2 mg, 0.90 mmol, 3.0 equiv.), $NiBr_2$·dme (36.8 mg, 0.090 mmol, 40% mol) and 4,4′-di-tert-butyl-2,2′-dipyridyl (32.3 mg, 0.090 mmol, 40% mol) in dimethylacetamide (20 mL), an HPLC product area of 16% was obtained. After work-up, the crude was purified by preparative liquid chromatography (XSelect CSH C18, Agilent D50 454 Prep-MS, eluents: H_2O + 0.1% TFA, MeCN, flow rate = 60 mL/min) to afford 9.1 mg (8%) of the title compound.

^{1}H NMR (700 MHz, DMSO-d$_6$): δ 10.97 (s, 1H), 7.53 (d, J = 7.5 Hz, 1H), 7.32 (t, J = 7.7 Hz, 1H), 7.07 (dd, J = 7.5, 0.8 Hz, 1H), 6.57 (d, J = 7.9 Hz, 1H), 5.08 (dd, J = 13.3, 5.2 Hz, 1H), 4.44 – 4.35 (m, 2H), 4.26 (d, J = 16.8 Hz, 1H), 4.23 – 4.16 (m, 2H), 3.75 (t, J = 6.7 Hz, 2H), 2.95 – 2.87 (m, 1H), 2.62 – 2.57 (m, 1H), 2.47 – 2.41 (m, 1H), 2.01 – 1.94 (m, 1H), 1.39 (s, 9H).

^{13}C NMR (176 MHz, DMSO-d$_6$): δ 173.4, 171.5, 168.7, 155.3, 146.9, 133.2, 129.4, 126.9, 115.0, 112.7, 78.7, 59.8, 59.7, 52.0, 46.5, 40.9, 40.4, 40.2, 40.1, 40.0, 39.9, 39.8, 39.7, 31.7, 28.7, 22.9.

HRMS (ESI-TOF) m/z: $[M + H]^+$ Calcd for $C_{21}H_{27}N_4O_5$ 415.1976; Found 415.1975.

Compound 4J

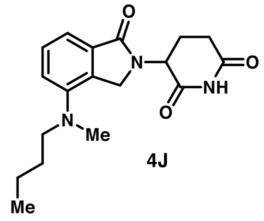

4J

Following the General Procedure B with aryl bromide 4 (96.9 mg, 0.30 mmol, 1.0 equiv.), amine J (78.3 mg, 0.90 mmol, 3.0 equiv.), LiBr (78.2 mg, 0.90 mmol, 3.0 equiv.), $NiBr_2$·dme (27.6 mg, 0.090 mmol, 30% mol) and 4,4′-di-tert-butyl-2,2′-dipyridyl (24.2 mg, 0.090 mmol, 30% mol) in dimethylacetamide (20 mL), an HPLC product area of 21% was obtained. After work-up, the crude was purified by preparative liquid chromatography (XSelect CSH C18, Agilent D50 454 Prep-MS, eluents: H_2O + 0.1% HCOOH, MeCN, flow rate = 60 mL/min) to afford 8.1 mg (9%) of the title compound.

^{1}H NMR (700 MHz, DMSO-d$_6$): δ 10.97 (s, 1H), 7.36 (t, J = 7.7 Hz, 1H), 7.15 (dd, J = 7.4, 0.8 Hz, 1H), 6.98 (dd, J = 8.2, 0.9 Hz, 1H), 5.10 (dd, J = 13.3, 5.2 Hz, 1H), 4.47 (d, J = 16.8 Hz, 1H), 4.35 (d, J = 16.7 Hz, 1H), 3.24 – 3.14 (m, 2H), 2.95 – 2.87 (m, 4H), 2.63 – 2.57 (m, 1H), 2.49 – 2.43 (m, 1H), 2.03 – 1.97 (m, 1H), 1.50 – 1.40 (m, 2H), 1.32 – 1.24 (m, 2H), 0.88 (t, J = 7.4 Hz, 3H).

^{13}C NMR (176 MHz, DMSO-d$_6$): δ 172.9, 171.1, 168.2, 146.8, 133.2, 129.7, 129.1, 118.4, 113.2, 53.1, 51.5, 47.7, 39.9, 39.8, 39.6, 39.5, 39.4, 39.3, 39.2, 31.2, 28.7, 22.4, 19.6, 13.9.

HRMS (ESI-TOF) m/z: $[M + H]^+$ Calcd for $C_{18}H_{24}N_3O_3$ 330.1812; Found 330.1804.

Compound 4K

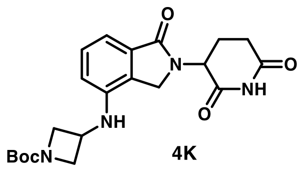

4K

Following the General Procedure B with aryl bromide 4 (96.9 mg, 0.30 mmol, 1.0 equiv.), amine K (155 mg, 0.90 mmol, 3.0 equiv.), LiBr (78.2 mg, 0.90 mmol, 3.0 equiv.), $NiBr_2$·dme (27.6 mg, 0.090 mmol, 30% mol) and 4,4′-di-tert-butyl-2,2′-dipyridyl (24.2 mg, 0.090 mmol, 30% mol) in dimethylacetamide (20 mL), an HPLC product area of 17% was obtained. After work-up, the crude was purified by preparative liquid chromatography (XSelect CSH C18, Agilent D50 454 Prep-MS, eluents: H_2O + 0.1% HCOOH, MeCN, flow rate = 60 mL/min) to afford 12.5 mg (12%) of the title compound.

^{1}H NMR (700 MHz, DMSO-d$_6$): δ 11.01 (s, 1H), 7.30 (t, J = 7.7 Hz, 1H), 7.02 (dd, J = 7.5, 0.8 Hz, 1H), 6.61 (d, J = 7.9 Hz, 1H), 6.22 (d, J = 6.7 Hz, 1H), 5.13 (dd, J = 13.3, 5.2 Hz, 1H), 4.31 – 4.15 (m, 5H), 3.74 – 3.68 (m, 2H), 2.96 – 2.89 (m, 1H), 2.65 – 2.59 (m, 1H), 2.35 – 2.26 (m, 1H), 2.07 – 2.00 (m, 1H), 1.38 (s, 9H).

^{13}C NMR (176 MHz, DMSO-d$_6$): δ 172.9, 171.1, 168.2, 146.8, 133.2, 129.7, 129.1, 118.4, 113.2, 53.1, 51.5, 47.7, 39.9, 39.8, 39.6, 39.5, 39.4, 39.3, 39.2, 31.2, 28.7, 22.4, 19.6, 13.9.

HRMS (ESI-TOF) m/z: $[M + H]^+$ Calcd for $C_{21}H_{27}N_4O_5$ 415.1976; Found 415.1983.

10. References

[1] C. Rosso, S. Gisbertz, J. D. Williams, H. Gemoets, W. Debrouwer, B. Pieber, C. O. Kappe, *React. Chem. Eng.* **2020**, 5, 597–604.

[2] W. Debrouwer, W. Kimpe, R. Dangreau, K. Huvaere, H. P. L. Gemoets, M. Mottaghi, S. Kuhn, K. Van Aken, *Org. Process Res. Dev*. **2020**, 24, 2319–2325.

[3] W. Jud, C. O. Kappe and D. Cantillo, *Chemistry–Methods*, **2021**, 1, 36-41.

[4] C. Li, Y. Kawamata, H. Nakamura, J. C. Vantourout, Z. Liu, Q. Hou, D. Bao, J. T. Starr, J. Chen, M. Yan, P. S. Baran, *Angew. Chem. Int. Ed.* **2017**, *56*, 13088.

11. NMR Spectra

Compound **1A**: ^{1}H NMR (400 MHz, DMSO-d$_6$)

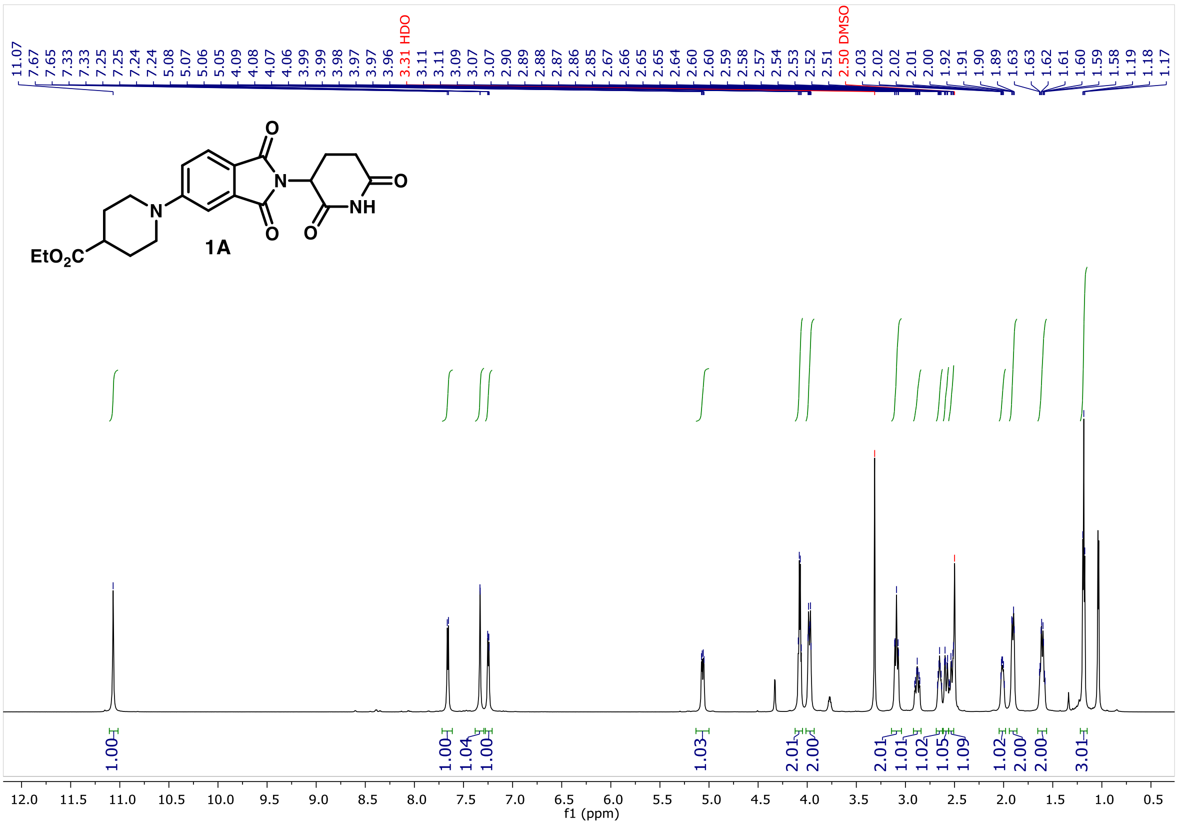

Compound **1A**: ^{13}C NMR (126 MHz, DMSO-d$_6$)

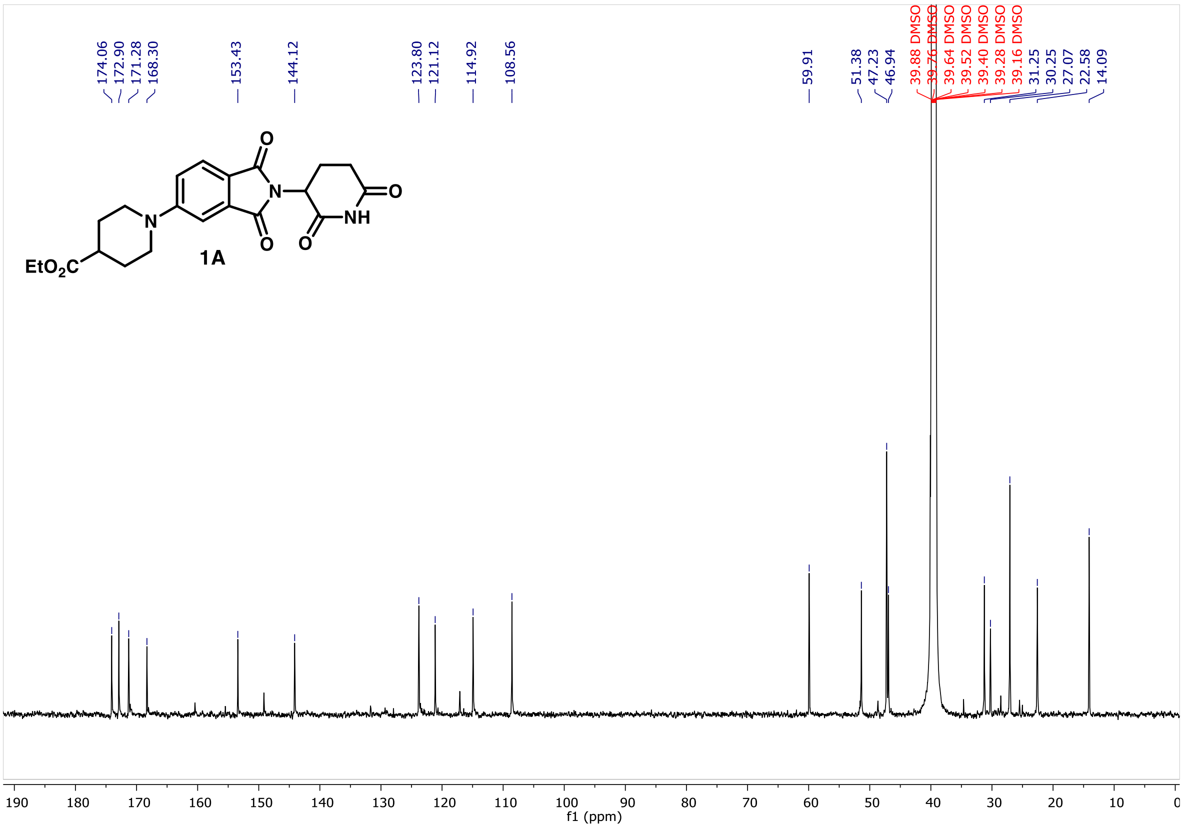

Compound **1B**: ^{1}H NMR (400 MHz, DMSO-d$_6$)

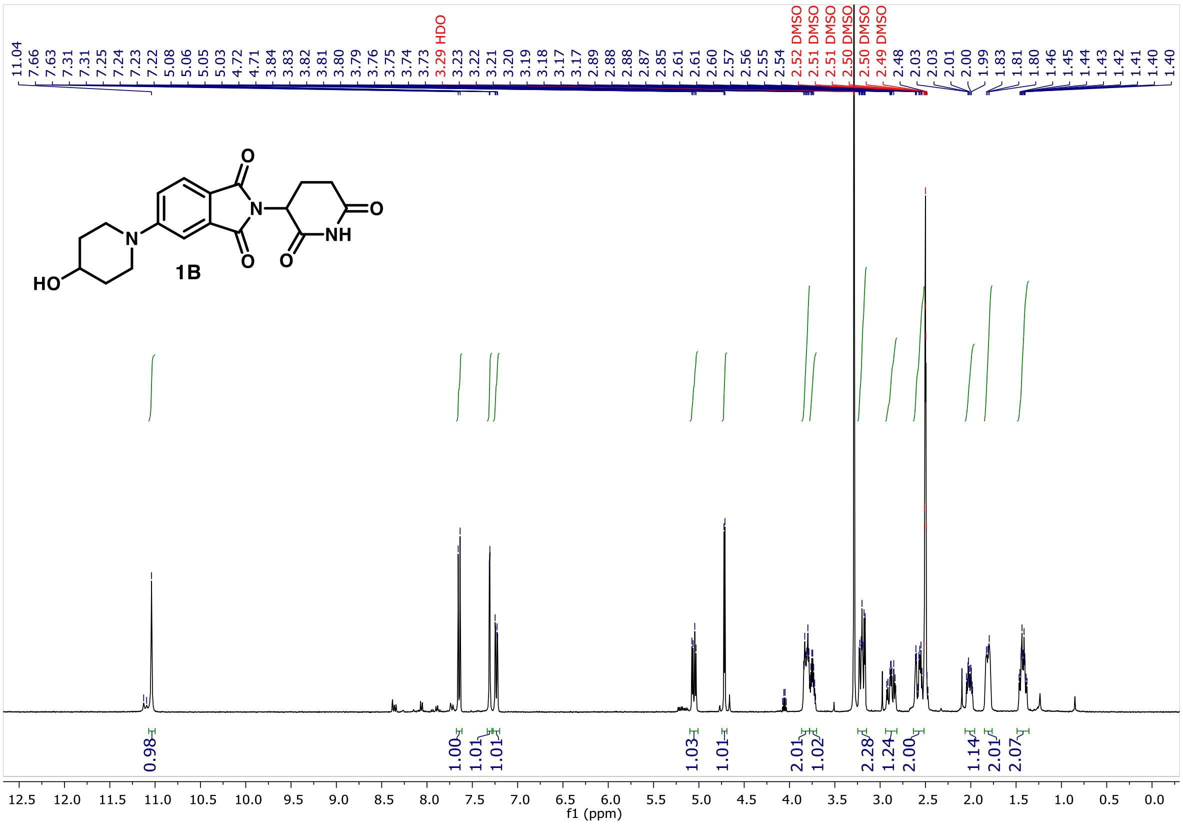

Compound **1B**: ^{13}C NMR (126 MHz, DMSO-d$_6$)

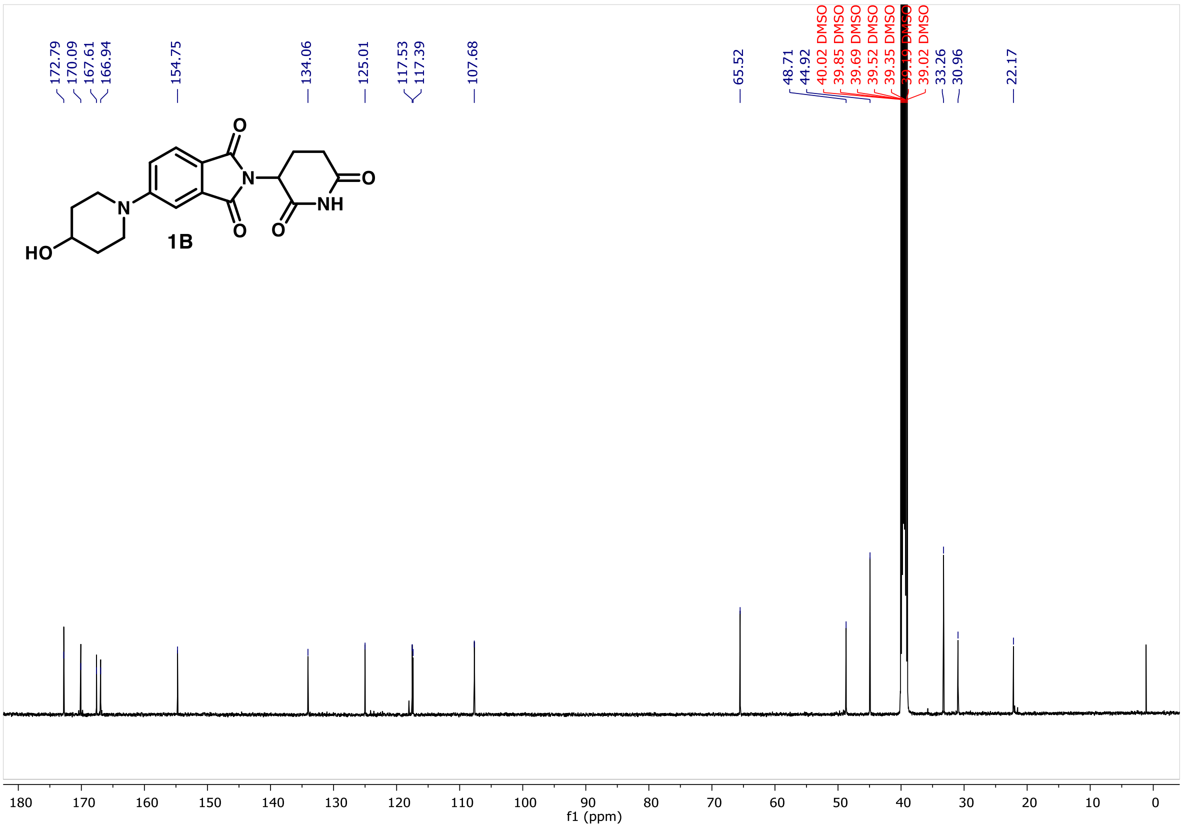

Compound **1C**: ^{1}H NMR (400 MHz, DMSO-d$_6$)

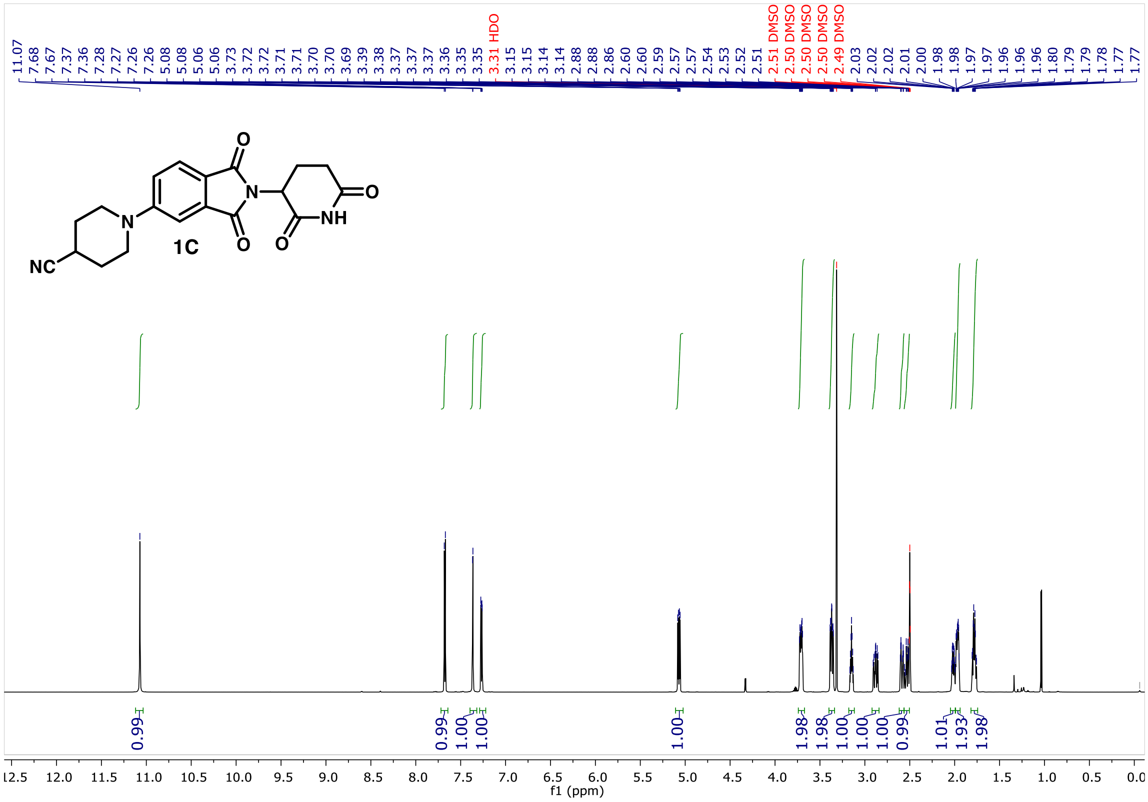

Compound **1C**: ^{13}C NMR (126 MHz, DMSO-d$_6$)

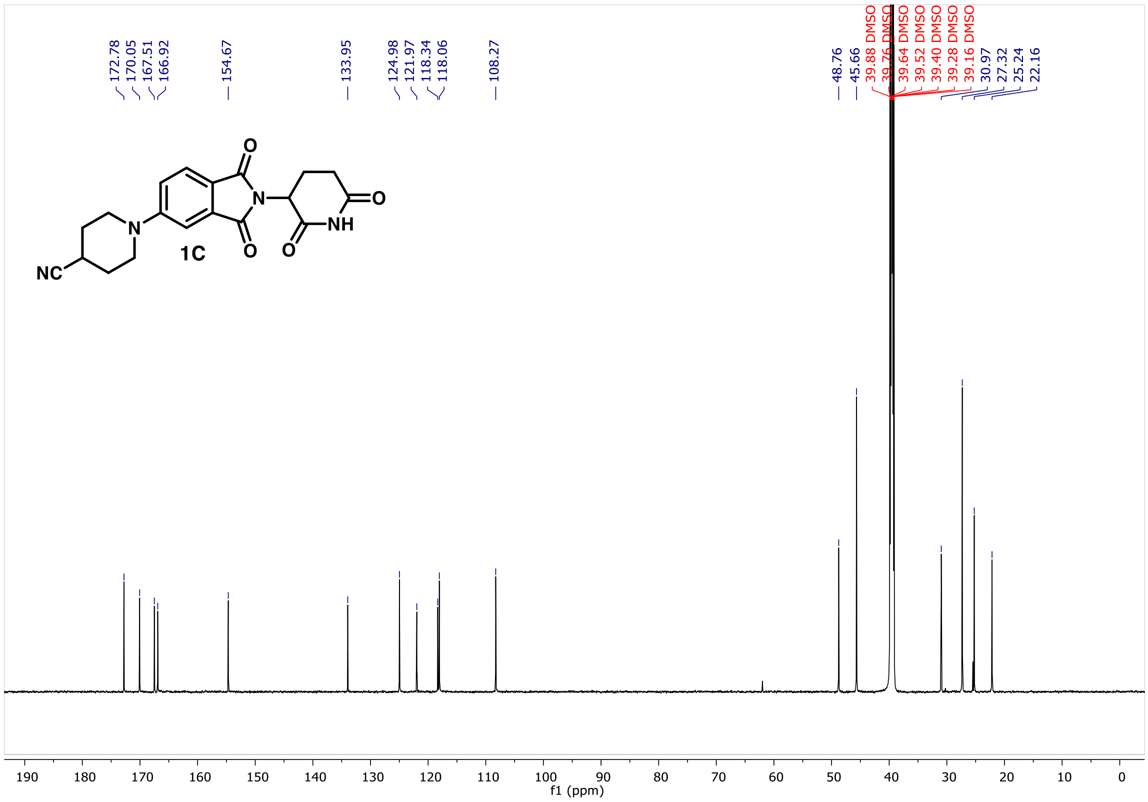

Compound **1D**: ^{1}H NMR (400 MHz, DMSO-d_6)

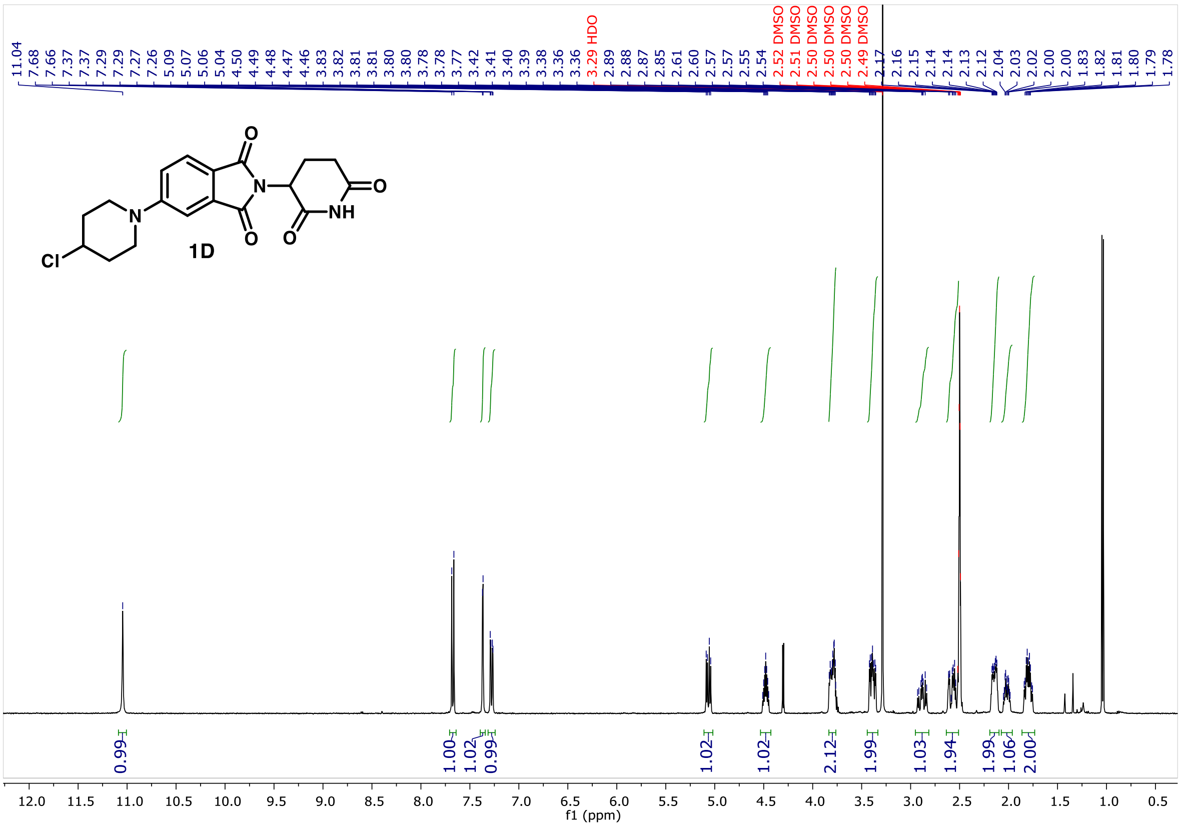

Compound **1D**: ^{13}C NMR (126 MHz, DMSO-d_6)

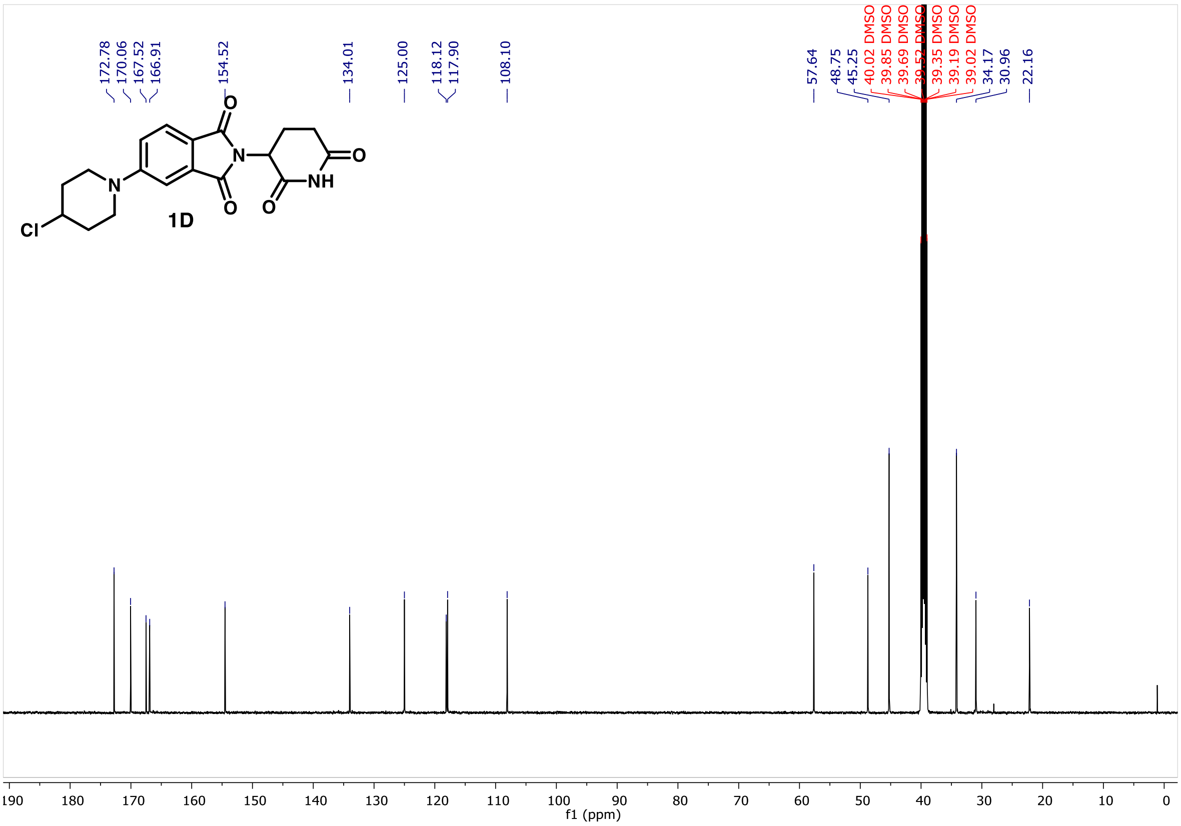

Compound **1E**: ^{1}H NMR (400 MHz, DMSO-d$_6$)

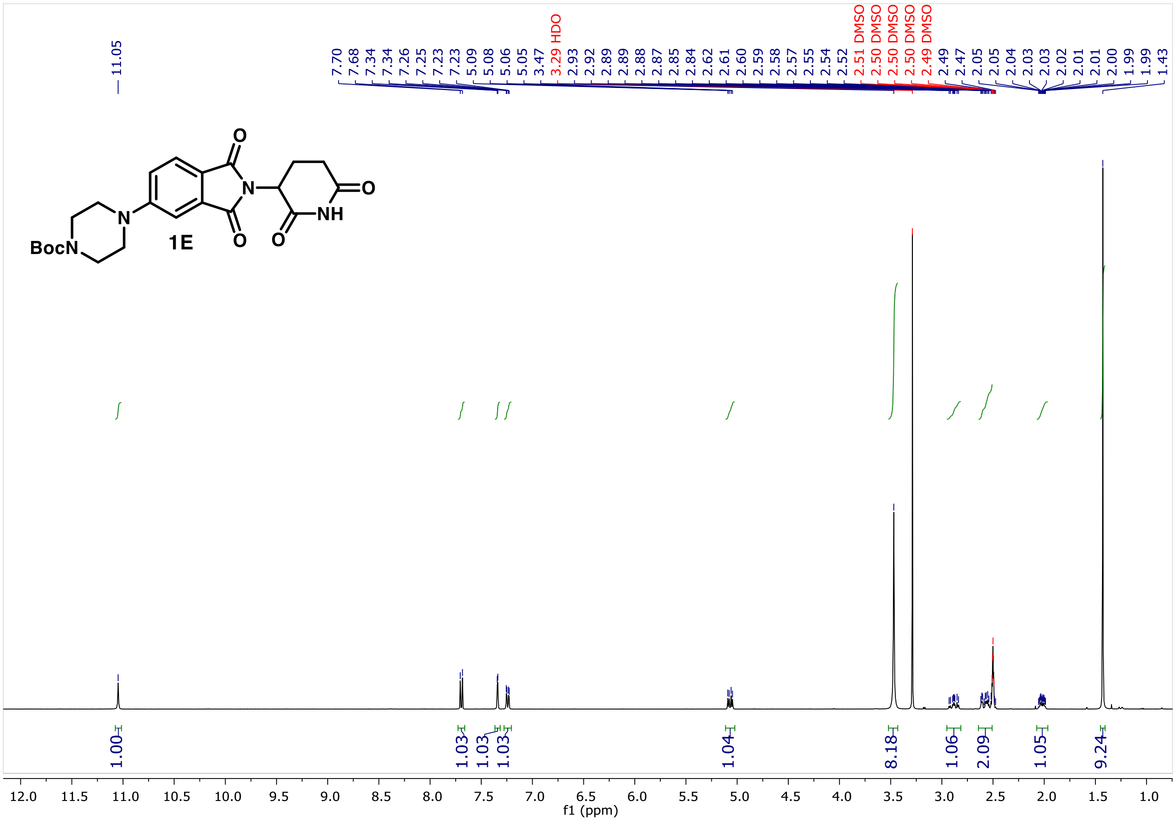

Compound **1E**: ^{13}C NMR (126 MHz, DMSO-d$_6$)

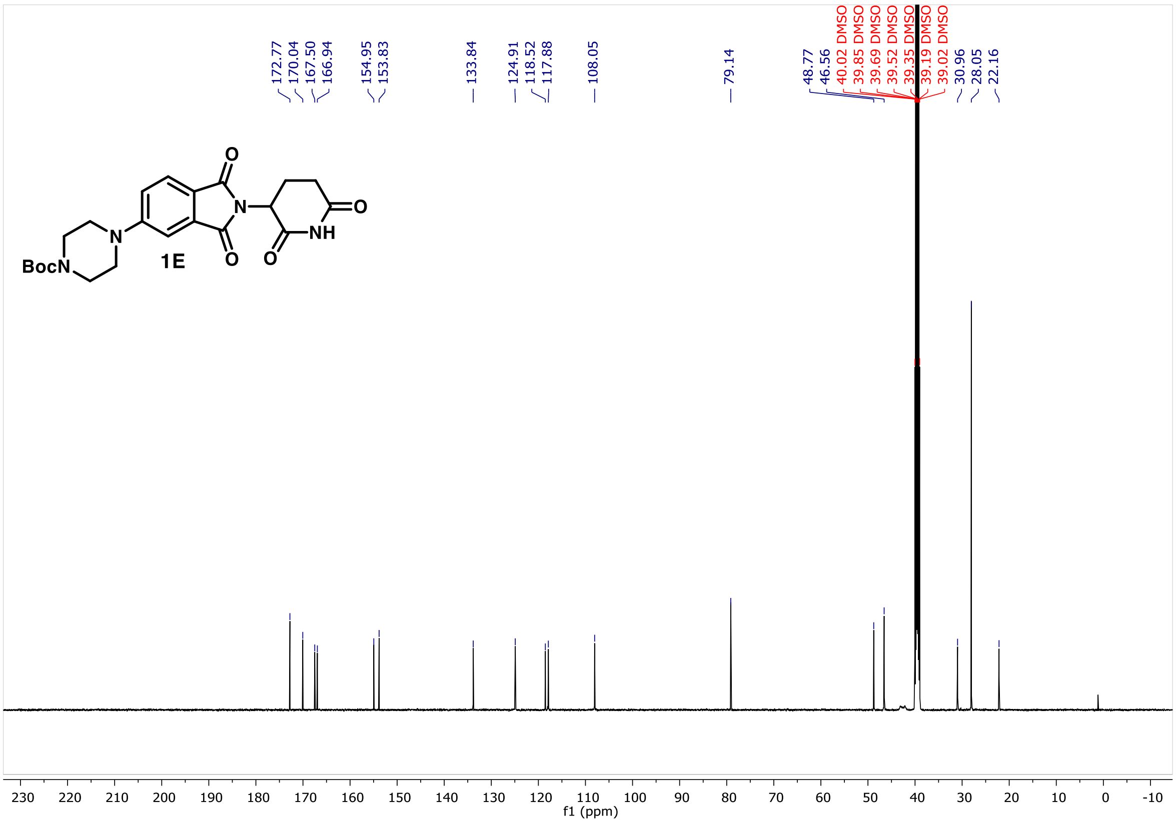

Compound **1F**: ^{1}H NMR (400 MHz, DMSO-d_6)

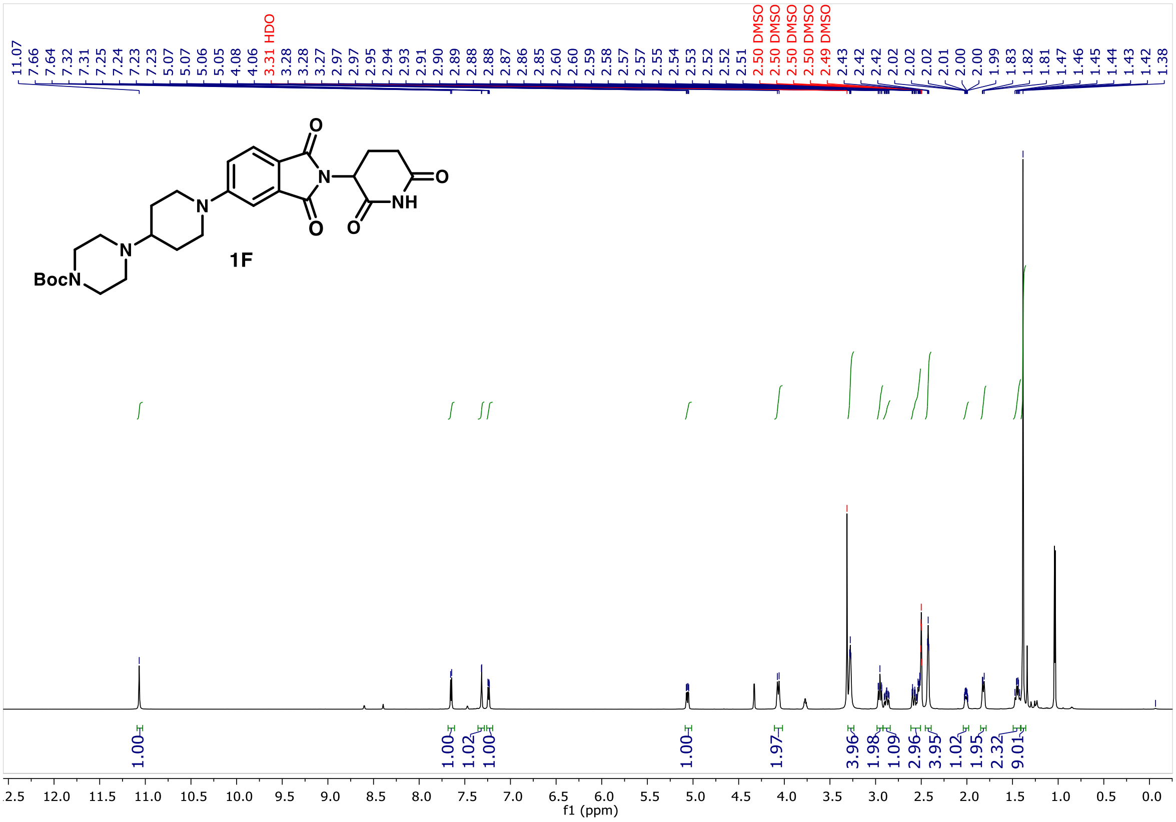

Compound **1F**: ^{13}C NMR (126 MHz, DMSO-d_6)

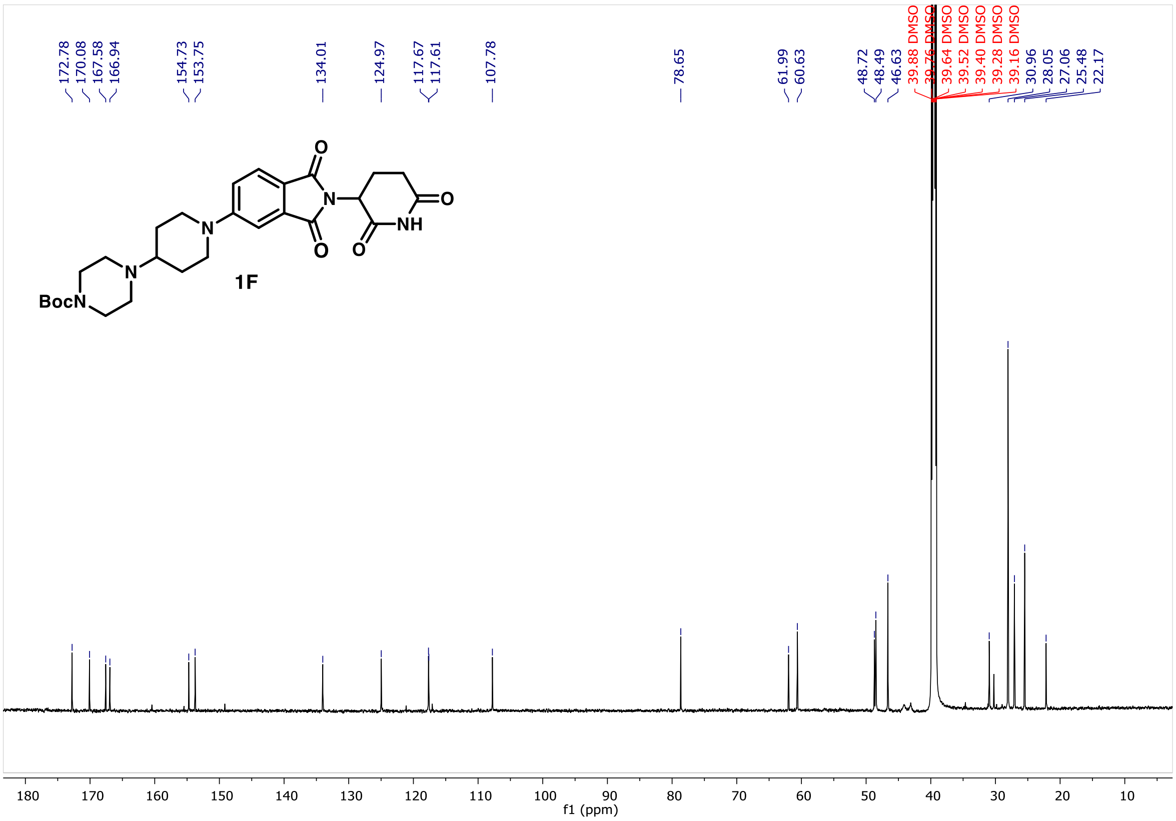

Compound **1G**: ^{1}H NMR (700 MHz, DMSO-d$_6$)

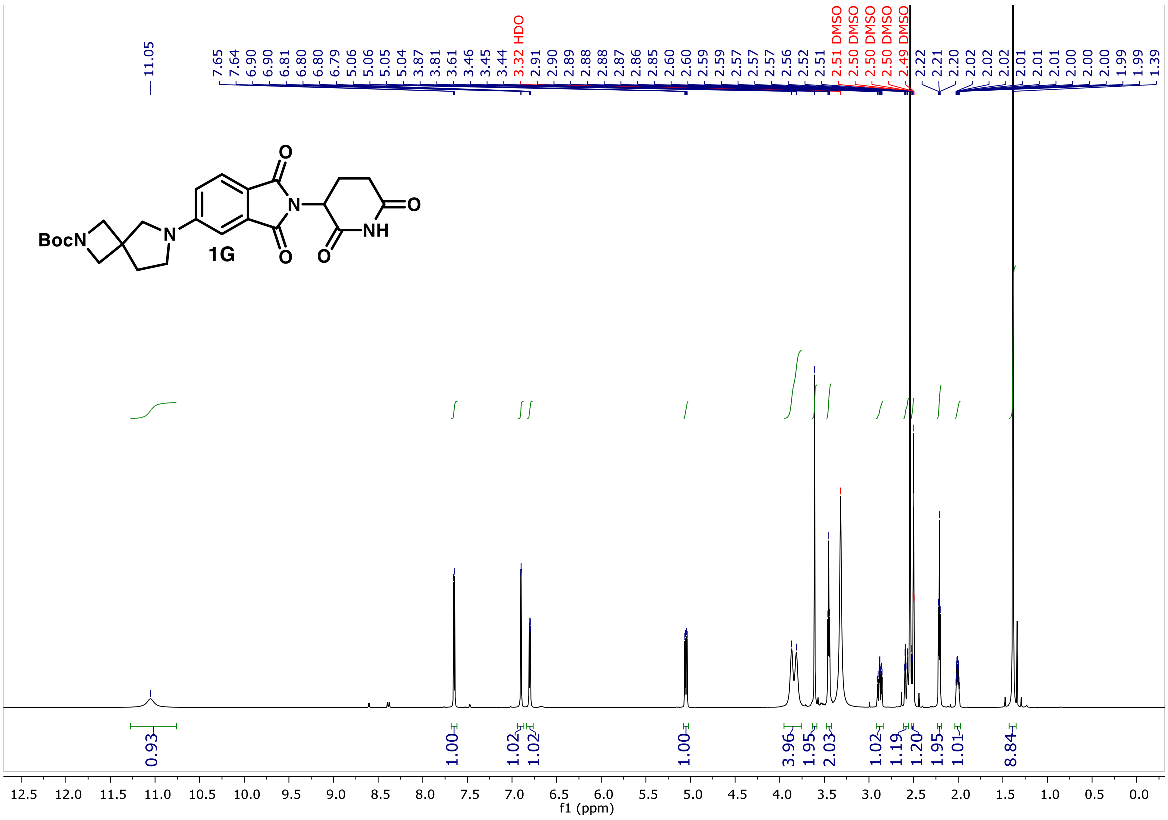

Compound **1G**: ^{13}C NMR (176 MHz, DMSO-d$_6$)

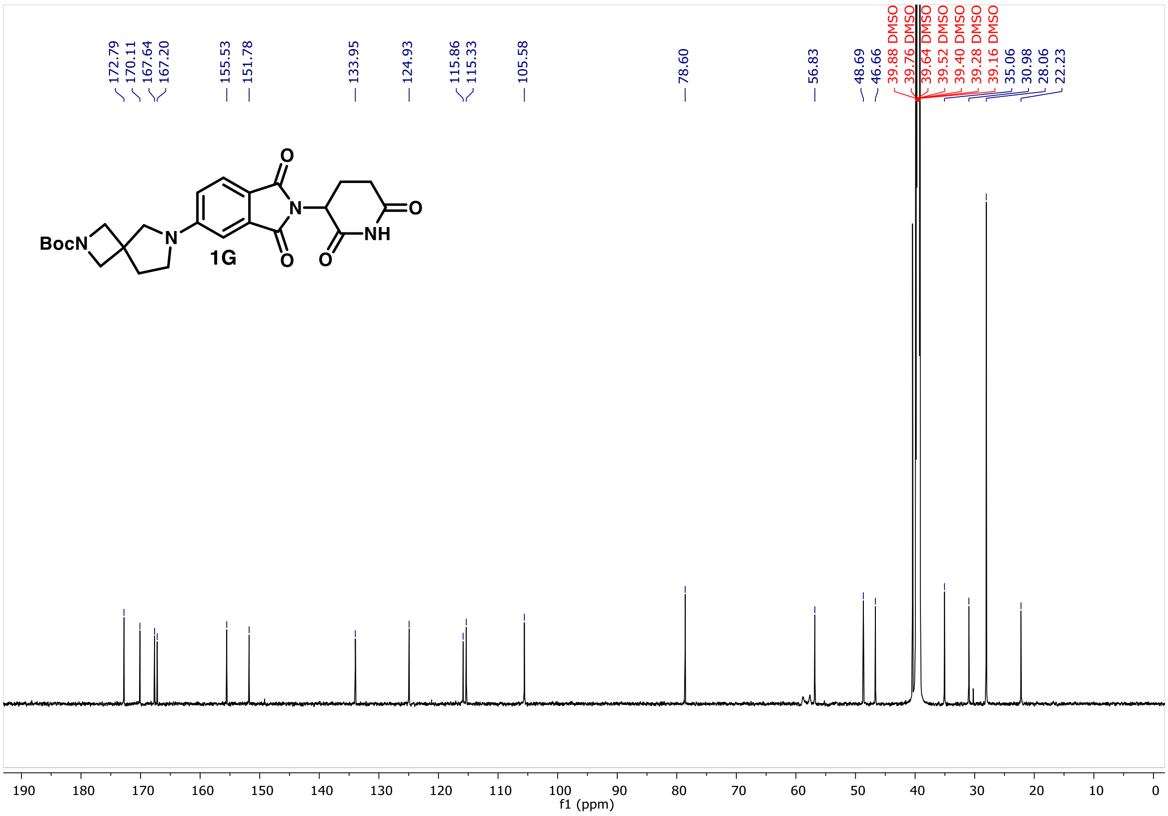

Compound **1H**: ^{1}H NMR (700 MHz, DMSO-d_6)

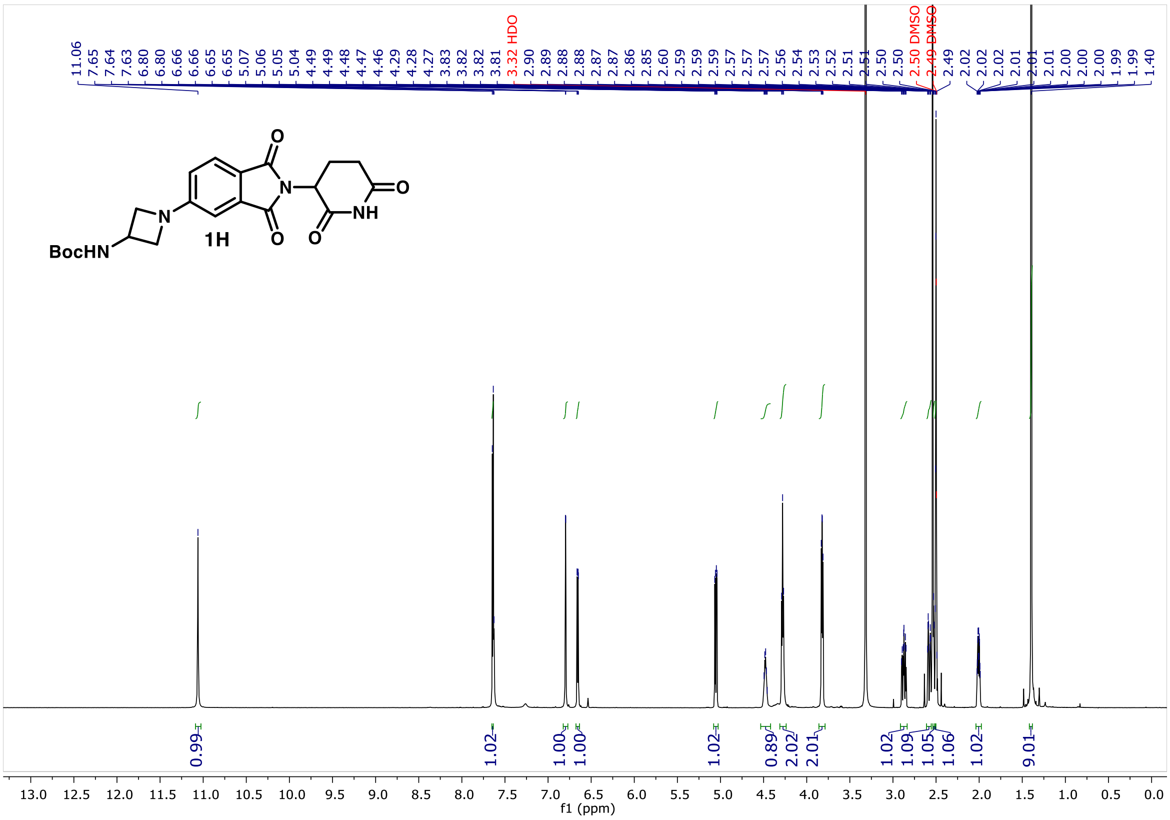

Compound **1H**: ^{13}C NMR (176 MHz, DMSO-d_6)

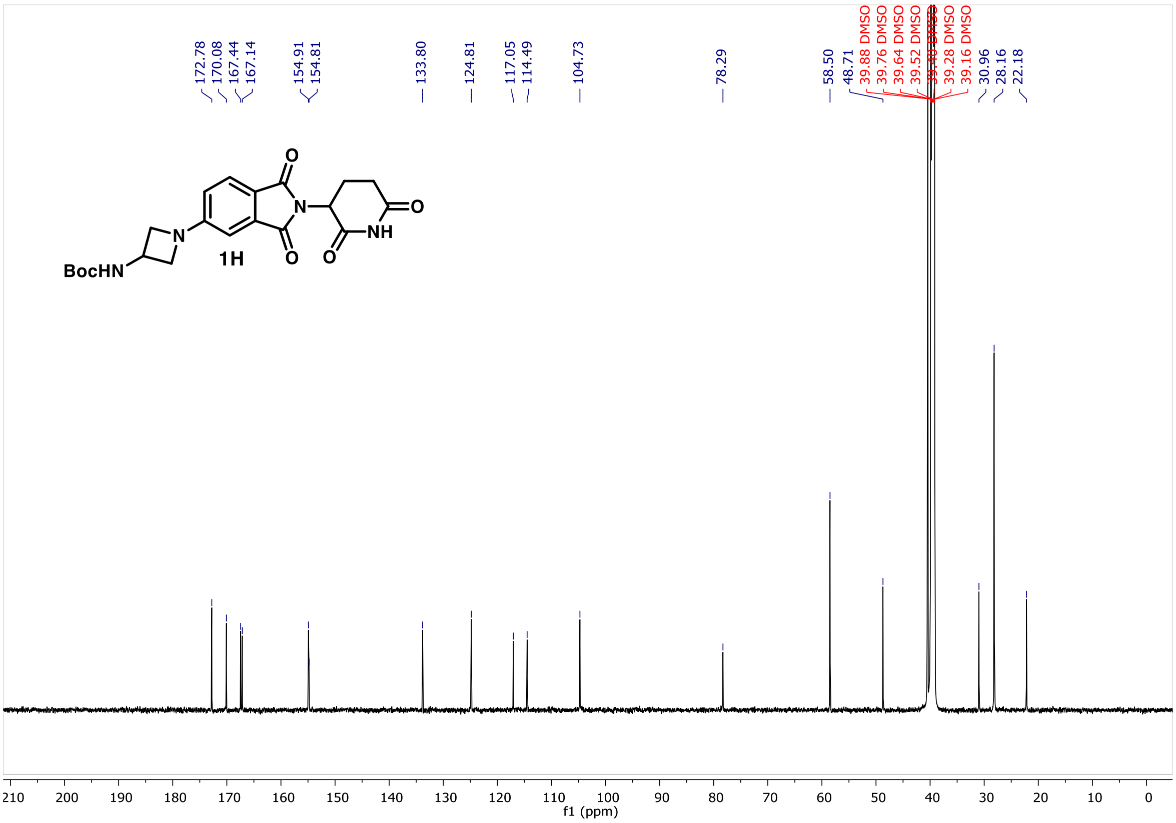

Compound **1J**: ^{1}H NMR (700 MHz, DMSO-d_6)

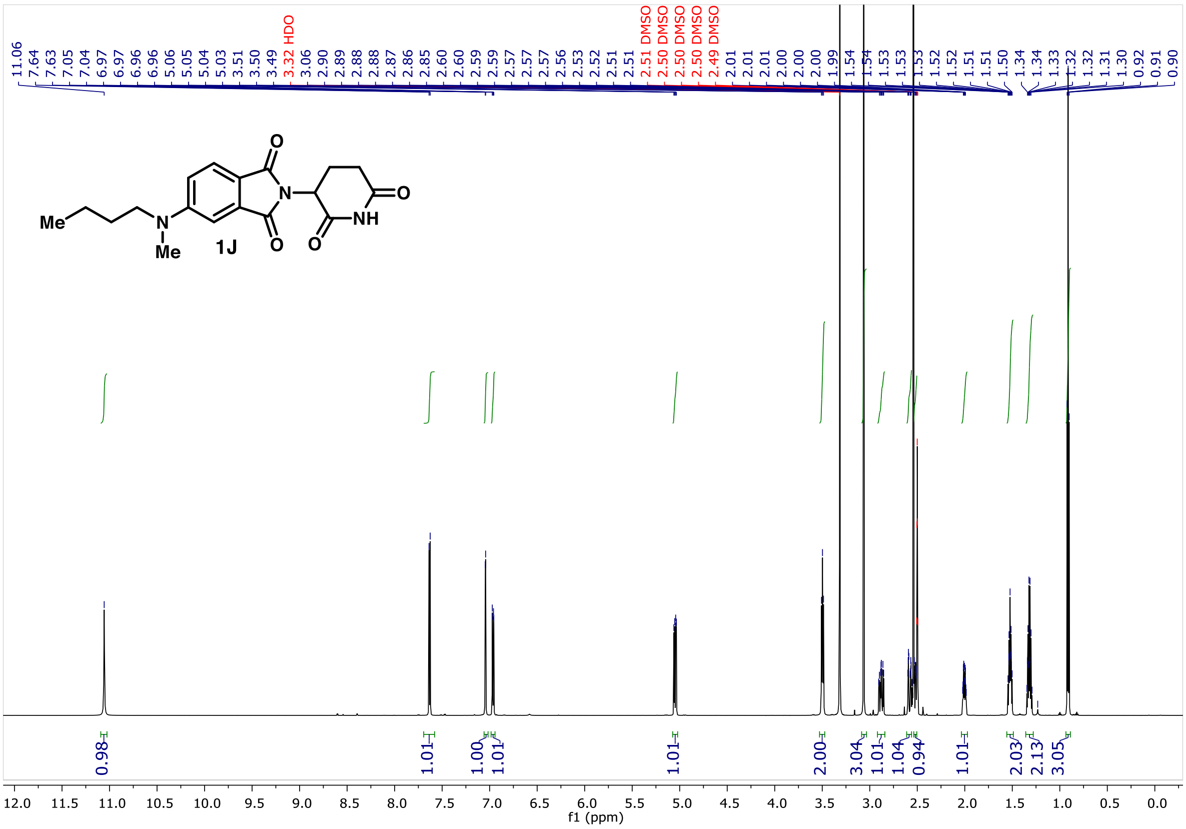

Compound **1J**: ^{13}C NMR (176 MHz, DMSO-d_6)

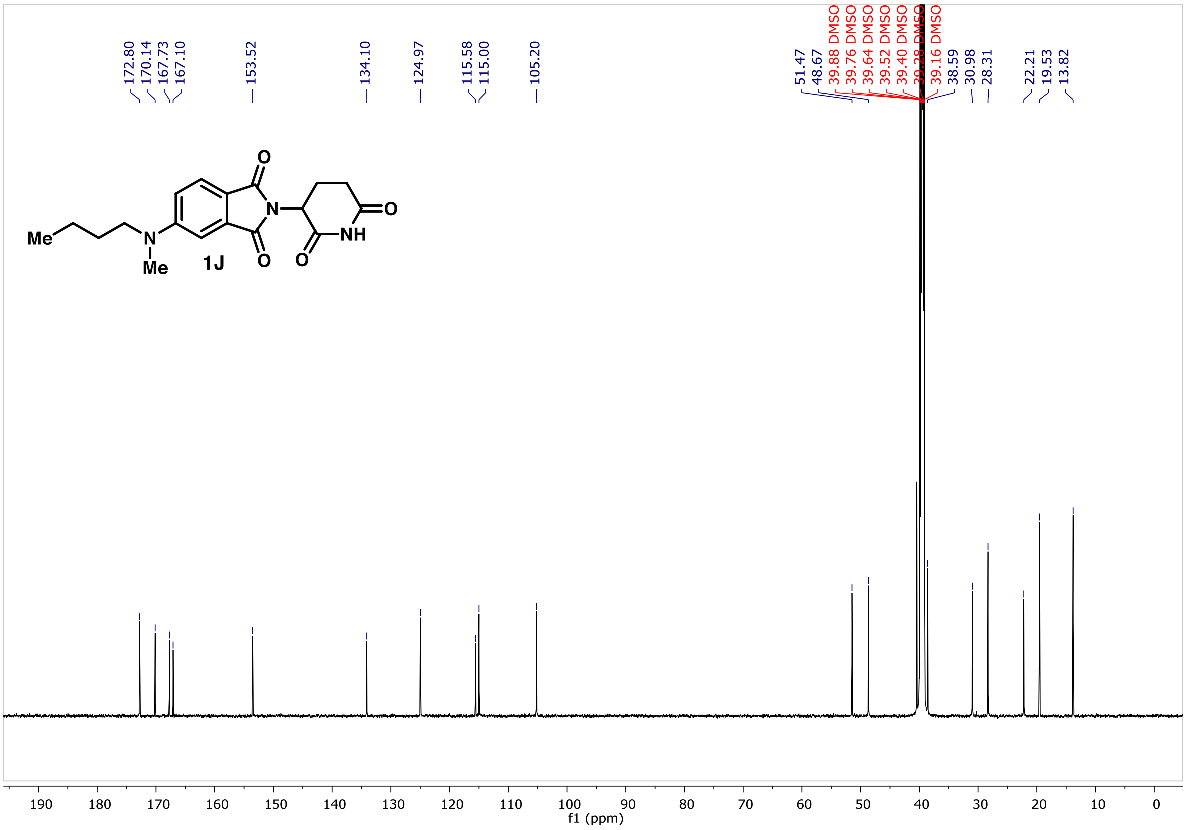

Compound **1K**: ^{1}H NMR (700 MHz, DMSO-d$_6$)

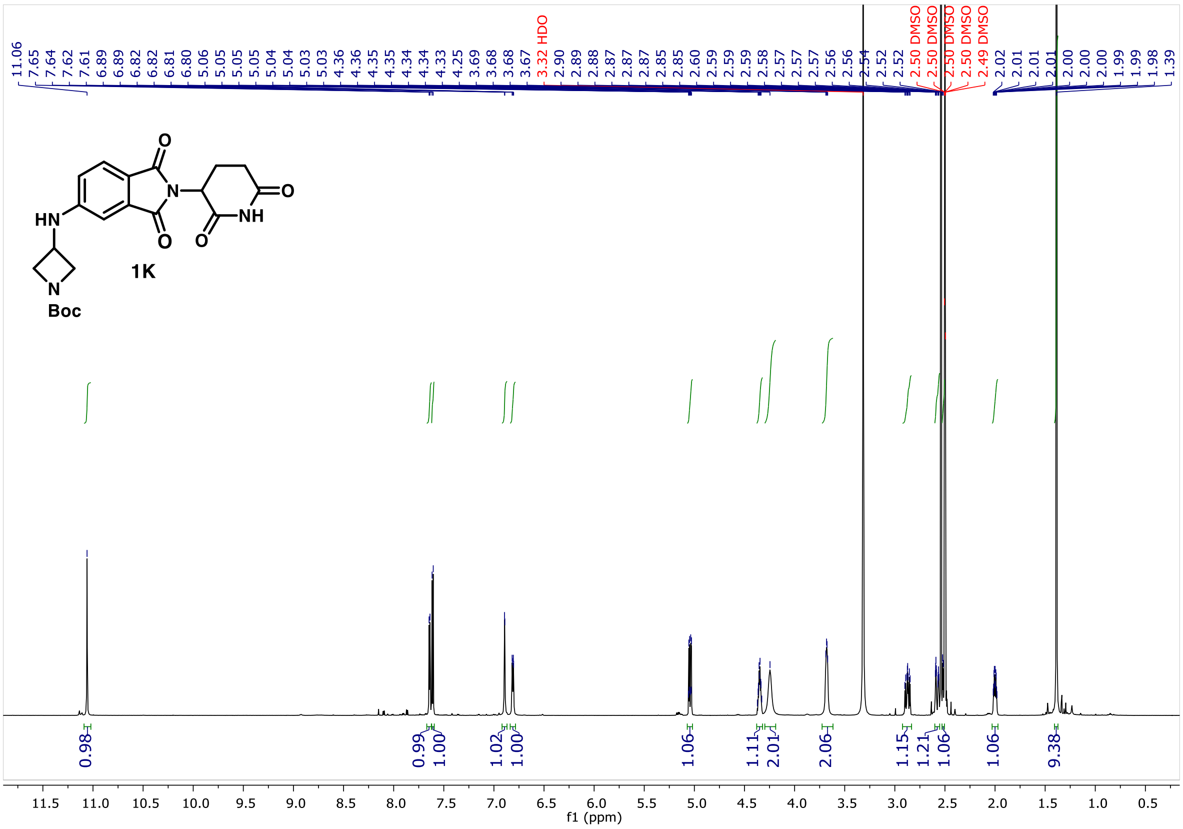

Compound **1K**: ^{13}C NMR (176 MHz, DMSO-d$_6$)

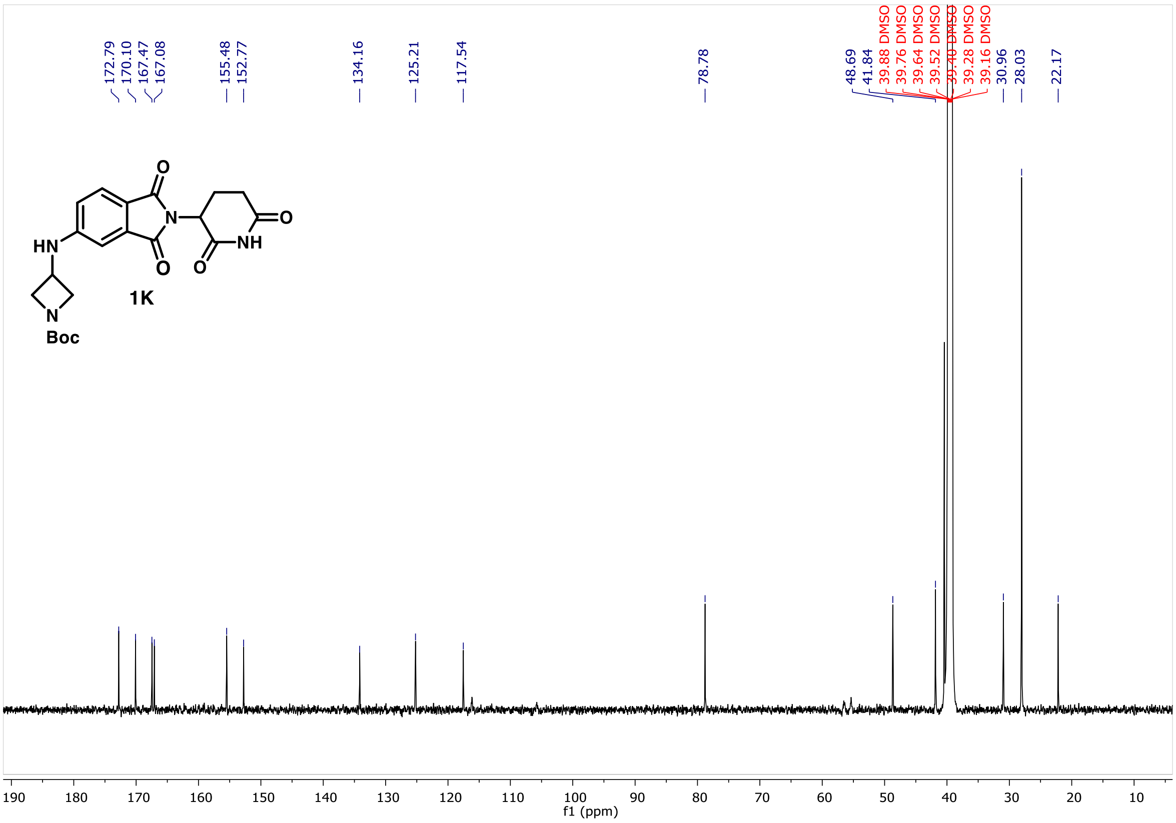

Compound **2A**: ^{1}H NMR (400 MHz, DMSO-d$_6$)

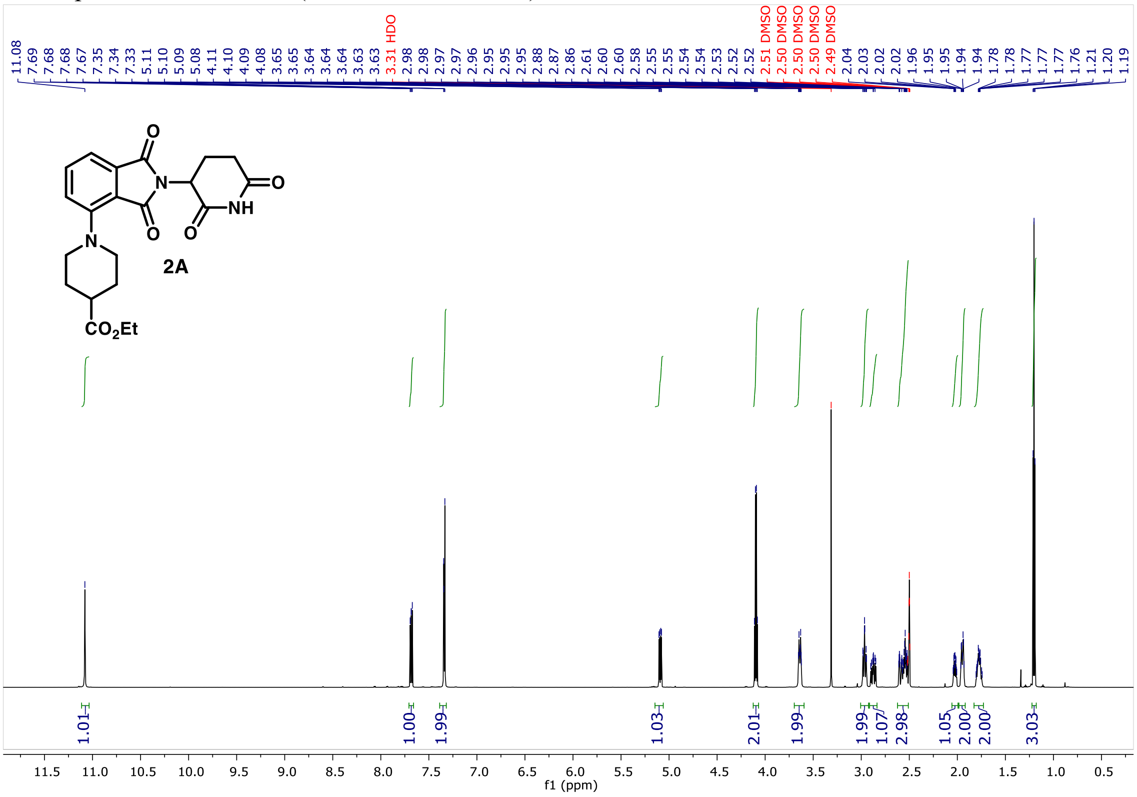

Compound **2A**: ^{13}C NMR (126 MHz, DMSO-d$_6$)

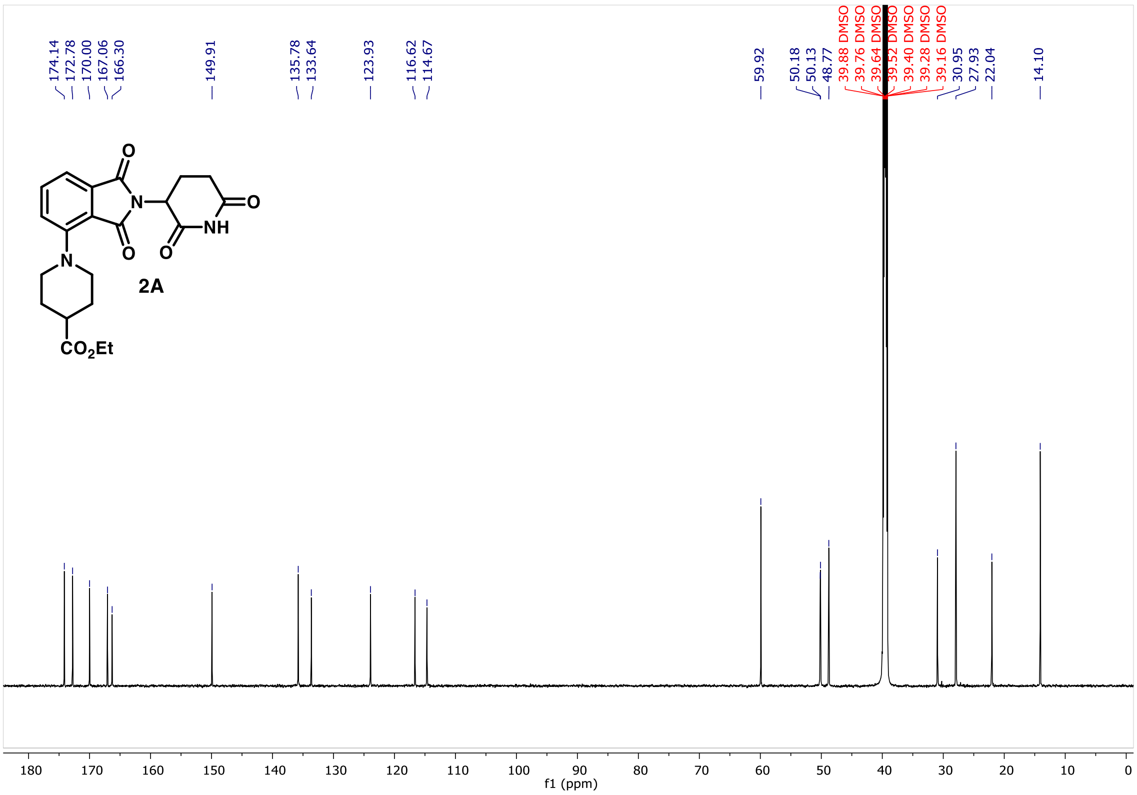

Compound **2B**: ^{1}H NMR (400 MHz, DMSO-d$_6$)

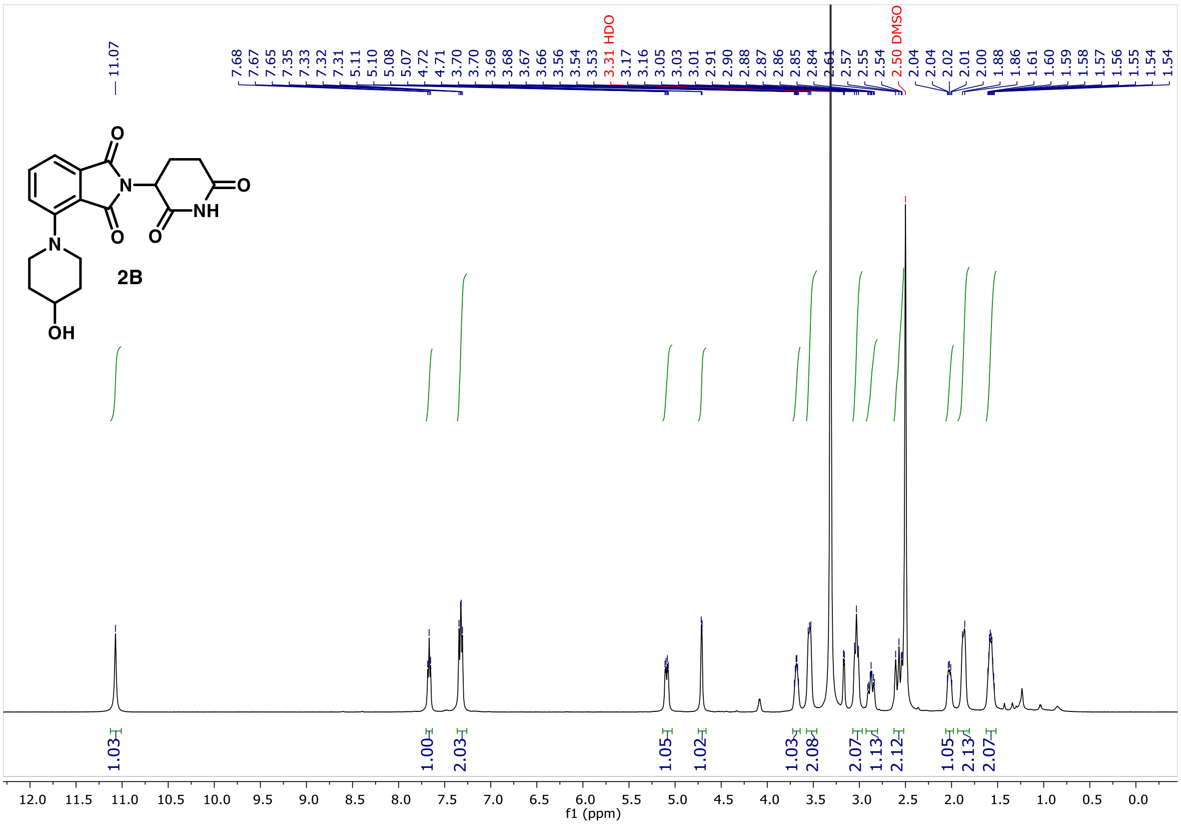

Compound **2B**: ^{13}C NMR (126 MHz, DMSO-d$_6$)

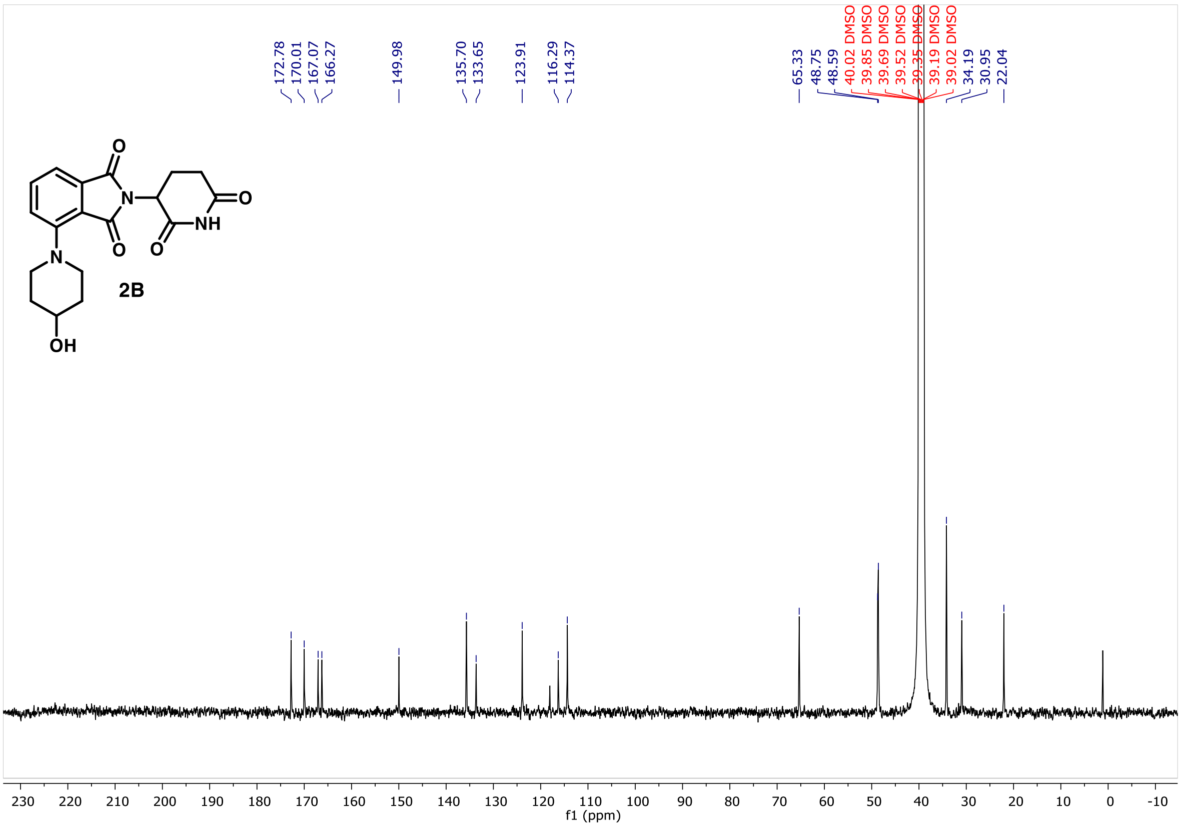

Compound **2C**: ^{1}H NMR (400 MHz, DMSO-d$_6$)

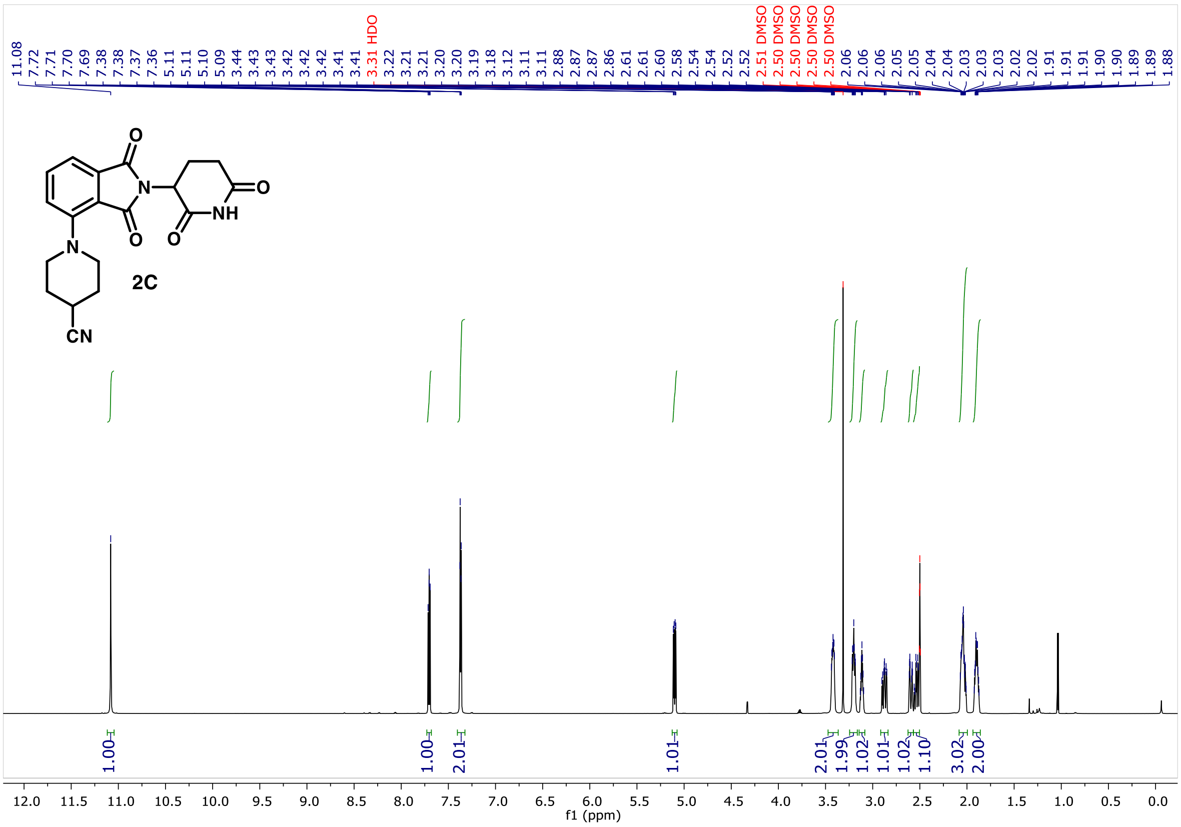

Compound **2C**: ^{13}C NMR (126 MHz, DMSO-d$_6$)

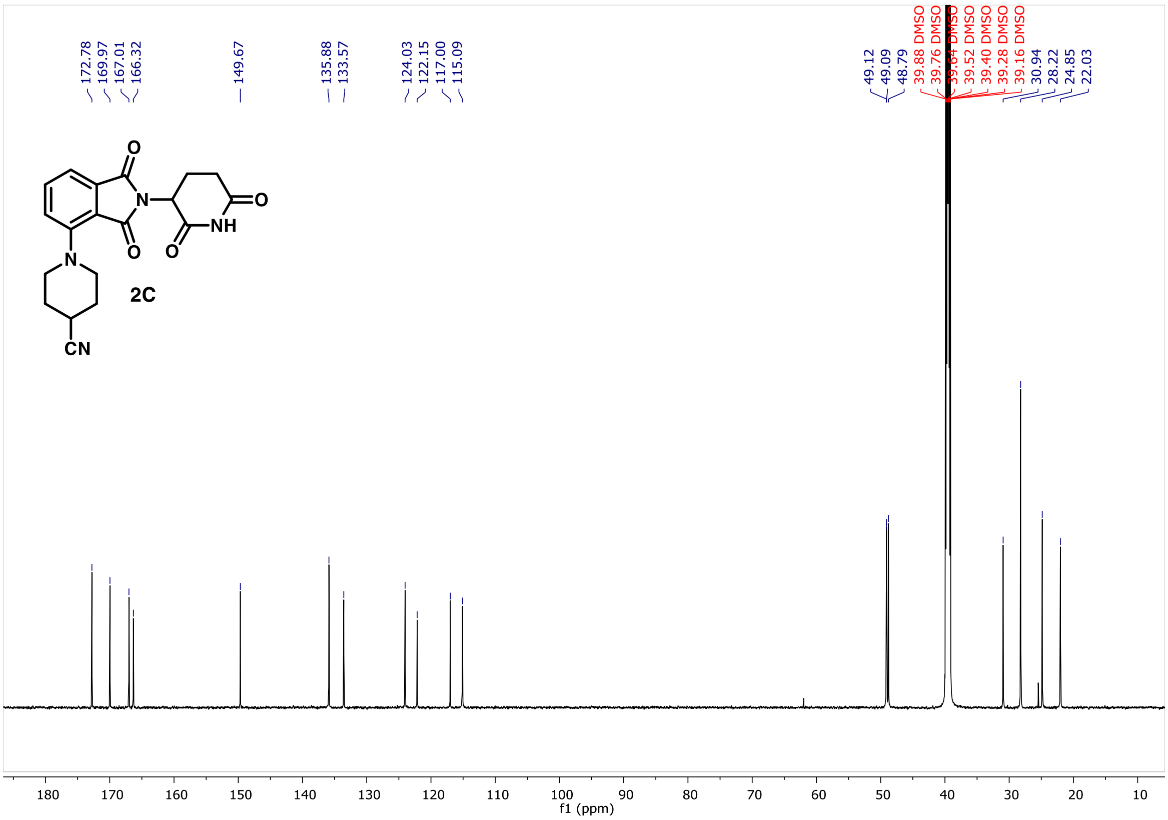

Compound **2D**: ^{1}H NMR (400 MHz, DMSO-d$_6$)

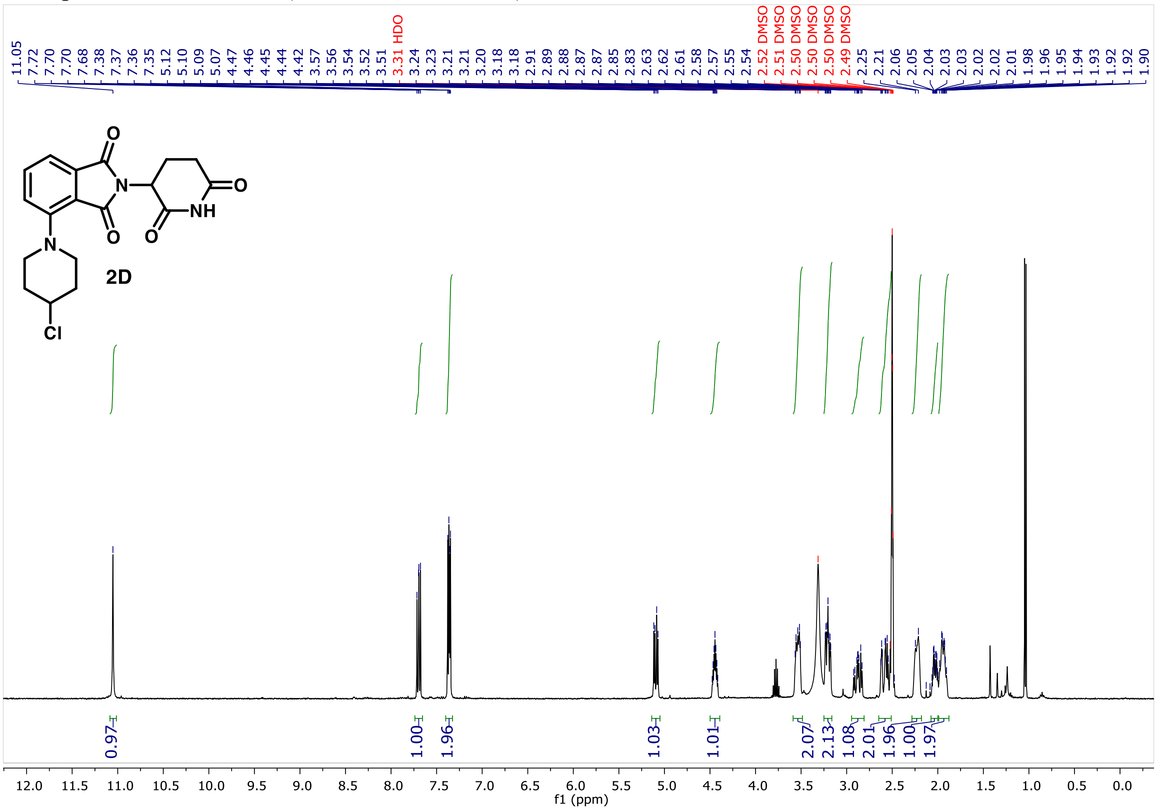

Compound **2D**: ^{13}C NMR (126 MHz, DMSO-d$_6$)

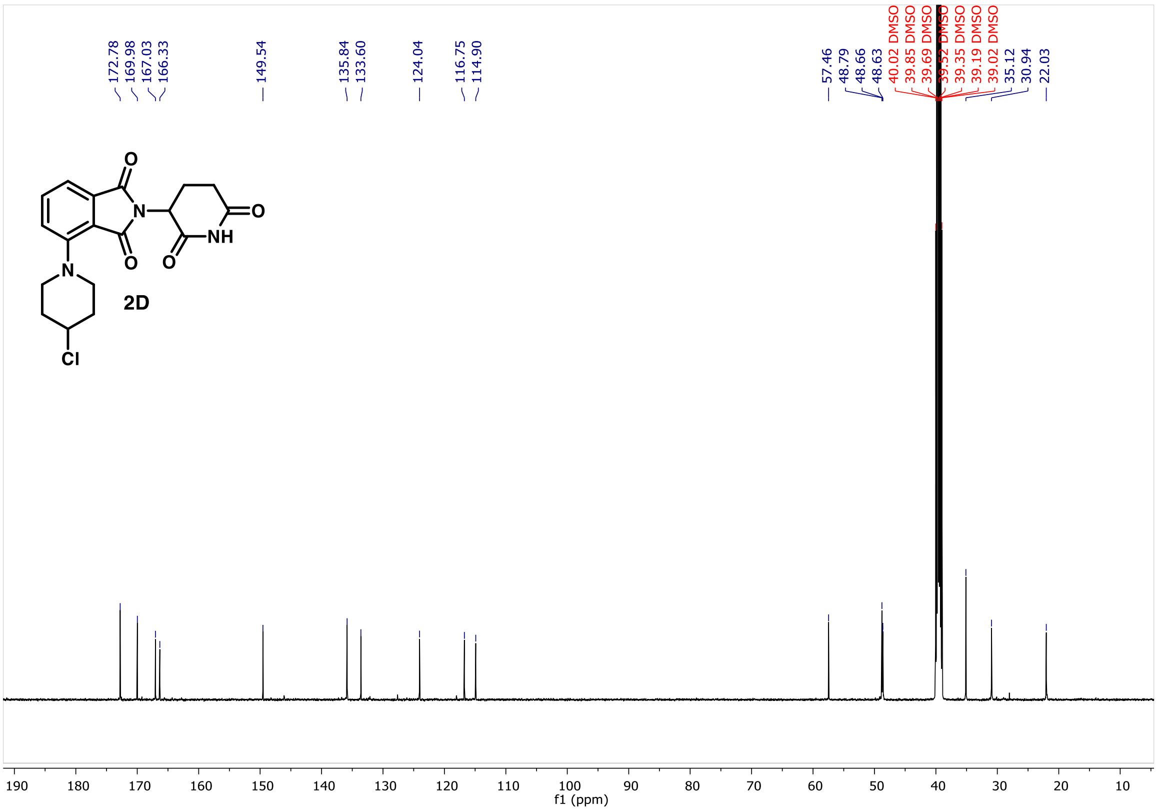

Compound **2E**: ^{1}H NMR (400 MHz, DMSO-d$_6$)

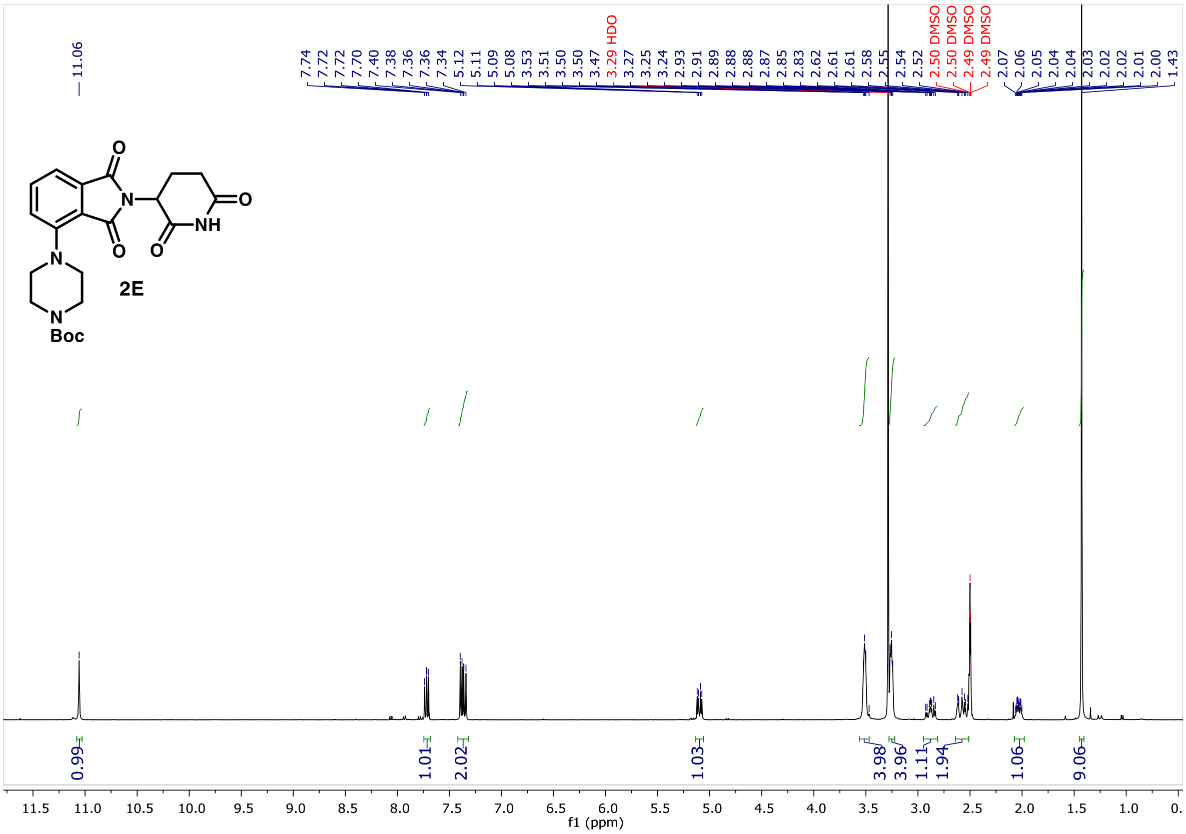

Compound **2E**: ^{13}C NMR (126 MHz, DMSO-d$_6$)

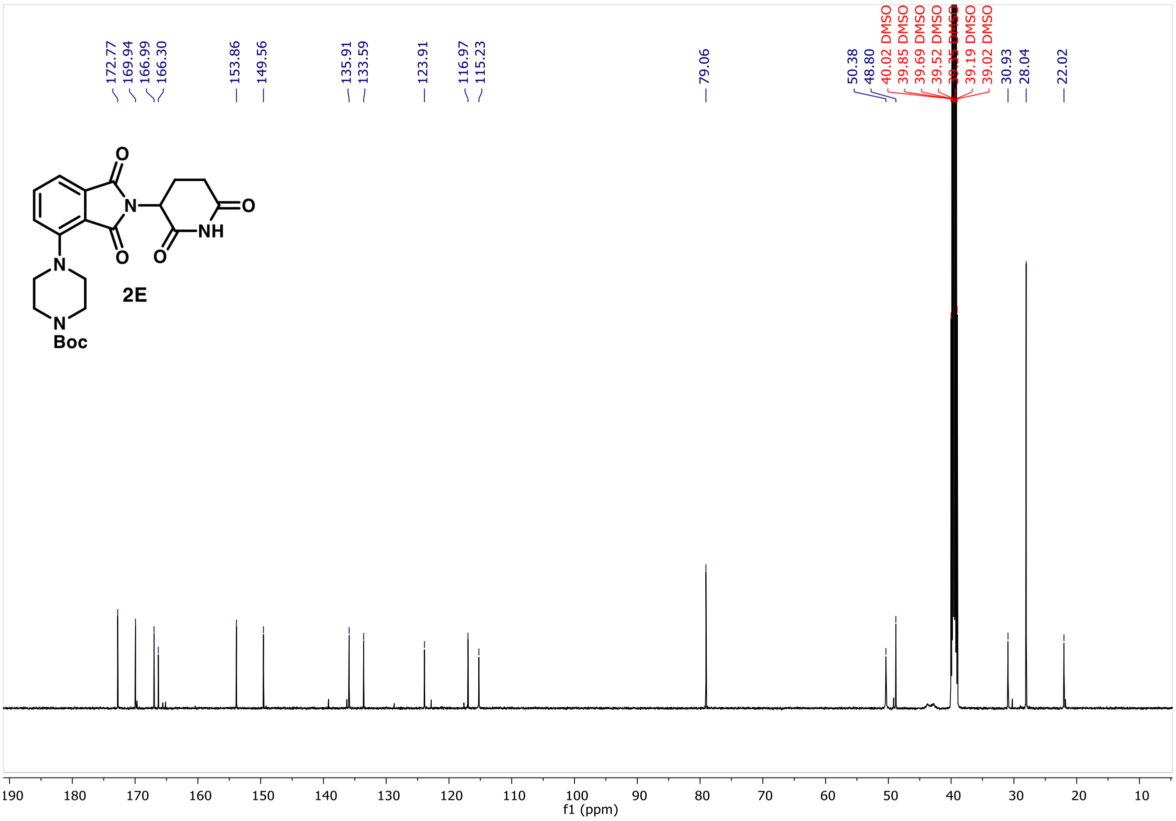

Compound **2F**: ^{1}H NMR (700 MHz, DMSO-d$_6$)

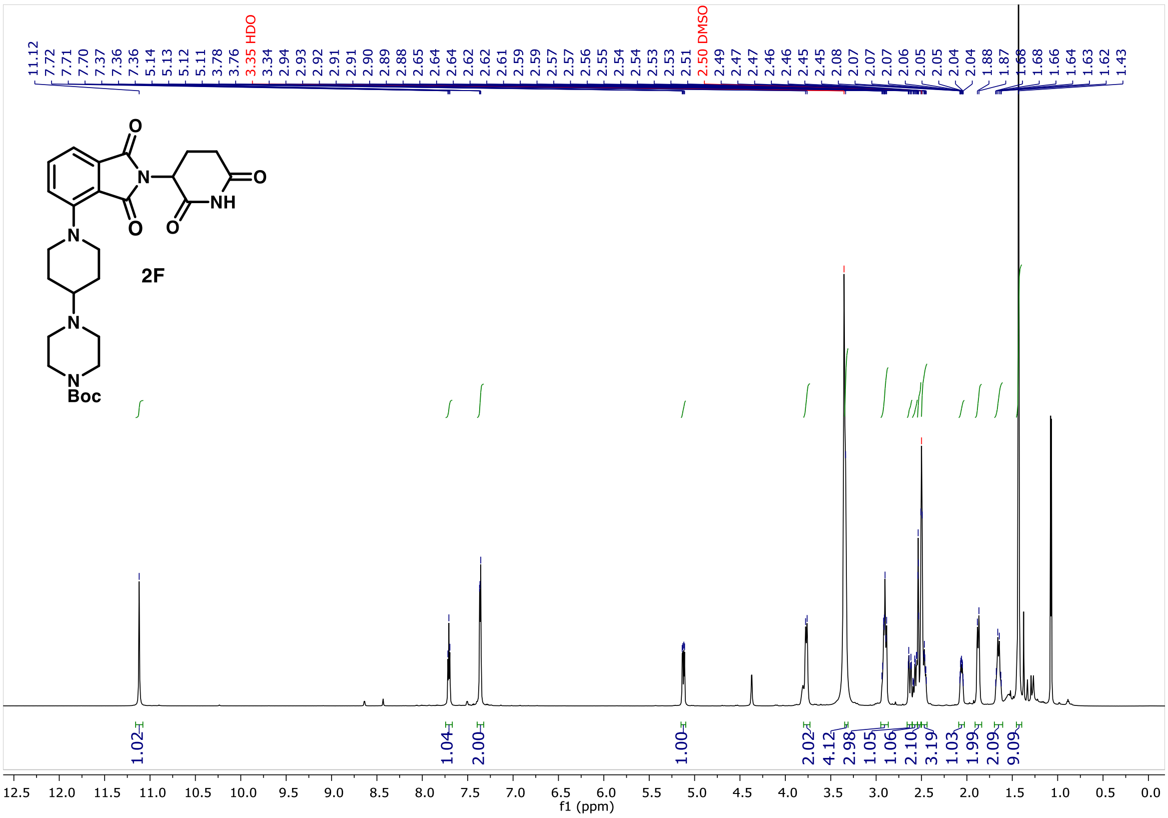

Compound **2F**: ^{13}C NMR (176 MHz, DMSO-d$_6$)

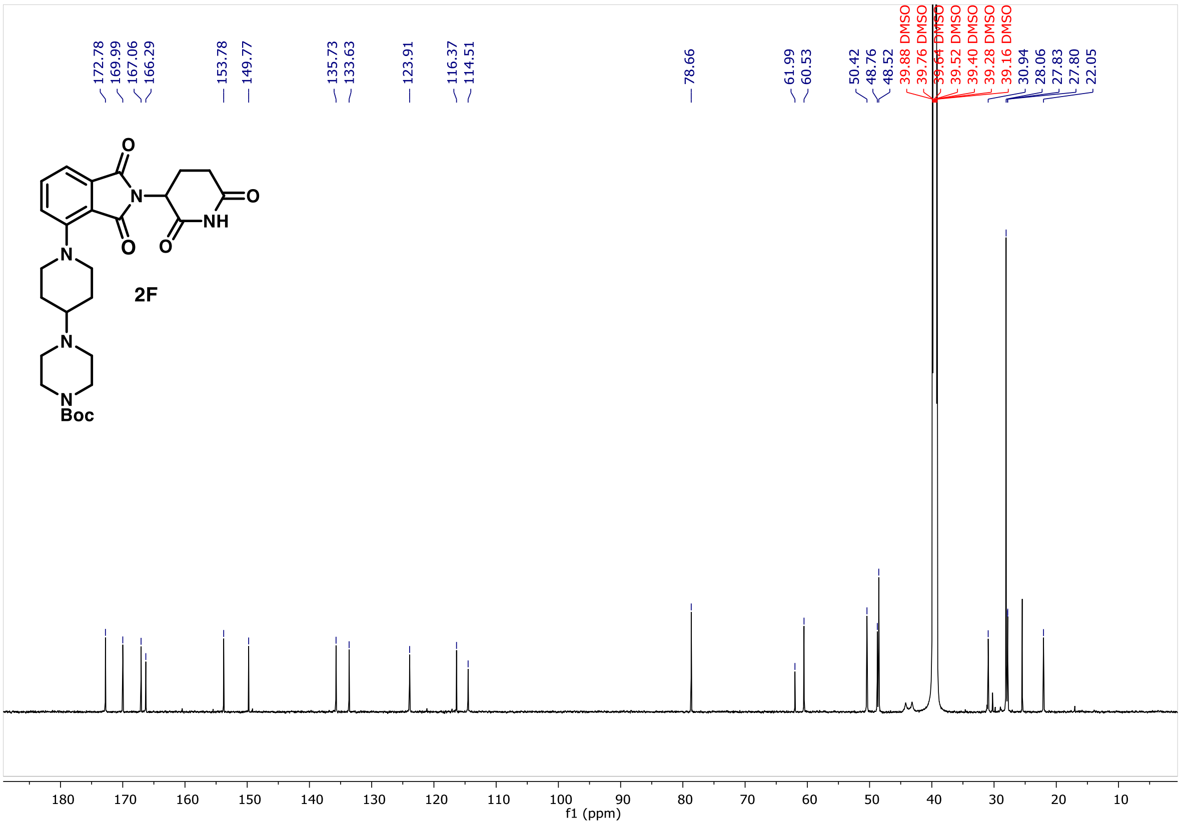

Compound **2G**: ^{1}H NMR (700 MHz, DMSO-d$_6$)

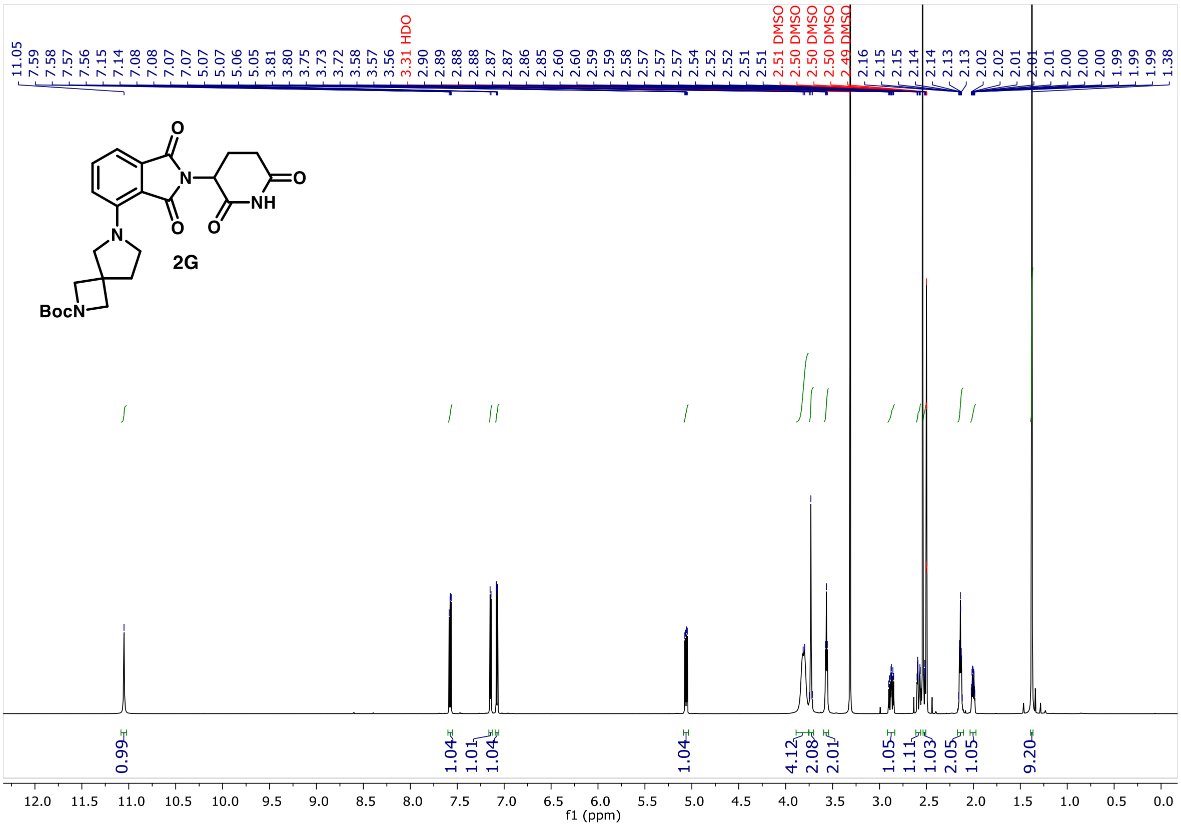

Compound **2G**: ^{13}C NMR (176 MHz, DMSO-d$_6$)

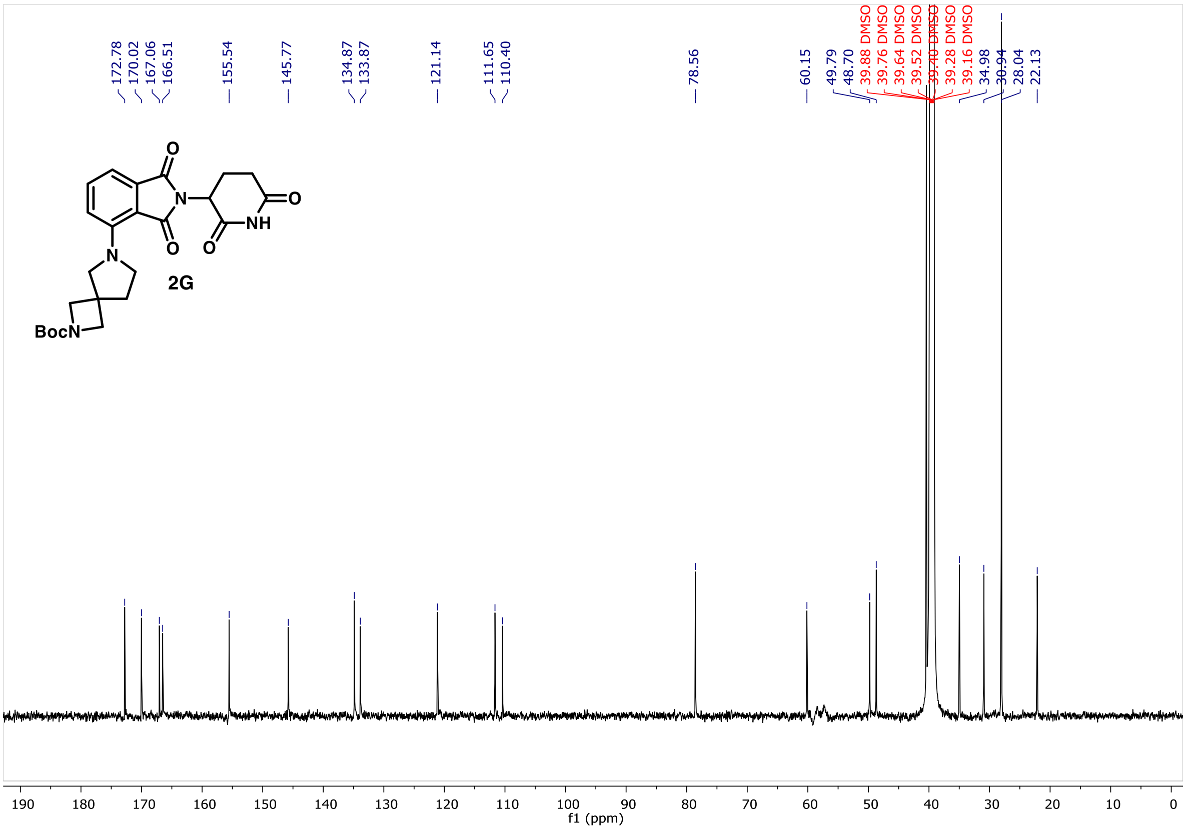

Compound **2H**: ^{1}H NMR (700 MHz, DMSO-d$_6$)

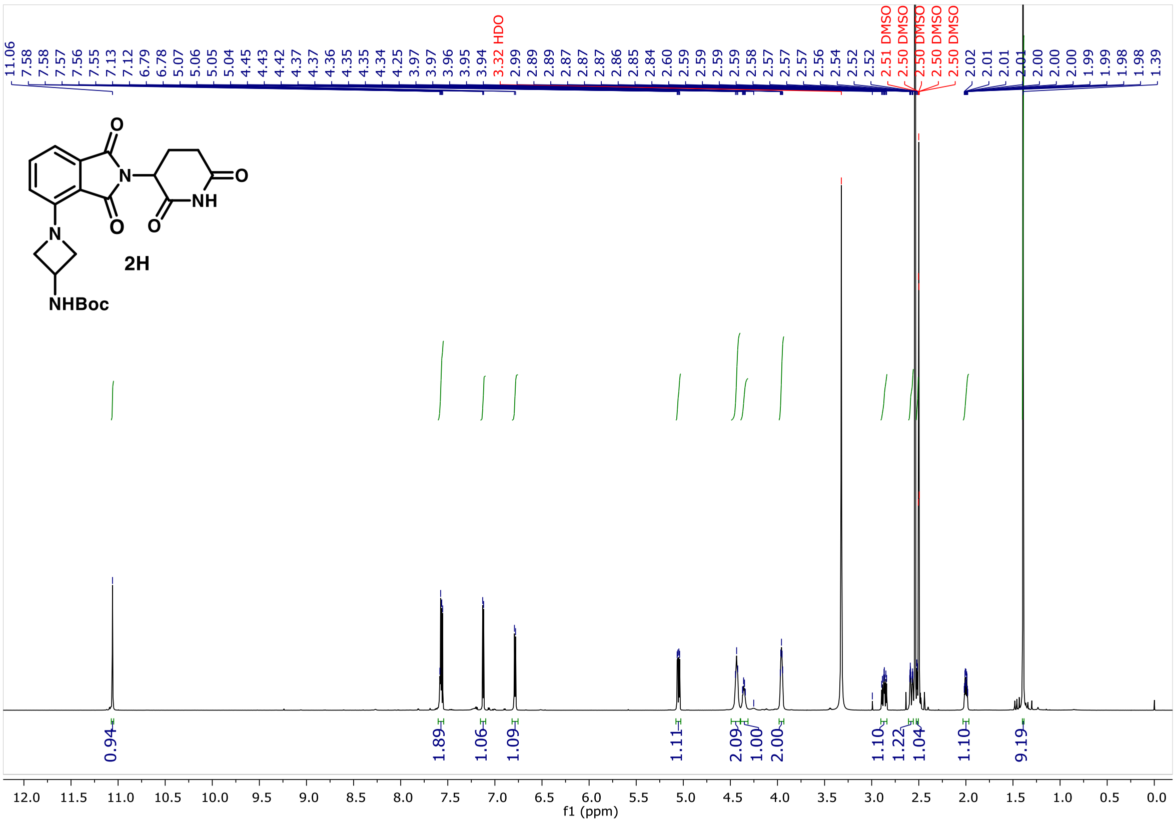

Compound **2H**: ^{13}C NMR (176 MHz, DMSO-d$_6$)

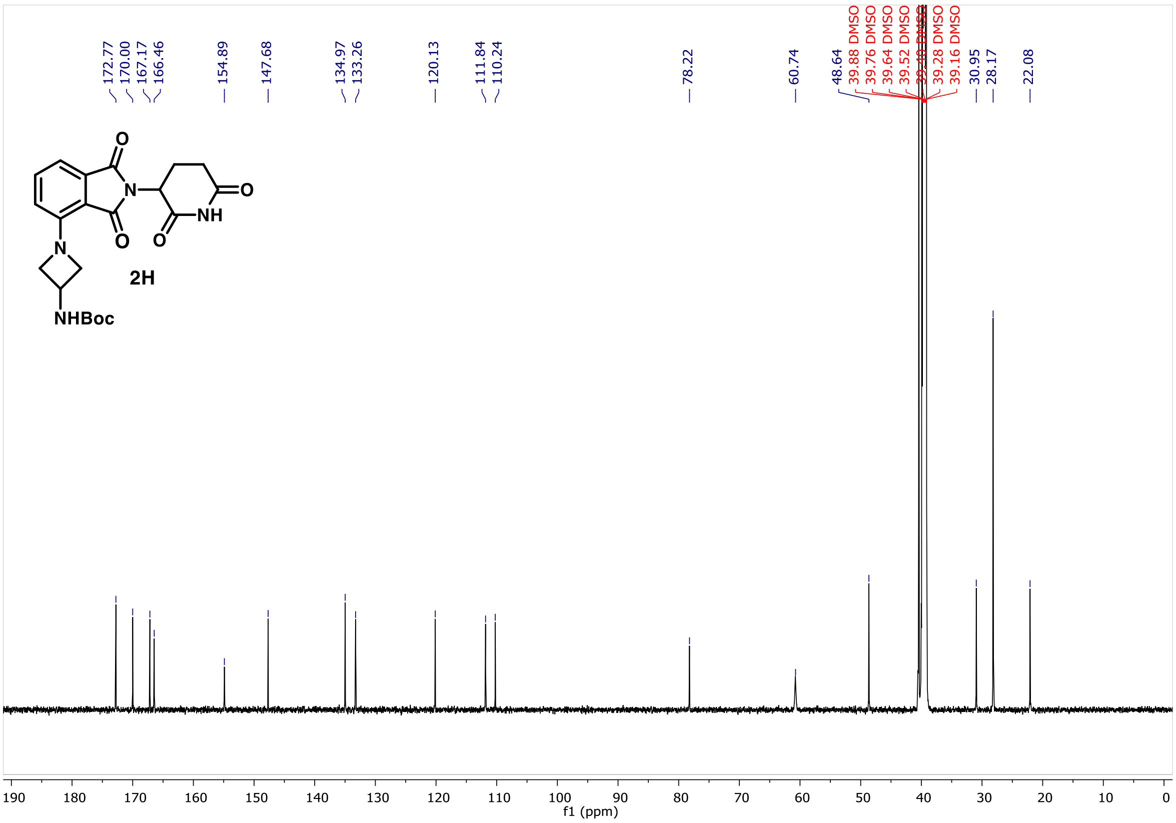

Compound **2J**: ^{1}H NMR (700 MHz, DMSO-d_6)

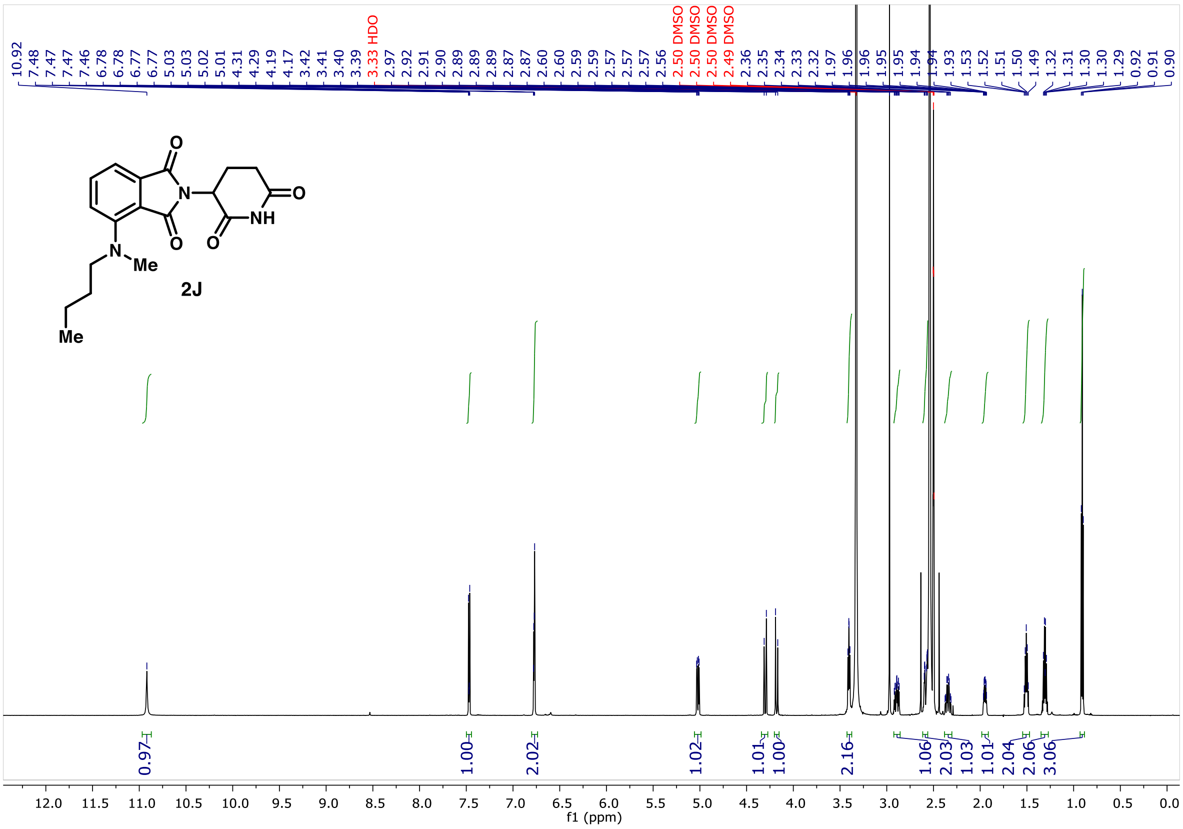

Compound **2J**: ^{13}C NMR (176 MHz, DMSO-d_6)

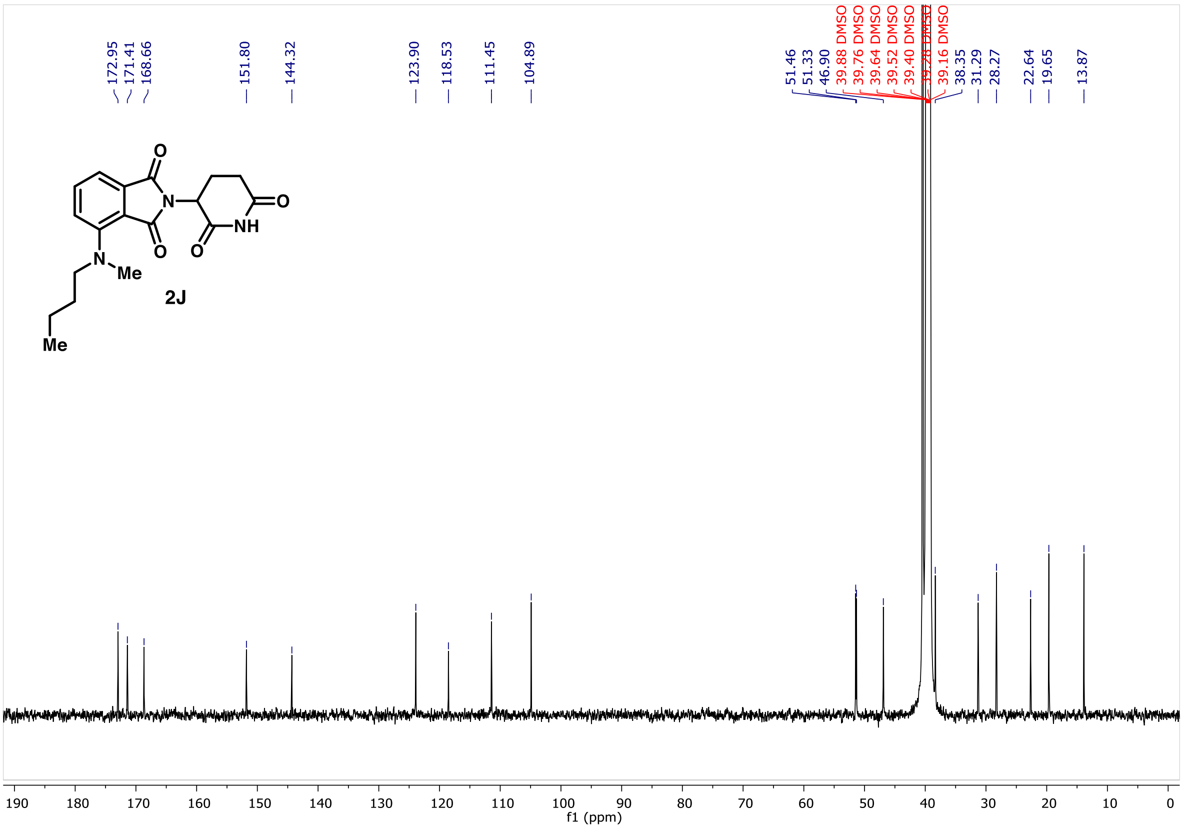

Compound **2K**: ^{1}H NMR (700 MHz, DMSO-d_6)

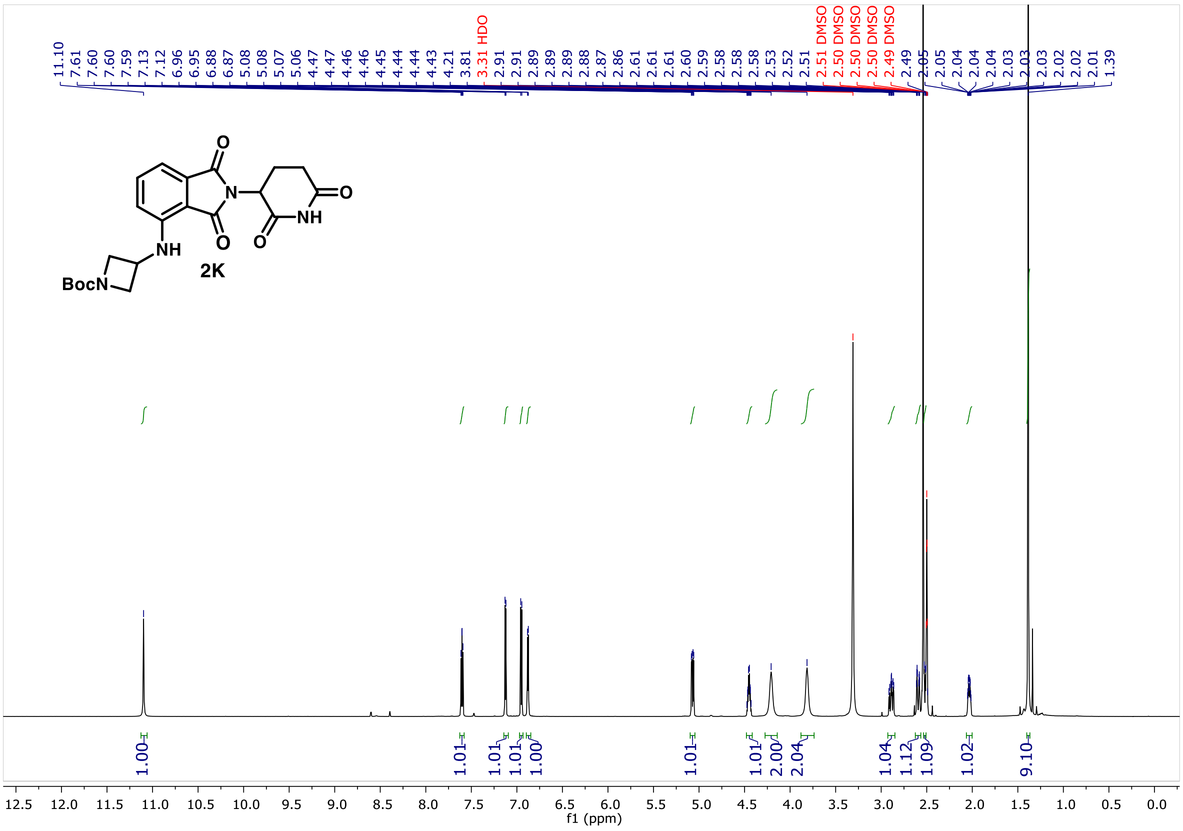

Compound **2K**: ^{13}C NMR (176 MHz, DMSO-d_6)

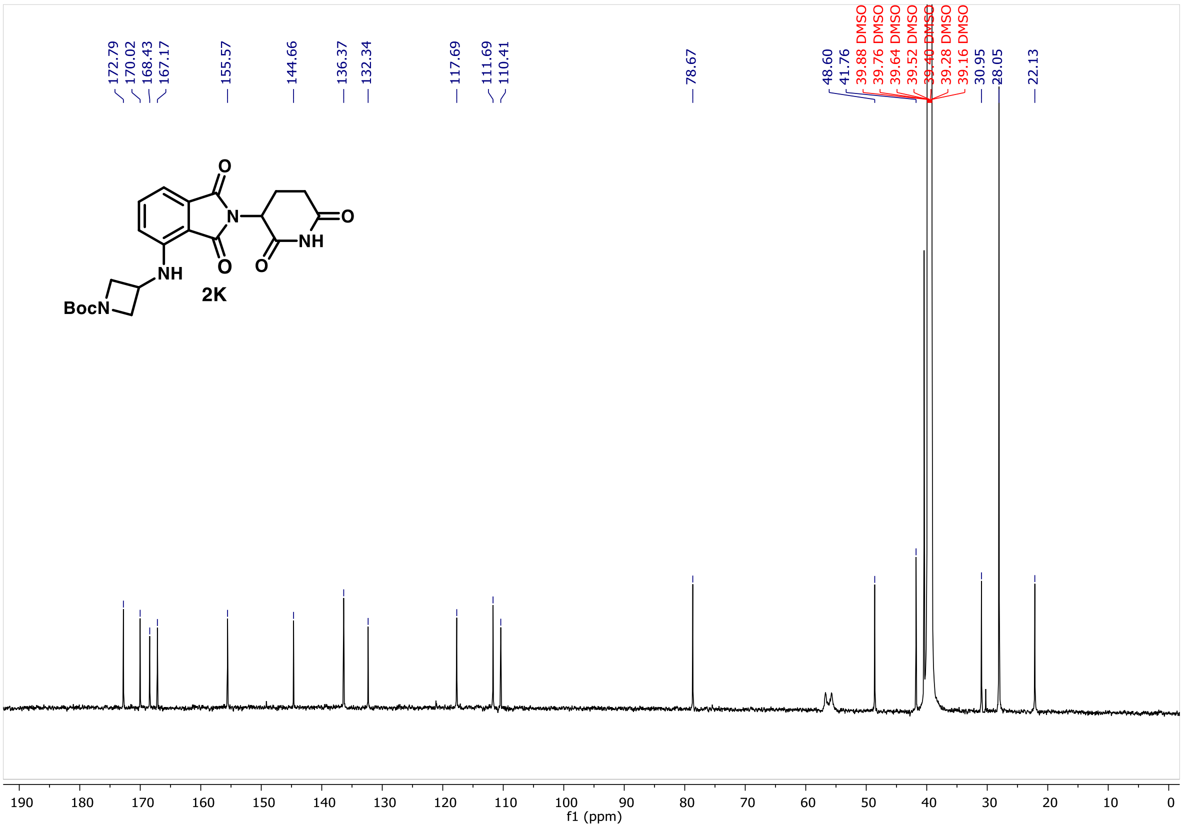

Compound **3A**: ^{1}H NMR (400 MHz, DMSO-d$_6$)

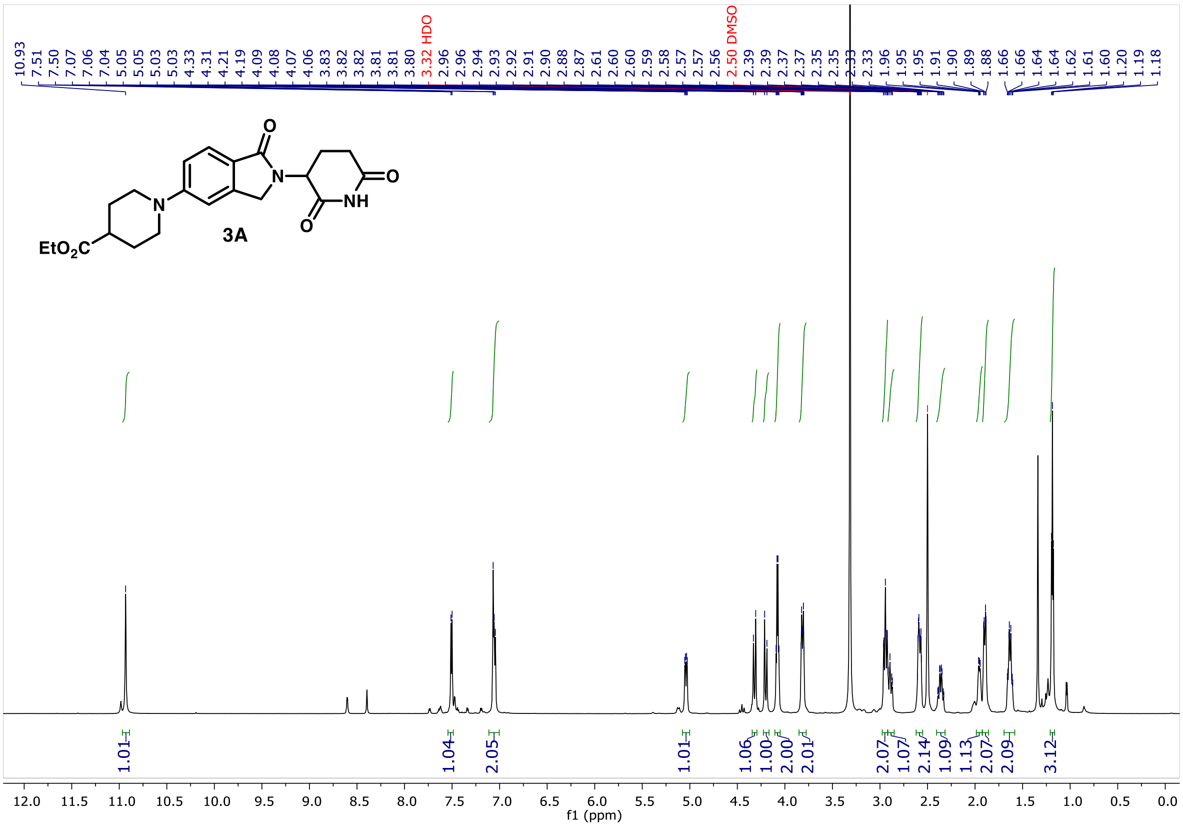

Compound **3A**: ^{13}C NMR (126 MHz, DMSO-d$_6$)

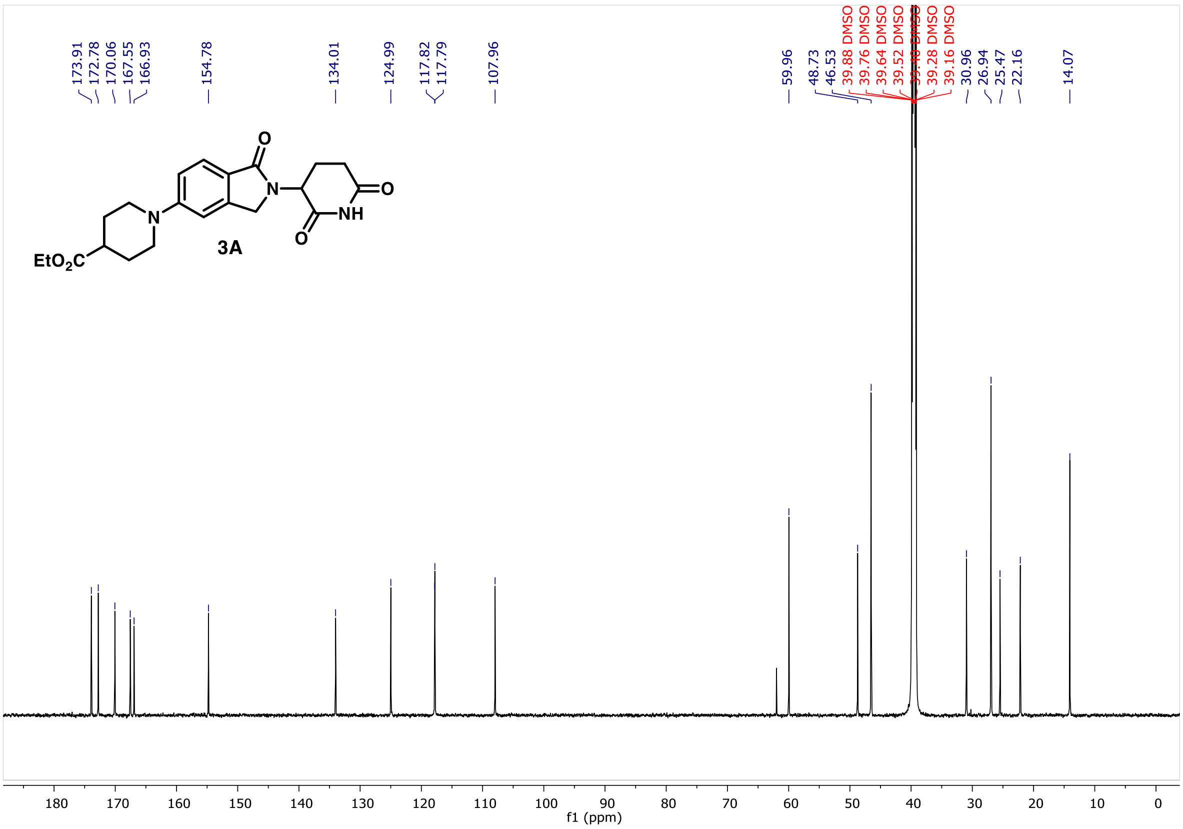

Compound **3C**: ^{1}H NMR (400 MHz, DMSO-d_6)

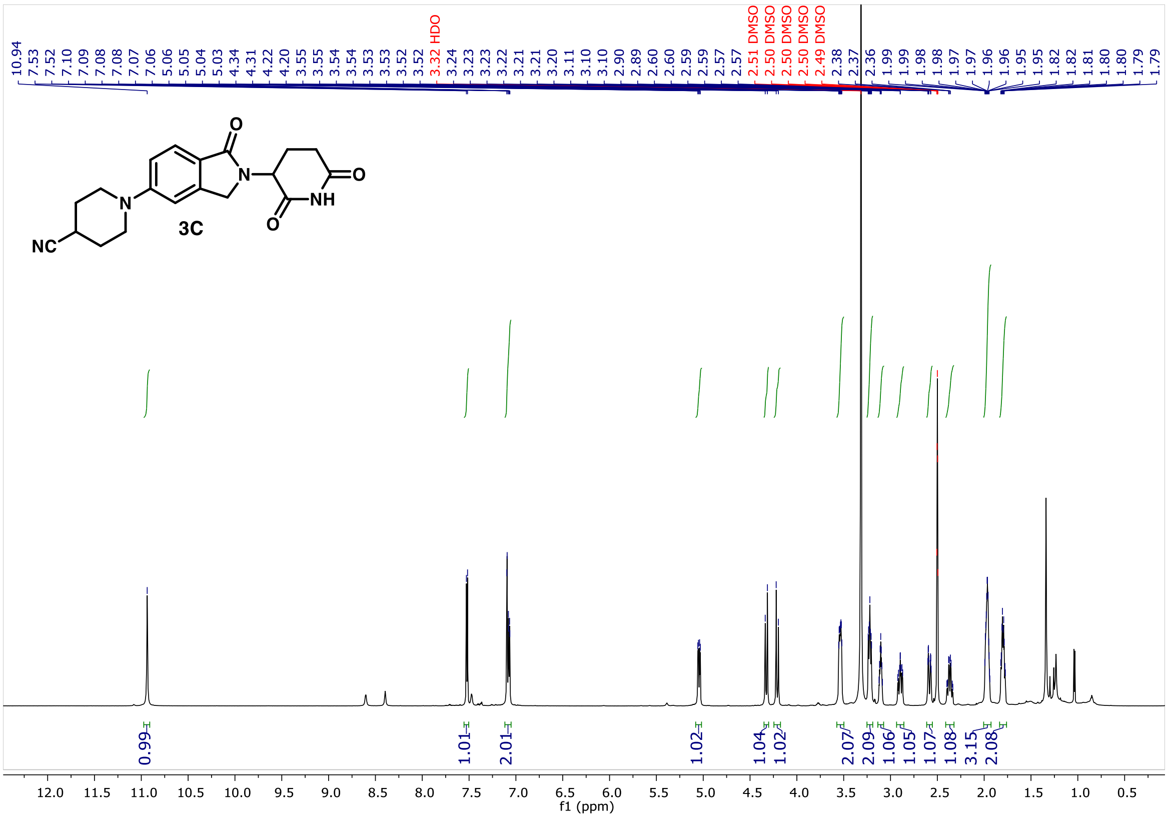

Compound **3C**: ^{13}C NMR (126 MHz, DMSO-d_6)

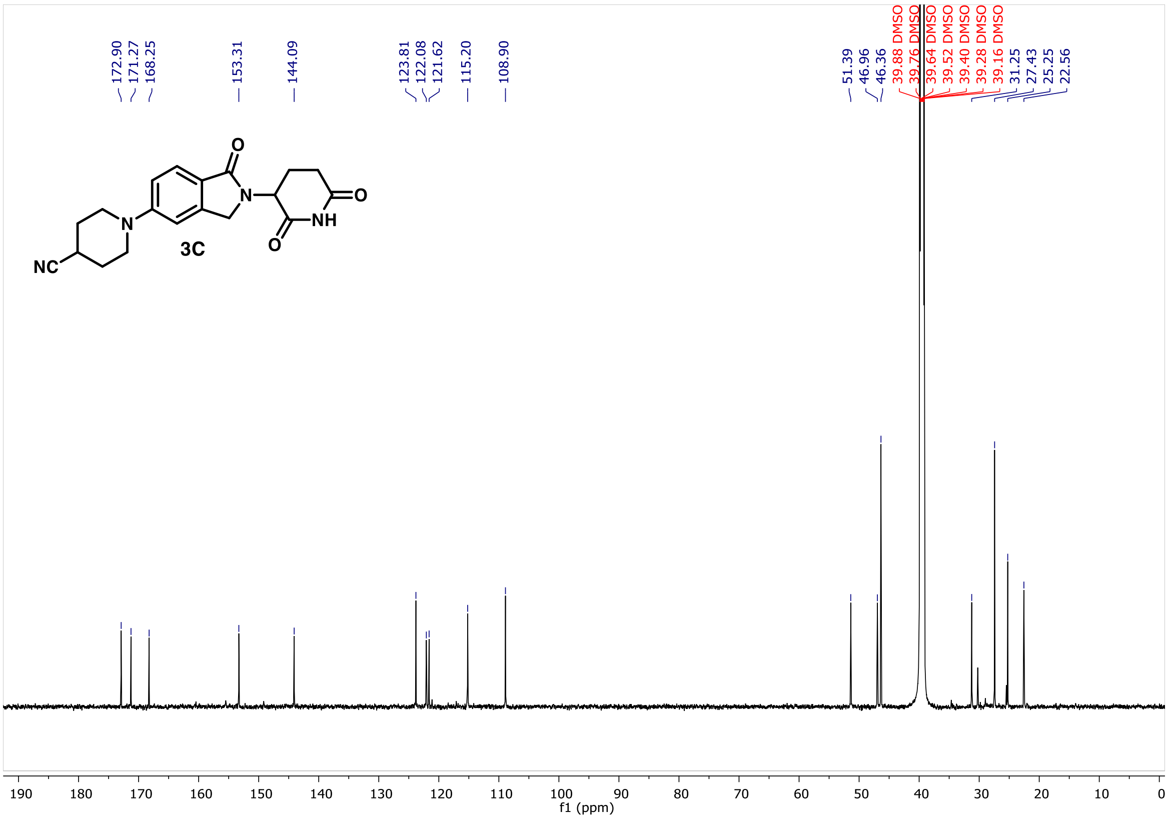

Compound **3D**: ^{1}H NMR (400 MHz, DMSO-d_6)

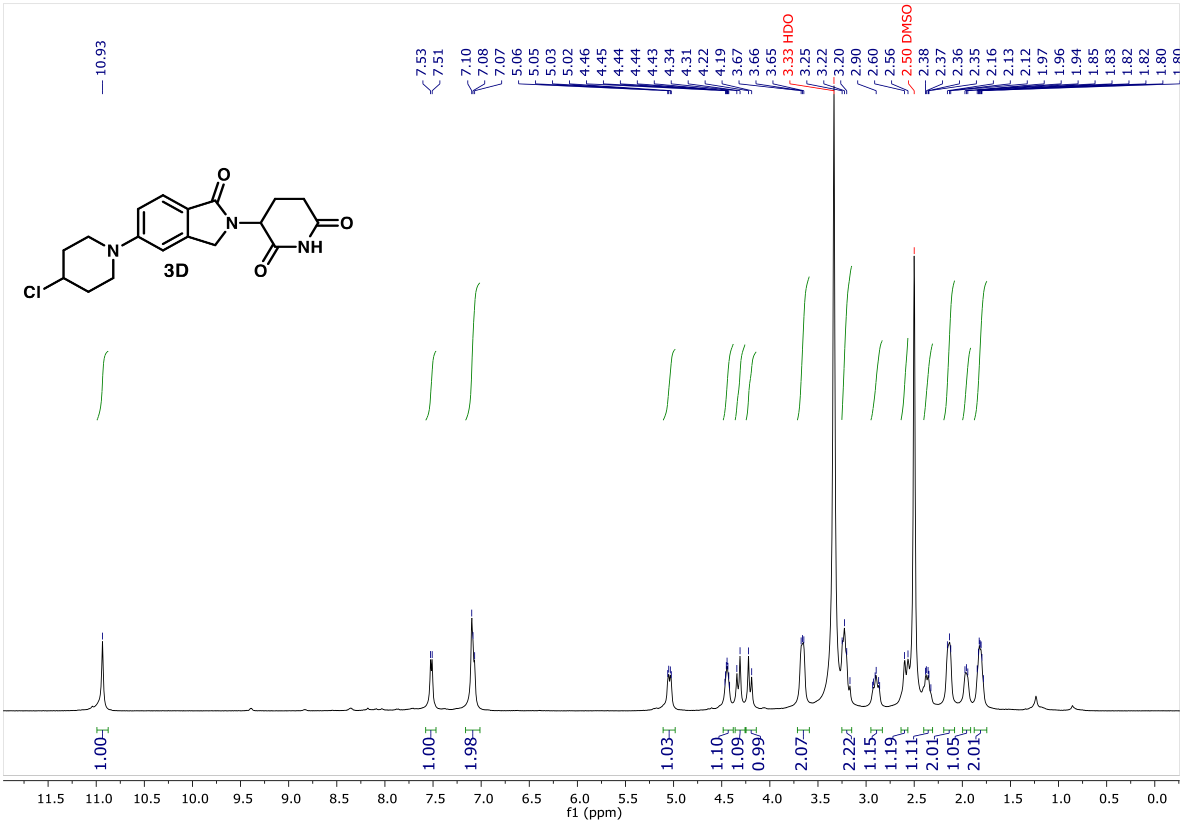

Compound **3D**: ^{13}C NMR (126 MHz, DMSO-d_6)

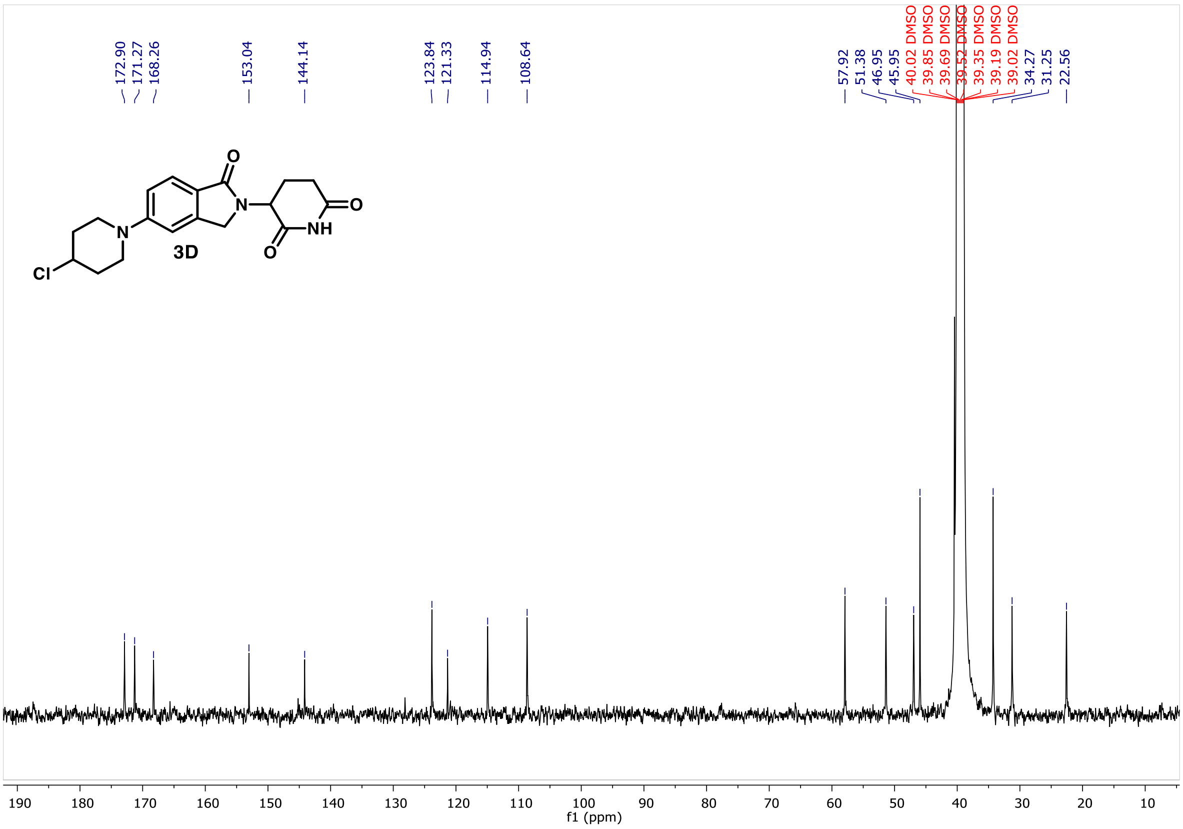

Compound **3E**: ^{1}H NMR (400 MHz, DMSO-d_6)

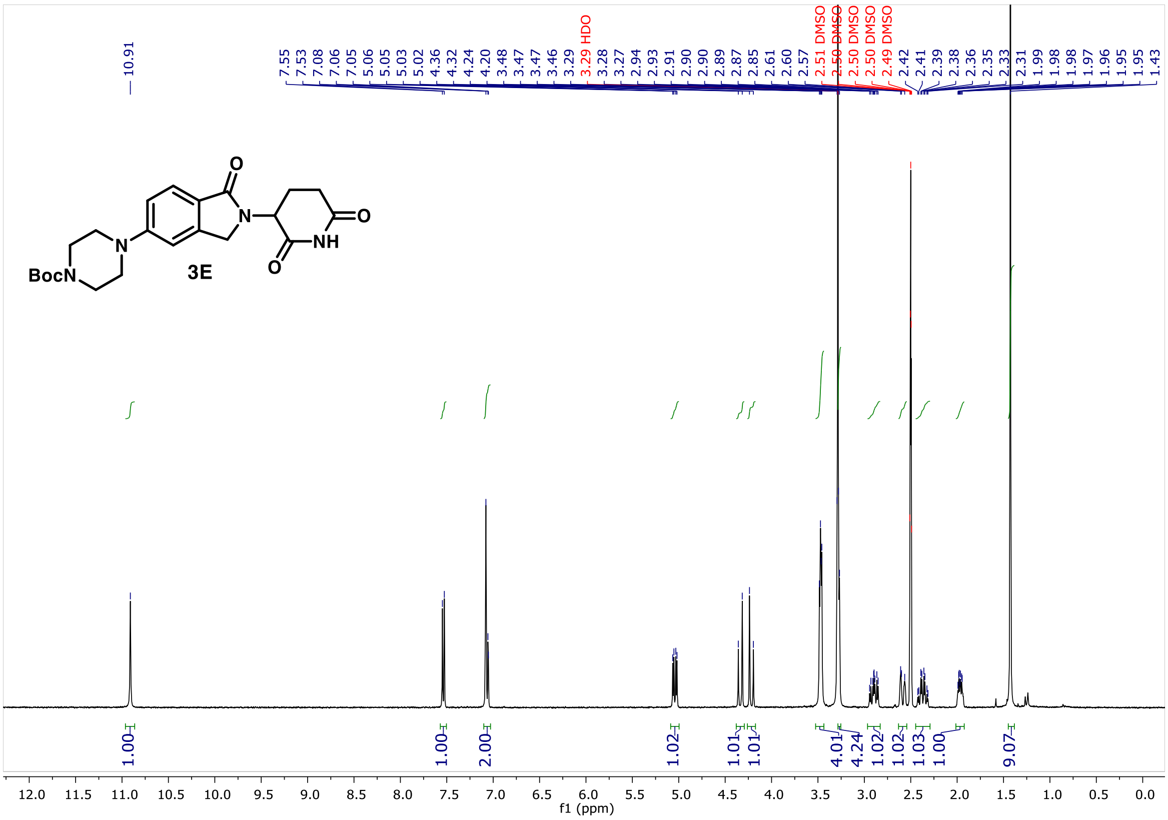

Compound **3E**: ^{13}C NMR (126 MHz, DMSO-d_6)

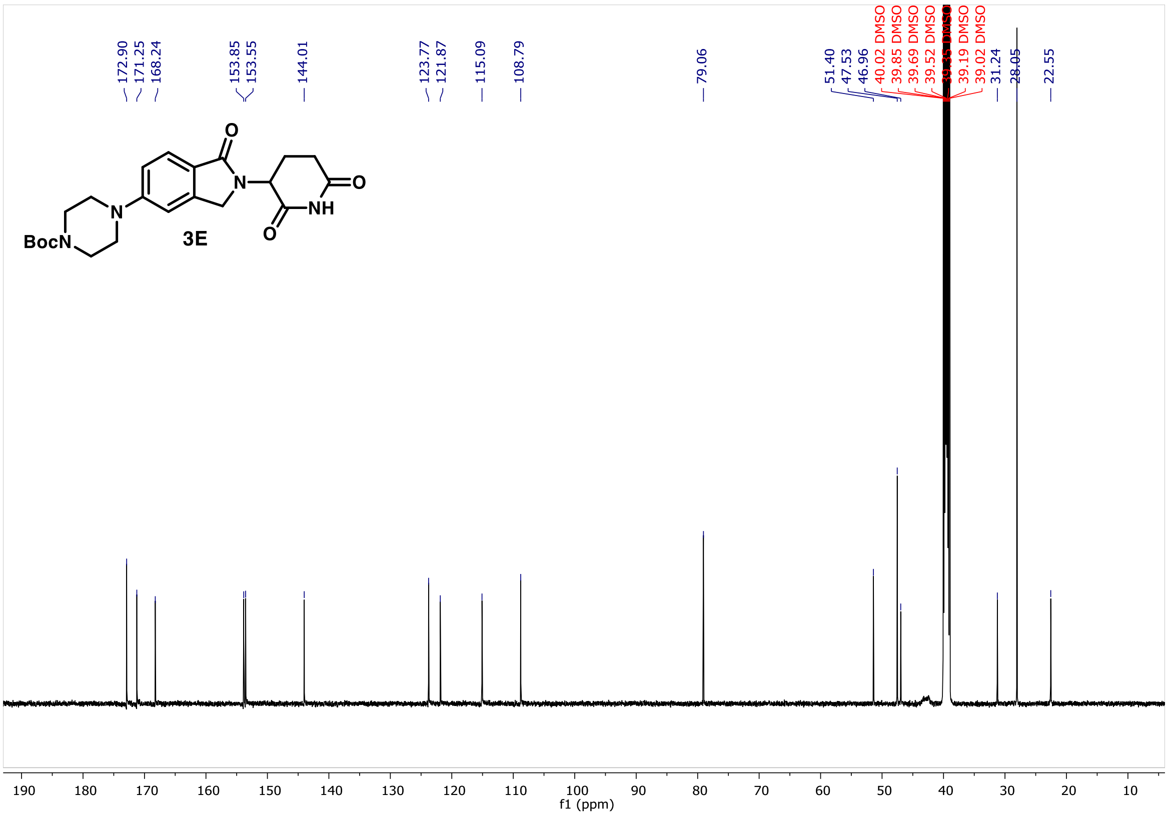

Compound **3F**: ^{1}H NMR (700 MHz, DMSO-d$_6$)

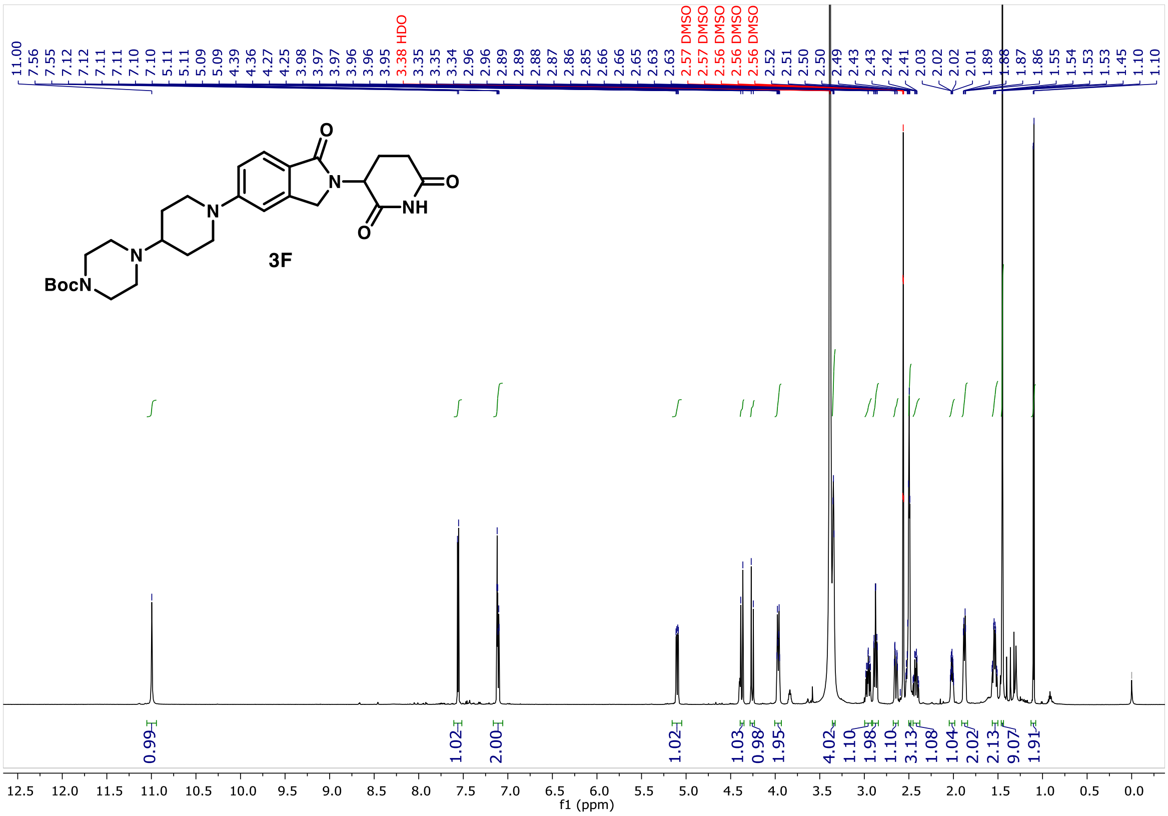

Compound **3F**: ^{13}C NMR (176 MHz, DMSO-d$_6$)

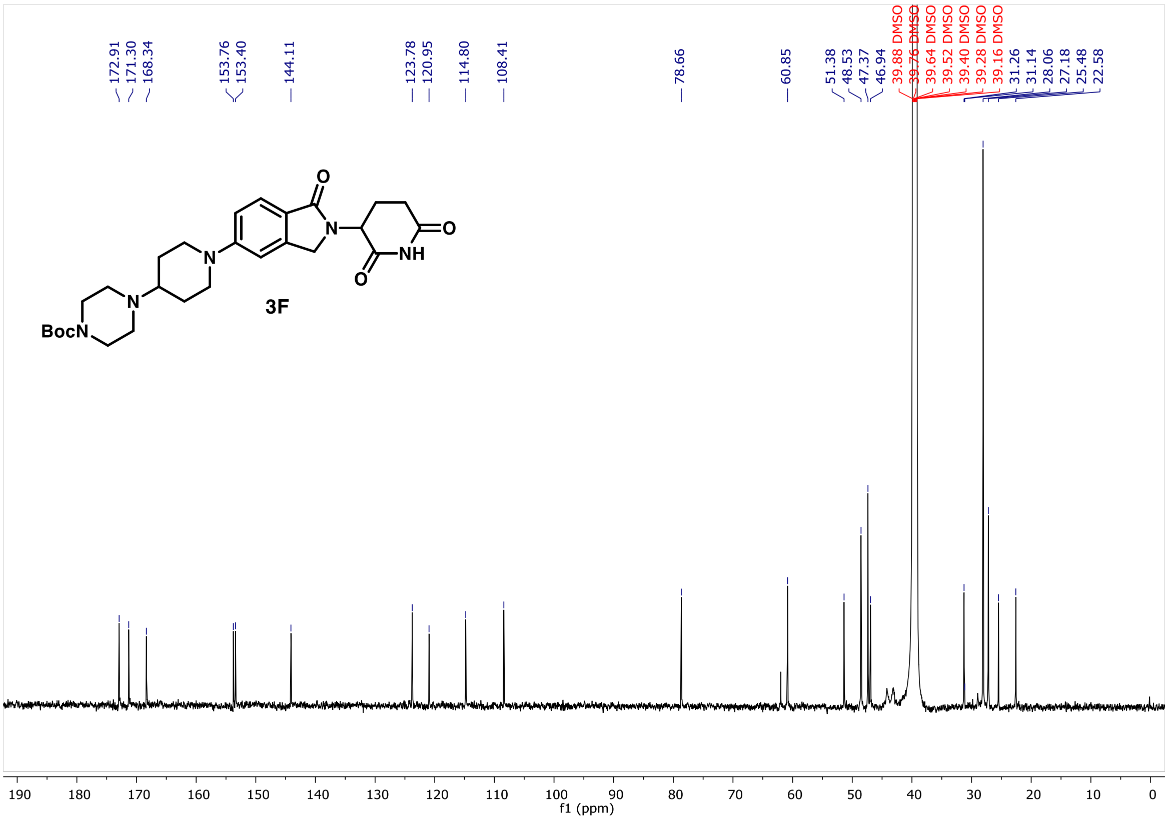

Compound **3H**: ^{1}H NMR (700 MHz, DMSO-d$_6$)

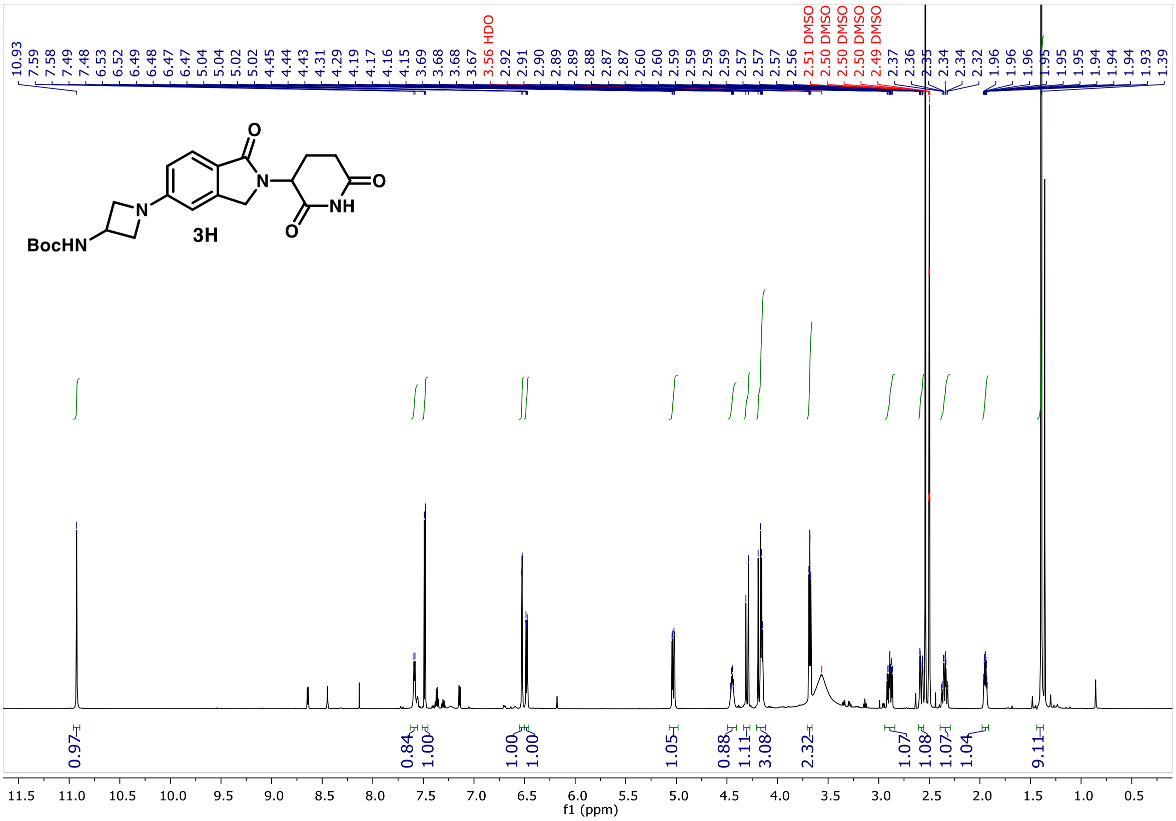

Compound **3H**: ^{13}C NMR (176 MHz, DMSO-d$_6$)

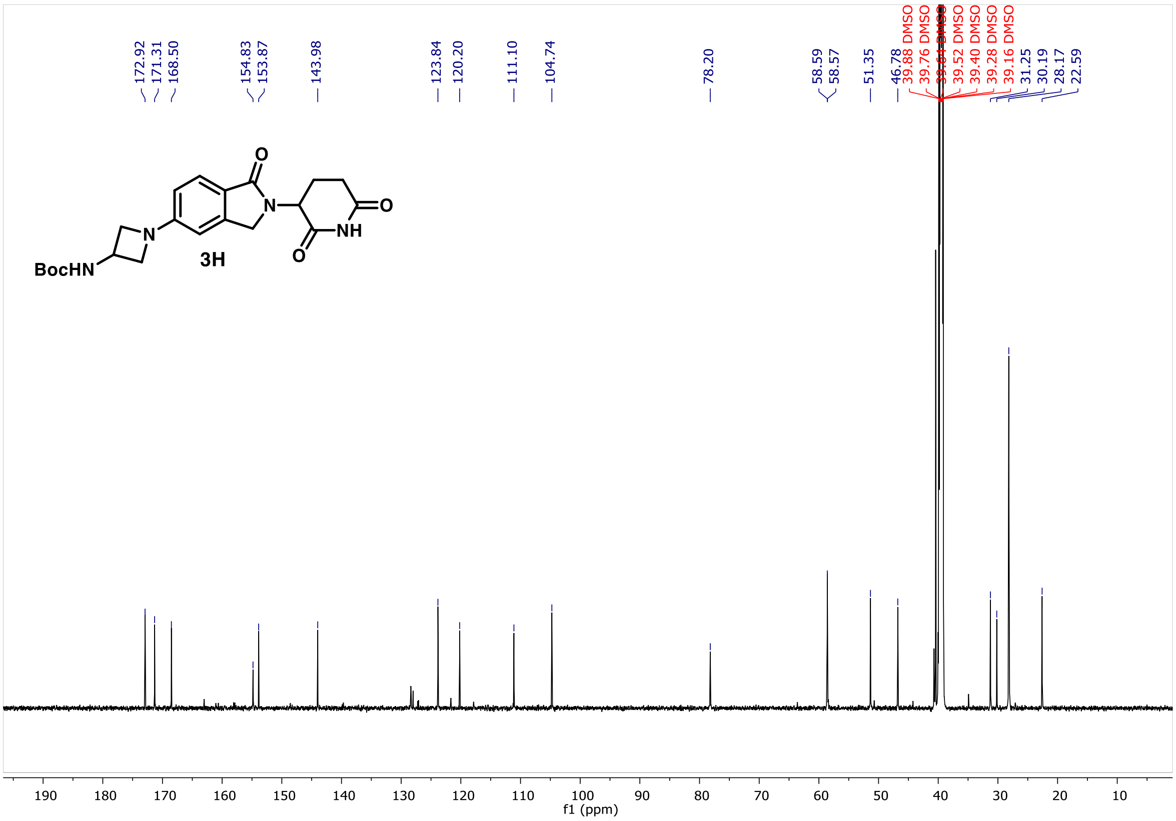

Compound **3J**: ^{1}H NMR (700 MHz, DMSO-d_6)

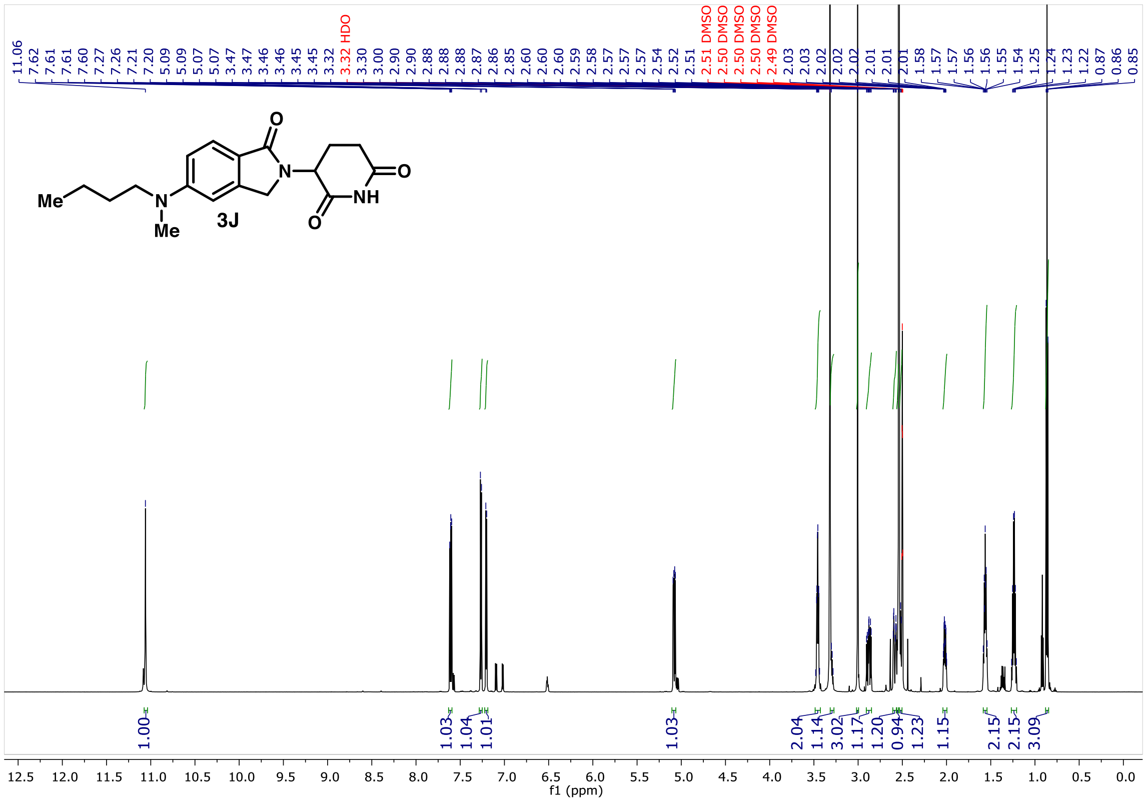

Compound **3J**: ^{13}C NMR (176 MHz, DMSO-d_6)

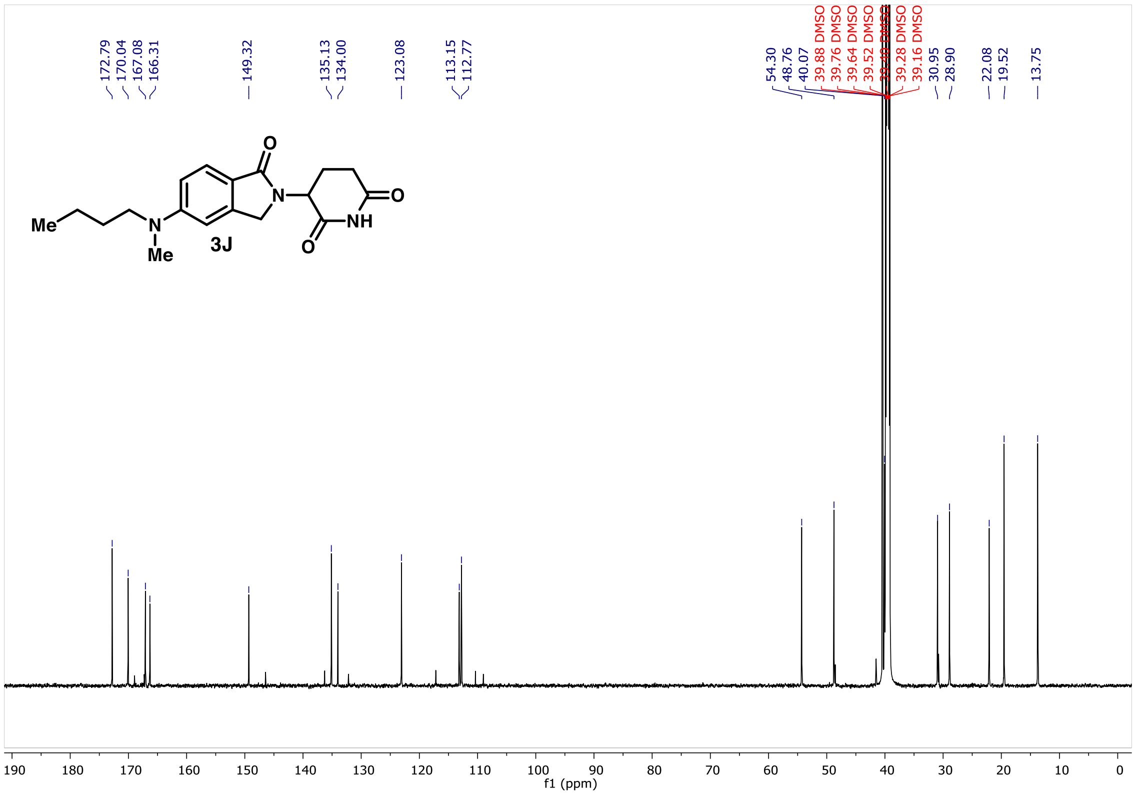

Compound **3K**: ^{1}H NMR (700 MHz, DMSO-d$_6$)

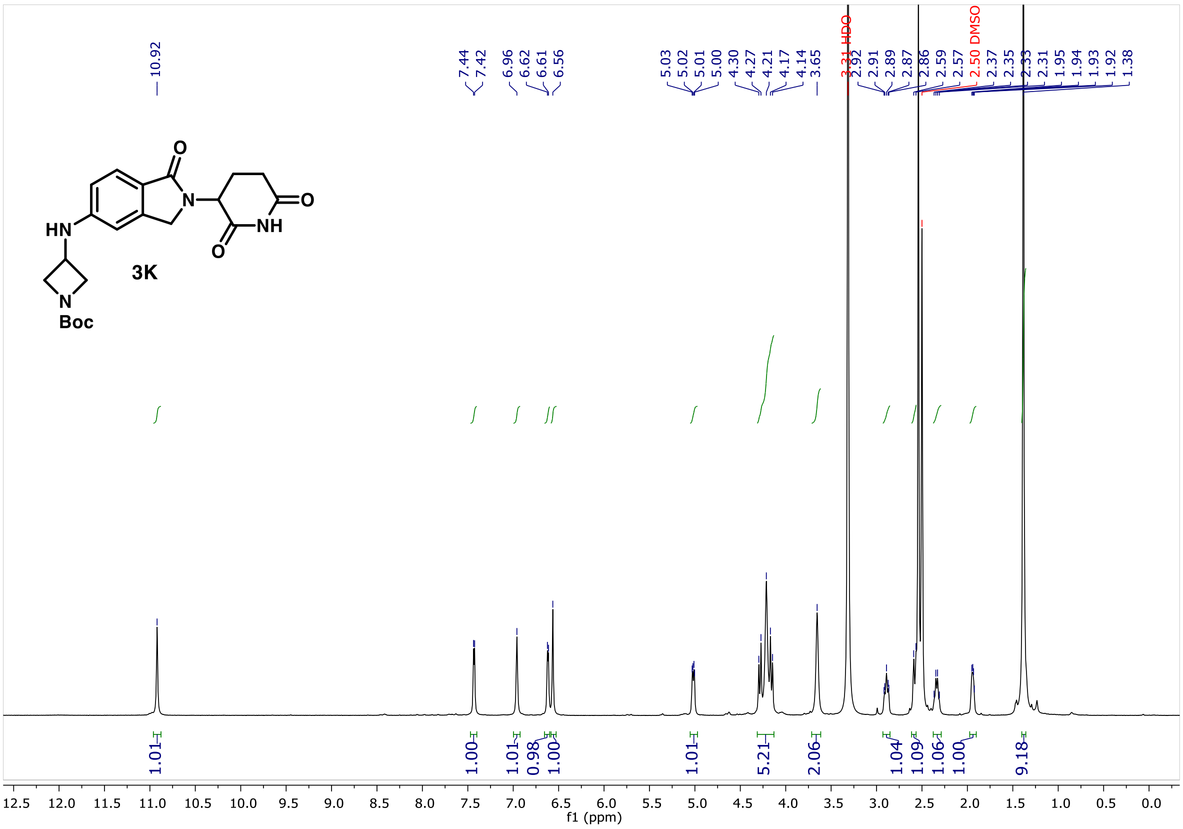

Compound **3K**: ^{13}C NMR (176 MHz, DMSO-d$_6$)

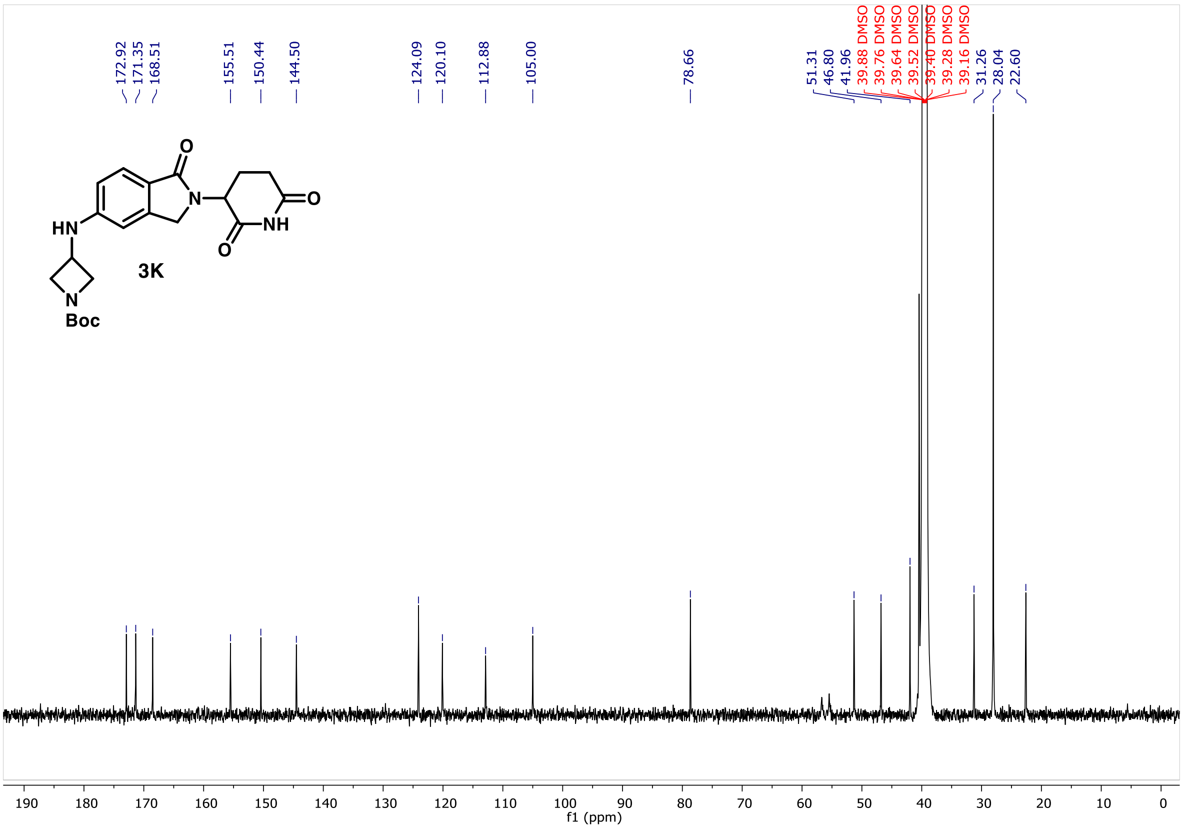

Compound **4A**: ^{1}H NMR (400 MHz, DMSO-d$_6$)

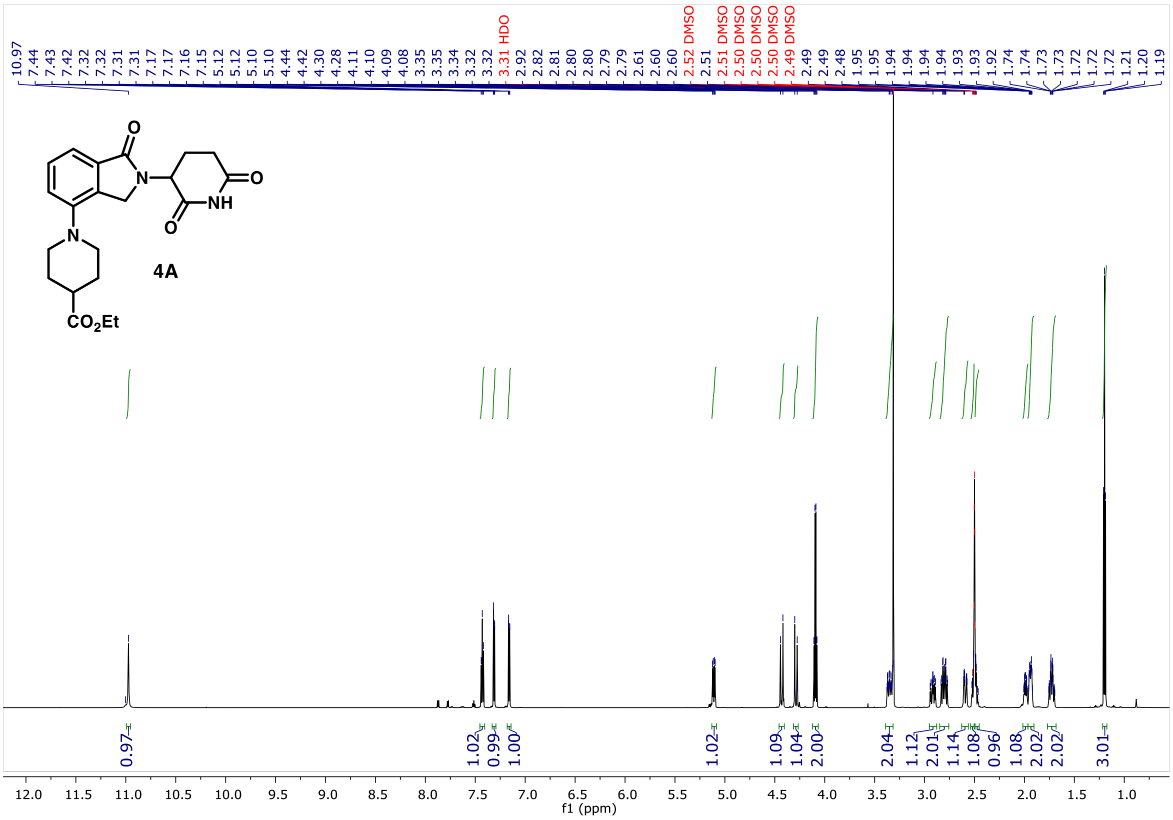

Compound **4A**: ^{13}C NMR (126 MHz, DMSO-d$_6$)

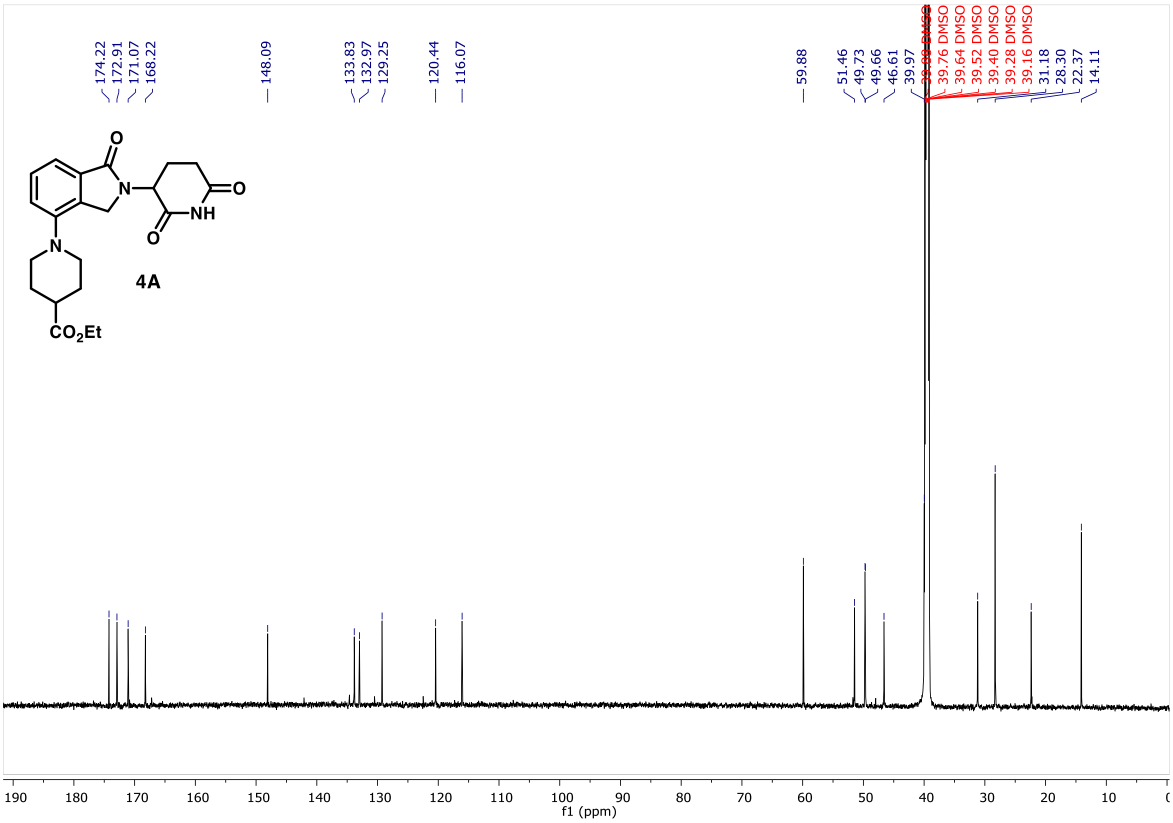

Compound **4B**: ^{1}H NMR (700 MHz, DMSO-d_6)

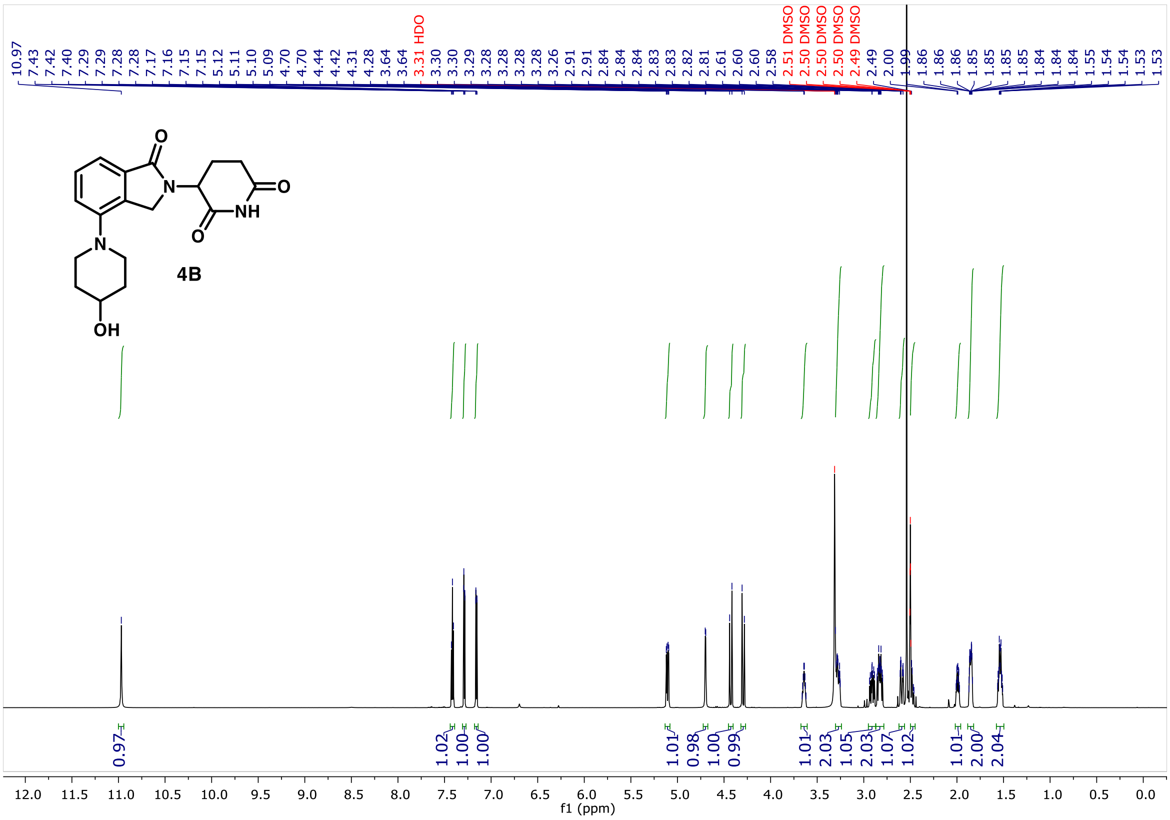

Compound **4B**: ^{13}C NMR (126 MHz, DMSO-d_6)

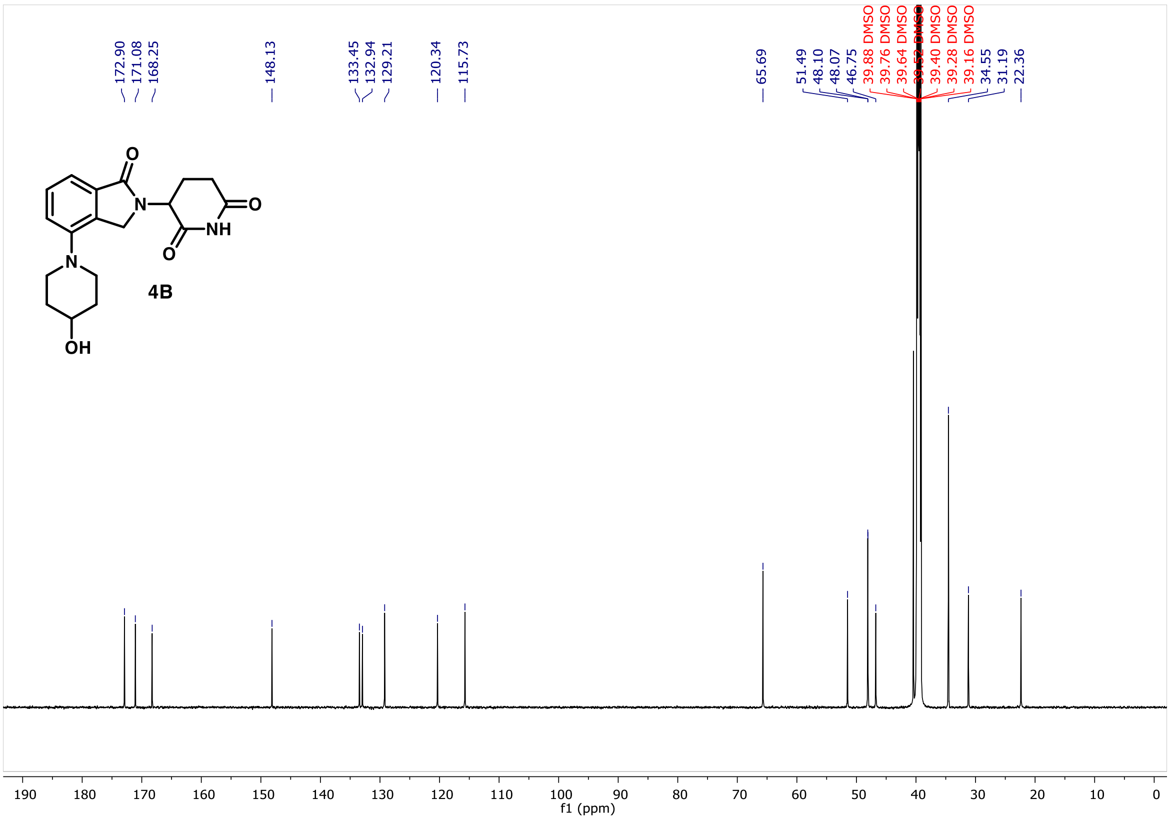

Compound **4C**: ^{1}H NMR (400 MHz, DMSO-d_6)

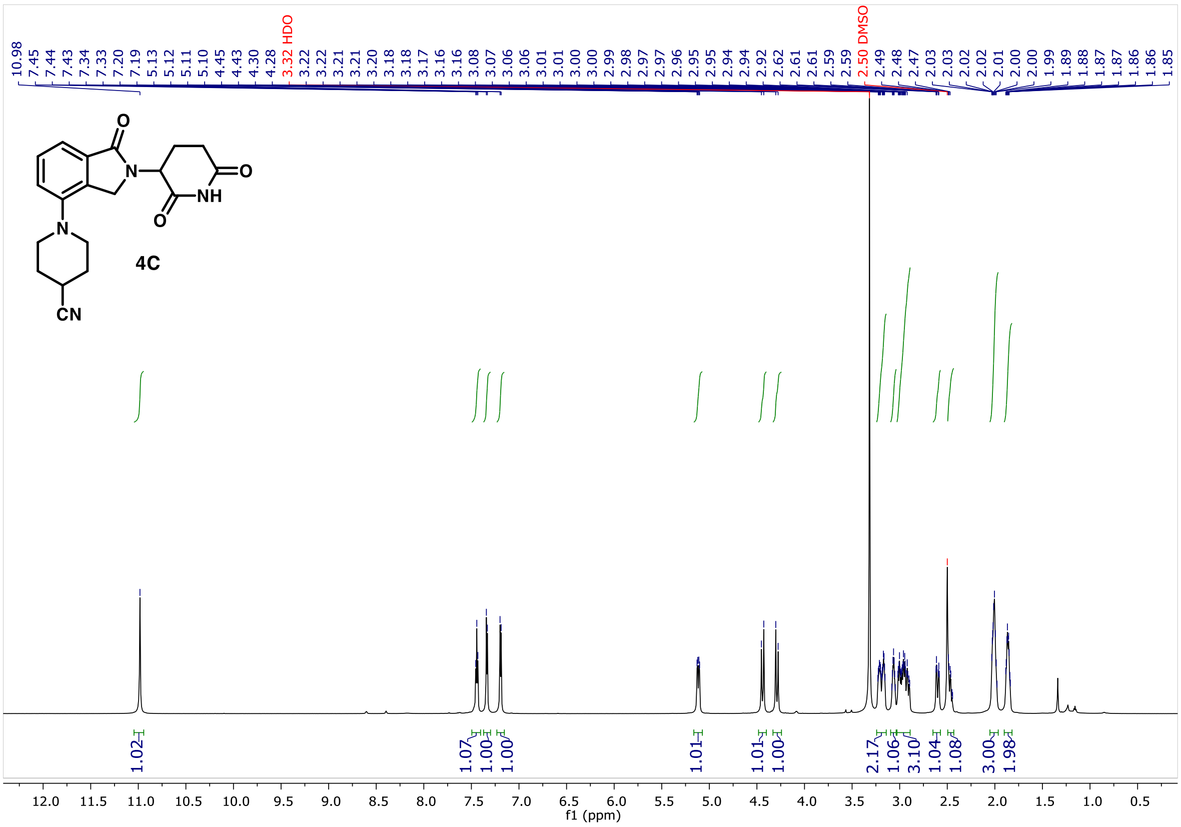

Compound **4C**: ^{13}C NMR (126 MHz, DMSO-d_6)

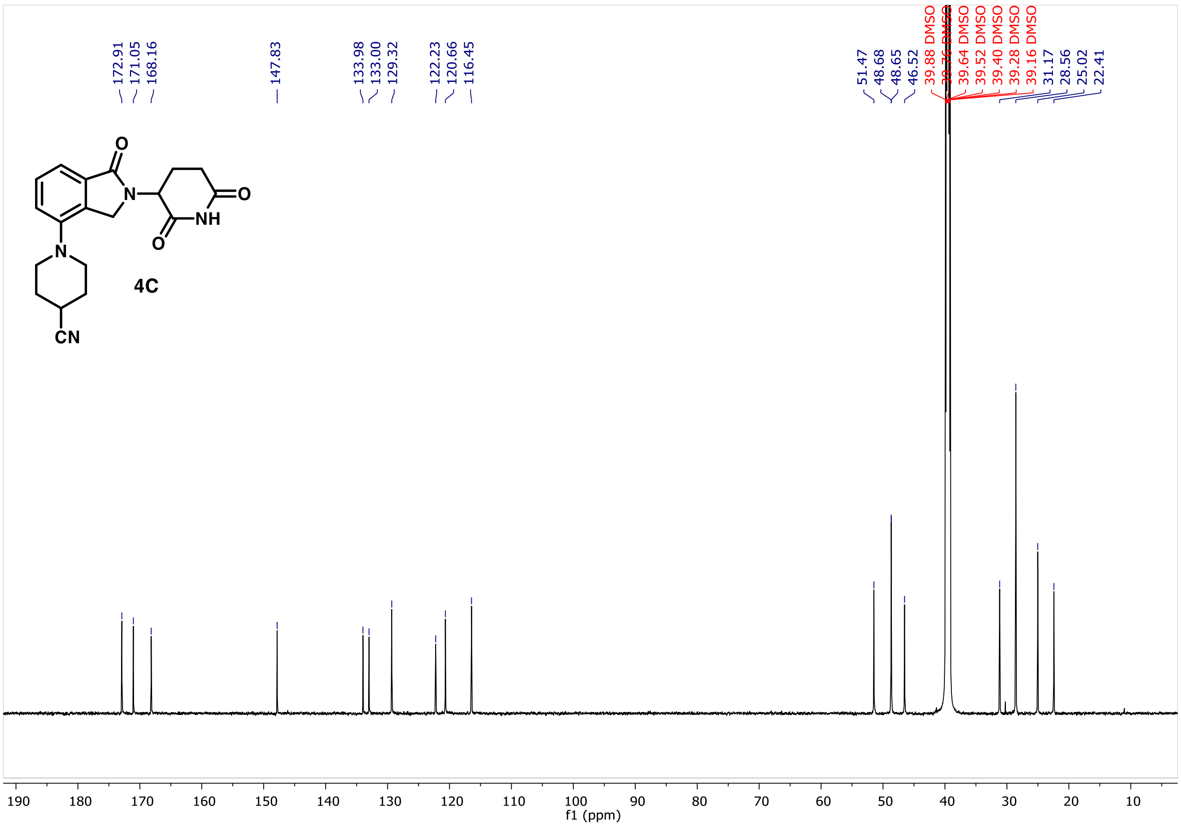

Compound **4D**: ^{1}H NMR (400 MHz, DMSO-d_6)

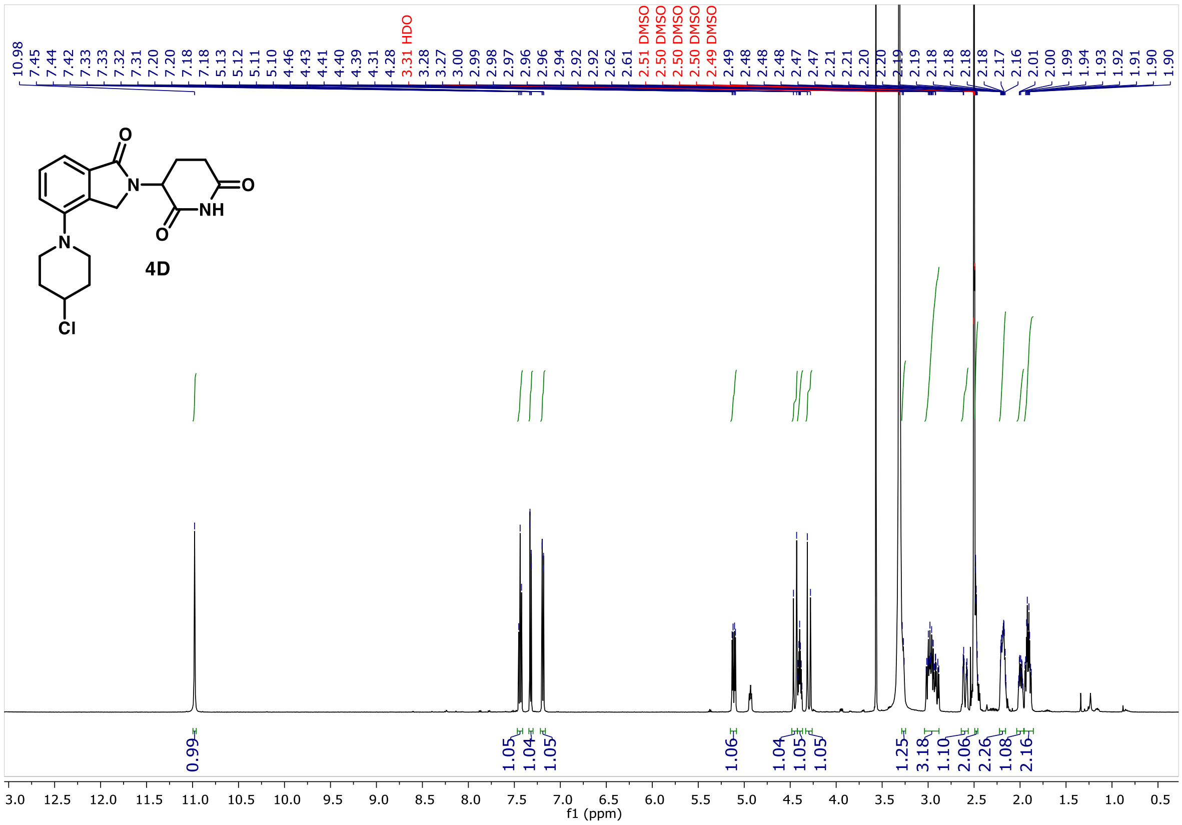

Compound **4D**: ^{13}C NMR (126 MHz, DMSO-d_6)

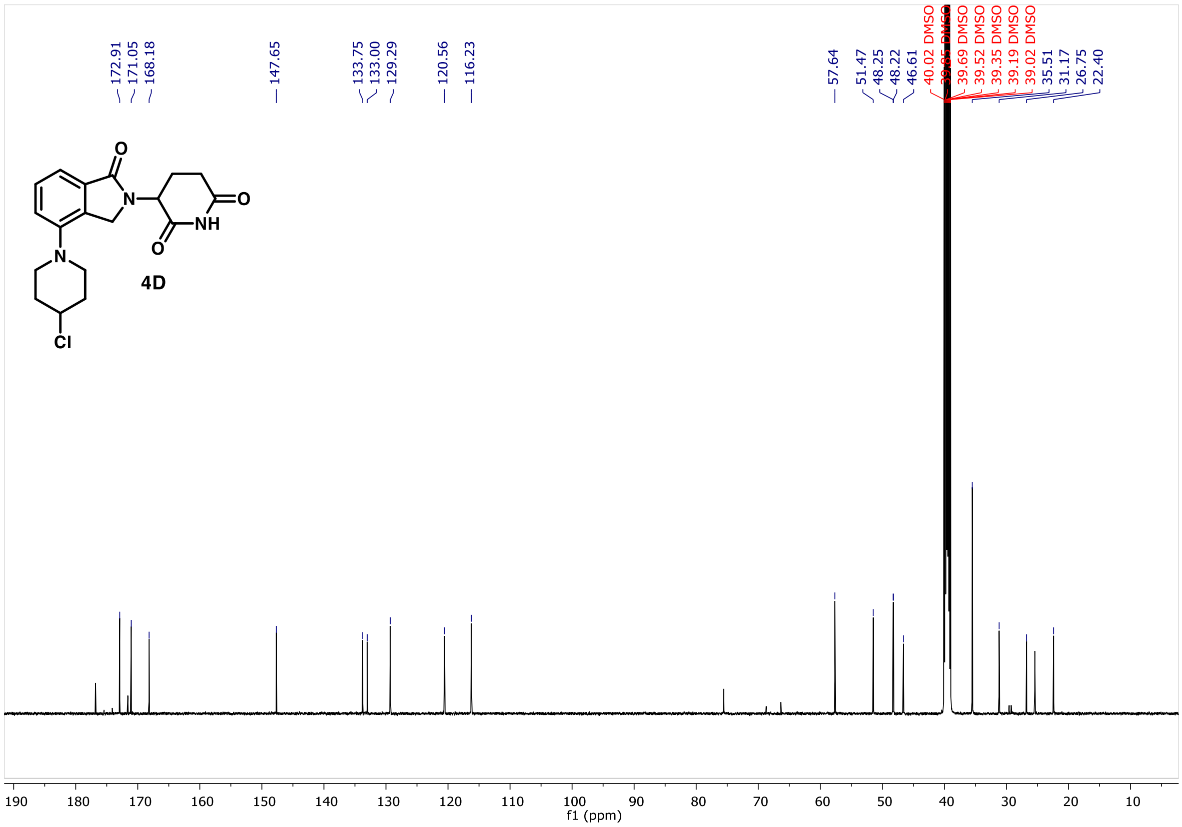

Compound **4E**: ^{1}H NMR (400 MHz, DMSO-d$_6$)

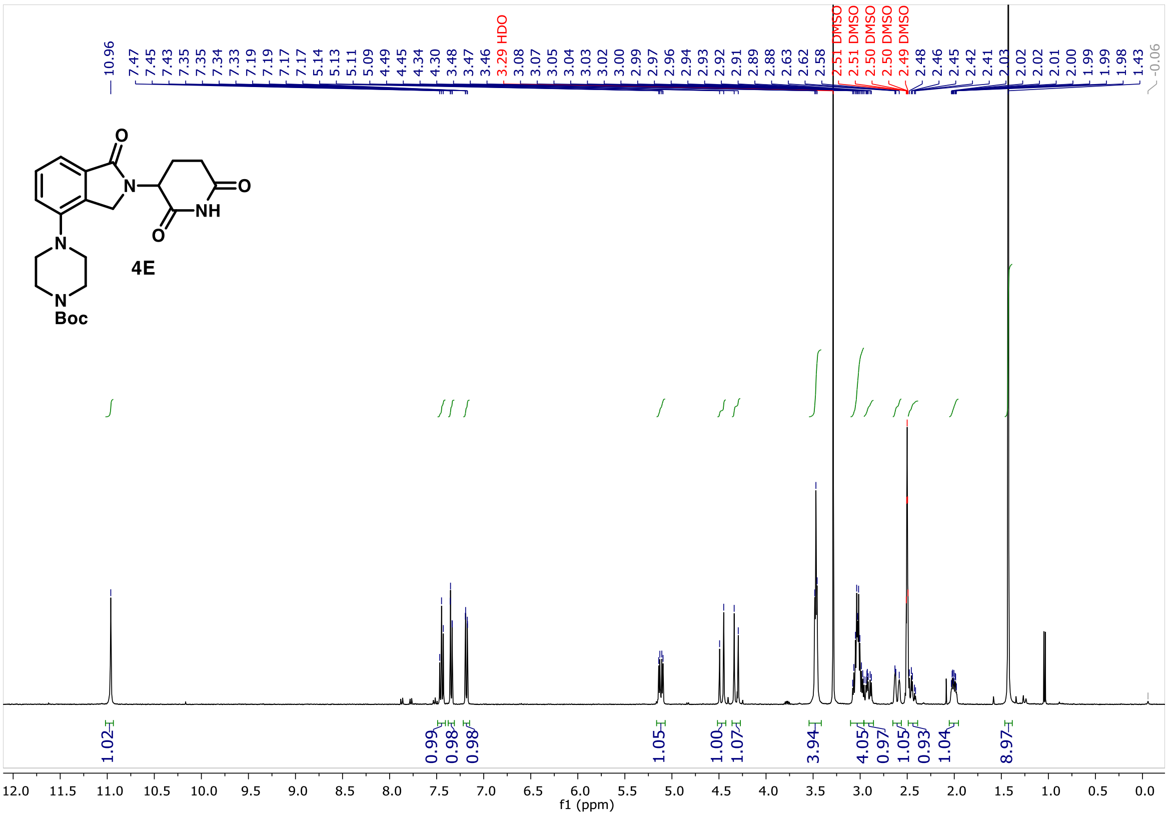

Compound **4E**: ^{13}C NMR (126 MHz, DMSO-d$_6$)

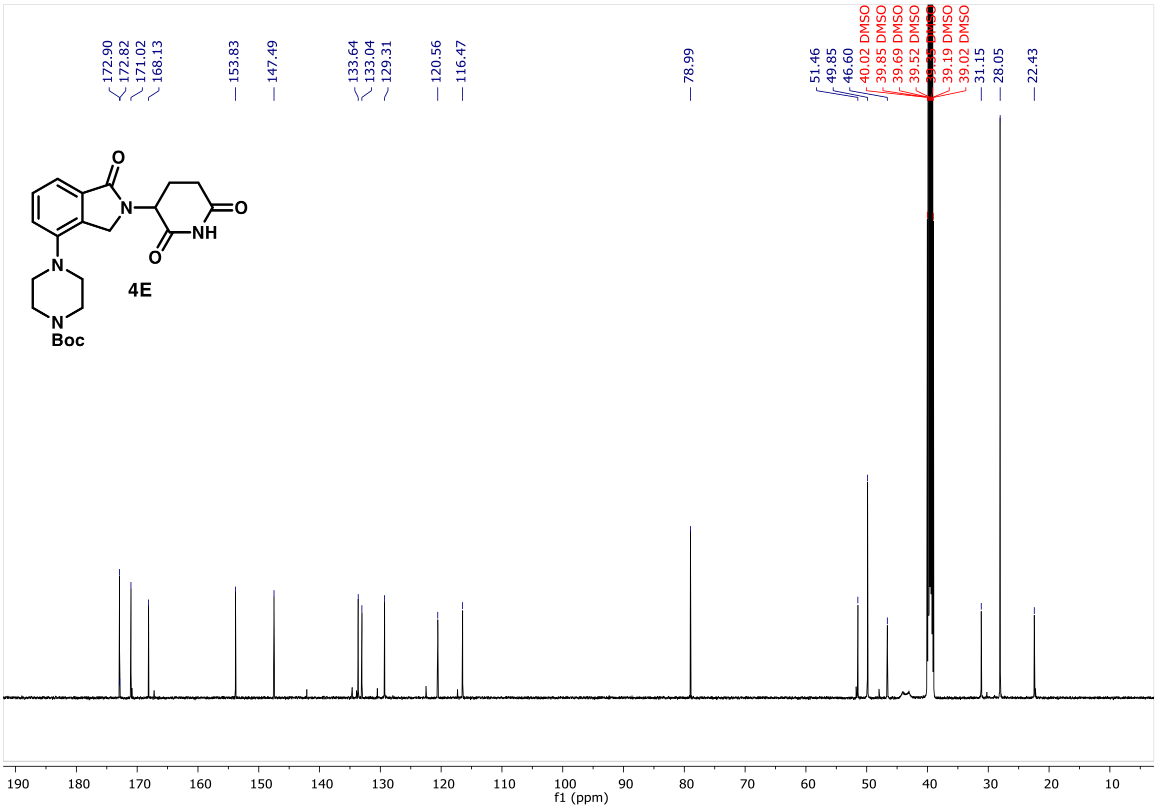

Compound **4F**: ^{1}H NMR (700 MHz, DMSO-d$_6$)

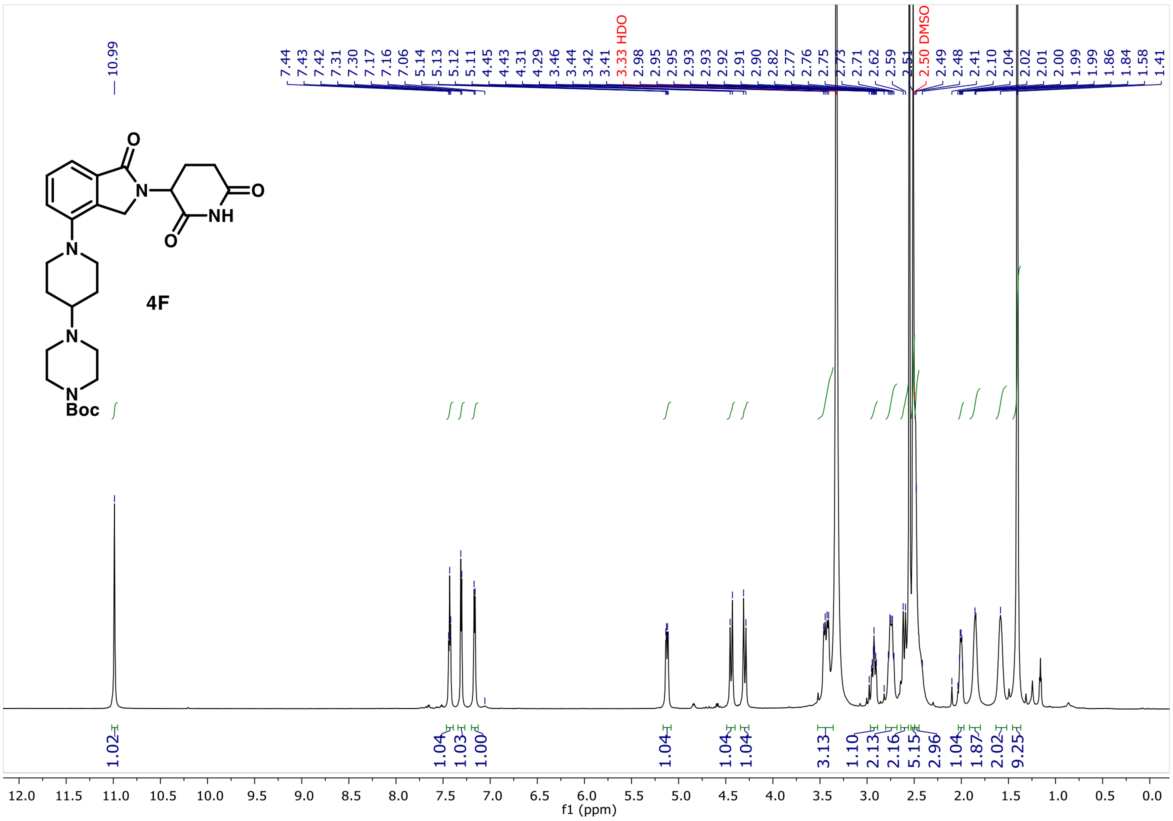

Compound **4F**: ^{13}C NMR (176 MHz, DMSO-d$_6$)

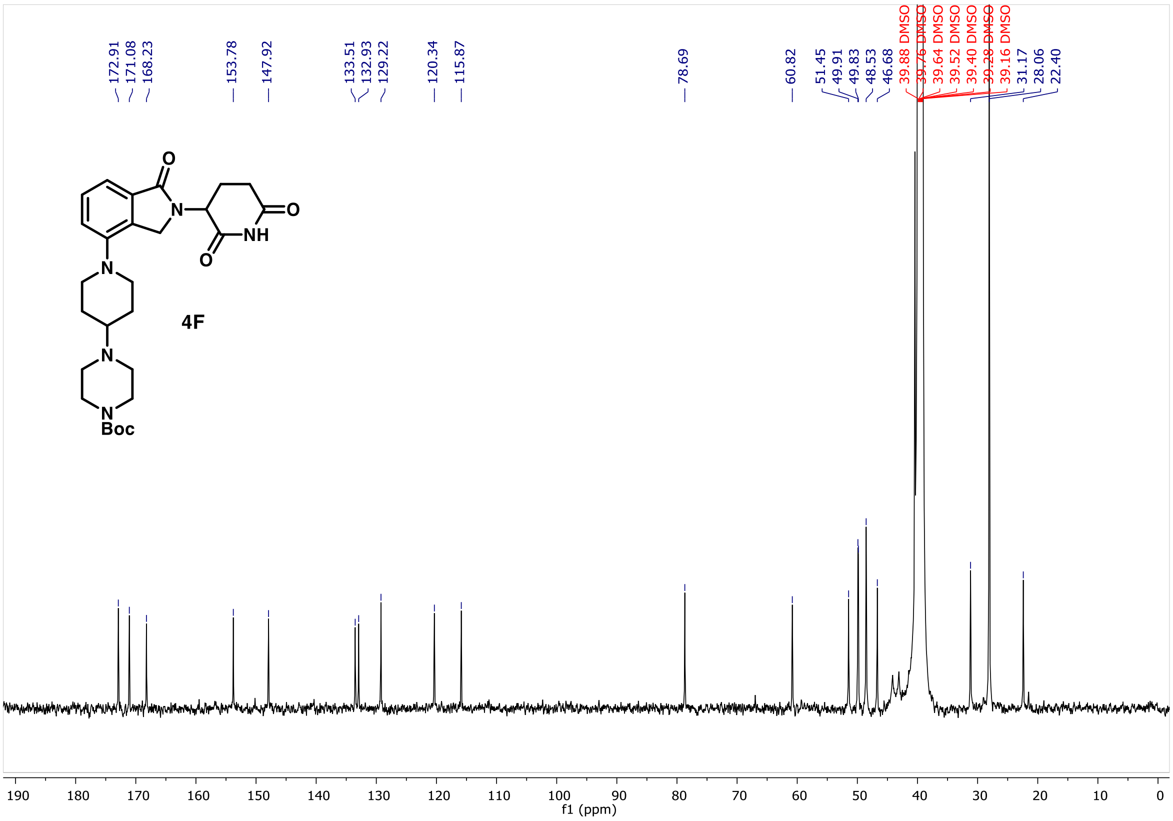

Compound **4G**: ^{1}H NMR (700 MHz, DMSO-d$_6$)

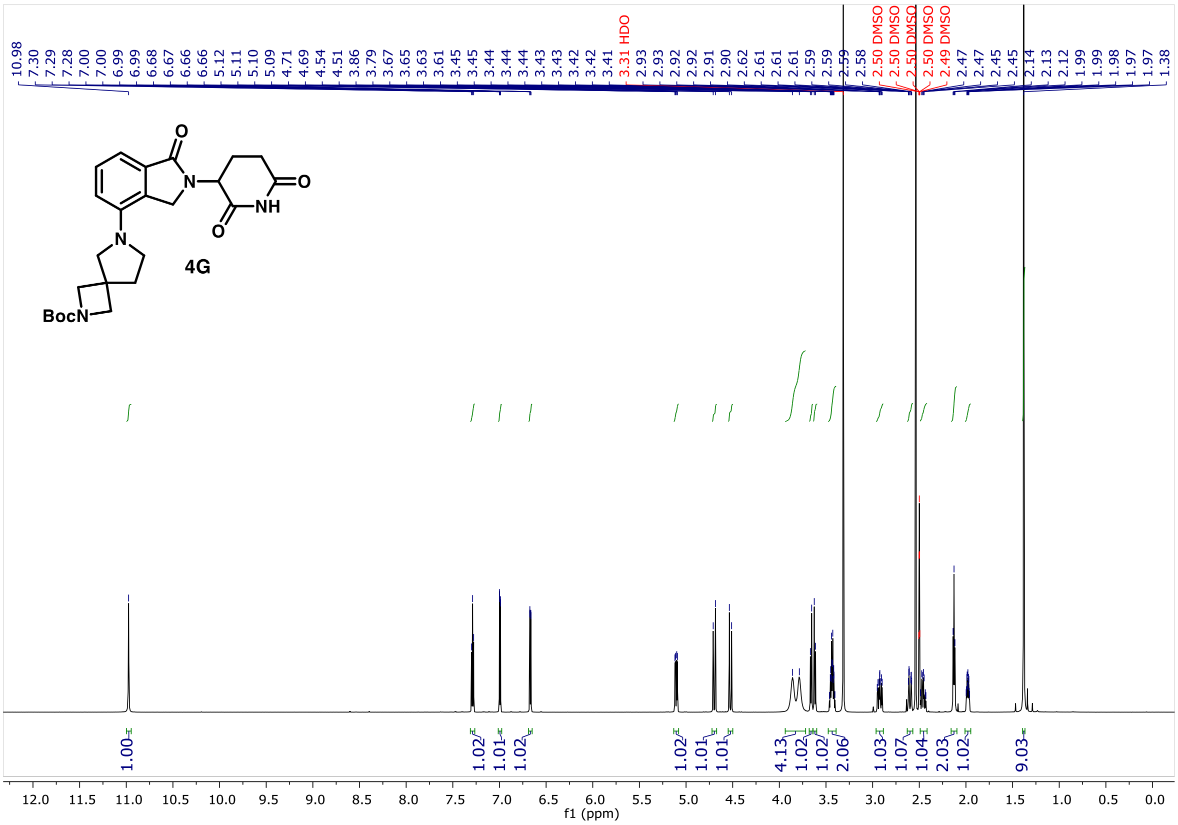

Compound **4G**: ^{13}C NMR (176 MHz, DMSO-d$_6$)

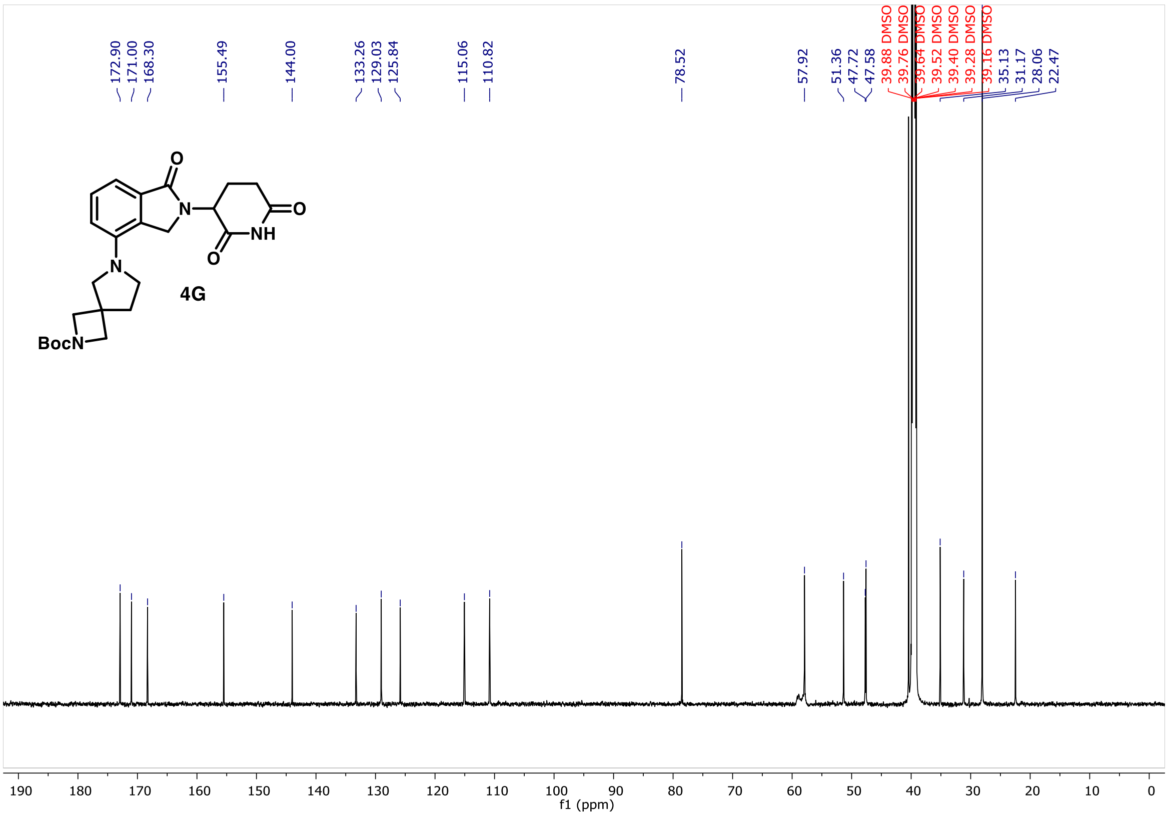

Compound **4H**: ^{1}H NMR (700 MHz, DMSO-d$_6$)

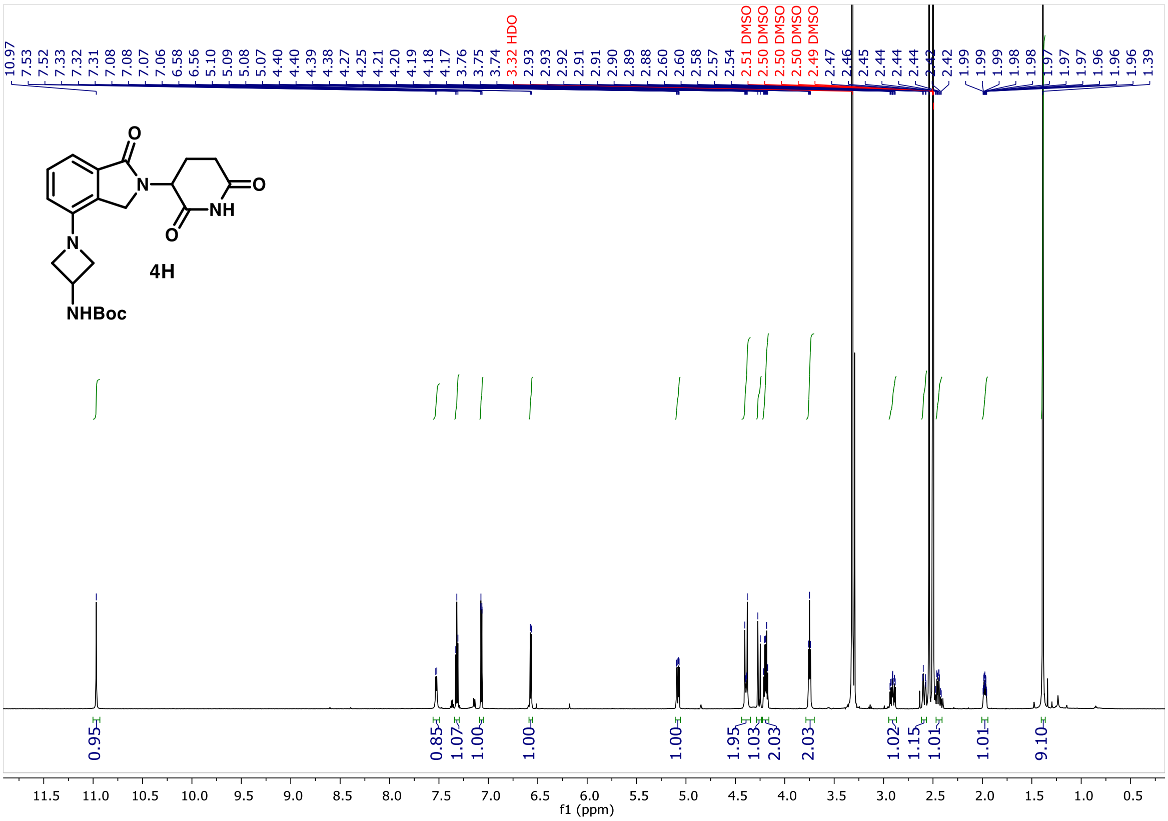

Compound **4H**: ^{13}C NMR (176 MHz, DMSO-d$_6$)

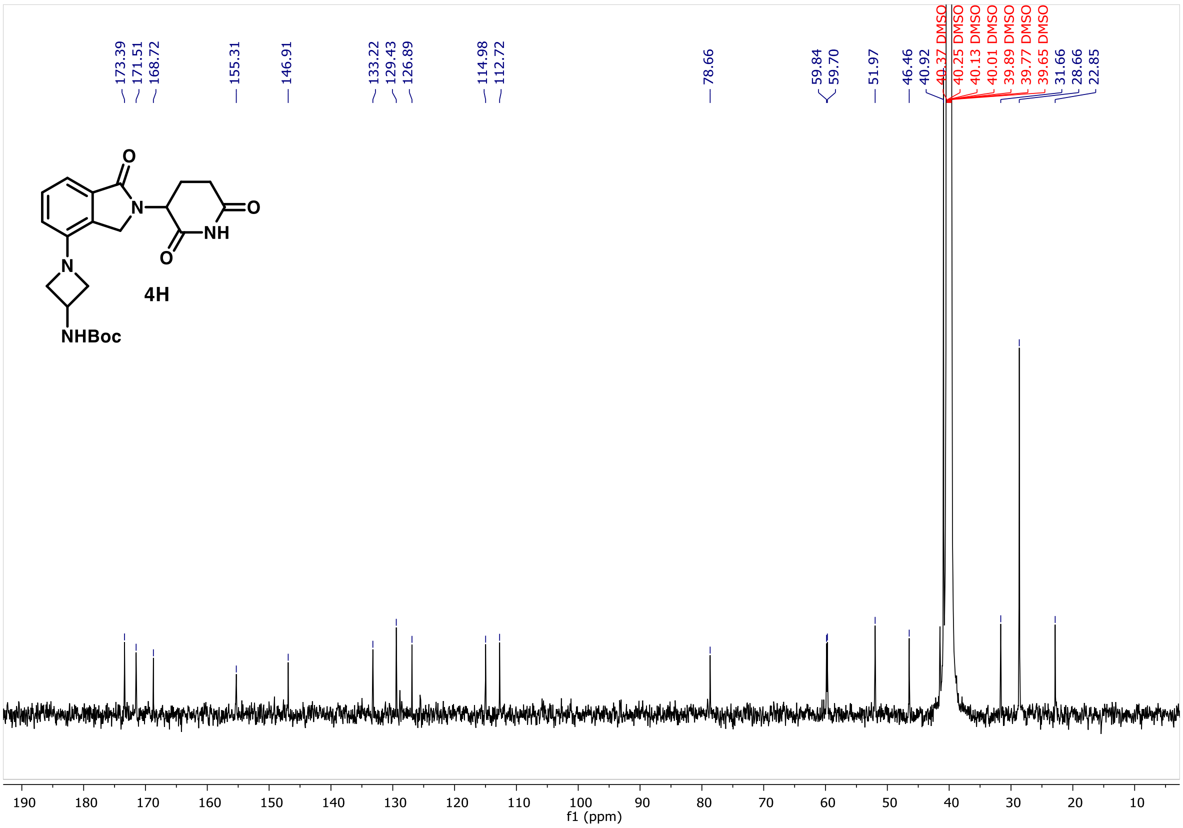

Compound **4J**: ^{1}H NMR (700 MHz, DMSO-d$_6$)

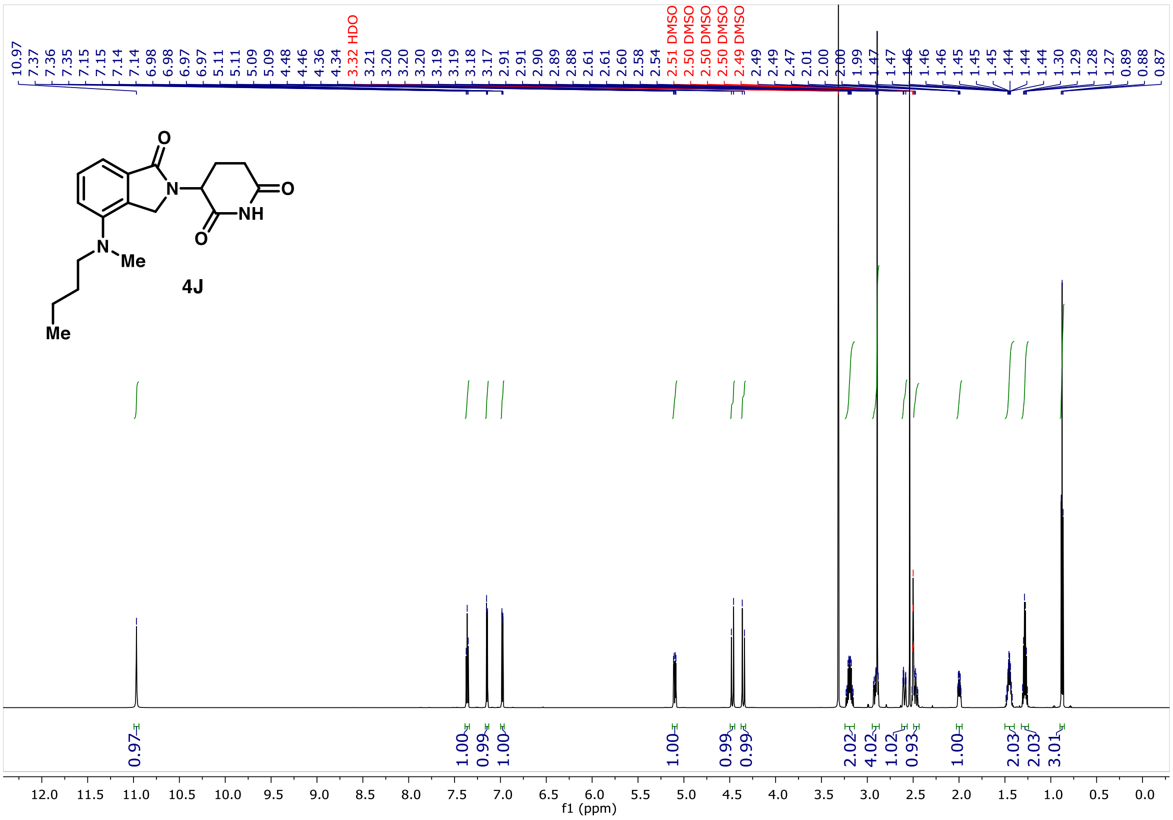

Compound **4J**: ^{13}C NMR (176 MHz, DMSO-d$_6$)

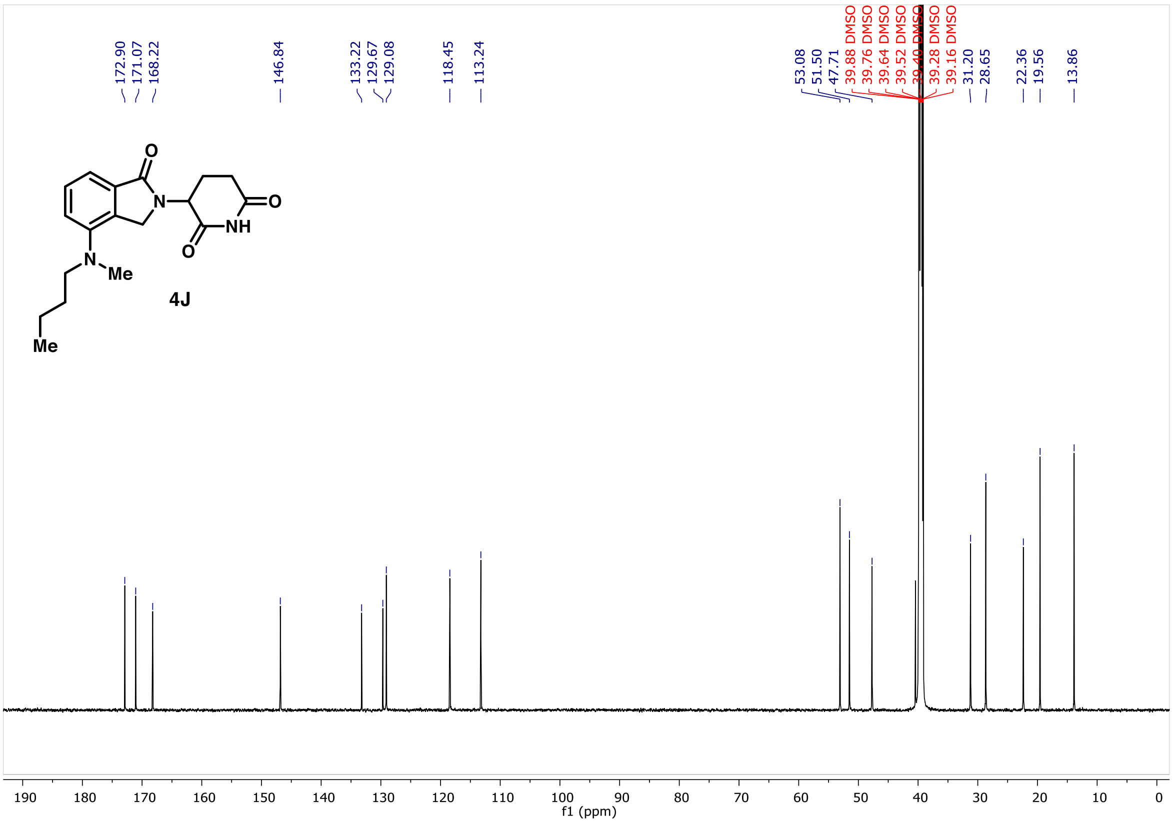

Compound **4K**: ^{1}H NMR (700 MHz, DMSO-d$_6$)

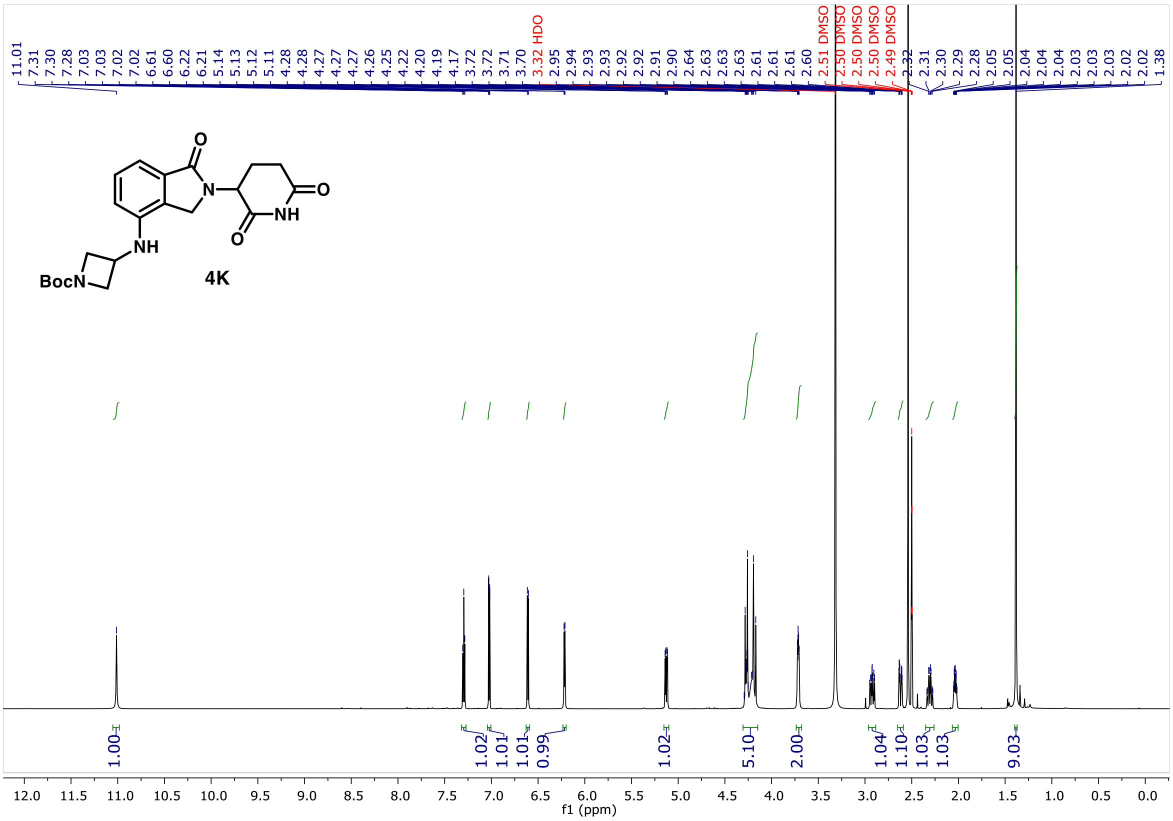

Compound **4K**: ^{13}C NMR (176 MHz, DMSO-d$_6$)

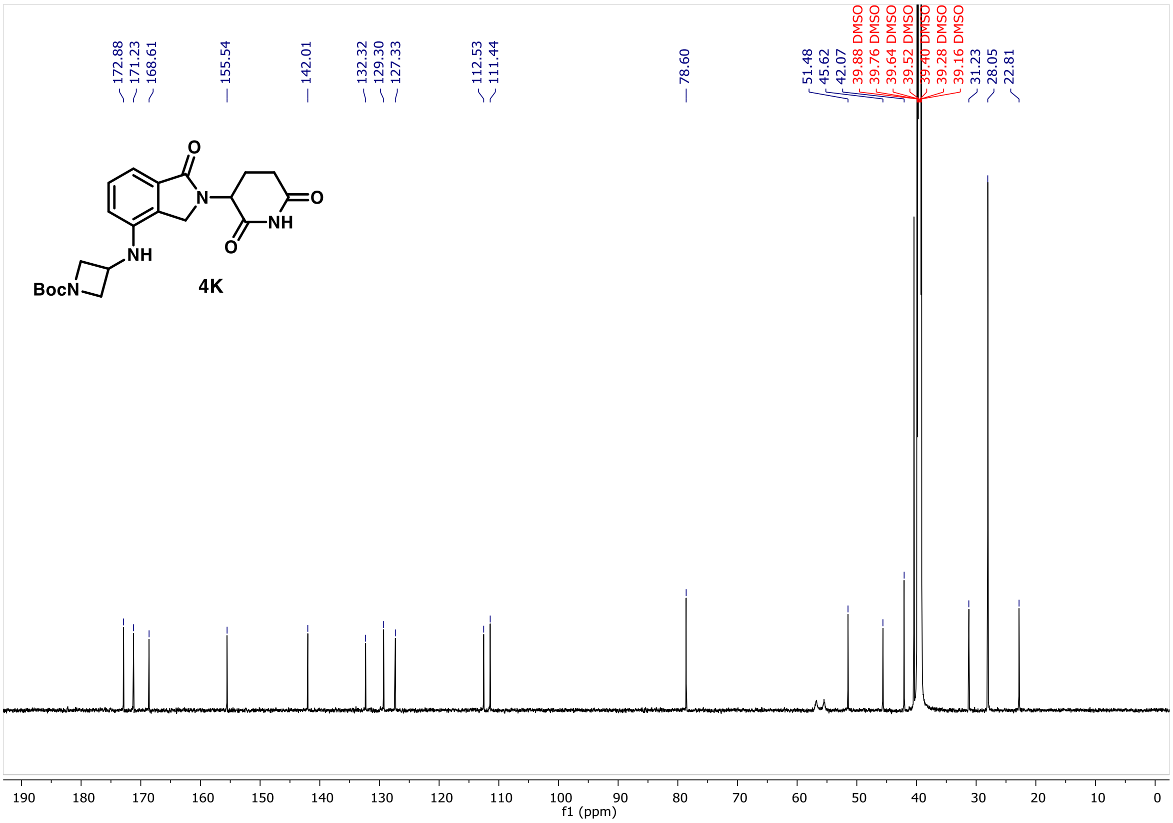

Supplement: Supplementary file 1 — Supporting Information [file ANIE-63-e202412045-s001.pdf]
